# Supplementary material for: Understanding the role of physical activity on the pathway from intra-articular knee injury to post-traumatic osteoarthritis disease in young people: a scoping review protocol
Source: BMJ Open. 2023 Mar 3;13(3):e067147. doi: 10.1136/bmjopen-2022-067147 (PMC9990625; doi:10.1136/bmjopen-2022-067147)
Supplement: Supplementary data [file bmjopen-2022-067147supp001.pdf]

Supplementary Material 1. Preferred Reporting Items for Systematic review and Meta-Analysis Protocols 2015 Checklist[1 2] Adapted for Scoping Reviews[3]

Section and topic	Item No	Checklist item	
**ADMINISTRATIVE INFORMATION**			
Protocol title:			
Identification	1a	Identify the report as a protocol of a scoping review	- Study title<br>- Page 6 of main manuscript
Update	1b	If the protocol is for an update of a previous scoping review, identify as such	Not applicable
Registration	2	If registered, provide the name of the registry (such as Open Science Framework, Figshare or Research Gate) and registration number	- Abstract, page 3 of main manuscript<br>- Methods, page 6 of the main manuscript
Authors:			
Contact	3a	Provide name, institutional affiliation, e-mail address of all protocol authors; provide physical mailing address of corresponding author	Title page, page 1 of main manuscript
Contributions	3b	Describe contributions of protocol authors and identify the guarantor of the review	CRediT statement, page 14 of main manuscript
Amendments	4	If the protocol represents an amendment of a previously completed or published protocol, identify as such and list changes; otherwise, state plan for documenting important protocol amendments	Not applicable
Support:			
Sources	5a	Indicate sources of financial or other support for the review	- Funding statement, page 15 of main manuscript<br>- Acknowledgements, page 15 of main manuscript
Sponsor	5b	Provide name for the review funder and/or sponsor	Funding statement, page 15 of main manuscript
Role of sponsor or funder	5c	Describe roles of funder(s), sponsor(s), and/or institution(s), if any, in developing the protocol	Not applicable

**INTRODUCTION**			
Rationale	6	Describe the rationale for the review in the context of what is already known	Introduction, pages 4-6 of the main manuscript
Objectives	7	Provide an explicit statement of the question(s) the review will address with reference to the inclusion/exclusion criteria	- Introduction, review aims, page 6 of the main manuscript<br>- Methods, framework, stage 1, pages 7-8 of the main manuscript
**METHODS**			
Eligibility criteria	8	Specify the study characteristics (such as PICO/PCC or other, study design, setting, time frame) and report characteristics (such as years considered, language, publication status) to be used as criteria for eligibility for the review	- Table 1., review definitions, page 8-9 of main manuscript<br>- Table 2., review inclusion and exclusion criteria, page 10 of main manuscript
Information sources	9	Describe all intended information sources (such as electronic databases, contact with study authors, trial registers or other grey literature sources) with planned dates of coverage	- Abstract, methods, page 2 of main manuscript<br>- Methods, framework, stage 2, page 9 of main manuscript
Search strategy	10	Present draft of search strategy to be used for at least one electronic database, including planned limits, such that it could be repeated	Supplementary material 2
Study records:			
Data management	11a	Describe the mechanism(s) that will be used to manage records and data throughout the review	- Supplementary material 4, 5, 7, 8<br>- Pages 11-13 of main manuscript
Selection process	11b	State the process that will be used for selecting studies (such as two independent reviewers) through each phase of the review (that is, screening, eligibility, and inclusion)	Pages 11-13 of main manuscript
Data collection process	11c	Describe planned method of extracting data from reports (such as piloting forms, done independently, in duplicate), any processes for obtaining and confirming data from investigators	Pages 11-13 of main manuscript
Data items	12	List and define all variables for which data will be sought (such as PICO/PCC or other items, funding sources), any pre-planned data assumptions and simplifications	Supplementary material 3

Outcomes and prioritization	13	List and define all outcomes for which data will be sought, including prioritization of main and additional outcomes, with rationale (applicable if extracting outcomes)	Supplementary material 3
Risk of bias in individual studies	14	Describe anticipated methods for assessing risk of bias of individual studies, including whether this will be done at the outcome or study level, or both; state how this information will be used in data synthesis	Supplementary material 7
Data synthesis	15a	Describe criteria under which study data will be presented	Methods, framework, stage 4, page 13 of main manuscript
	15b	Describe the planned approach to how, extracted data will be presented (such as figures, tables, evidence gaps maps)	Methods, framework, stage 4 and stage 5, page 13 of main manuscript
	15c	Describe any proposed additional analyses (such as thematic analyses)	Methods, framework, stage 5, pages 13-14 of main manuscript
	15d	If quantitative synthesis is not appropriate, describe the type of summary planned	Methods, framework, stage 5, pages 13-14 of main manuscript
Meta-bias(es)	16	Specify any planned assessment of meta-bias(es) (such as publication bias across studies, selective reporting within studies)	Not applicable
Confidence in cumulative evidence	17	Describe how the strength of the body of evidence will be assessed (such as GRADE)	Methods, framework, stage 5, pages 13-14 of main manuscript

1. Moher D, Shamseer L, Clarke M, et al. Preferred reporting items for systematic review and meta-analysis protocols (PRISMA-P) 2015 statement. *Syst Rev* 2015;4(1):1. doi: 10.1186/2046-4053-4-1
2. Shamseer L, Moher D, Clarke M, et al. Preferred reporting items for systematic review and meta-analysis protocols (PRISMA-P) 2015: elaboration and explanation. *BMJ* 2015;349(jan02 1):g7647-g47. doi: 10.1136/bmj.g7647
3. Peters MDJ, Godfrey C, McInerney P, et al. Best practice guidance and reporting items for the development of scoping review protocols. *JBI Evid Synth* 2022;20(4)
