# Supplementary material for: Understanding the role of physical activity on the pathway from intra-articular knee injury to post-traumatic osteoarthritis disease in young people: a scoping review protocol
Source: BMJ Open. 2023 Mar 3;13(3):e067147. doi: 10.1136/bmjopen-2022-067147 (PMC9990625; doi:10.1136/bmjopen-2022-067147)
Supplement: Supplementary data [file bmjopen-2022-067147supp002.pdf]

PubMed Search Strategy

The search terms are structured as follows:

Population AND independent variables (e.g., physical activity, adiposity, knee joint loading) AND outcome variables (e.g., osteoarthritis, biomarkers of cartilage or bone metabolism)

Search Terms

(("knee injur*" OR "cruciate ligament injur*" OR "cruciate ligament tear" OR "cruciate ligament damage*" OR "cruciate ligament rupture*" OR “injured cruciate ligament” OR "torn cruciate ligament" OR “damaged cruciate ligament” OR “ruptured cruciate ligament” OR “ACL injury” OR “ACL tear” OR “ACL damage” OR “ACL rupture” OR “injured ACL” OR “torn ACL” OR “damaged ACL” OR “ruptured ACL” OR “PCL injury” OR “PCL tear” OR “PCL damage” OR “PCL rupture” OR “injured PCL” OR “torn PCL” OR “PCL damage” OR “ruptured PCL” OR "cruciate ligament reconstruct*" OR "cruciate ligament surger*" OR “cruciate ligament repair” OR “ACL reconstr*” OR “ACL surger*” OR “PCL reconstr*” OR “PCL surger*” OR "meniscus injur*" OR "meniscal injur*" OR "meniscus tear" OR "meniscal tear" OR "meniscus damage" OR "meniscal damage" OR "meniscus rupture" OR "meniscal rupture" OR “injured menisc*” OR “torn menisc*” OR "meniscus repair" OR “repaired menis*” OR "meniscus surger*" OR Anterior Cruciate Ligament Injuries[MeSH Terms] OR Anterior Cruciate Ligament Reconstruction[MeSH Terms] OR Posterior Cruciate Ligament Reconstruction[MeSH Terms] OR Tibial Meniscus Injuries[MeSH Terms] OR Meniscectomy[MeSH Terms]) AND ("physical activit*" OR "physical inactivit*" OR exercis* OR "rehab*" OR train* OR inflamma* OR "c reactive protein" OR cytokine* OR interleukin OR "reaction force*" OR "contact force*" OR "knee load*" OR "cumulative load*" OR "knee adduction moment" OR "knee abduction moment" OR "knee flexion moment" OR "knee extension moment" OR "contact pressure" OR "ground reaction force*" OR "musculoskeletal model*" OR "inverse dynamics" OR simulat* OR "muscle strength" OR "muscle contract*" OR neurophysiolog* OR neuromuscular OR "body composition" OR "body mass inde*" OR adipos* OR obes* OR fat OR "muscle quality" OR "echo intensity" OR echogenicity OR "inter-muscular adipose" OR "intermuscular adipose" OR "intermuscular fat" OR "intra-muscular adipose" OR "intramuscular adipose" OR "intra-muscular fat" OR "intramuscular fat" OR "muscle attenuation" OR "muscle density" OR "muscle mass" OR "muscle volume" OR "muscle cross-sectional area" OR "muscle thickness" OR "muscle atrophy" OR "bone mineral" OR "bone density" OR Exercise[MeSH Terms] OR Sedentary Behaviour[MeSH Terms] OR Rehabilitation[MeSH Terms] OR Inflammation[MeSH Terms] OR Cytokines[MeSH Terms] OR Interleukins[MeSH Terms] OR Muscle Strength[MeSH Terms] OR Muscle Contraction[MeSH Terms] OR Muscle Weakness[MeSH Terms] OR Body Composition[MeSH Terms] OR Body Mass Index[MeSH Terms] OR Subcutaneous Fat[MeSH Terms] OR Adipose Tissue[MeSH Terms] OR Obesity[MeSH Terms] OR Leptin[MeSH Terms] OR Adiponectin[MeSH Terms] OR Muscular Atrophy[MeSH Terms] OR Bone Density[MeSH Terms]) AND (osteoarthr* OR chondrosis OR “degenerative arthritis” OR "t1 relaxation time" OR t1rho OR "t2 relaxation time" OR t2 OR "delayed gadolinium-enhanced magnetic resonance imaging of cartilage" OR dGEMRIC OR “cartilage volume” OR "cartilage thickness" OR matrix OR MMP OR collagen OR “pro-collagen” OR procollagen OR collagenase OR gelatinase OR proteoglycan OR hyaluronic OR “keratin

sulphate" OR chondroitin OR pyridinoline OR "alkaline phosphatase bone isoenzyme" OR "bone alkaline phosphatase" OR "tartrate-resistant acid phosphatase 5b" OR osteocalcin OR deoxypyridinoline OR osteophyte* OR "bone remodel*" OR "bone regenerat*" OR 'insulin growth factor 1' OR 'transforming growth factor beta' OR gene* OR allele OR polymorphism OR variant OR expression OR proteom* OR "mass spectrometry" OR stromelysin OR 'a disintegrin and metalloproteinase with thrombospondin motifs' OR ADAMTS OR metaboli* OR biomarker* OR "blood marker" OR "urine marker" OR "synovial fluid marker" OR biochemical OR Osteoarthritis, Knee[MeSH Terms] OR Fibrillar Collagens[MeSH Terms] OR Proteoglycans[MeSH Terms] OR Bone Remodelling[MeSH Terms] OR Bone Regeneration[MeSH Terms] OR Osteogenesis[MeSH Terms] OR Osteophytes[MeSH Terms] OR Biomarkers[MeSH Terms]) NOT (Review[Publication Type] OR Systematic Review[Publication Type] OR Meta Analysis[Publication Type] OR Scoping Review[Publication Type] OR Aged[MeSH Terms] OR Arthroplasty, Replacement, Knee[MeSH Terms] OR (animals[mh] NOT humans[mh])))

Filters Applied: Results by Year: 1970-2023, Species: Human, Language: English

Number of Records: 2,810
