# Supplementary material for: Understanding the role of physical activity on the pathway from intra-articular knee injury to post-traumatic osteoarthritis disease in young people: a scoping review protocol
Source: BMJ Open. 2023 Mar 3;13(3):e067147. doi: 10.1136/bmjopen-2022-067147 (PMC9990625; doi:10.1136/bmjopen-2022-067147)
Supplement: Supplementary data [file bmjopen-2022-067147supp003.pdf]

Supplementary Material 3. PTOA Disease Outcomes

This list of outcome measures is not exhaustive and will be added to if other relevant outcomes are identified during Stage 2 - Identifying Relevant Studies.

Table 1. Disease Outcome Measures

**Diagnoses of Knee PTOA Disease**	
**Radiographic Diagnostic System**	**Scoring System**
Kellgren and Lawrence (K/L) system[1]	A K/L score of 1 indicates early knee OA and an >1 K/L score indicates knee OA[1]
Osteoarthritis Research Society International (OARSI) Atlas criteria[2]	OARSI Atlas scores for knee OA diagnosis are joint space narrowing ≥ grade 2, or grade 1 joint space narrowing occurred in combination with grade 1 osteophyte[2]
International Knee Documentation Committee (IKDC) System[3]	An IKDC score from C-D indicates reduction in joint space, development of small osteophytes, slight sclerosis, or femoral condyle flattening[3]
Ahlback System[4]	An Ahlback score of >0 indicates development of osteoarthritic features[4]
Fairbank Classification[5]	A Fairbank classification of >0 indicates development of osteoarthritic features[5]
Brandt Grading Scale[6]	
Jager-Wirth Classification[7]	A Jager-Wirth Classification of >0 indicates development of osteoarthritic features[7]
**Magnetic Resonance Imaging Diagnostic System**	**Scoring System**
Magnetic Resonance Imaging Osteoarthritis Knee Score (MOAKS)[8]	For brevity due to the multi-tissue assessment lower scores for each tissue inside the joint indicates degeneration.[8]
Whole Organ Magnetic Resonance Imaging Score (WORMS)[9]	For brevity due to the multi-tissue assessment, lower scores for each tissue inside the joint indicates degeneration.[9]
Boston Leeds OA Knee Score (BLOKS)[10]	For brevity due to the multi-tissue assessment, lower scores for each tissue inside the joint indicates degeneration.[10]

Indicators of Structural Deterioration Preceding PTOA Disease

Measurement Tool	Index
Magnetic resonance imaging (MRI) quantified cartilage thickness and volume[11]	Lower MRI cartilage thickness and volume indicates degenerated cartilage[11 12]
T1rho mapping[12]	Higher signal intensity of T1rho mapping indicates lower collagen and/or proteoglycan content[12 13]
T2 mapping[14]	Higher signal intensity of T2 mapping indicates lower collagen and/or proteoglycan content[12 13]
Delayed contrast-enhanced MRI of cartilage (dGEMRIC)[15]	Higher signal intensity for dGEMRIC is indicative of low glycosaminoglycan (GAG) content[12 15]
Ultrasonography (US) quantified cartilage thickness[16-18]	Lower US cartilage thickness and volume indicates degenerated cartilage[16-18]

Systemic Biomarkers Indicating Structural Deterioration of Knee PTOA Disease

Substrate Biomarkers	Index
Matrix Metalloprotease 3 (MMP-3)[19]	Serum MMP-3 concentration positively associated with knee OA and cartilage volume loss[20-22]
Matrix Metalloprotease 9 (MMP-9)[23]	Serum and synovial fluid MMP-9 concentration positively associated with OA diagnosis[24]
Matrix Metalloprotease 13 (MMP-13) [25-29]	Serum MMP-13 concentration negatively associated with knee cartilage volume and positively associated with knee OA diagnosis[22 30]
A Disintegrin and Metalloproteinase with Thrombospondin Motifs 4 (ADAMTS-4)[31]	Serum ADAMTS-4 concentration positively associated with knee OA diagnosis[32]
Tissue Inhibitor of Metalloproteinase 1 (TIMP-1)[33]	Plasma TIMP-1 concentration positively associated with OA severity[34]
**Collagen Biomarker**	**Index**
N-Propeptide of Collagen IIA (PIIANP)[35]	Serum PIIANP concentration negatively associated with knee osteophytes[24] and knee OA[35 36]
N-Propeptide of Collagen IIB (PIIBNP)[37]	Serum and Plasma concentrations negatively associated with joint space narrowing[38]
C-Telopeptide of Type II Collagen (CTX-II)[39]	Urinary concentration of CTX-II positively associated with knee OA diagnosis and cartilage degeneration[21 40-44]
C-Propeptide of Type II Collagen (CP-II) [45]	Serum concentration of CP-II negatively associated with knee OA diagnosis[45 46]

CTX-II:CP-II[46]	Ratio of urinary concentration of CTX-II to serum concentrations of CP-II positively associated with knee OA[35 46]
Collagen Type II Cleavage Product (C2C)[47]	Serum and urine C2C concentration positively associated with cartilage degeneration and knee joint pain worsening and joint space narrowing[48-50]
Col2-3/4 C-terminal cleavage product of types I and II collagen (C1,2C)[25]	Serum C1,2C concentration positively associated with knee OA diagnosis[50]
Neoepitope of Collagen X (C10C or Col10Neo)[50]	Serum C10C concentration positively associated with knee OA diagnosis[51] and plasma C10C/Col10Neo concentration positively associated with knee OA diagnosis[52]
**Proteoglycan Biomarker**	**Index**
Hyaluronic Acid (HA)[53]	Serum HA concentration positively associated with knee OA diagnosis and joint space narrowing[21 48 54-56]
Chondroitin Sulphate 846 Epitope (CS846)[57]	Serum CS846 concentration positively associated with femur cartilage thickening [58 59]
Aggrecan[60] or Aggrecan Fragments (ARGS)[61 62]	Serum aggrecan concentration at baseline inversely associated with joint space narrowing in medial compartment after 10 years of follow-up[63] and synovial fluid ARGS concentration negatively associated with joint space narrowing[52 64]
Cartilage Oligomeric Matrix Protein (COMP)[53 65]	Serum COMP concentration positively associate with knee OA diagnosis and cartilage degeneration[21 41 43 44 50 63 66-70]
**Bone Formation**	**Index**
Osteocalcin (OC)[71]	Serum increase in OC concentration over a year positively associated with knee OA severity progression over three years[72]
Bone Specific Alkaline Phosphate (BAP) or alkaline phosphatase (ALP)[73]	Serum ALP concentrations positively associated with knee OA severity[74]
Procollagen Type I N-Terminal Propeptide (PINP)[75]	Baseline PINP serum concentration predicts OA progression[76]
**Bone Resorption**	**Index**
Pyridinoline (PYD)[71 77]	Urinary PYD concentration negatively associated with the joint space width and OA severity[34]
Deoxypyridinoline (DPD)[71]	Synovial fluid DPD concentration positively associated with disease severity[78]
N-Telopeptide of Type I Collagen (NTX-I)[79 80]	Urinary and serum NTX-I concentration positively associated with knee OA severity progression[48 81]
C-Telopeptide of Type I Collagen (CTX-I)[79]	Urinary and serum CTX-I concentration positively associated with knee OA severity progression[48]

BMJ Publishing Group Limited (BMJ) disclaims all liability and responsibility arising from any reliance placed on this supplemental material which has been supplied by the author(s)

References

1. Kellgren JH, Lawrence JS. Radiological Assessment of Osteo-Arthrosis. *Ann Rheum Dis* 1957;16(4):494-502. doi: 10.1136/ard.16.4.494
2. Altman RD, Gold GE. Atlas of individual radiographic features in osteoarthritis, revised. *Osteoarthritis Cartilage* 2007;15:A1-A56. doi: 10.1016/j.joca.2006.11.009
3. Hefti E, Müller W, Jakob RP, et al. Evaluation of knee ligament injuries with the IKDC form. *Knee Surgery, Sports Traumatology, Arthroscopy* 1993;1(3-4):226-34. doi: 10.1007/bf01560215
4. Ahlbäck S. Osteoarthrosis of the knee. A radiographic investigation. *Acta Radiol Diagn (Stockh)* 1968:Suppl 277:7-72.
5. Fairbank TJ. KNEE JOINT CHANGES AFTER MENISCECTOMY. *The Journal of Bone and Joint Surgery British volume* 1948;30-B(4):664-70. doi: 10.1302/0301-620X.30B4.664
6. Brandt KD, Fife RS, Braunstein EM, et al. Radiographic grading of the severity of knee osteoarthritis: Relation of the kellgren and lawrence grade to a grade based on joint space narrowing, and correlation with arthroscopic evidence of articular cartilage degeneration. *Arthritis & Rheumatism* 2010;34(11):1381-86. doi: 10.1002/art.1780341106
7. Scheller G, Sobau C, Bülow JU. Arthroscopic partial lateral meniscectomy in an otherwise normal knee: Clinical, functional, and radiographic results of a long-term follow-up study. *Arthroscopy* 2001;17(9):946-52. doi: 10.1053/jars.2001.28952
8. Hunter DJ, Guermazi A, Lo GH, et al. Evolution of semi-quantitative whole joint assessment of knee OA: MOAKS (MRI Osteoarthritis Knee Score). *Osteoarthritis Cartilage* 2011;19(8):990-1002. doi: 10.1016/j.joca.2011.05.004 [published Online First: 2011/06/08]
9. Peterfy CG, Guermazi A, Zaim S, et al. Whole-Organ Magnetic Resonance Imaging Score (WORMS) of the knee in osteoarthritis. *Osteoarthritis Cartilage* 2004;12(3):177-90. doi: 10.1016/j.joca.2003.11.003
10. Hunter DJ, Lo GH, Gale D, et al. The reliability of a new scoring system for knee osteoarthritis MRI and the validity of bone marrow lesion assessment: BLOKS (Boston Leeds Osteoarthritis Knee Score). *Ann Rheum Dis* 2008;67(2):206-11. doi: 10.1136/ard.2006.066183 [published Online First: 2007/05/03]
11. Eckstein F, Cicuttini F, Raynauld JP, et al. Magnetic resonance imaging (MRI) of articular cartilage in knee osteoarthritis (OA): morphological assessment. *Osteoarthritis Cartilage* 2006;14:46-75. doi: 10.1016/j.joca.2006.02.026
12. Blumenkrantz G, Majumdar S. Quantitative magnetic resonance imaging of articular cartilage in osteoarthritis. *Eur Cell Mater* 2007; 13. http://europepmc.org/abstract/MED/17506024

https://doi.org/10.22203/ecm.v013a08 (accessed 2007/05//).
13. Atkinson HF, Birmingham TB, Moyer RF, et al. MRI T2 and T1ρ relaxation in patients at risk for knee osteoarthritis: a systematic review and meta-analysis. *BMC Musculoskelet Disord* 2019;20(1):1-18.
14. Dunn TC, Lu Y, Jin H, et al. T2 relaxation time of cartilage at MR imaging: comparison with severity of knee osteoarthritis. *Radiology* 2004;232(2):592-8. doi: 10.1148/radiol.2322030976 [published Online First: 20040623]
15. Bashir A, Gray ML, Boutin RD, et al. Glycosaminoglycan in articular cartilage: in vivo assessment with delayed Gd(DTPA)(2-)-enhanced MR imaging. *Radiology* 1997;205(2):551-8. doi: 10.1148/radiology.205.2.9356644
16. Naredo E, Acebes C, Moller I, et al. Ultrasound validity in the measurement of knee cartilage thickness. *Ann Rheum Dis* 2009;68(8):1322-7. doi: 10.1136/ard.2008.090738 [published Online First: 2008/08/08]
17. Abraham AM, Goff I, Pearce MS, et al. Reliability and validity of ultrasound imaging of features of knee osteoarthritis in the community. *BMC Musculoskelet Disord* 2011;12(1):70. doi: 10.1186/1471-2474-12-70

18. Schmitz RJ, Wang HM, Polprasert DR, et al. Evaluation of knee cartilage thickness: A comparison between ultrasound and magnetic resonance imaging methods. *The Knee* 2017;24(2):217-23. doi: 10.1016/j.knee.2016.10.004 [published Online First: 2016/12/05]
19. Okada Y, Shinmei M, Tanaka O, et al. Localization of matrix metalloproteinase 3 (stromelysin) in osteoarthritic cartilage and synovium. *Lab Invest* 1992;66(6):680-90. [published Online First: 1992/06/01]
20. Pelletier JP, Raynauld JP, Caron J, et al. Decrease in serum level of matrix metalloproteinases is predictive of the disease-modifying effect of osteoarthritis drugs assessed by quantitative MRI in patients with knee osteoarthritis. *Ann Rheum Dis* 2010;69(12):2095-101. doi: 10.1136/ard.2009.122002 [published Online First: 2010/06/24]
21. Joseph GB, Nevitt MC, McCulloch CE, et al. Associations between molecular biomarkers and MR-based cartilage composition and knee joint morphology: data from the Osteoarthritis Initiative. *Osteoarthritis Cartilage* 2018;26(8):1070-77. doi: 10.1016/j.joca.2018.04.019 [published Online First: 2018/05/29]
22. Pengas I, Eldridge S, Assiotis A, et al. MMP-3 in the peripheral serum as a biomarker of knee osteoarthritis, 40 years after open total knee meniscectomy. *J Exp Orthop* 2018;5(1):1-8.
23. Fosang AJ, Neame PJ, Last K, et al. The interglobular domain of cartilage aggrecan is cleaved by PUMP, gelatinases, and cathepsin B. *Journal of Biological Chemistry* 1992;267(27):19470-74. doi: https://doi.org/10.1016/S0021-9258(18)41799-1
24. Zeng GQ, Chen AB, Li W, et al. High MMP-1, MMP-2, and MMP-9 protein levels in osteoarthritis. *Genetics and Molecular Research* 2015;14(4):14811-22. doi: 10.4238/2015.november.18.46
25. Billinghurst RC, Dahlberg L, Ionescu M, et al. Enhanced cleavage of type II collagen by collagenases in osteoarthritic articular cartilage. *J Clin Invest* 1997;99(7):1534-45. doi: 10.1172/JCI119316 [published Online First: 1997/04/01]
26. Neuhold LA, Killar L, Zhao W, et al. Postnatal expression in hyaline cartilage of constitutively active human collagenase-3 (MMP-13) induces osteoarthritis in mice. *J Clin Invest* 2001;107(1):35-44. doi: 10.1172/JCI10564 [published Online First: 2001/01/03]
27. Shlopov BV, Lie WR, Mainardi CL, et al. Osteoarthritic lesions: involvement of three different collagenases. *Arthritis Rheum* 1997;40(11):2065-74. doi: 10.1002/art.1780401120 [published Online First: 1997/11/19]
28. Wang M, Sampson ER, Jin H, et al. MMP13 is a critical target gene during the progression of osteoarthritis. *Arthritis Res Ther* 2013;15(1):R5. doi: 10.1186/ar4133 [published Online First: 2013/01/10]
29. Hu Q, Ecker M. Overview of MMP-13 as a promising target for the treatment of osteoarthritis. *Int J Mol Sci* 2021;22(4):1742. doi: 10.3390/ijms22041742
30. Ruan G, Xu J, Wang K, et al. Associations between knee structural measures, circulating inflammatory factors and MMP13 in patients with knee osteoarthritis. *Osteoarthritis Cartilage* 2018;26(8):1063-69. doi: 10.1016/j.joca.2018.05.003 [published Online First: 2018/05/14]
31. Tortorella M, Pratta M, Liu R-Q, et al. The Thrombospondin Motif of Aggrecanase-1 (ADAMTS-4) Is Critical for Aggrecan Substrate Recognition and Cleavage. *Journal of Biological Chemistry* 2000;275(33):25791-97. doi: 10.1074/jbc.m001065200
32. Li W, Du C, Wang H, et al. Increased serum ADAMTS-4 in knee osteoarthritis: a potential indicator for the diagnosis of osteoarthritis in early stages. *Genetics and Molecular Research* 2014;13(4):9642-49. doi: 10.4238/2014.november.14.9
33. Woolley DE, Roberts DR, Evanson JM. Inhibition of human collagenase activity by a small molecular weight serum protein. *Biochem Biophys Res Commun* 1975;66(2):747-54. doi: 10.1016/0006-291x(75)90573-2
34. Takahashi M, Naito K, Abe M, et al. *Arthritis Research & Therapy* 2004;6(3):R208. doi: 10.1186/ar1166

35. Rousseau JC, Sandell LJ, Delmas PD, et al. Development and clinical application in arthritis of a new immunoassay for serum type IIA procollagen NH2 propeptide. *Methods Mol Med* 2004;101:25-37. doi: 10.1385/1-59259-821-8:025 [published Online First: 2004/08/10]
36. Daghestani HN, Jordan JM, Renner JB, et al. Serum N-propeptide of collagen IIA (PIIANP) as a marker of radiographic osteoarthritis burden. *PLoS One* 2017;12(12):e0190251. doi: 10.1371/journal.pone.0190251
37. Luo Y, He Y, Reker D, et al. A Novel High Sensitivity Type II Collagen Blood-Based Biomarker, PRO-C2, for Assessment of Cartilage Formation. *International Journal of Molecular Sciences* 2018;19(11):3485. doi: 10.3390/ijms19113485
38. Luo Y, Samuels J, Krasnokutsky S, et al. A low cartilage formation and repair endotype predicts radiographic progression of symptomatic knee osteoarthritis. *J Orthop Traumatol* 2021;22(1) doi: 10.1186/s10195-021-00572-0
39. Christgau S, Garnero P, Fledelius C, et al. Collagen type II C-telopeptide fragments as an index of cartilage degradation. *Bone* 2001;29(3):209-15. doi: 10.1016/s8756-3282(01)00504-x [published Online First: 2001/09/15]
40. Cheng H, Hao B, Sun J, et al. C-terminal cross-linked telopeptides of type II collagen as biomarker for radiological knee osteoarthritis: a meta-analysis. *Cartilage* 2020;11(4):512-20. doi: 10.1177/1947603518798884 [published Online First: 2018/09/18]
41. Valdes AM, Meulenbelt I, Chassaing E, et al. Large scale meta-analysis of urinary C-terminal telopeptide, serum cartilage oligomeric protein and matrix metalloprotease degraded type II collagen and their role in prevalence, incidence and progression of osteoarthritis. *Osteoarthritis Cartilage* 2014;22(5):683-9. doi: 10.1016/j.joca.2014.02.007 [published Online First: 2014/03/01]
42. Dam EB, Byrjalsen I, Karsdal MA, et al. Increased urinary excretion of C-telopeptides of type II collagen (CTX-II) predicts cartilage loss over 21 months by MRI. *Osteoarthritis Cartilage* 2009;17(3):384-9. doi: 10.1016/j.joca.2008.07.009 [published Online First: 2008/09/05]
43. Sowers MF, Karvonen-Gutierrez CA, Yosef M, et al. Longitudinal changes of serum COMP and urinary CTX-II predict X-ray defined knee osteoarthritis severity and stiffness in women. *Osteoarthritis Cartilage* 2009;17(12):1609-14. doi: 10.1016/j.joca.2009.06.001 [published Online First: 2009/07/01]
44. Hao HQ, Zhang JF, He QQ, et al. Cartilage oligomeric matrix protein, C-terminal cross-linking telopeptide of type II collagen, and matrix metalloproteinase-3 as biomarkers for knee and hip osteoarthritis (OA) diagnosis: a systematic review and meta-analysis. *Osteoarthritis Cartilage* 2019;27(5):726-36. doi: 10.1016/j.joca.2018.10.009 [published Online First: 2018/11/06]
45. Nelson F, Dahlberg L, Laverty S, et al. Evidence for altered synthesis of type II collagen in patients with osteoarthritis. *J Clin Invest* 1998;102(12):2115-25. doi: 10.1172/JCI4853 [published Online First: 1998/12/17]
46. Cibere J, Zhang H, Garnero P, et al. Association of biomarkers with pre-radiographically defined and radiographically defined knee osteoarthritis in a population-based study. *Arthritis Rheum* 2009;60(5):1372-80. doi: 10.1002/art.24473 [published Online First: 2009/05/01]
47. Poole AR, Ionescu M, Fitzcharles MA, et al. The assessment of cartilage degradation in vivo: development of an immunoassay for the measurement in body fluids of type II collagen cleaved by collagenases. *J Immunol Methods* 2004;294(1-2):145-53. doi: 10.1016/j.jim.2004.09.005 [published Online First: 2005/01/11]
48. Kraus VB, Collins JE, Hargrove D, et al. Predictive validity of biochemical biomarkers in knee osteoarthritis: data from the FNIH OA Biomarkers Consortium. *Ann Rheum Dis* 2017;76(1):186-95. doi: 10.1136/annrheumdis-2016-209252 [published Online First: 2016/06/15]
49. King KB, Lindsey CT, Dunn TC, et al. A study of the relationship between molecular biomarkers of joint degeneration and the magnetic resonance-measured characteristics of cartilage in 16

symptomatic knees. *Magn Reson Imaging* 2004;22(8):1117-23. doi: 10.1016/j.mri.2004.08.001

50. Kong SY, Stabler TV, Criscione LG, et al. Diurnal variation of serum and urine biomarkers in patients with radiographic knee osteoarthritis. *Arthritis Rheum* 2006;54(8):2496-504. doi: 10.1002/art.21977 [published Online First: 2006/07/27]
51. He Y, Siebuhr AS, Brandt-Hansen NU, et al. Type X collagen levels are elevated in serum from human osteoarthritis patients and associated with biomarkers of cartilage degradation and inflammation. *BMC Musculoskelet Disord* 2014;15(1):309. doi: 10.1186/1471-2474-15-309
52. He Y, Manon-Jensen T, Arendt-Nielsen L, et al. Potential diagnostic value of a type X collagen neo-epitope biomarker for knee osteoarthritis. *Osteoarthritis Cartilage* 2019;27(4):611-20. doi: 10.1016/j.joca.2019.01.001
53. Laurent TC, Laurent UB, Fraser JRE. Serum hyaluronan as a disease marker. *Ann Med* 1996;28(3):241-53.
54. Georges C, Vigneron H, Ayral X, et al. Serum biologic markers as predictors of disease progression in osteoarthritis of the knee. *Arthritis Rheum* 1997;40(3):590-1. doi: 10.1002/art.1780400333 [published Online First: 1997/03/01]
55. Elliott AL, Kraus VB, Luta G, et al. Serum hyaluronan levels and radiographic knee and hip osteoarthritis in African Americans and Caucasians in the Johnston County Osteoarthritis Project. *Arthritis Rheum* 2005;52(1):105-11. doi: 10.1002/art.20724 [published Online First: 2005/01/11]
56. Sharif M, George E, Shepstone L, et al. Serum hyaluronic acid level as a predictor of disease progression in osteoarthritis of the knee. *Arthritis Rheum* 1995;38(6):760-7. doi: 10.1002/art.1780380608 [published Online First: 1995/06/01]
57. Rizkalla G, Reiner A, Bogoch E, et al. Studies of the articular cartilage proteoglycan aggrecan in health and osteoarthritis. Evidence for molecular heterogeneity and extensive molecular changes in disease. *J Clin Invest* 1992;90(6):2268-77. doi: 10.1172/JCI116113 [published Online First: 1992/12/01]
58. Chu CR, Sheth S, Erhart-Hledik JC, et al. Mechanically stimulated biomarkers signal cartilage changes over 5 years consistent with disease progression in medial knee osteoarthritis patients. *J Orthop Res* 2018;36(3):891-97. doi: 10.1002/jor.23720 [published Online First: 2017/09/02]
59. Mazzuca SA, Poole AR, Brandt KD, et al. Associations between joint space narrowing and molecular markers of collagen and proteoglycan turnover in patients with knee osteoarthritis. *J Rheumatol* 2006;33(6):1147-51.
60. Lohmander SL, Neame PJ, Sandy JD. The structure of aggrecan fragments in human synovial fluid. evidence that aggrecanase mediates cartilage degradation in inflammatory joint disease, joint injury, and osteoarthritis. *Arthritis Rheum* 1993;36(9):1214-22. doi: https://doi.org/10.1002/art.1780360906
61. Larsson S, Lohmander LS, Struglics A. Synovial fluid level of aggrecan ARGS fragments is a more sensitive marker of joint disease than glycosaminoglycan or aggrecan levels: a cross-sectional study. *Arthritis Res Ther* 2009;11(3):R92. doi: 10.1186/ar2735
62. Larsson S, Lohmander LS, Struglics A. An ARGS-aggrecan assay for analysis in blood and synovial fluid. *Osteoarthritis Cartilage* 2014;22(2):242-9. doi: 10.1016/j.joca.2013.12.010 [published Online First: 2013/12/24]
63. Blumenfeld O, Williams FMK, Hart DJ, et al. Association between cartilage and bone biomarkers and incidence of radiographic knee osteoarthritis (RKOA) in UK females: a prospective study. *Osteoarthritis Cartilage* 2013;21(7):923-29. doi: 10.1016/j.joca.2013.04.009
64. Larsson S, Englund M, Struglics A, et al. Association between synovial fluid levels of aggrecan ARGS fragments and radiographic progression in knee osteoarthritis. *Arthritis Res Ther* 2010;12(6):R230. doi: 10.1186/ar3217

65. Saxne T, Heinegård D. Cartilage oligomeric matrix protein: a novel marker of cartilage turnover detectable in synovial fluid and blood. *Rheumatology* 1992;31(9):583-91. doi: 10.1093/rheumatology/31.9.583
66. Bi X. Correlation of serum cartilage oligomeric matrix protein with knee osteoarthritis diagnosis: a meta-analysis. *J Orthop Surg Res* 2018;13(1):262. doi: 10.1186/s13018-018-0959-y [published Online First: 2018/10/21]
67. Vilim V, Olejarova M, Machacek S, et al. Serum levels of cartilage oligomeric matrix protein (COMP) correlate with radiographic progression of knee osteoarthritis. *Osteoarthritis Cartilage* 2002;10(9):707-13. doi: 10.1053/joca.2002.0819 [published Online First: 2002/08/31]
68. Verma P, Dalal K. Serum cartilage oligomeric matrix protein (COMP) in knee osteoarthritis: A novel diagnostic and prognostic biomarker. *J Orthop Res* 2013;31(7):999-1006. doi: 10.1002/jor.22324
69. Hoch JM, Mattacola CG, Medina McKeon JM, et al. Serum cartilage oligomeric matrix protein (sCOMP) is elevated in patients with knee osteoarthritis: a systematic review and meta-analysis. *Osteoarthritis Cartilage* 2011;19(12):1396-404. doi: 10.1016/j.joca.2011.09.005 [published Online First: 2011/10/18]
70. Clark AG, Jordan JM, Vilim V, et al. Serum cartilage oligomeric matrix protein reflects osteoarthritis presence and severity: The Johnston county osteoarthritis project. *Arthritis Rheum* 1999;42(11):2356-64. doi: 10.1002/1529-0131(199911)42:11<2356::aid-anr14>3.0.co;2-r
71. Delmas PD. Biochemical markers of bone turnover. *J Bone Miner Res* 1993;8 Suppl 2:S549-55. doi: 10.1002/jbmr.5650081323
72. Bruyere O, Collette JH, Ethgen O, et al. Biochemical markers of bone and cartilage remodeling in prediction of longterm progression of knee osteoarthritis. *J Rheumatol* 2003;30(5):1043-50.
73. Gomez B, Jr., Ardakani S, Ju J, et al. Monoclonal antibody assay for measuring bone-specific alkaline phosphatase activity in serum. *Clinical Chemistry* 1995;41(11):1560-66. doi: 10.1093/clinchem/41.11.1560
74. Park H-M, Lee J-H, Lee Y-J. Positive Association of Serum Alkaline Phosphatase Level with Severe Knee Osteoarthritis: A Nationwide Population-Based Study. *Diagnostics* 2020;10(12):1016. doi: 10.3390/diagnostics10121016
75. Orum O, Hansen M, Jensen CH, et al. Procollagen type I N-terminal propeptide (PINP) as an indicator of type I collagen metabolism: ELISA development, reference interval, and hypovitaminosis D induced hyperparathyroidism. *Bone* 1996;19(2):157-63. doi: 10.1016/8756-3282(96)00165-2
76. Kumm J, Tamm A, Lintrop M, et al. Diagnostic and prognostic value of bone biomarkers in progressive knee osteoarthritis: a 6-year follow-up study in middle-aged subjects. *Osteoarthritis and cartilage* 2013;21(6):815-22. doi: 10.1016/j.joca.2013.03.008
77. Delmas PD, Schlemmer A, Gineyts E, et al. Urinary excretion of pyridinoline crosslinks correlates with bone turnover measured on iliac crest biopsy in patients with vertebral osteoporosis. *J Bone Miner Res* 1991;6(6):639-44. doi: 10.1002/jbmr.5650060615
78. Schmidt-Rohlfing B, Thomsen M, Niedhart C, et al. Correlation of bone and cartilage markers in the synovial fluid with the degree of osteoarthritis. *Rheumatology International* 2002;21(5):193-99. doi: 10.1007/s00296-001-0170-y
79. Rosen HN, Moses AC, Garber J, et al. Serum CTX: A New Marker of Bone Resorption That Shows Treatment Effect More Often Than Other Markers Because of Low Coefficient of Variability and Large Changes with Bisphosphonate Therapy. *Calcified Tissue International* 2000;66(2):100-03. doi: 10.1007/pl00005830
80. Rosen HN, Dresner-Pollak R, Moses AC, et al. Specificity of urinary excretion of cross-linked N-telopeptides of type I collagen as a marker of bone turnover. *Calcified Tissue International* 1994;54(1):26-29. doi: 10.1007/bf00316285

81. Bettica P, Cline G, Hart DJ, et al. Evidence for increased bone resorption in patients with progressive knee osteoarthritis: Longitudinal results from the Chingford study. *Arthritis & Rheumatism* 2002;46(12):3178-84. doi: 10.1002/art.10630
