# Supplementary material for: Understanding the role of physical activity on the pathway from intra-articular knee injury to post-traumatic osteoarthritis disease in young people: a scoping review protocol
Source: BMJ Open. 2023 Mar 3;13(3):e067147. doi: 10.1136/bmjopen-2022-067147 (PMC9990625; doi:10.1136/bmjopen-2022-067147)
Supplement: Supplementary data [file bmjopen-2022-067147supp005.pdf]

Screening Tool: Full Text

Codebook

Data Dictionary Codebook

2022-12-22 21:35:11

Collapse all instruments

	#	Variable / Field Name	Field Label<br>*Field Note*	Field Attributes (Field Type, Validation, Choices, Calculations, etc.)
Instrument: **Screening Tool** (screening_tool)				Collapse
	1	record_id	Record ID	text
	2	instructions	Yes - There is explicit evidence in the title or abstract of the research including this.	descriptive
	3	maybe	Maybe - It is unclear whether there is evidence of this in the title/abstract. It may or may not be relevant but I feel this needs further investigation.	descriptive
	4	no	No - There is explicit evidence in the title or abstract of the research not including this.	descriptive
	5	reviewer_screening	Section Header: *Reviewer Details*<br>Reviewer	radio, Required<br>1 Karl Morgan<br>2 James Cowburn<br>3 Mathew Farrow<br>4 Josh Carter
	6	reviewer_date_screening	Date<br>*Just press 'Today'*	text (date_dmy), Required
	7	record_number_screening	Record number	text (number), Required
	8	review_no_meta	Section Header: *1. Study Information*<br>Is this a review, meta-analysis, book, protocol or commentary?	checkbox<br>1 review_no_meta___1 No<br>2 review_no_meta___2 Maybe<br>3 review_no_meta___3 Yes
	9	animal<br>Show the field ONLY if:<br>[review_no_meta(3)] <> 1	Is the study an animal model?	checkbox<br>1 animal___1 No<br>2 animal___2 Maybe<br>3 animal___3 Yes
	10	in_silico<br>Show the field ONLY if:<br>[review_no_meta(3)] <> 1 and [animal(3)] <> 1	Is the study purely in silico?	checkbox<br>1 in_silico___1 No<br>2 in_silico___2 Maybe<br>3 in_silico___3 Yes
	11	in_vitro<br>Show the field ONLY if:<br>[review_no_meta(3)] <> 1 and [animal(3)] <> 1 and [in_silico (3)] <> 1	Is the study purely in vitro (i.e. no human participants)?	checkbox<br>1 in_vitro___1 No<br>2 in_vitro___2 Maybe<br>3 in_vitro___3 Yes
	12	cadavers<br>Show the field ONLY if:<br>[review_no_meta(3)] <> 1 and [animal(3)] <> 1 and [in_silico (3)] <> 1 and [in_vitro(3)] <> 1	Is the study only working with cadavers?	checkbox<br>1 cadavers___1 No<br>2 cadavers___2 Maybe<br>3 cadavers___3 Yes
	13	knee_oa<br>Show the field ONLY if:<br>[review_no_meta(3)] <> 1 and [animal(3)] <> 1 and [in_silico (3)] <> 1 and [in_vitro(3)] <> 1 and [cadavers(3)] <> 1	Do the participants already have knee osteoarthritis (e.g., a cross-sectional study looking at the effects of exercise in people who already have knee OA disease) or another type of arthritis/disease?	checkbox<br>1 knee_oa___1 No<br>2 knee_oa___2 Maybe<br>3 knee_oa___3 Yes

	14	knee_injury<br>Show the field ONLY if:<br>[review_no_meta(3)] <> 1 and [animal(3)] <> 1 and [in_silico(3)] <> 1 and [in_vitro(3)] <> 1 and [cadavers(3)] <> 1 and [knee_oa(3)] <> 1	Section Header: *2. Population*<br>Could this study involve those at risk of knee post-traumatic osteoarthritis (PTOA) such as after a knee injury?<br>Cruciate ligament injuryCruciate ligament reconstructionMeniscus injuryMeniscectomy<br><br>This does NOT include:<br>Total knee replacementKnee arthroplastyosteotomy<br>*Please select one option*	checkbox, Required<br>1 | knee_injury___1 | No<br>2 | knee_injury___2 | Maybe<br>3 | knee_injury___3 | Yes
	15	age<br>Show the field ONLY if:<br>[review_no_meta(3)] <> 1 and [animal(3)] <> 1 and [in_silico(3)] <> 1 and [in_vitro(3)] <> 1 and [cadavers(3)] <> 1 and [knee_oa(3)] <> 1 and [knee_injury(1)] <> 1	Could the study participants be aged 18-40?<br>At least 50% of the population must be within 18-40 years old.<br><br>If the study is longitudinal, use the baseline age.<br>*Please select one option.*	checkbox, Required<br>1 | age___1 | No<br>2 | age___2 | Maybe<br>3 | age___3 | Yes
	16	independent_variable<br>Show the field ONLY if:<br>[review_no_meta(3)] <> 1 and [animal(3)] <> 1 and [in_silico(3)] <> 1 and [in_vitro(3)] <> 1 and [cadavers(3)] <> 1 and [knee_oa(3)] <> 1 and [knee_injury(1)] <> 1 and [age(1)] <> 1	Section Header: *3. Independent Variable*<br>Could the study have an independent variable which measures:<br>Physical activity (including Tegner)ExerciseSystemic inflammation (e.g., IL-6 concentrations)Knee joint load (moments or MSK modelling)Adipose tissueMuscle strengthMuscle sizeIntra-muscular adipose tissueBone mineral content or density<br>Or may the study have an intervention involving:<br><br>Physical activity or exerciseManipulation of joint loadWeight lossDisuse or bedrest<br>*Please select one option*	checkbox, Required<br>1 | independent_variable___1 | No<br>2 | independent_variable___2 | Maybe<br>3 | independent_variable___3 | Yes
	17	diagnosis_header<br>Show the field ONLY if:<br>[review_no_meta(3)] <> 1 and [animal(3)] <> 1 and [in_silico(3)] <> 1 and [in_vitro(3)] <> 1 and [cadavers(3)] <> 1 and [knee_oa(3)] <> 1 and [knee_injury(1)] <> 1 and [age(1)] <> 1 and [independent_variable(1)] <> 1	Section Header: *4. Outcome Variable*<br>Knee Osteoarthritis (OA) Diagnosis	descriptive
	18	disease_diagnoses<br>Show the field ONLY if:<br>[review_no_meta(3)] <> 1 and [animal(3)] <> 1 and [in_silico(3)] <> 1 and [in_vitro(3)] <> 1 and [cadavers(3)] <> 1 and [knee_oa(3)] <> 1 and [knee_injury(1)] <> 1 and [age(1)] <> 1 and [independent_variable(1)] <> 1	Could the study investigate knee OA diagnosis including the following scales:<br>Kellgren and Lawrence (K/L)Osteoarthritis Research Society International (OARSI) AtlasInternational Knee Documentation Committee (IKDC) SystemAhlbäck SystemFairbank ClassificationBrandt Grading ScaleJäger-Wirth ClassificationMerchant SystemMRI Osteoarthritis Knee Score (MOAKS)Whole Organ Magnetic Resonance Imaging Score (WORMS)Boston Leeds OA Knee Score (BLOKS)<br><br>P.s if there is another system mentioned that your are unsure of then please send me a message.<br>*Please select one option*	checkbox, Required<br>1 | disease_diagnoses___1 | No<br>2 | disease_diagnoses___2 | Maybe<br>3 | disease_diagnoses___3 | Yes
	19	imaging_indicator_header<br>Show the field ONLY if:<br>[review_no_meta(3)] <> 1 and [animal(3)] <> 1 and [in_silico(3)] <> 1 and [in_vitro(3)] <> 1 and [cadavers(3)] <> 1 and [knee_oa(3)] <> 1 and [knee_injury(1)] <> 1 and [age(1)] <> 1 and [independent_variable(1)] <> 1	Medical Imaging Indicator	descriptive
	20	medical_imaging_indicators<br>Show the field ONLY if:<br>[review_no_meta(3)] <> 1 and [animal(3)] <> 1 and [in_silico(3)] <> 1 and [in_vitro(3)] <> 1 and [cadavers(3)] <> 1 and [knee_oa(3)] <> 1 and [knee_injury(1)] <> 1 and [age(1)] <> 1 and [independent_variable(1)] <> 1	Could the study investigate medical imaging indicators of tissue health including the following techniques:<br>MRI quantified cartilage thickness and volumeT1rho mappingT2 mappingDelayed contrast-enhanced MRI of cartilage (dGEMRIC)Ultrasound quantified cartilage thickness<br>*Please select one option*	checkbox, Required<br>1 | medical_imaging_indicators___1 | No<br>2 | medical_imaging_indicators___2 | Maybe<br>3 | medical_imaging_indicators___3 | Yes

	21	biomarkers_header<br>Show the field ONLY if:<br>[review_no_meta(3)] <> 1 and [animal(3)] <> 1 and [in_silico(3)] <> 1 and [in_vitro(3)] <> 1 and [cadavers(3)] <> 1 and [knee_oa(3)] <> 1 and [knee_injury(1)] <> 1 and [age(1)] <> 1 and [independent_variable(1)] <> 1	Biomarkers	descriptive
	22	biomarkers<br>Show the field ONLY if:<br>[review_no_meta(3)] <> 1 and [animal(3)] <> 1 and [in_silico(3)] <> 1 and [in_vitro(3)] <> 1 and [cadavers(3)] <> 1 and [knee_oa(3)] <> 1 and [knee_injury(1)] <> 1 and [age(1)] <> 1 and [independent_variable(1)] <> 1	Could the study investigate biomarkers including the following:<br>Cartilage synthesisC-propeptide of type II collagen (CP-II)Chondroitin sulphate 846 epitope (CS846)N-Propeptide of Collagen IIA (PIIANP)N-Propeptide of Collagen IIB (PIIBNP)<br>Cartilage degradationMatrix metalloproteases (e.g., MMP-3, MMP-9, MMP-13)A disintegrin and metalloproteinase with thrombospondin motifs (e.g., ADAMTS-4, ADAMTS-5)Collagen type II cleavage product (C2C)Col2-3/4 C-terminal cleavage product of types I and II collagen (C1,2C)Neoepitope of collagen X (C10C or Col10Neo)Hyaluronic acid (HA) Aggrecan or aggrecan fragments (ARGS)Keratin sulphate (KS)Cartilage oligomeric matrix protein (COMP)<br>Bone formationOsteocalcin (OC)Bone alkaline phosphatase (BAP)Procollagen type I N-terminal propeptide (PINP)Procollagen type I C-terminal propeptide (PICP)<br>Bone resorptionPyridinoline (PYD)Deoxypyridinoline (DPD)Tartrate-resistant acid phosphatase 5b (TRAP-5)N-telopeptide if type I collagen (NTX-I)C-telopeptide of type I collagen (CTX-I)Insulin-like growth factor 1 (IGF-1)Transforming growth factor beta (TGFB)<br>*Please select one option*	checkbox, Required<br>1 | biomarkers___1 | No<br>2 | biomarkers___2 | Maybe<br>3 | biomarkers___3 | Yes
	23	all_yes_dec<br>Show the field ONLY if:<br>[knee_injury(3)]= 1 and [age(3)] = 1 and [independent_variable(3)] = 1 and [review_no_meta(3)] <> 1 and [animal(3)] <> 1 and [in_silico(3)] <> 1 and [cadavers(3)] <> 1 or [disease_diagnoses(3)] = 1 or [medical_imaging_indicators(3)] = 1 or [biomarkers(3)] = 1	Section Header: *Decision*<br>Select 'Yes' in Covidence	descriptive
	24	all_maybe_dec<br>Show the field ONLY if:<br>[knee_injury(2)]= 1 and [age(2)] = 1 and [independent_variable(2)] = 1 and [review_no_meta(1)] <> 1 and [animal(1)] <> 1 and [in_silico(1)] <> 1 and [cadavers(1)] <> 1	Select 'Maybe' in Covidence	descriptive
	25	knee_inj_maybe<br>Show the field ONLY if:<br>[knee_injury(2)]= 1 and [age(1)] = 1 and [independent_variable(1)] = 1 and [disease(1)] = 1	Select 'Maybe' in Covidence	descriptive
	26	age_maybe<br>Show the field ONLY if:<br>[knee_injury(1)]= 1 and [age(2)] = 1 and [independent_variable(1)] = 1 and [disease(1)] = 1 and [review_no_meta(1)] <> 1 and [animal(1)] <> 1 and [in_silico(1)] <> 1 and [cadavers(1)] <> 1	Select 'Maybe' in Covidence	descriptive

	27	iv_maybe<br>Show the field ONLY if: [knee_injury(1)]= 1 and [age(1)] = 1 and [independent_variable(2)] = 1 and [disease(1)] = 1 and [review_no_meta(1)] <> 1 and [animal(1)] <> 1 and [in_silico(1)] <> 1 and [cadavers(1)] <> 1	Select 'Maybe' in Covidence	descriptive
	28	ov_maybe<br>Show the field ONLY if: [knee_injury(1)]= 1 and [age(1)] = 1 and [independent_variable(1)] = 1 and [disease(2)] = 1 and [review_no_meta(1)] <> 1 and [animal(1)] <> 1 and [in_silico(1)] <> 1 and [cadavers(1)] <> 1	Select 'Maybe' in Covidence	descriptive
	29	age_iv_ov_maybe<br>Show the field ONLY if: [knee_injury(1)]= 1 and [age(2)] = 1 and [independent_variable(2)] = 1 and [disease(2)] = 1 and [review_no_meta(1)] <> 1 and [animal(1)] <> 1 and [in_silico(1)] <> 1 and [cadavers(1)] <> 1	Select 'Maybe' in Covidence	descriptive
	30	knee_inj_iv_ov_maybe<br>Show the field ONLY if: [knee_injury(2)]= 1 and [age(1)] = 1 and [independent_variable(2)] = 1 and [disease(2)] = 1 and [review_no_meta(1)] <> 1 and [animal(1)] <> 1 and [in_silico(1)] <> 1 and [cadavers(1)] <> 1	Select 'Maybe' in Covidence	descriptive
	31	knee_inj_age_ov_maybe<br>Show the field ONLY if: [knee_injury(2)]= 1 and [age(2)] = 1 and [independent_variable(1)] = 1 and [disease(2)] = 1 and [review_no_meta(1)] <> 1 and [animal(1)] <> 1 and [in_silico(1)] <> 1 and [cadavers(1)] <> 1	Select 'Maybe' in Covidence	descriptive
	32	knee_inj_age_iv_maybe<br>Show the field ONLY if: [knee_injury(2)]= 1 and [age(2)] = 1 and [independent_variable(2)] = 1 and [review_no_meta(1)] <> 1 and [animal(1)] <> 1 and [in_silico(1)] <> 1 and [cadavers(1)] <> 1	Select 'Maybe' in Covidence	descriptive
	33	knee_inj_age_iv_maybe_2<br>Show the field ONLY if: [disease_diagnoses(2)] = 1 AND [medical_imaging_indicators(2)] = 1 AND [biomarkers(2)] = 1	Select 'Maybe' in Covidence	descriptive
	34	select_no<br>Show the field ONLY if: [review_no_meta(3)] = 1 or [animal(3)] = 1 or [in_silico(3)] = 1 or [in_vitro(3)] = 1 or [cadavers(3)] = 1 or [knee_oa(3)] =1 or [knee_injury(1)] = 1 or [age(1)] = 1 or [independent_variable(1)] = 1	Select 'No' in Covidence	descriptive

	35	select_no_2<br><br>Show the field ONLY if: [disease_diagnoses(1)] = 1 AND [medical_imaging_indicators(1)] = 1 AND [biomarkers(1)] = 1	Select 'No' in Covidence	descriptive
	36	screening_tool_complete	Section Header: *Form Status*<br><br>Complete?	dropdown<br>0 Incomplete<br>1 Unverified<br>2 Complete
