# Supplementary figures and images for: Understanding the role of physical activity on the pathway from intra-articular knee injury to post-traumatic osteoarthritis disease in young people: a scoping review protocol
Source: BMJ Open. 2023 Mar 3;13(3):e067147. doi: 10.1136/bmjopen-2022-067147 (PMC9990625; doi:10.1136/bmjopen-2022-067147)

Supplementary Material 5. Protocol for Contacting Authors

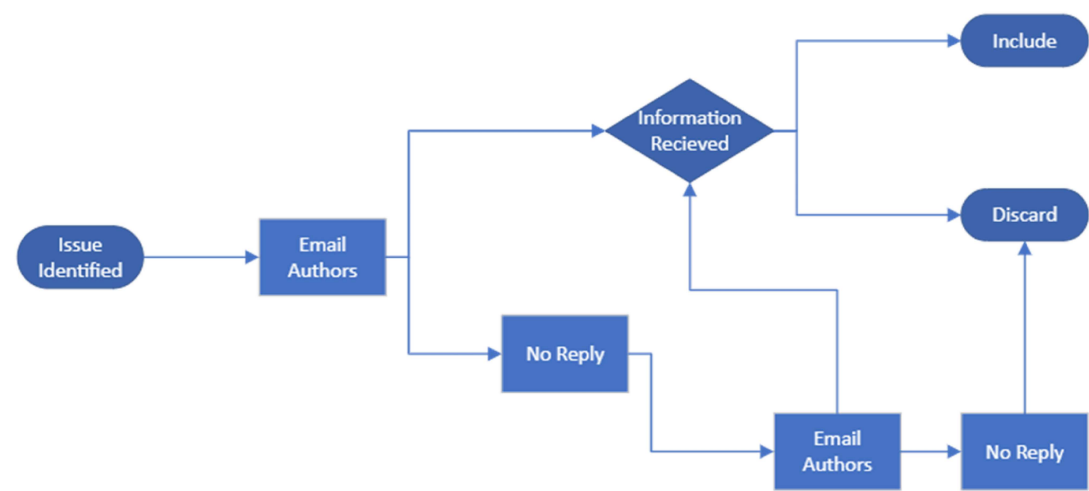

Supplement: Supplementary data [file bmjopen-2022-067147supp006.pdf]
