# Supplementary material for: Understanding the role of physical activity on the pathway from intra-articular knee injury to post-traumatic osteoarthritis disease in young people: a scoping review protocol
Source: BMJ Open. 2023 Mar 3;13(3):e067147. doi: 10.1136/bmjopen-2022-067147 (PMC9990625; doi:10.1136/bmjopen-2022-067147)
Supplement: Supplementary data [file bmjopen-2022-067147supp007.pdf]

Supplementary Material 6. Johanna Brigg's Institute (JBI) Appraisal Criteria

REDCap Flow Diagram for Critical Appraisal

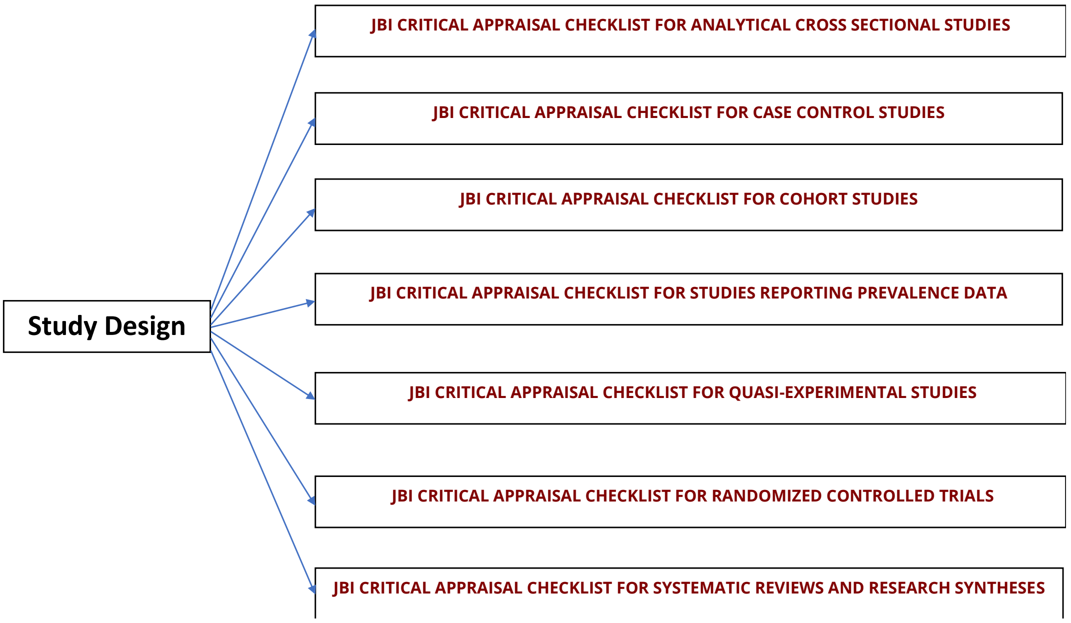

JBI Quality Appraisal

Codebook

Data Dictionary Codebook

2022-08-02 19:46:29

Collapse all instruments

	#	Variable / Field Name	Field Label<br>*Field Note*	Field Attributes (Field Type, Validation, Choices, Calculations, etc.)
Instrument: **Study Design** (study_design) Collapse				
	1	record_id	Record ID	text
	2	study_design	Please select the study design	radio<br>1 Analytical Cross-Sectional Study<br>2 Case Control Study<br>3 Cohort Study<br>4 Prevalence Study<br>5 Quasi-Experimental Study<br>6 Randomised Control Trial<br>7 Systematic Review and Research Synthesis<br>8 Unclear
	3	study_design_complete	Section Header: *Form Status*<br>Complete?	dropdown<br>0 Incomplete<br>1 Unverified<br>2 Complete
Instrument: **Study Design Unclear** (study_design_unclear) Collapse				
	4	reviewer_name_unclear	Section Header: *Reviewer Details*<br>Reviewer	dropdown<br>1 Karl Morgan<br>2 James Cowburn<br>3 Matthew Farrow
	5	reviewer_date_unclear	Date reviewed<br>*Just press 'Today'*	text (date_dmy)
	6	authors_unclear	Section Header: *Study Information*<br>Authors	text
	7	year_unclear	Publication year	text
	8	record_number_unclear	Record number	text
	9	study_design_unclear_complete	Section Header: *Form Status*<br>Complete?	dropdown<br>0 Incomplete<br>1 Unverified<br>2 Complete
Instrument: **JBI Critical Appraisal Checklist for Analytical Cross-Sectional Studies** (jbi_critical_appraisal_checklist_for_analytical_cr) Collapse				
	10	reviewer_name_cross	Section Header: *Reviewer Details*<br>Reviewer	dropdown<br>1 Karl Morgan<br>2 James Cowburn<br>3 Matthew Farrow
	11	reviewer_date_cross	Date reviewed<br>*Just press 'Today'*	text (date_dmy)
	12	authors_cross	Section Header: *Study Information*<br>Authors	text
	13	year_cross	Publication year	text
	14	record_number_cross	Record number	text

	15	cross_section_01	Section Header: *JBI Critical Appraisal Checklist for Analytical Cross-Sectional Studies*<br>1. Were the criteria for inclusion in the sample clearly defined?	radio (Matrix)<br>1 Yes<br>2 No<br>3 Unclear<br>4 Not Applicable
	16	cross_section_02	2. Were the study subjects and the setting described in detail?	radio (Matrix)<br>1 Yes<br>2 No<br>3 Unclear<br>4 Not Applicable
	17	cross_section_03	3. Was the exposure measured in a valid and reliable way?	radio (Matrix)<br>1 Yes<br>2 No<br>3 Unclear<br>4 Not Applicable
	18	cross_section_04	4. Were objective, standard criteria used for measurement of the condition?	radio (Matrix)<br>1 Yes<br>2 No<br>3 Unclear<br>4 Not Applicable
	19	cross_section_05	5. Were confounding factors identified?	radio (Matrix)<br>1 Yes<br>2 No<br>3 Unclear<br>4 Not Applicable
	20	cross_section_06	6. Were strategies to deal with confounding factors stated?	radio (Matrix)<br>1 Yes<br>2 No<br>3 Unclear<br>4 Not Applicable
	21	cross_section_07	7. Were the outcomes measured in a valid and reliable way?	radio (Matrix)<br>1 Yes<br>2 No<br>3 Unclear<br>4 Not Applicable
	22	cross_section_08	8. Was appropriate statistical analysis used?	radio (Matrix)<br>1 Yes<br>2 No<br>3 Unclear<br>4 Not Applicable
	23	jbi_critical_appraisal_checklist_for_analytical_cr_complete	Section Header: *Form Status*<br>Complete?	dropdown<br>0 Incomplete<br>1 Unverified<br>2 Complete
Instrument: **JBI Critical Appraisal Checklist for Case Control Studies** (jbi_critical_appraisal_checklist_for_case_control) Collapse				
	24	reviewer_name_case	Section Header: *Reviewer Details*<br>Reviewer	dropdown<br>1 Karl Morgan<br>2 James Cowburn<br>3 Matthew Farrow
	25	reviewer_date_case	Date reviewed<br>*Just press 'Today'*	text (date_dmy)
	26	authors_case	Section Header: *Study Information*<br>Authors	text

	27	year_case	Publication year	text
	28	record_number_case	Record number	text
	29	case_control_01	Section Header: *JBI Critical Appraisal Checklist for Case Control Studies*<br>1. Were the groups comparable other than the presence of disease in cases or the absence of disease in controls?	radio (Matrix)<br>1 Yes<br>2 No<br>3 Unclear<br>4 Not Applicable
	30	case_control_02	2. Were cases and controls matched appropriately?	radio (Matrix)<br>1 Yes<br>2 No<br>3 Unclear<br>4 Not Applicable
	31	case_control_03	3. Were the same criteria used for identification of cases and controls?	radio (Matrix)<br>1 Yes<br>2 No<br>3 Unclear<br>4 Not Applicable
	32	case_control_04	4. Was exposure measured in a standard, valid and reliable way?	radio (Matrix)<br>1 Yes<br>2 No<br>3 Unclear<br>4 Not Applicable
	33	case_control_05	5. Was exposure measured in the same way for cases and controls?	radio (Matrix)<br>1 Yes<br>2 No<br>3 Unclear<br>4 Not Applicable
	34	case_control_06	6. Were confounding factors identified?	radio (Matrix)<br>1 Yes<br>2 No<br>3 Unclear<br>4 Not Applicable
	35	case_control_07	7. Were strategies to deal with confounding factors stated?	radio (Matrix)<br>1 Yes<br>2 No<br>3 Unclear<br>4 Not Applicable
	36	case_control_08	8. Were outcomes assessed in a standard, valid and reliable way for cases and controls?	radio (Matrix)<br>1 Yes<br>2 No<br>3 Unclear<br>4 Not Applicable
	37	case_control_09	9. Was the exposure period of interest long enough to be meaningful?	radio (Matrix)<br>1 Yes<br>2 No<br>3 Unclear<br>4 Not Applicable
	38	case_control_10	10. Was appropriate statistical analysis used?	radio (Matrix)<br>1 Yes<br>2 No<br>3 Unclear<br>4 Not Applicable

	39	jbi_critical_appraisal_checklist_for_case_control_complete	Section Header: *Form Status*<br>Complete?	dropdown<br>0 Incomplete<br>1 Unverified<br>2 Complete
Instrument: **JBI Critical Appraisal Checklist for Cohort Studies** (jbi_critical_appraisal_checklist_for_cohort_studie) Collapse				
	40	reviewer_name_cohort	Section Header: *Reviewer Details*<br>Reviewer	dropdown<br>1 Karl Morgan<br>2 James Cowburn<br>3 Matthew Farrow
	41	reviewer_date_cohort	Date reviewed<br>*Just press 'Today'*	text (date_dmy)
	42	authors_cohort	Section Header: *Study Information*<br>Authors<br>*Surname*	text
	43	year_cohort	Publication year<br>*e.g., 2010*	text
	44	record_number_cohort	Record number	text
	45	cohort_01	Section Header: *JBI Critical Appraisal Checklist for Cohort Studies*<br>1. Were the two groups similar and recruited from the same population?	radio (Matrix)<br>1 Yes<br>2 No<br>3 Unclear<br>4 Not Applicable
	46	cohort_02	2. Were the exposures measured similarly to assign people to both exposed and unexposed groups?	radio (Matrix)<br>1 Yes<br>2 No<br>3 Unclear<br>4 Not Applicable
	47	cohort_03	3. Was the exposure measured in a valid and reliable way?	radio (Matrix)<br>1 Yes<br>2 No<br>3 Unclear<br>4 Not Applicable
	48	cohort_04	4. Were confounding factors identified?	radio (Matrix)<br>1 Yes<br>2 No<br>3 Unclear<br>4 Not Applicable
	49	cohort_05	5. Were strategies to deal with confounding factors stated?	radio (Matrix)<br>1 Yes<br>2 No<br>3 Unclear<br>4 Not Applicable
	50	cohort_06	6. Were the groups/participants free of the outcome at the start of the study (or at the moment of exposure)?	radio (Matrix)<br>1 Yes<br>2 No<br>3 Unclear<br>4 Not Applicable
	51	cohort_07	7. Were the outcomes measured in a valid and reliable way?	radio (Matrix)<br>1 Yes<br>2 No<br>3 Unclear<br>4 Not Applicable

	52	cohort_08	8. Was the follow up time reported and sufficient to be long enough for outcomes to occur?	radio (Matrix)<br>1 Yes<br>2 No<br>3 Unclear<br>4 Not Applicable
	53	cohort_09	9. Was follow up complete, and if not, were the reasons to loss to follow up described and explored?	radio (Matrix)<br>1 Yes<br>2 No<br>3 Unclear<br>4 Not Applicable
	54	cohort_10	10. Were strategies to address incomplete follow up utilized?	radio (Matrix)<br>1 Yes<br>2 No<br>3 Unclear<br>4 Not Applicable
	55	cohort_11	11. Was appropriate statistical analysis used?	radio (Matrix)<br>1 Yes<br>2 No<br>3 Unclear<br>4 Not Applicable
	56	jbi_critical_appraisal_checklist_for_cohort_studie_complete	Section Header: *Form Status*<br>Complete?	dropdown<br>0 Incomplete<br>1 Unverified<br>2 Complete
Instrument: **JBI Critical Appraisal Checklist for Studies Reporting Prevalence Data** (jbi_critical_appraisal_checklist_for_studies_repor) Collapse				
	57	reviewer_name_prevalence	Section Header: *Reviewer Details*<br>Reviewer	dropdown<br>1 Karl Morgan<br>2 James Cowburn<br>3 Matthew Farrow
	58	reviewer_date_prevalence	Date reviewed<br>*Just press 'Today'*	text (date_dmy)
	59	authors_prevalence	Section Header: *Study Information*<br>Authors	text
	60	year_prevalence	Publication year	text
	61	record_number_prevalence	Record number	text
	62	prevalence_01	Section Header: *JBI Critical Appraisal Checklist for Studies Reporting Prevalence Data*<br>1. Was the sample frame appropriate to address the target population?	radio (Matrix)<br>1 Yes<br>2 No<br>3 Unclear<br>4 Not Applicable
	63	prevalence_02	2. Were study participants sampled in an appropriate way?	radio (Matrix)<br>1 Yes<br>2 No<br>3 Unclear<br>4 Not Applicable
	64	prevalence_03	3. Was the sample size adequate?	radio (Matrix)<br>1 Yes<br>2 No<br>3 Unclear<br>4 Not Applicable

	65	prevalence_04	4. Were the study subjects and the setting described in detail?	radio (Matrix)<br>1 Yes<br>2 No<br>3 Unclear<br>4 Not Applicable
	66	prevalence_05	5. Was the data analysis conducted with sufficient coverage of the identified sample?	radio (Matrix)<br>1 Yes<br>2 No<br>3 Unclear<br>4 Not Applicable
	67	prevalence_06	6. Were valid methods used for the identification of the condition?	radio (Matrix)<br>1 Yes<br>2 No<br>3 Unclear<br>4 Not Applicable
	68	prevalence_07	7. Was the condition measured in a standard, reliable way for all participants?	radio (Matrix)<br>1 Yes<br>2 No<br>3 Unclear<br>4 Not Applicable
	69	prevalence_08	8. Was there appropriate statistical analysis?	radio (Matrix)<br>1 Yes<br>2 No<br>3 Unclear<br>4 Not Applicable
	70	prevalence_09	9. Was the response rate adequate, and if not, was the low response rate managed appropriately?	radio (Matrix)<br>1 Yes<br>2 No<br>3 Unclear<br>4 Not Applicable
	71	jbi_critical_appraisal_checklist_for_studies_repor_complete	Section Header: *Form Status*<br>Complete?	dropdown<br>0 Incomplete<br>1 Unverified<br>2 Complete
Instrument: **JBI Critical Appraisal Checklist for Quasi-Experimental Studies** (jbi_critical_appraisal_checklist_for_quasiexperime) Collapse				
	72	reviewer_name_quasi	Section Header: *Reviewer Details*<br>Reviewer	dropdown<br>1 Karl Morgan<br>2 James Cowburn<br>3 Matthew Farrow
	73	reviewer_date_quasi	Date reviewed<br>*Just press 'Today'*	text (date_dmy)
	74	author_quasi	Section Header: *Study Information*<br>Authors	text
	75	year_quasi	Publication year	text
	76	record_number_quasi	Record number	text
	77	quasi_experimental_01	Section Header: *JBI Critical Appraisal Checklist for Quasi-Experimental Studies*<br>1. Is it clear in the study what is the 'cause' and what is the 'effect' (i.e. there is no confusion about which variable comes first)?	radio (Matrix)<br>1 Yes<br>2 No<br>3 Unclear<br>4 Not Applicable

	78	quasi_experimental_02	2. Were the participants included in any comparisons similar?	radio (Matrix)<br>1 Yes<br>2 No<br>3 Unclear<br>4 Not Applicable
	79	quasi_experimental_03	3. Were the participants included in any comparisons receiving similar treatment/care, other than the exposure or intervention of interest?	radio (Matrix)<br>1 Yes<br>2 No<br>3 Unclear<br>4 Not Applicable
	80	quasi_experimental_04	4. Was there a control group?	radio (Matrix)<br>1 Yes<br>2 No<br>3 Unclear<br>4 Not Applicable
	81	quasi_experimental_05	5. Were there multiple measurements of the outcome both pre and post the intervention/exposure?	radio (Matrix)<br>1 Yes<br>2 No<br>3 Unclear<br>4 Not Applicable
	82	quasi_experimental_06	6. Was follow up complete and if not, were differences between groups in terms of their follow up adequately described and analyzed?	radio (Matrix)<br>1 Yes<br>2 No<br>3 Unclear<br>4 Not Applicable
	83	quasi_experimental_07	7. Were the outcomes of participants included in any comparisons measured in the same way?	radio (Matrix)<br>1 Yes<br>2 No<br>3 Unclear<br>4 Not Applicable
	84	quasi_experimental_08	8. Were outcomes measured in a reliable way?	radio (Matrix)<br>1 Yes<br>2 No<br>3 Unclear<br>4 Not Applicable
	85	quasi_experimental_09	9. Was appropriate statistical analysis used?	radio (Matrix)<br>1 Yes<br>2 No<br>3 Unclear<br>4 Not Applicable
	86	jbi_critical_appraisal_checklist_for_quasiexperime_complete	Section Header: *Form Status*<br>Complete?	dropdown<br>0 Incomplete<br>1 Unverified<br>2 Complete
Instrument: **JBI Critical Appraisal Checklist for Randomised Controlled Trials** (jbi_critical_appraisal_checklist_for_randomised_co) Collapse				
	87	reviewer_name_rct	Section Header: *Reviewer Details*<br>Reviewer	dropdown<br>1 Karl Morgan<br>2 James Cowburn<br>3 Matthew Farrow
	88	reviewer_date_rct	Date reviewed<br>*Just press 'Today'*	text (date_dmy)

	89	authors_rct	Section Header: *Study Information*<br>Authors	text
	90	year_rct	Publication year	text
	91	record_number_rct	Record number	text
	92	rct_01	Section Header: *JBI Critical Appraisal Checklist for Randomised Controlled Trials*<br>1. Was true randomization used for assignment of participants to treatment groups?	radio (Matrix)<br>1 Yes<br>2 No<br>3 Unclear<br>4 Not Applicable
	93	rct_02	2. Was allocation to treatment groups concealed?	radio (Matrix)<br>1 Yes<br>2 No<br>3 Unclear<br>4 Not Applicable
	94	rct_03	3. Were treatment groups similar at the baseline?	radio (Matrix)<br>1 Yes<br>2 No<br>3 Unclear<br>4 Not Applicable
	95	rct_04	4. Were participants blind to treatment assignment?	radio (Matrix)<br>1 Yes<br>2 No<br>3 Unclear<br>4 Not Applicable
	96	rct_05	5. Were those delivering treatment blind to treatment assignment?	radio (Matrix)<br>1 Yes<br>2 No<br>3 Unclear<br>4 Not Applicable
	97	rct_06	6. Were outcomes assessors blind to treatment assignment?	radio (Matrix)<br>1 Yes<br>2 No<br>3 Unclear<br>4 Not Applicable
	98	rct_07	7. Were treatment groups treated identically other than the intervention of interest?	radio (Matrix)<br>1 Yes<br>2 No<br>3 Unclear<br>4 Not Applicable
	99	rct_08	8. Was follow up complete and if not, were differences between groups in terms of their follow up adequately described and analyzed?	radio (Matrix)<br>1 Yes<br>2 No<br>3 Unclear<br>4 Not Applicable
	100	rct_09	9. Were participants analyzed in the groups to which they were randomized?	radio (Matrix)<br>1 Yes<br>2 No<br>3 Unclear<br>4 Not Applicable

	101	rct_10	10. Were outcomes measured in the same way for treatment groups?	radio (Matrix)<br>1 Yes<br>2 No<br>3 Unclear<br>4 Not Applicable
	102	rct_11	11. Were outcomes measured in a reliable way?	radio (Matrix)<br>1 Yes<br>2 No<br>3 Unclear<br>4 Not Applicable
	103	rct_12	12. Was appropriate statistical analysis used?	radio (Matrix)<br>1 Yes<br>2 No<br>3 Unclear<br>4 Not Applicable
	104	rct_13	13. Was the trial design appropriate, and any deviations from the standard RCT design (individual randomization, parallel groups) accounted for in the conduct and analysis of the trial?	radio (Matrix)<br>1 Yes<br>2 No<br>3 Unclear<br>4 Not Applicable
	105	jbi_critical_appraisal_checklist_for_randomised_co_complete	Section Header: *Form Status*<br>Complete?	dropdown<br>0 Incomplete<br>1 Unverified<br>2 Complete
Instrument: **JBI Critical Appraisal Checklist for Systematic Reviews and Research Syntheses** (jbi_critical_appraisal_checklist_for_systematic_re) Collapse				
	106	reviewer_name_review	Section Header: *Study Information*<br>Reviewer	dropdown<br>1 Karl Morgan<br>2 James Cowburn<br>3 Matthew Farrow
	107	reviewer_date_review	Date reviewed<br>*Just press 'Today'*	text (date_dmy)
	108	authors_review	Section Header: *Study Information*<br>Authors	text
	109	year_review	Publication year	text
	110	record_number_review	Record number	text
	111	reviews_01	Section Header: *JBI Critical Appraisal Checklist for Systematic Reviews and Research Syntheses*<br>1. Is the review question clearly and explicitly stated?	radio (Matrix)<br>1 Yes<br>2 No<br>3 Unclear<br>4 Not Applicable
	112	reviews_02	2. Were the inclusion criteria appropriate for the review question?	radio (Matrix)<br>1 Yes<br>2 No<br>3 Unclear<br>4 Not Applicable
	113	reviews_03	3. Was the search strategy appropriate?	radio (Matrix)<br>1 Yes<br>2 No<br>3 Unclear<br>4 Not Applicable

	114	reviews_04	4. Were the sources and resources used to search for studies adequate?	radio (Matrix)<br>1 Yes<br>2 No<br>3 Unclear<br>4 Not Applicable
	115	reviews_05	5. Were the criteria for appraising studies appropriate?	radio (Matrix)<br>1 Yes<br>2 No<br>3 Unclear<br>4 Not Applicable
	116	reviews_06	6. Was critical appraisal conducted by two or more reviewers independently?	radio (Matrix)<br>1 Yes<br>2 No<br>3 Unclear<br>4 Not Applicable
	117	reviews_07	7. Were there methods to minimize errors in data extraction?	radio (Matrix)<br>1 Yes<br>2 No<br>3 Unclear<br>4 Not Applicable
	118	reviews_08	8. Were the methods used to combine studies appropriate?	radio (Matrix)<br>1 Yes<br>2 No<br>3 Unclear<br>4 Not Applicable
	119	reviews_09	9. Was the likelihood of publication bias assessed?	radio (Matrix)<br>1 Yes<br>2 No<br>3 Unclear<br>4 Not Applicable
	120	reviews_10	10. Were recommendations for policy and/or practice supported by the reported data?	radio (Matrix)<br>1 Yes<br>2 No<br>3 Unclear<br>4 Not Applicable
	121	reviews_11	11. Were the specific directives for new research appropriate?	radio (Matrix)<br>1 Yes<br>2 No<br>3 Unclear<br>4 Not Applicable
	122	jbi_critical_appraisal_checklist_for_systematic_re_complete	Section Header: *Form Status*<br>Complete?	dropdown<br>0 Incomplete<br>1 Unverified<br>2 Complete
