# Supplementary material for: Understanding the role of physical activity on the pathway from intra-articular knee injury to post-traumatic osteoarthritis disease in young people: a scoping review protocol
Source: BMJ Open. 2023 Mar 3;13(3):e067147. doi: 10.1136/bmjopen-2022-067147 (PMC9990625; doi:10.1136/bmjopen-2022-067147)
Supplement: Supplementary data [file bmjopen-2022-067147supp008.pdf]

## Data Charting Draft

Codebook

## Data Dictionary Codebook

2022-12-22 21:44:12

Collapse all instruments

| | # | Variable / Field Name | Field Label<br>*Field Note* | Field Attributes (Field Type, Validation, Choices, Calculations, etc.) |
|---|---|---|---|---|
| Instrument: **Data Extraction 2** (data_extraction_2) Collapse | | | | |
| | 1 | record_id | Record ID | text |
| | 2 | reviewer_name_extraction_v2 | Reviewer | dropdown, Required<br>1 Karl Morgan<br>2 James Cowburn<br>3 Mathew Farrow<br>4 Josh Carter |
| | 3 | reviewer_date_extraction_v2 | Date of extraction<br>*Just press 'Today'* | text (date_dmy), Required |
| | 4 | record_number_extraction_v2 | Section Header: *Study Information*<br>Record number | text, Required |
| | 5 | first_author_v2 | First author<br>*Format as: second name, first name initial (e.g., Morgan, K.).* | text, Required |
| | 6 | co_authors_v2 | Co-authors<br>*Format as: Initial followed by surname (e.g., Morgan, K.). Write n/a if none.* | notes, Required |
| | 7 | publication_year_v2 | Publication year<br>*Please insert as number (e.g., 2006).* | text (number), Required |
| | 8 | country_research_origin_v2 | Country of research origin<br><br>ISO Alpha 3 codes<br>*Please use ISO Alpha 3 codes (e.g., USA, GBR, FRA, DNK, CAN). Write unclear if unknown.* | text, Required |
| | 9 | study_aim_1_v2 | Section Header: *Study aims*<br>Study aim 1<br>*Write unclear if unknown.* | notes, Required |
| | 10 | study_aim_2_v2 | Study aim 2<br>*Write unclear if unknown. If no secondary aim write n/a.* | notes, Required |
| | 11 | study_aim_3_v2 | Study aim 3<br>*Write unclear if unknown. If no tertiary aim write n/a.* | notes, Required |
| | 12 | study_design_v2 | Section Header: *Methodology - Study Design*<br>Study design | radio, Required<br>1 Analytical cross-sectional<br>2 Case Control<br>3 Cohort<br>4 Prevalence data<br>5 Quasi-experimental<br>6 Randomised control trial<br>7 Unclear |
| | 13 | number_of_participants_v2 | Section Header: *Methodology - Experimental Population Size*<br>Total number of participants<br>*Report as: number* | text (number), Required |
| | 14 | number_of_males_v2 | Number of males<br>*Report as: number* | text, Required |
| | 15 | number_of_females_v2 | Number of females<br>*Report as: number* | text, Required |
| | 16 | age_heading_v2 | Section Header: *Methodology - Experimental Group Demographic Information*<br>Experimental Group Age | descriptive |
| | 17 | total_population_age_v2 | Total Population | descriptive |

| | | | | |
|---|---|---|---|---|
| | 18 | participant_age_total_v2 | How was the age of the total study population reported?<br>*Select all relevant answers* | checkbox, Required<br>1 participant_age_total_v2___1 Mean<br>2 participant_age_total_v2___2 Standard deviation<br>3 participant_age_total_v2___3 Median<br>4 participant_age_total_v2___4 Range |
| | 19 | age_mean_v2<br>Show the field ONLY if: [participant_age_total_v2(1)] = '1' | Mean<br>*Years* | text, Required |
| | 20 | age_stdev_v2<br>Show the field ONLY if: [participant_age_total_v2(2)] = '1' | Standard deviation<br>*Years* | text, Required |
| | 21 | age_median_v2<br>Show the field ONLY if: [participant_age_total_v2(3)] = '1' | Median<br>*Years* | text, Required |
| | 22 | age_range_v2<br>Show the field ONLY if: [participant_age_total_v2(4)] = '1' | Range<br>*Years* | text, Required |
| | 23 | male_population_age_v2 | Male Population | descriptive |
| | 24 | participant_age_male_v2 | How was the age of the male study population reported?<br>*Select all relevant answers* | checkbox, Required<br>1 participant_age_male_v2___1 Mean<br>2 participant_age_male_v2___2 Standard deviation<br>3 participant_age_male_v2___3 Median<br>4 participant_age_male_v2___4 Range |
| | 25 | age_mean_male_v2<br>Show the field ONLY if: [participant_age_male_v2(1)] = '1' | Mean<br>*Years* | text, Required |
| | 26 | age_stdev_male_v2<br>Show the field ONLY if: [participant_age_male_v2(2)] = '1' | Standard deviation<br>*Years* | text, Required |
| | 27 | age_median_male_v2<br>Show the field ONLY if: [participant_age_male_v2(3)] = '1' | Median<br>*Years* | text, Required |
| | 28 | age_range_male_v2<br>Show the field ONLY if: [participant_age_male_v2(4)] = '1' | Range<br>*Years* | text, Required |
| | 29 | population_age_female_v2 | Female Population | descriptive |
| | 30 | participant_age_female_v2 | How was the age of the female study population reported?<br>*Select all relevant answers* | checkbox, Required<br>1 participant_age_female_v2___1 Mean<br>2 participant_age_female_v2___2 Standard deviation<br>3 participant_age_female_v2___3 Median<br>4 participant_age_female_v2___4 Range |
| | 31 | age_mean_female_v2<br>Show the field ONLY if: [participant_age_female_v2 (1)] = '1' | Mean<br>*Years* | text, Required |
| | 32 | age_stdev_female_v2<br>Show the field ONLY if: [participant_age_female_v2 (2)] = '1' | Standard deviation<br>*Years* | text, Required |

| | | | | |
|---|---|---|---|---|
| | 33 | age_median_female_v2<br><br>Show the field ONLY if:<br>[participant_age_female_v2(3)] = '1' | Median<br>*Years* | text, Required |
| | 34 | age_range_female_v2<br><br>Show the field ONLY if:<br>[participant_age_female_v2(4)] = '1' | Range<br>*Years* | text, Required |
| | 35 | participant_anth_info_v2 | Participant Anthropometric Information | descriptive |
| | 36 | total_population_anthro_v2 | Total Population | descriptive |
| | 37 | participant_height_v2 | How was participant height reported?<br>*Meters* | checkbox, Required<br>1 \| participant_height_v2___1 \| Mean<br>2 \| participant_height_v2___2 \| Standard deviation<br>3 \| participant_height_v2___3 \| Median<br>4 \| participant_height_v2___4 \| Range |
| | 38 | participant_height_mean_v2<br><br>Show the field ONLY if:<br>[participant_height_v2(1)] = '1' | Mean | text, Required |
| | 39 | participant_height_stdev_v2<br><br>Show the field ONLY if:<br>[participant_height_v2(2)] = '1' | Standard deviation | text, Required |
| | 40 | participant_height_median_v2<br><br>Show the field ONLY if:<br>[participant_height_v2(3)] = '1' | Median | text, Required |
| | 41 | participant_height_range_v2<br><br>Show the field ONLY if:<br>[participant_height_v2(4)] = '1' | Range | text, Required |
| | 42 | participant_mass_v2 | Participant mass<br>*Kilograms* | checkbox, Required<br>1 \| participant_mass_v2___1 \| Mean<br>2 \| participant_mass_v2___2 \| Standard deviation<br>3 \| participant_mass_v2___3 \| Median<br>4 \| participant_mass_v2___4 \| Range |
| | 43 | participant_mass_mean_v2<br><br>Show the field ONLY if:<br>[participant_mass_v2(1)] = '1' | Mean | text, Required |
| | 44 | participant_mass_stdev_v2<br><br>Show the field ONLY if:<br>[participant_mass_v2(2)] = '1' | Standard deviation | text, Required |
| | 45 | participant_mass_median_v2<br><br>Show the field ONLY if:<br>[participant_mass_v2(3)] = '1' | Median | text, Required |
| | 46 | participant_mass_range_v2<br><br>Show the field ONLY if:<br>[participant_mass_v2(4)] = '1' | Range | text, Required |
| | 47 | participant_bmi_v2 | Participant BMI<br>*Kilograms/meters^2* | checkbox, Required<br>1 \| participant_bmi_v2___1 \| Mean<br>2 \| participant_bmi_v2___2 \| Standard deviation<br>3 \| participant_bmi_v2___3 \| Median<br>4 \| participant_bmi_v2___4 \| Range |
| | 48 | participant_bmi_mean_v2<br><br>Show the field ONLY if:<br>[participant_bmi_v2(1)] = '1' | Mean | text, Required |
| | 49 | participant_bmi_stdev_v2<br><br>Show the field ONLY if:<br>[participant_bmi_v2(2)] = '1' | Standard deviation | text, Required |
| | 50 | participant_bmi_median_v2<br><br>Show the field ONLY if:<br>[participant_bmi_v2(3)] = '1' | Median | text, Required |

| | | | | |
|---|---|---|---|---|
| | 51 | participant_bmi_range_v2<br>Show the field ONLY if:<br>[participant_bmi_v2(4)] = '1' | Range | text, Required |
| | 52 | male_population_anthro_v2 | Male Population | descriptive |
| | 53 | male_exp_pp_height_v2 | How was participant height reported?<br>*Meters* | checkbox, Required<br>1 male_exp_pp_height_v2___1 Mean<br>2 male_exp_pp_height_v2___2 Standard deviation<br>3 male_exp_pp_height_v2___3 Median<br>4 male_exp_pp_height_v2___4 Range |
| | 54 | male_exp_mean_height_v2<br>Show the field ONLY if:<br>[male_exp_pp_height_v2(1)] = '1' | Mean | text, Required |
| | 55 | male_exp_stdev_height_v2<br>Show the field ONLY if:<br>[male_exp_pp_height_v2(2)] = '1' | Standard deviation | text, Required |
| | 56 | male_exp_median_height_v2<br>Show the field ONLY if:<br>[male_exp_pp_height_v2(3)] = '1' | Median | text, Required |
| | 57 | male_exp_range_height_v2<br>Show the field ONLY if:<br>[male_exp_pp_height_v2(4)] = '1' | Range | text, Required |
| | 58 | male_exp_pp_mass_v2 | How was participant mass reported?<br>*Meters* | checkbox, Required<br>1 male_exp_pp_mass_v2___1 Mean<br>2 male_exp_pp_mass_v2___2 Standard deviation<br>3 male_exp_pp_mass_v2___3 Median<br>4 male_exp_pp_mass_v2___4 Range |
| | 59 | male_exp_mean_mass_v2<br>Show the field ONLY if:<br>[male_exp_pp_mass_v2(1)] = '1' | Mean | text, Required |
| | 60 | male_exp_stdev_mass_v2<br>Show the field ONLY if:<br>[male_exp_pp_mass_v2(2)] = '1' | Standard deviation | text, Required |
| | 61 | male_exp_median_mass_v2<br>Show the field ONLY if:<br>[male_exp_pp_mass_v2(3)] = '1' | Median | text, Required |
| | 62 | male_exp_range_mass_v2<br>Show the field ONLY if:<br>[male_exp_pp_mass_v2(4)] = '1' | Range | text, Required |
| | 63 | male_exp_pp_bmi_v2 | How was participant body mass index reported?<br>*Meters* | checkbox, Required<br>1 male_exp_pp_bmi_v2___1 Mean<br>2 male_exp_pp_bmi_v2___2 Standard deviation<br>3 male_exp_pp_bmi_v2___3 Median<br>4 male_exp_pp_bmi_v2___4 Range |
| | 64 | male_exp_mean_bmi_v2<br>Show the field ONLY if:<br>[male_exp_pp_bmi_v2(1)] = '1' | Mean | text, Required |
| | 65 | male_exp_stdev_bmi_v2<br>Show the field ONLY if:<br>[male_exp_pp_bmi_v2(2)] = '1' | Standard deviation | text, Required |
| | 66 | male_exp_median_bmi_v2<br>Show the field ONLY if:<br>[male_exp_pp_bmi_v2(3)] = '1' | Median | text, Required |

| | | | | |
|---|---|---|---|---|
| | 67 | male_exp_range_bmi_v2<br><br>Show the field ONLY if:<br>[male_exp_pp_bmi_v2(4)] = '1' | Range | text, Required |
| | 68 | female_population_anthro_v2 | Female Population | descriptive |
| | 69 | female_exp_pp_height_v2 | How was participant height reported?<br>*Meters* | checkbox, Required<br>1 female_exp_pp_height_v2___1 Mean<br>2 female_exp_pp_height_v2___2 Standard deviation<br>3 female_exp_pp_height_v2___3 Median<br>4 female_exp_pp_height_v2___4 Range |
| | 70 | female_exp_mean_height_v2<br><br>Show the field ONLY if:<br>[female_exp_pp_height_v2(1)] = '1' | Mean | text, Required |
| | 71 | female_exp_sdtev_height_v2<br><br>Show the field ONLY if:<br>[female_exp_pp_height_v2(2)] = '1' | Standard deviation | text, Required |
| | 72 | female_exp_median_height_v2<br><br>Show the field ONLY if:<br>[female_exp_pp_height_v2(3)] = '1' | Median | text, Required |
| | 73 | female_exp_range_height_v2<br><br>Show the field ONLY if:<br>[female_exp_pp_height_v2(4)] = '1' | Range | text, Required |
| | 74 | female_exp_pp_mass_v2 | How was participant mass reported?<br>*Meters* | checkbox, Required<br>1 female_exp_pp_mass_v2___1 Mean<br>2 female_exp_pp_mass_v2___2 Standard deviation<br>3 female_exp_pp_mass_v2___3 Median<br>4 female_exp_pp_mass_v2___4 Range |
| | 75 | female_exp_mean_mass_v2<br><br>Show the field ONLY if:<br>[female_exp_pp_mass_v2(1)] = '1' | Mean | text, Required |
| | 76 | female_exp_stdev_mass_v2<br><br>Show the field ONLY if:<br>[female_exp_pp_mass_v2(2)] = '1' | Standard deviation | text, Required |
| | 77 | female_exp_median_mass_v2<br><br>Show the field ONLY if:<br>[female_exp_pp_mass_v2(3)] = '1' | Median | text, Required |
| | 78 | female_exp_range_mass_v2<br><br>Show the field ONLY if:<br>[female_exp_pp_mass_v2(4)] = '1' | Range | text, Required |
| | 79 | female_exp_pp_bmi_v2 | How was participant body mass index reported?<br>*Meters* | checkbox, Required<br>1 female_exp_pp_bmi_v2___1 Mean<br>2 female_exp_pp_bmi_v2___2 Standard deviation<br>3 female_exp_pp_bmi_v2___3 Median<br>4 female_exp_pp_bmi_v2___4 Range |
| | 80 | female_exp_mean_bmi_v2<br><br>Show the field ONLY if:<br>[female_exp_pp_bmi_v2(1)] = '1' | Mean | text, Required |

| | | | | |
|---|---|---|---|---|
| | 81 | female_exp_stdev_bmi_v2<br><br>Show the field ONLY if:<br>[female_exp_pp_bmi_v2(2)] = '1' | Standard deviation | text, Required |
| | 82 | female_exp_median_bmi_v2<br><br>Show the field ONLY if:<br>[female_exp_pp_bmi_v2(3)] = '1' | Median | text, Required |
| | 83 | female_exp_range_bmi_v2<br><br>Show the field ONLY if:<br>[female_exp_pp_bmi_v2(4)] = '1' | Range | text, Required |
| | 84 | number_of_controls_v2<br><br>Show the field ONLY if:<br>[cntrl_participants_included] = 1 | Section Header: *Methodology - Control Population Size*<br>Number of controls<br>*Report as: number* | text (number), Required |
| | 85 | number_of_males_control_v2<br><br>Show the field ONLY if:<br>[cntrl_participants_included] = 1 | Number of control males<br>*Report as: number* | text, Required |
| | 86 | number_of_females_control_v2<br><br>Show the field ONLY if:<br>[number_of_controls_v2] = 1 | Number of control females<br>*Report as: number* | text, Required |
| | 87 | cntrl_group_age_v2 | Section Header: *Methodology - Control Demographic Information*<br>Control Group Age | descriptive |
| | 88 | total_cntrl_age_v2 | Total Population | descriptive |
| | 89 | participant_cntrl_age_type_v2<br><br>Show the field ONLY if:<br>[number_of_controls_v2] = 1 | How was the age of control participants reported?<br>*Select all relevant answers* | checkbox, Required<br>1 participant_cntrl_age_type_v2___1 Mean<br>2 participant_cntrl_age_type_v2___2 Standard deviation<br>3 participant_cntrl_age_type_v2___3 Median<br>4 participant_cntrl_age_type_v2___4 Range |
| | 90 | age_cntrl_mean_v2<br><br>Show the field ONLY if:<br>[participant_cntrl_age_type_v2(1)] = '1' | Mean | text, Required |
| | 91 | age_cntrl_stdev_v2<br><br>Show the field ONLY if:<br>[participant_cntrl_age_type_v2(2)] = '1' | Standard deviation | text, Required |
| | 92 | age_cntrl_median_v2<br><br>Show the field ONLY if:<br>[participant_cntrl_age_type_v2(3)] = '1' | Median | text, Required |
| | 93 | age_cntrl_range_v2<br><br>Show the field ONLY if:<br>[participant_cntrl_age_type_v2(4)] = '1' | Range | text, Required |
| | 94 | male_cntrl_age_v2 | Male Population | descriptive |
| | 95 | male_pp_cntrl_age_type_v2 | How was the age of control participants reported?<br>*Select all relevant answers* | checkbox, Required<br>1 male_pp_cntrl_age_type_v2___1 Mean<br>2 male_pp_cntrl_age_type_v2___2 Standard deviation<br>3 male_pp_cntrl_age_type_v2___3 Median<br>4 male_pp_cntrl_age_type_v2___4 Range |
| | 96 | age_cntrl_male_mean_v2<br><br>Show the field ONLY if:<br>[male_pp_cntrl_age_type_v2(1)] = '1' | Mean | text |

| | | | | |
|---|---|---|---|---|
| | 97 | age_cntrl_male_stdev_v2<br>Show the field ONLY if:<br>[male_pp_cntrl_age_type_v2(2)] = '1' | Standard deviation | text |
| | 98 | age_cntrl_male_median_v2<br>Show the field ONLY if:<br>[male_pp_cntrl_age_type_v2(3)] = '1' | Median | text |
| | 99 | age_cntrl_male_range_v2<br>Show the field ONLY if:<br>[male_pp_cntrl_age_type_v2(4)] = '1' | Range | text |
| | 100 | female_cntrl_age_v2 | Female Population | descriptive |
| | 101 | female_pp_cntrl_age_type_v2 | How was the age of control participants reported?<br>*Select all relevant answers* | checkbox, Required<br>1 female_pp_cntrl_age_type_v2___1 Mean<br>2 female_pp_cntrl_age_type_v2___2 Standard deviation<br>3 female_pp_cntrl_age_type_v2___3 Median<br>4 female_pp_cntrl_age_type_v2___4 Range |
| | 102 | age_cntrl_female_mean_v2<br>Show the field ONLY if:<br>[female_pp_cntrl_age_type_v2(1)] = '1' | Mean | text |
| | 103 | age_cntrl_female_stdev_v2<br>Show the field ONLY if:<br>[female_pp_cntrl_age_type_v2(2)] = '1' | Standard deviation | text |
| | 104 | age_cntrl_female_median_v2<br>Show the field ONLY if:<br>[female_pp_cntrl_age_type_v2(3)] = '1' | Median | text |
| | 105 | age_cntrl_female_range_v2<br>Show the field ONLY if:<br>[female_pp_cntrl_age_type_v2(4)] = '1' | Range | text |
| | 106 | cntrl_anth_info_v2 | Control Anthropometric Information | descriptive |
| | 107 | cntrl_anth_info_2_v2 | Total Population | descriptive |
| | 108 | control_height_v2<br>Show the field ONLY if:<br>[number_of_controls_v2] = 1 | Control height<br>*Meters* | text, Required |
| | 109 | control_mass_v2<br>Show the field ONLY if:<br>[number_of_controls_v2] = 1 | Control mass<br>*Kilograms* | text, Required |
| | 110 | control_bmi_v2<br>Show the field ONLY if:<br>[number_of_controls_v2] = 1 | Control body mass index<br>*Kilograms/meters^2* | text, Required |
| | 111 | male_cntrl_anth_info_v2 | Male Population | descriptive |
| | 112 | female_cntrl_anth_info_v2 | Female Population | descriptive |
| | 113 | injury_type_v2 | Section Header: *Injury History*<br>Type of knee injury<br>*Select all relevant answers* | checkbox, Required<br>1 injury_type_v2___1 Intra-articular (general unspecified)<br>2 injury_type_v2___2 Ligament<br>3 injury_type_v2___3 Meniscus<br>4 injury_type_v2___4 Fracture<br>5 injury_type_v2___5 Dislocation<br>6 injury_type_v2___6 Contusion<br>7 injury_type_v2___7 Unclear |

| | | | | |
|---|---|---|---|---|
| | 114 | time_since_injury_type_v2 | How was time between injury incidence and observation/follow up reported?<br>*Select all relevant answers* | checkbox, Required<br>1 time_since_injury_type_v2___1 Mean<br>2 time_since_injury_type_v2___2 Standard deviation<br>3 time_since_injury_type_v2___3 Median<br>4 time_since_injury_type_v2___4 Range<br>5 time_since_injury_type_v2___5 Not reported |
| | 115 | time_since_injury_mean_v2<br>Show the field ONLY if:<br>[time_since_injury_type_v2(1)] = '1' | Mean | text (number), Required |
| | 116 | time_since_injury_stdev_v2<br>Show the field ONLY if:<br>[time_since_injury_type_v2(2)] = '1' | Standard deviation | text (number), Required |
| | 117 | time_since_injury_median_v2<br>Show the field ONLY if:<br>[time_since_injury_type_v2(3)] = '1' | Median | text (number), Required |
| | 118 | time_since_injury_range_v2<br>Show the field ONLY if:<br>[time_since_injury_type_v2(4)] = '1' | Range | text (number), Required |
| | 119 | knee_surgery_v2 | Section Header: *Surgery History*<br>Did the participants have knee surgery? | yesno, Required<br>1 Yes<br>0 No |
| | 120 | surgery_type_v2<br>Show the field ONLY if:<br>[knee_surgery_v2] = '1' | Type of knee surgery<br>*Select all relevant answers* | checkbox, Required<br>1 surgery_type_v2___1 Tendon graft<br>2 surgery_type_v2___2 Meniscectomy<br>3 surgery_type_v2___3 Chondroplasty<br>7 surgery_type_v2___7 Unclear |
| | 121 | time_since_surgery_type_v2<br>Show the field ONLY if:<br>[knee_surgery_v2] = '1' | How was time between between surgery and observation/follow up reported?<br>*Select all relevant answers* | checkbox, Required<br>1 time_since_surgery_type_v2___1 Mean<br>2 time_since_surgery_type_v2___2 Standard deviation<br>3 time_since_surgery_type_v2___3 Median<br>4 time_since_surgery_type_v2___4 Range<br>5 time_since_surgery_type_v2___5 Not reported |
| | 122 | time_since_surgery_mean_v2<br>Show the field ONLY if:<br>[time_since_surgery_type_v2(1)] = 1 | Mean | text, Required |
| | 123 | time_since_surgery_stdev_v2<br>Show the field ONLY if:<br>[time_since_surgery_type_v2(2)] = 1 | Standard deviation | text, Required |
| | 124 | time_since_surgery_median_v2<br>Show the field ONLY if:<br>[time_since_surgery_type_v2(3)] = 1 | Median | text, Required |
| | 125 | time_since_surgery_range_v2<br>Show the field ONLY if:<br>[time_since_surgery_type_v2(4)] = 1 | Range | text, Required |
| | 126 | sport_type_v2 | Section Header: *Sporting History*<br>What sport did the participants engage in at the time of injury?<br>*If not detailed state N/A* | text, Required |
| | 127 | sport_level_v2 | What was the competition level at the time of injury?<br>*If not detailed state N/A* | text, Required |

| | | | | |
|---|---|---|---|---|
| | 128 | was_there_intervention_v2 | Section Header: *Intervention Details*<br>Was there an intervention? | yesno<br>1 Yes<br>0 No |
| | 129 | intervention_type_v2<br>Show the field ONLY if: [was_there_intervention_v2] = 1 | Intervention type<br>*Please detail the type of intervention* | notes, Required |
| | 130 | intervention_setting_v2<br>Show the field ONLY if: [was_there_intervention_v2] = 1 | Intervention setting<br>*E.g., home, hospital, physio clinic* | text, Required |
| | 131 | duration_of_intervention_v2<br>Show the field ONLY if: [was_there_intervention_v2] = 1 | Duration of intervention<br>*Months* | text (number), Required |
| | 132 | adherence_v2<br>Show the field ONLY if: [was_there_intervention_v2] = 1 | Adherence rate | text (number), Required |
| | 133 | drop_out_v2<br>Show the field ONLY if: [was_there_intervention_v2] = 1 | Drop out rate | text (number), Required |
| | 134 | independent_variable_v2 | Independent Variables | descriptive |
| | 135 | what_did_the_study_measure_v2 | What was the independent variable measuring?<br>*Select all relevant answers* | checkbox, Required<br>1 what_did_the_study_measure_v2___1 Physical activity<br>2 what_did_the_study_measure_v2___2 Systemic inflammation<br>3 what_did_the_study_measure_v2___3 Knee joint loading<br>4 what_did_the_study_measure_v2___4 Adiposity<br>5 what_did_the_study_measure_v2___5 Muscle size<br>6 what_did_the_study_measure_v2___6 Muscle strength<br>7 what_did_the_study_measure_v2___7 Intramuscular fat<br>8 what_did_the_study_measure_v2___8 Bone mineral density<br>9 what_did_the_study_measure_v2___9 Unclear (need to contact authors) |
| | 136 | physical_activity_tool_v2<br>Show the field ONLY if: [what_did_the_study_measure_v2(1)] = 1 | Please select the tool used to measure physical activity<br>*Select all relevant answers* | checkbox, Required<br>1 physical_activity_tool_v2___1 Doubly labelled water<br>2 physical_activity_tool_v2___2 Accelerometers<br>3 physical_activity_tool_v2___3 Pedometers<br>4 physical_activity_tool_v2___4 Subjective questionnaires<br>5 physical_activity_tool_v2___5 Diaries<br>6 physical_activity_tool_v2___6 Other (please state below)<br>7 physical_activity_tool_v2___7 Unclear (need to contact authors) |
| | 137 | subjective_pa_tool_type_v2<br>Show the field ONLY if: [physical_activity_tool_v2(4)] = '1' | What was the name of the subjective assessment tool?<br>*Please use full name, not abbreviation. E.g., International Physical Activity Questionnaire* | text, Required |

| | | | | |
|---|---|---|---|---|
| | 138 | inflammation_tool_v2<br>Show the field ONLY if:<br>[what_did_the_study_measure_v2(2)] = '1' | Which cytokine/protein(s) were used to measure systemic inflammation?<br>*Select all relevant answers* | checkbox, Required<br>1 inflammation_tool_v2___1 Interleukin 6 (IL-6)<br>2 inflammation_tool_v2___2 Interleukin 1Beta (IL-1β)<br>3 inflammation_tool_v2___3 Tumor Necrosis Factor Alpha (TNF-α)<br>4 inflammation_tool_v2___4 C-Reactive Protein (CRP)<br>5 inflammation_tool_v2___5 Other (please state below)<br>6 inflammation_tool_v2___6 Unclear (need to contact authors) |
| | 139 | knee_load_tool_v2<br>Show the field ONLY if:<br>[what_did_the_study_measure_v2(3)] = '1' | What tool(s) used to measure knee joint loading?<br>*Select all relevant answers* | checkbox, Required<br>1 knee_load_tool_v2___1 Ground reaction force<br>2 knee_load_tool_v2___2 Moments<br>3 knee_load_tool_v2___3 Inverse dynamics<br>4 knee_load_tool_v2___4 Forward dynamics<br>5 knee_load_tool_v2___5 Other (please state below)<br>6 knee_load_tool_v2___6 Unclear (need to contact authors) |
| | 140 | adiposity_tool_v2<br>Show the field ONLY if:<br>[what_did_the_study_measure_v2(4)] = '1' | What was the tool to measure adiposity? | checkbox, Required<br>1 adiposity_tool_v2___1 Body Mass Index (BMI)<br>2 adiposity_tool_v2___2 Air displacement plethysmograph (ADP)<br>3 adiposity_tool_v2___3 Bioelectrical impedance (BI)<br>4 adiposity_tool_v2___4 Dual energy x-ray absorptiometry (DEXA or DXA)<br>5 adiposity_tool_v2___5 Computerised tomography (CT)<br>6 adiposity_tool_v2___6 Peripheral computerised tomography (pQCT)<br>7 adiposity_tool_v2___7 Other (please state below)<br>8 adiposity_tool_v2___8 Unclear (need to contact authors) |
| | 141 | muslce_tool_v2 | What was the tool used to measure muscle size? | checkbox<br>1 muslce_tool_v2___1 Ultrasound (US)<br>2 muslce_tool_v2___2 Dual energy x-ray absorptiometry (DEXA or DXA)<br>3 muslce_tool_v2___3 Peripheral quantitative computerised tomography (pQCT)<br>4 muslce_tool_v2___4 Computerised tomography (CT)<br>5 muslce_tool_v2___5 Magnetic resonance imaging (MRI)<br>6 muslce_tool_v2___6 Other (please state below)<br>7 muslce_tool_v2___7 Unclear (need to contact authors) |
| | 142 | muscle_strength_tool_v2<br>Show the field ONLY if:<br>[what_did_the_study_measure_v2(5)] = '1' | What was the tool to measure muscle strength? | checkbox, Required<br>1 muscle_strength_tool_v2___1 Isokinetic dynamometry<br>2 muscle_strength_tool_v2___2 Hand-held dynamometer<br>3 muscle_strength_tool_v2___3 Other (please state below)<br>4 muscle_strength_tool_v2___4 Unclear (need to contact authors) |

| | | | | |
|---|---|---|---|---|
| | 143 | intra_muscular_fat_tool_v2<br>Show the field ONLY if:<br>[what_did_the_study_measure_v2(6)] = '1' | What was the tool to measure intra-muscular fat? | checkbox, Required<br>1 intra_muscular_fat_tool_v2___1 Ultrasound (US)<br>2 intra_muscular_fat_tool_v2___2 Computerised tomography (CT)<br>3 intra_muscular_fat_tool_v2___3 Peripheral quantitative computerised tomography (pQCT)<br>4 intra_muscular_fat_tool_v2___4 Magnetic resonance imaging (MRI)<br>5 intra_muscular_fat_tool_v2___5 Other (please state below)<br>6 intra_muscular_fat_tool_v2___6 Unclear (need to contact authors) |
| | 144 | bone_mineral_density_tool_v2<br>Show the field ONLY if:<br>[what_did_the_study_measure_v2(7)] = '1' | What was the tool to measure bone mineral density? | checkbox, Required<br>1 bone_mineral_density_tool_v2___1 Dual energy x-ray absorptiometry (DEXA or DXA)<br>2 bone_mineral_density_tool_v2___2 Computerised tomography (CT)<br>3 bone_mineral_density_tool_v2___3 Peripheral quantitative computerised tomography (pQCT)<br>4 bone_mineral_density_tool_v2___4 Other (please state below)<br>5 bone_mineral_density_tool_v2___5 Unclear (need to contact authors) |
| | 145 | ptoa_disease_head_v2 | Section Header: *Outcome Variables*<br>PTOA Disease | descriptive |
| | 146 | ptoa_disease_v2 | Does the study measure PTOA disease? | yesno<br>1 Yes<br>0 No |
| | 147 | radiography_diagnostic_v2 | Radiography diagnostic system | checkbox, Required<br>1 radiography_diagnostic_v2___1 Kellgren and Lawrence (K/L) system<br>2 radiography_diagnostic_v2___2 Osteoarthritis Research Society International (OARSI) Atlas criteria<br>3 radiography_diagnostic_v2___3 Unclear (need to contact authors) |
| | 148 | mri_diagnostic_v2 | Magnetic resonance imaging diagnostic system | checkbox, Required<br>1 mri_diagnostic_v2___1 MRI Osteoarthritis Knee Score (MOAKS)<br>2 mri_diagnostic_v2___2 Whole Organ Magnetic Resonance Imaging Score (WORMS)<br>3 mri_diagnostic_v2___3 Boston Leeds OA Knee Score (BLOKS)<br>4 mri_diagnostic_v2___4 Unclear (need to contact authors) |

| | | | | |
|---|---|---|---|---|
| | 149 | indicators_of_disease_v2 | Indicators of structural deterioration preceding or exacerbating PTOA disease | checkbox, Required<br>1 \| indicators_of_disease_v2___1 \| MRI quantified cartilage thickness and volume<br>2 \| indicators_of_disease_v2___2 \| T1rho mapping<br>3 \| indicators_of_disease_v2___3 \| T2 mapping<br>4 \| indicators_of_disease_v2___4 \| Delayed contrast-enhanced MRI of cartilage (dGEMRIC)<br>5 \| indicators_of_disease_v2___5 \| Unclear (need to contact authors) |
| | 150 | systemic_biomarkers_v2 | Systemic biomarkers indicating structural deterioration of knee PTOA disease | checkbox<br>1 \| systemic_biomarkers_v2___1 \| Matrix Metalloprotease 3 (MMP-3)<br>2 \| systemic_biomarkers_v2___2 \| Matrix Metalloprotease 13 (MMP-13)<br>3 \| systemic_biomarkers_v2___3 \| N-Propeptide of Collagen IIA (PIIANP)<br>4 \| systemic_biomarkers_v2___4 \| C-Telopeptide of Type II Collagen (CTX)<br>5 \| systemic_biomarkers_v2___5 \| C-Propeptide of Type II Collagen (CP-II)<br>6 \| systemic_biomarkers_v2___6 \| CTX:CP-II<br>7 \| systemic_biomarkers_v2___7 \| Collagen Type II Cleavage Product (C2C)<br>8 \| systemic_biomarkers_v2___8 \| Col2-3/4 C-terminal cleavage product of types I and II collagen (C1,2C)<br>9 \| systemic_biomarkers_v2___9 \| Hyaluronic Acid (HA)<br>10 \| systemic_biomarkers_v2___10 \| Chondroitin Sulphate 846 Epitope (CS846)<br>11 \| systemic_biomarkers_v2___11 \| Aggrecan164 or Aggrecan Fragments (ARGS)<br>12 \| systemic_biomarkers_v2___12 \| Cartilage Oligomeric Matrix Protein (COMP)<br>13 \| systemic_biomarkers_v2___13 \| Unclear (need to contact authors) |
| | 151 | outcome | Section Header: *Results*<br>Outcome 1<br>*Primary Outcome* | descriptive |
| | 152 | outcome_name | Please state what the independent variable is (e.g., physical activity), how it is measured (e.g., accelerometery), and the make/model of the measurement tool. | notes |
| | 153 | iv_name | Please state what the outcome variable is (e.g., c-telopeptide of type II collagen), how it is measured (e.g., blood serum), and the make/model of the measurement tool. | notes |
| | 154 | sample_size | Sample size for the test | text |

| | | | | |
|---|---|---|---|---|
| | 155 | stat_test_1 | What was the type of statistical test used? | radio<br>1 Difference test (t-test, ANOVA)<br>2 Correlation<br>3 Regression<br>4 Odds Ratio<br>5 Risk Ratio |
| | 156 | outcome_mean | Injured mean | text |
| | 157 | outcome_stdev | Injured standard deviation | text |
| | 158 | control_mean | Control mean | text |
| | 159 | cntrl_outcome_stdev | Control standard deviation | text |
| | 160 | p_correlation_coefficient | Pearson correlation coefficient | text |
| | 161 | s_correlation_coefficient | Spearman's correlation coefficient | text |
| | 162 | odds_ratio | Odds ratio | text |
| | 163 | risk_ratio | Risk ratio | text |
| | 164 | outcome_2 | Outcome 2<br>*Primary Outcome* | descriptive |
| | 165 | outcome_name_2 | Please state what the independent variable is (e.g., physical activity), how it is measured (e.g., accelerometery), and the make/model of the measurement tool. | notes |
| | 166 | iv_name_2 | Please state what the outcome variable is (e.g., c-telopeptide of type II collagen), how it is measured (e.g., blood serum), and the make/model of the measurement tool. | notes |
| | 167 | sample_size_2 | Sample size for the test | text |
| | 168 | stat_test_2 | What was the type of statistical test used? | radio<br>1 Difference test (t-test, ANOVA)<br>2 Correlation<br>3 Regression<br>4 Odds Ratio<br>5 Risk Ratio |
| | 169 | outcome_mean_2 | Injured mean | text |
| | 170 | outcome_stdev_2 | Injured standard deviation | text |
| | 171 | control_mean_2 | Control mean | text |
| | 172 | cntrl_outcome_stdev_2 | Control standard deviation | text |
| | 173 | p_correlation_coefficient_2 | Pearson correlation coefficient | text |
| | 174 | s_correlation_coefficient_2 | Spearman's correlation coefficient | text |
| | 175 | odds_ratio_2 | Odds ratio | text |
| | 176 | risk_ratio_2 | Risk ratio | text |
| | 177 | outcome_3 | Outcome 3<br>*Primary Outcome* | descriptive |
| | 178 | outcome_name_3 | Please state what the independent variable is (e.g., physical activity), how it is measured (e.g., accelerometery), and the make/model of the measurement tool. | notes |
| | 179 | iv_name_3 | Please state what the outcome variable is (e.g., c-telopeptide of type II collagen), how it is measured (e.g., blood serum), and the make/model of the measurement tool. | notes |
| | 180 | sample_size_3 | Sample size for the test | text |
| | 181 | stat_test_3 | What was the type of statistical test used? | radio<br>1 Difference test (t-test, ANOVA)<br>2 Correlation<br>3 Regression<br>4 Odds Ratio<br>5 Risk Ratio |
| | 182 | outcome_mean_3 | Injured mean | text |
| | 183 | outcome_stdev_3 | Injured standard deviation | text |
| | 184 | control_mean_3 | Control mean | text |
| | 185 | cntrl_outcome_stdev_3 | Control standard deviation | text |

| | | | | |
|---|---|---|---|---|
| | 186 | p_correlation_coefficient_3 | Pearson correlation coefficient | text |
| | 187 | s_correlation_coefficient_3 | Spearman's correlation coefficient | text |
| | 188 | odds_ratio_3 | Odds ratio | text |
| | 189 | risk_ratio_3 | Risk ratio | text |
| | 190 | conclusion01 | Section Header: *Conclusion*<br>Please state the author's conclusions | notes |
| | 191 | other_comments | Please state any comments/conclusions you have | notes |
| | 192 | data_extraction_2_complete | Section Header: *Form Status*<br>Complete? | dropdown<br>0 Incomplete<br>1 Unverified<br>2 Complete |
