# Supplementary material for: Understanding the role of physical activity on the pathway from intra-articular knee injury to post-traumatic osteoarthritis disease in young people: a scoping review protocol
Source: BMJ Open. 2023 Mar 3;13(3):e067147. doi: 10.1136/bmjopen-2022-067147 (PMC9990625; doi:10.1136/bmjopen-2022-067147)
Supplement: Supplementary data [file bmjopen-2022-067147supp009.pdf]

Supplementary Material 8. Strength of the treatment response or relationship between variables

Interpreting effect size values[1,2,3,4]

Effect Size Measure	Effect Size			
	Small	Medium	Large	Very Large
Odds Ratio	1.5	2.5	4	10
Cohen's *d*	0.2	0.5	0.8	1.3
r	0.1	0.3	0.5	0.7
Cohen's *f*	0.1	0.25	0.4	-
Eta-squared	0.01	0.06	0.14	-

1. Maher JM, Markey JC, Ebert-May D. The Other Half of the Story: Effect Size Analysis in Quantitative Research. *CBE Life Sci Educ* 2013;12(3):345-51. doi: 10.1187/cbe.13-04-0082

2. Rosenthal JA. Qualitative Descriptors of Strength of Association and Effect Size. J Soc Serv Res 1996;21(4):37-59. doi: 10.1300/j079v21n04_02

3. Cohen J. A power primer. Psychological bulletin 1992;112(1):155.

4. Cohen J. Statistical Power Analysis for the Behavioral Sciences. 2nd Edition ed. New York: Routledge 1988.
