# Supplementary material for: Understanding the role of physical activity on the pathway from intra-articular knee injury to post-traumatic osteoarthritis disease in young people: a scoping review protocol
Source: BMJ Open. 2023 Mar 3;13(3):e067147. doi: 10.1136/bmjopen-2022-067147 (PMC9990625; doi:10.1136/bmjopen-2022-067147)
Supplement: Supplementary data [file bmjopen-2022-067147supp010.pdf]

Supplementary Material 9. Adapted Grading of Recommendations, Assessment, Development and Evaluations (GRADE) Guidelines for Scoping Review

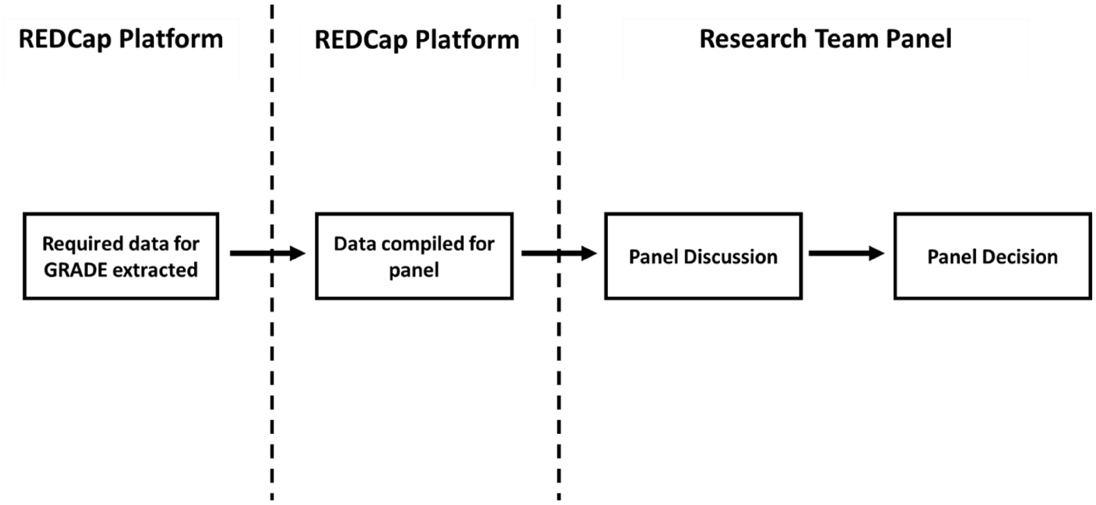

Draft - Adapted Grading of Recommendations, Assessment, Development and Evaluations (GRADE) Guidelines for Scoping Review

Overview:

Grading of Recommendations, Assessment, Development and Evaluations (GRADE)[1-3] is structured as:

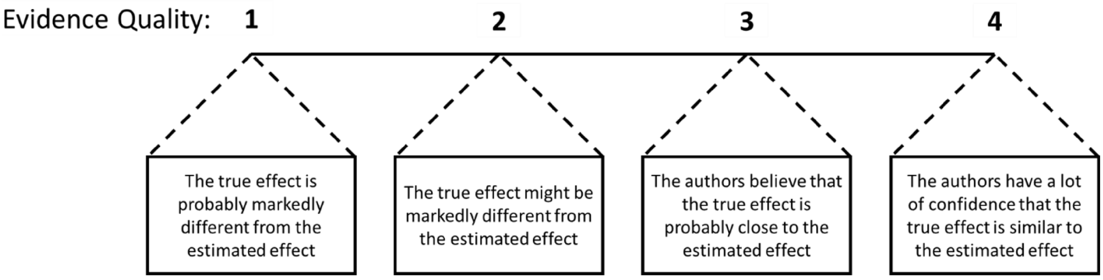

In GRADE, randomised control trials (RCTs) are the highest quality of research evidence (4), whilst observational studies (e.g., analytical cross-sectional studies) are the lowest form of evidence (1).[1-3] From there, individual studies will either be downgraded or upgraded for quality of evidence. For example, if an RCT is not downgraded it will provide the highest quality of evidence (4).[1-3]

In this review, the GRADE criteria will be applied to the research question '***what is the role of physical activity in the trajectory from intra-articular knee injury to post-traumatic osteoarthritis in young men and women?***' and will be considered through each of the following themes:

- Physical activity
- Systemic inflammation
- Adiposity
- Knee joint loading
- Lower body muscle strength
- Muscle size
- Intra-muscular fat
- Bone mineral density

Downgrade quality of evidence

1. Risk of Bias (RoB)

According to Ma *et al.*,[4] "Quality includes internal and external validity, while methodological quality usually refers to internal validity. Internal validity is also recommended as "risk of bias (RoB)" by the Cochrane Collaboration."

In this review, we will use the Joanna Briggs Institute (JBI) Critical Appraisal Checklists (Supplementary Material 6) to assess the RoB of each individual study. The Critical Appraisal Checklists apply to:

- Analytical Cross Sectional Studies[5]
- Case Control Studies[5]
- Cohort Studies[5]
- Studies Reporting Prevalence Data[6]
- Quasi-Experimental Studies[7]
- Randomised Control Trials[7]
- Systematic Review and Research Synthesis[8]

For the purpose of this review, we will classify the scores generated from JBI Critical Appraisal Checklists as either:

- Very low quality (0 – 25.0%)
- Low quality (>25.0 – 50.0%)
- Moderate quality (>50.0 – 75.0%)
- High quality (75.0 – 100%)

This scoring system will be applied to studies on a case-by-case basis.

Following application of the scoring system to all studies, we will adopt GRADE guidelines by downgrading studies if, collectively for the outcome, there is either a serious risk of bias or a very serious risk of bias. The scoring criteria for this review will be as follows:

- (**0**) No serious risk of bias overall = all studies scored moderate to high quality
- (**-1**) Serious risk of bias overall = more than 25% of studies considered low or very low quality
- (**-2**) Very serious risk of bias overall = More than 50% of studies considered low or very low quality

2. Inconsistency

Study results will be considered consistent when directions (e.g., positive or negative association), are considered similar enough to draw uniform conclusions. In this review, consistency in direction will be defined as >75% of the included studies for each theme demonstrating the same positive effect only or negative effect only.

- (**-1**) Serious risk of inconsistency when ≤75% of the included studies have the same direction of effect.
- (**-2**) Very serious risk of inconsistency when there is only one or no studies.

3. Indirectness

GRADE distinguishes several types of indirectness[9]. In this review, indirectness may be due to any of the factors detailed below:

Independent variable (*applicability*)[9]

- (**-1**) Serious risk of indirectness when the independent variable typically used by studies is deemed less valid than the gold standard (e.g., adiposity inferred from body mass index rather than dual-energy x-ray absorptiometry)
- (**-1**) Serious risk of indirectness an intervention cannot be completed in multiple settings (e.g., hospital and home based)

If no studies test a relevant intervention to the research question, or there is no research investigating any of the identified themes, we will conclude that the effects are uncertain.

Differences in outcome measures (*surrogate outcomes*)[9]

- (**-1**) Serious risk of indirectness when research typically uses of a surrogate measure which is associated with knee PTOA (e.g., systemic concentrations of type-II collagen biomarker), rather than directly diagnosing PTOA
- (**-1)** Serious risk of indirectness when studies do not provide information on full time course of PTOA development (e.g., only short-term effects of a physical activity intervention on a surrogate provide, and no studies investigating the long-term impact of PTOA diagnosis)

Indirect comparison[9]

- (**-1**) Serious risk of indirectness by lack of comparisons between physical activity types or exercise modalities
- (**-1**) Serious risk of indirectness by lack of comparison to alternative approaches (e.g., pharmaceutical driven pain relief)

4. Imprecision

As the data will not be combined in a meta-analysis, the following qualitative assessment will be applied to determine imprecision[10]:

- (**-1**) Serious risk of imprecision when the lower threshold of 95% CI reduces the effect to negligible

- (**-1**) Serious risk of imprecision when effect of estimate presented in only one or two studies or estimate comes from one or two events
- (**-2**) Very serious risk of imprecision if the lower threshold of 95% CI is consistent with conflicting conclusions
- (**-2**) Very serious risk of imprecision if CIs were not provided in any of the studies

5. Publication Bias

Assessment of publication bias using statistical methods or funnel plots will not be possible due to heterogeneity of the studies in this review. Publication bias will be screened for qualitatively as detailed in Chapter 7 of the Cochrane Handbook.

Upgrade quality of evidence

1. Large Magnitude of Effect

In this review, when a very large magnitude of effect is observed (assessed using Supplementary Material 8), there is greater certainty that there is at least a small effect.[11]

- (**+1**) when the effect size is large
- (**+2**) when the effect size is very large

The time-course of outcomes will also be considered in this review[12] when upgrading due to large magnitude of effect and devised into:

- (**+1**) Short-term (e.g., response to single exercise session/alteration of joint loading)
  - Systemic biomarkers in synovial fluid/serum/plasma/urine
  - MRI or US indicates deterioration
  - Symptoms or function

- (**+1**) Medium-term (e.g., response to 12-week activity intervention)
  - Systemic biomarkers in synovial fluid/serum/plasma/urine
  - MRI or US indicates deterioration
  - Symptoms or function

- (**+2**) Long-term (e.g., Body Mass Index at one-year post-injury and PTOA development in the years following)
  - X-Ray or MRI PTOA Diagnosis

This is because biomarkers concentrations changes can occur rapidly due to mechanical compression,[13-16] or an exercise-based intervention.[17] Furthermore, the basal protein synthesis rates in various musculoskeletal tissues (including cartilage, meniscus, bone) are within the same range of skeletal muscle protein synthesis rates[18] which may indicate pathology much faster than it takes for PTOA to fully develop.

Lastly, if there are major concerns regarding risk of bias, consistency, precision, or publication bias, in addition to being downgraded, the quality of evidence will not be upgraded via magnitude of effect.[12]

2. Dose-Response Gradient

This increases our confidence in the findings of observational studies and thus enhance the assigned quality of evidence.[12] This is best represented by RCTs and quasi-experimental trials.

- (**+1**) when there is evidence of a dose (e.g., physical activity-based intervention) and response (e.g., increased concentrations of aggrecan

synthesis, lower concentrations of type-II collagen breakdown, slower MRI defined joint tissue degeneration)

3. All residual confounding would decrease magnitude of effect (in situations with an effect)

All plausible residual confounders or biases would reduce a demonstrated effect, or suggest a spurious effect when results show no effect.[12]

REFERENCES

1. Guyatt GH, Oxman AD, Kunz R, et al. What is “quality of evidence” and why is it important to clinicians? *BMJ* 2008;336(7651):995-98. doi: 10.1136/bmj.39490.551019.be
2. Guyatt GH, Oxman AD, Vist GE, et al. GRADE: an emerging consensus on rating quality of evidence and strength of recommendations. *BMJ* 2008;336(7650):924-26. doi: 10.1136/bmj.39489.470347.ad
3. Guyatt G, Oxman AD, Akl EA, et al. GRADE guidelines: 1. Introduction-GRADE evidence profiles and summary of findings tables. *J Clin Epidemiol* 2011;64(4):383-94. doi: 10.1016/j.jclinepi.2010.04.026 [published Online First: 20101231]
4. Ma L-L, Wang Y-Y, Yang Z-H, et al. Methodological quality (risk of bias) assessment tools for primary and secondary medical studies: what are they and which is better? *Mil Med Res* 2020;7(1) doi: 10.1186/s40779-020-00238-8
5. Moola S, Munn Z, Tufanaru C, et al. Chapter 7: Systematic reviews of etiology and risk. In: Aromataris E, Munn Z, eds. JBI Manual for Evidence Synthesis: JBI, 2020.
6. Munn Z, Moola S, Lisy K, et al. Methodological guidance for systematic reviews of observational epidemiological studies reporting prevalence and cumulative incidence data. *Int J Evid Based Healthc* 2015;13(3):147-53. doi: 10.1097/XEB.0000000000000054
7. Tufanaru C, Munn Z, Aromataris E, et al. Chapter 3: Systematic reviews of effectiveness. In: Aromataris E, Munn Z, eds. JBI Manual for Evidence Synthesis: JBI, 2020.
8. Aromataris E, Fernandez R, Godfrey CM, et al. Summarizing systematic reviews: methodological development, conduct and reporting of an umbrella review approach. *Int J Evid Based Healthc* 2015;13(3):132-40. doi: 10.1097/XEB.0000000000000055
9. Guyatt GH, Oxman AD, Kunz R, et al. GRADE guidelines: 8. Rating the quality of evidence—indirectness. *J Clin Epidemiol* 2011;64(12):1303-10. doi: 10.1016/j.jclinepi.2011.04.014
10. Guyatt GH, Oxman AD, Kunz R, et al. GRADE guidelines 6. Rating the quality of evidence—imprecision. *J Clin Epidemiol* 2011;64(12):1283-93. doi: 10.1016/j.jclinepi.2011.01.012

11. Reed S, Guyatt G. What is GRADE? BMJ: BMJ; 2022 [Available from: https://bestpractice.bmj.com/info/toolkit/learn-ebm/what-is-grade/ accessed 20/06/2022 2022.
12. Guyatt GH, Oxman AD, Sultan S, et al. GRADE guidelines: 9. Rating up the quality of evidence. *J Clin Epidemiol* 2011;64(12):1311-16. doi: 10.1016/j.jclinepi.2011.06.004
13. Mundermann A, Dyrby CO, Andriacchi TP, et al. Serum concentration of cartilage oligomeric matrix protein (COMP) is sensitive to physiological cyclic loading in healthy 9adults. *Osteoarthritis Cartilage* 2005;13(1):34-38. doi: 10.1016/j.joca.2004.09.007 [published Online First: 2005/01/11]
14. Mündermann A, King KB, Smith RL, et al. Change in serum COMP concentration due to ambulatory load is not related to knee OA status. *J Orthop Res* 2009;27(11):1408-13. doi: 10.1002/jor.20908 [published Online First: 2009/05/08]
15. Mundermann A, Klenk C, Billich C, et al. Changes in Cartilage Biomarker Levels During a Transcontinental Multistage Footrace Over 4486 km. *Am J Sports Med* 2017;45(11):2630-36. doi: 10.1177/0363546517712945 [published Online First: 2017/06/27]
16. Roberts HM, Law RJ, Thom JM. The time course and mechanisms of change in biomarkers of joint metabolism in response to acute exercise and chronic training in physiologic and pathological conditions. *Eur J Appl Physiol* 2019;119(11-12):2401-20. doi: 10.1007/s00421-019-04232-4 [published Online First: 2019/10/28]
17. Azukizawa M, Ito H, Hamamoto Y, et al. The effects of well-rounded exercise program on systemic biomarkers related to cartilage metabolism. *Cartilage* 2019;10(4):451-58. doi: 10.1177/1947603518767998 [published Online First: 2018/04/13]
18. Smeets JSJ, Horstman AMH, Vles GF, et al. Protein synthesis rates of muscle, tendon, ligament, cartilage, and bone tissue in vivo in humans. *PLoS One* 2019;14(11):e0224745. doi: 10.1371/journal.pone.0224745 [published Online First: 2019/11/08]
