# Supplementary material for: Understanding the role of physical activity on the pathway from intra-articular knee injury to post-traumatic osteoarthritis disease in young people: a scoping review protocol
Source: BMJ Open. 2023 Mar 3;13(3):e067147. doi: 10.1136/bmjopen-2022-067147 (PMC9990625; doi:10.1136/bmjopen-2022-067147)
Supplement: Author's manuscript [file bmjopen-2022-067147.draft_revisions.pdf]

BMJ Open

BMJ Open is committed to open peer review. As part of this commitment we make the peer review history of every article we publish publicly available.

When an article is published we post the peer reviewers' comments and the authors' responses online. We also post the versions of the paper that were used during peer review. These are the versions that the peer review comments apply to.

The versions of the paper that follow are the versions that were submitted during the peer review process. They are not the versions of record or the final published versions. They should not be cited or distributed as the published version of this manuscript.

BMJ Open is an open access journal and the full, final, typeset and author-corrected version of record of the manuscript is available on our site with no access controls, subscription charges or pay-per-view fees (http://bmjopen.bmj.com).

If you have any questions on BMJ Open's open peer review process please email
info.bmjopen@bmj.com

BMJ Open

Understanding the Role of Physical Activity on the Pathway from Intra-Articular Knee Injury to Post-Traumatic Osteoarthritis in Young People: A Scoping Review Protocol

Journal:	*BMJ Open*
Manuscript ID	bmjopen-2022-067147
Article Type:	Protocol
Date Submitted by the Author:	02-Aug-2022
Complete List of Authors:	Morgan, Karl; University of Bath, Department for Health; Centre for Health and Injury and Illness Prevention in Sport (CHI2PS), University of Bath<br>Cowburn, James; University of Bath, Department for Health<br>Farrow, Matthew; University of Bath, Department for Health; Centre for Nutrition and Exercise Metabolism (CNEM), University of Bath<br>Cazzola, Dario; University of Bath, Department for Health; Centre for the Analysis of Motion, Entertainment Research and Applications (CAMERA), University of Bath<br>Walhin, Jean-Philippe; University of Bath, Department for Health; Centre for Nutrition and Exercise Metabolism (CNEM), University of Bath<br>McKay, Carly; University of Bath, Department for Health; Centre for Health and Injury and Illness Prevention in Sport (CHI2PS), University of Bath
Keywords:	Knee < ORTHOPAEDIC & TRAUMA SURGERY, Orthopaedic sports trauma < ORTHOPAEDIC & TRAUMA SURGERY, SPORTS MEDICINE, RHEUMATOLOGY, Musculoskeletal disorders < ORTHOPAEDIC & TRAUMA SURGERY

SCHOLARONE™
Manuscripts

Title Page

Title: Understanding the Role of Physical Activity on the Pathway from Intra-Articular Knee Injury to Post-Traumatic Osteoarthritis in Young People: A Scoping Review Protocol

Authors: Karl Morgan*[1 2 3 5], Dr James Cowburn[1], Dr Matthew Farrow[1 3 5], Dr Dario Cazzola[1 2 4], Dr Jean-Philippe Walhin[1 3], Dr Carly McKay[1 2 5]

Department for Health, University of Bath, Bath, UK[1]

Centre for Health and Injury and Illness Prevention in Sport (CHI²PS), Department for Health, University of Bath, Bath, UK[2]

Centre for Nutrition and Exercise Metabolism (CNEM), Department for Health, University of Bath, Bath, UK[3]

Centre for the Analysis of Motion, Entertainment Research and Applications (CAMERA), University of Bath, Bath, UK[4]

Centre for Sport, Exercise and Osteoarthritis Versus Arthritis, University of Bath, Bath, UK[5]

***Corresponding Author:** kdm40@bath.ac.uk, Department for Health, Claverton Down, Bath, UK, BA2 7AY

Word Count: 2528

For peer review only

ABSTRACT

Introduction

The prevalence of intra-articular knee injuries and reparative surgeries is increasing in many countries. Alarmingly, there is a risk of developing post-traumatic osteoarthritis (PTOA) after sustaining a serious intra-articular knee injury. Although physical inactivity is suggested as a risk factor contributing to the high prevalence of the condition, there is a paucity of research characterising the association between PA and joint health. Consequently, the primary aim of this review will be to identify and present available empirical evidence regarding the association between PA and joint degeneration after intra-articular knee injury and summarise the evidence using an adapted Grading of Recommendations, Assessment, Development and Evaluations. The secondary aim will be to identify potential mechanistic pathways through which PA could influence PTOA pathogenesis. The tertiary aim will be to highlight gaps in current understanding of the association between PA and joint degeneration following joint injury.

Methods

A scoping review will be conducted using the Preferred Reporting Items for Systematic reviews and Meta-Analyses extension for Scoping Reviews checklist and best-practice recommendations. The review will be guided by the following research question: what is the role of PA in the trajectory from intra-articular knee injury to post-traumatic osteoarthritis in young men and women? We will identify primary research studies, meta-analyses, and grey literature by searching the electronic databases Scopus, Embase, Medline, Web of Science: all databases, SPORTDiscus, Physiotherapy Evidence Database (PEDro), and Google Scholar. Reviewing pairs will screen abstracts, full texts, and will extract data. Data will be presented descriptively using charts, graphs, plots, and tables.

Ethics and dissemination

For peer review only - http://bmjopen.bmj.com/site/about/guidelines.xhtml

This research does not require ethical approval due to the data being published and publicly available. This review will be submitted for publication in a peer-reviewed sports medicine journal irrespective of discoveries and disseminated through scientific conference presentations and social media.

Registration

This scoping review is registered on the Open Science Framework (https://osf.io/84pnh/)

Strengths and limitations of this study

- A scoping review is the most appropriate approach to identify and describe current evidence on the complex relationship between PA and post-traumatic osteoarthritis due to the multi-faceted aetiology underpinning the condition.
- This novel research lays an important foundation for further empirical research by mapping current evidence in the literature, examining potential mechanistic pathways using a multi-disciplinary approach, and identifying gaps in current knowledge.
- Due to the broad nature of a scoping review, this approach may limit the ability to comprehensively elucidate pathological mechanisms.

For peer review only - http://bmjopen.bmj.com/site/about/guidelines.xhtml

INTRODUCTION

Knee injuries, resulting from both contact and non-contact mechanisms,[1-9] are a common orthopaedic complaint in young, sporting populations worldwide[10] and often involve damage to multiple tissues inside the joint.[11-14] Reconstruction surgery is commonly used to treat the injured tissue,[9 11 13 15 16] with hundreds of thousands of knee injuries and reparative reconstruction procedures performed worldwide every year. [13 17-19] Current data suggests rates of intra-articular injury and tissue repair are rising in several countries,[17-19] with knee injuries predominantly affecting people under 40 years of age, [13 17-19] which is alarming considering injury greatly increases the risk of developing knee osteoarthritis (OA).[20] Knee OA is a highly prevalent disorder[21] which is becoming more common,[21-23] and is composed of both a disease and an illness component.[24] OA disease is associated with illness,[25] and the threshold for disease inducing illness is low. [24] However, the presence of illness does not always indicate structural deterioration and disease does not always induce symptomology,[26 27] with history of knee injury alone enough to induce illness.[27]

Knee OA affecting people with a history of intra-articular injury is defined as post-traumatic osteoarthritis (PTOA).[28-31] There is 10-fold increased odds of experiencing PTOA disease between 3-10 years following a knee injury[27]. Further, the prevalence of the disease 11-17 years following knee trauma (48-52%)[29 32] is concerning when considering average age at diagnosis for idiopathic knee OA is 55 years,[33] which indicates a typical case of PTOA would increase disease burden duration. After disease diagnosis, the joint may deteriorate to end stage OA, resulting in increased odds for total knee replacement (TKR), which occurs at a noticeably younger age in populations with an injury history than those without.[34] Knee OA is the primary cause for TKR[35] and the number of TKRs in people aged under 55 is increasing.[36] As TKRs typically last 25 years,[37] these younger patients experiencing PTOA may sustain subsequent prosthesis failure within their lifetime.[38]

In terms of PTOA illness, after intra-articular knee injury or anterior cruciate ligament reconstruction there is a high prevalence of knee symptomology (e.g., pain, stiffness, swelling, crepitus) and loss of function.[39-43] Knee pain is described as the cardinal symptom of OA,[44] and often drives people to seek medical care.[45-47] When applying the pain-avoidance model,[48] developed from idiopathic

For peer review only - http://bmjopen.bmj.com/site/about/guidelines.xhtml

knee OA populations, to PTOA, experiencing disease and illness due to PTOA may exacerbate loss of function across the lifespan, thereby accelerating the decline below the threshold of independence (Figure 1).[46 49] Consequently, understanding joint deterioration and characterising risk factors for PTOA in knee injury populations, with a view to prevent or slow condition progression, is paramount.

FIGURE 1. HYPOTHETICAL TRAJECOTRY OF INJURY TO LIFELONG DISEASE BURDEN

Physical inactivity is postulated as one of several risk factors that may compound joint degeneration following intra-articular knee injury.[50 51] Subjective and objective measurements of physical activity (PA) levels indicate reduced participation following knee injury[52-60] and many individuals do not return to the same level of sports participation or return to sport at all after anterior cruciate ligament reconstruction.[61-63] Therapeutic exercise is a core treatment for knee OA, and is considered beneficial to joint health,[64] whilst physical inactivity has been touted as a key contributor to joint deterioration in idiopathic OA.[22 50] However, a recent narrative review suggested there is little characterisation of whether there is a direct association between PA and joint health following injury.[51] Furthermore, although PA often involves knee joint loading (e.g., walking or running), there is limited research investigating the association between cumulative joint loading (dictated by PA) and joint health after knee joint trauma. Lastly, there are several physiological maladaptations that can occur after a traumatic knee injury, such as; increased adiposity,[41 54 59 65-67] knee extensor and flexor weakness,[68-70] muscle atrophy,[71 72] intra-muscular fat accumulation,[73 74] and bone mineral density reduction,[75] which could influence joint health. However, these associations with PTOA are yet to be fully understood. Considering PA is causally associated with systemic inflammation,[76-78] adiposity,[79] muscle size[80 81] and quality,[81-86] and bone mineral density[87 88] in uninjured populations, exploring the associations between these factors in injured populations may help to explain the complex relationship between PA and joint health following intra-articular knee injury.

For peer review only - http://bmjopen.bmj.com/site/about/guidelines.xhtml

For peer review only

Consequently, this protocol documents the procedure for conducting the review. The primary aim of the scoping review will be to identify and present available empirical evidence regarding the association between PA and joint degeneration after intra-articular knee injury and summarise the evidence using an adapted Grading of Recommendations, Assessment, Development and Evaluations (GRADE).[89-91] The secondary aim will be to establish potential mechanistic pathways through which PA could influence PTOA pathogenesis. The tertiary aim will be to highlight gaps in current understanding of the association between PA and joint health following injury.

METHODS

This scoping review is an exploratory project that systematically maps the literature relevant to the research question, identifies key concepts, theories and sources of evidence, and gaps in the research.[92 93] This protocol has been prepared to ensure the transparency of the review process and will be used as a guide when conducting the review and reporting the outcomes to minimise the risk of reporting bias.[94 95] Further, this protocol has been developed in accordance with the adapted Preferred Reporting Items for Systematic Review and Meta-Analysis Protocol (PRISMA-P)[96 97] for scoping reviews[95] (Supplementary Material 1). This review is registered on Open Science Framework (https://osf.io/84pnh/) and the final output will be underpinned both by the Preferred Reporting Items for Systematic reviews and Meta-Analyses extension for Scoping Reviews (PRISMA-ScR)[98] and best practice recommendations.[99-101] Deviations from this protocol when conducting the review will be documented and published in supplementary material of the final output.

Patient and Public Involvement

During protocol design and planning, there was no patient or public involvement. However, previous research conducted by our group has highlighted people with history of a knee injury can experience a lack of long-term support and often alter their physical activity participation because of their previous knee injury.[102] Furthermore, a recent narrative review[51] highlighted the need to

For peer review only - http://bmjopen.bmj.com/site/about/guidelines.xhtml

characterise the relationship between physical activity and joint health following injury. Considering the complexity and multifaceted relationship between physical activity and joint health, and the heterogenous study designs used by researchers in the topic, the research team concluded a scoping review would provide a coherent and informative assessment of current knowledge regarding physical activity and joint health in those at risk of experiencing post-traumatic osteoarthritis.

Framework

This review will follow the Arksey and O'Malley framework and will adhere to the Joanna Briggs Institute quality assessment recommendations (Figure 2).[99 103]

FIGURE 2. OUTLINE OF THE STUDY METHODOLOGY

Stage 1: Identifying the Research Question

The review will be guided by the following research question: What is the role of PA on the trajectory from intra-articular knee injury to post-traumatic osteoarthritis in young men and women? As PTOA is a joint condition with a 'disease' component and an 'illness' component,[24] we will describe the association between PA with both PTOA disease and illness. Disease will be defined as: 'abnormalities of the structure and function of body organs and systems that can be specifically identified and described by reference to certain biological, chemical or other evidence'.[104] Illness will be defined as: 'the human response to disease',[104] which describes reported symptomology (e.g., pain, stiffness, swelling, crepitus) and disability (e.g., functional or mental impairment which acts as a barrier to life activities including self-sustaining tasks).[24] Within the research question, the following themes have been identified *a priori*: PA, systemic inflammation, knee joint load, adiposity, strength (lower body), intra-muscular adipose tissue (lower body), muscle size (lower body), and bone mineral content. For this review, we will use consensus definitions for intra-articular knee injury, PA, the themes

104 underpinned by PA, and PTOA (Table 1). If there is no agreed or clear definition, then best practices
105 used by previous literature or consensus among the research team will be adopted.

Table 1. Review Definitions

Term	Definition
**Post-traumatic osteoarthritis**	Joint disorder characterised by extracellular matrix degradation initiated by micro- and macro-injury resulting in the manifestation of molecular derangement (e.g., abnormal joint tissue metabolism) followed by anatomic, and/or physiologic derangements (e.g., cartilage degradation), that can culminate in illness.[24]
**Intra-articular knee injury**	In accordance with previous research,[20 54 56 67 105] we will define intra-articular knee injury as any of the following knee injuries or reparative surgeries for the following tissues inside the articular cavity of the knee joint:<br>- ligament<br>- meniscus<br>- fracture<br>- dislocation<br>- contusion
**Physical activity**	Any bodily movement produced by skeletal muscles that results in energy expenditure.[106] Further, we will include the term 'exercise' within this definition of physical activity, as although they describe different concepts, they are often used as synonyms.[106] Exercise is defined as a subset of physical activity that is planned, structured, and repetitive and has as a final or an immediate objective the improvement or maintenance of physical fitness.[106]
**Systemic inflammation**	A consensus definition on the type, number, and value of inflammatory biomarkers for systemic inflammation diagnosis has not been devised. In this review, we will use concentrations of key circulating proteins and cytokines identified in previous research as being involved with inflammation processes and OA.[107-112]
**Knee joint load**	The research team defines knee joint load as quantification of the forces and/or moments applied to the knee joint specifically.
**Strength (lower body)**	Strength is the ability to produce a maximal voluntary muscular contraction against an external resistance.[113] We consider the following movements as relevant to this review:<br>- Hip extension/flexion/abduction/adduction<br>- Knee extension/flexion/abduction/adduction<br>- Dorsi/plantar flexion, supination/pronation
**Adipose tissue**	Specialised loose connective tissue that is extensively laden with adipocytes.[114] Adipose tissue is arranged in discrete depots,[115] and we consider the following depots at any body location relevent to this review:<br>- subcutaneous<br>- intraperitoneal/viseral<br>- inter-muscular (considered to be similar to intraperitoneal/visceral[116]
**Intra-muscular adipose tissue (lower body)**	Intra-muscular adipose tissue is the visible fat found within a muscle.[116] We consider the intra-muscular adipose content in any of the following muscle groups/muscles relevant to this review:<br>- Gluteal<br>- Hamstring<br>- Quadriceps<br>- Tibialis anterior<br>- Gastrocnemius<br>- Soleus

For peer review only - http://bmjopen.bmj.com/site/about/guidelines.xhtml

**Muscle size (lower body)**	Muscle size if the dimension of skeletal muscle, which can be devised into a multi-scale[117] consisting of;<br>- organ<br>- tissue<br>- cellular<br>- sub-cellular<br>- molecular<br><br>We consider the muscle size of muscle groups/muscles relevant to this review:<br>- Gluteal<br>- Hamstring<br>- Quadriceps<br>- Tibialis anterior<br>- Gastrocnemius<br>- Soleus
**Bone mineral content (lower body)**	The research team defines bone mineral content as the amount of minerals (mostly calcium and phosphorous) contained in bone. The mineral content of the following bones is considered relevant to this review:<br>- Femur<br>- Tibia<br>- Fibular

Stage 2: Identifying Relevant Studies

The search terms include population, independent variable, and outcome (Supplementary Material 2). Using the Peer Review of Electronic Search Strategy (PRESS)[118] guidelines, a professional health science librarian was consulted to develop and tailor search strategies for each database. Electronic databases Scopus, Embase, Medline, Web of Science: all databases, SPORTDiscus, Physiotherapy Evidence Database (PEDro), and Google Scholar will be searched. Literature published between 1970 and 2022 will be included, covering the period during which clinical knee injury diagnosis was formalised and surgical reparative techniques were popularised.[119 120] Primary research studies, meta-analyses, and grey literature will be included.[93 121 122]

Stage 3: Study Selection

Inclusion and exclusion criteria are presented in Table 2.

Table 2. Review Inclusion and Exclusion Criteria

**Inclusion**	**Exclusion**

For peer review only

Includes participants with a history of experiencing intra-articular injury or reparative surgery for the injury.	Reporting of any other medical condition (disease, illness, or disorder):<br>- Musculoskeletal<br>- Immunological<br>- Cardiological<br>- Respiratory<br>- Neurological<br>- Metabolic
Measures any association between PA or factors determined by PA (systemic markers of inflammation, joint loading, adiposity, muscle strength, lower body muscle size, inter-muscular fat, bone density), and either post-traumatic osteoarthritis diagnosis or indicators of joint degeneration.	Evidence of intra-articular injury incidence to any of the following joints prior to or following intra-articular knee injury:<br>- Ankle<br>- Knee<br>- Hip<br>- Shoulder<br>- Elbow
18-40 years of age	Revision surgery if undergone initial reparative surgery
Primary research studies, reviews, meta-analyses, and grey literature	Animal models, *in vitro* studies, purely *in silico* studies with no human component (i.e., simulated data with no data capture element working with humans)
Human research	
Published in English	
Literature published between 1970 and 2022	

For peer review only

Outcome measures for PTOA disease must include medical imaging or molecular indicators of joint health (e.g., metabolic, proteomic, genomic, transcriptomic).[24] Imaging diagnosis of disease may include radiography[123 124] or magnetic resonance imaging (MRI).[125-127] Imaging indicators for disease processes include MRI[128-131] and ultrasound (US) techniques[132-134]. For molecular indicators of disease processes, OARSI recommended biomarkers[135] will be included alongside other biomarkers of cartilage metabolism which have been deemed of interest in previous reviews due to their association with joint health (Supplementary Material 3).[136 137] Outcome measures for PTOA illness will include Luyten Criteria[138] or Englund Criteria[42] diagnosis or indicative signs of PTOA including knee specific and general measures of symptomology, function, and quality of life (Supplementary Material 3).[139-141] This list of outcome measures is not exhaustive and will be added to if other relevant outcomes are identified during Stage 2 - Identifying Relevant Studies.

For peer review only - http://bmjopen.bmj.com/site/about/guidelines.xhtml

To establish the face and content validity of the inclusion and exclusion criteria, and the selected outcome measures, feedback was sought from a surgeon, an osteopath, a physiotherapist, a physiologist, and a biomechanist independent from the research team, with experience ranging from clinical to sport research. These inclusion and exclusion criteria will be applied to title/abstract screening, which will be conducted using Covidence (Covidence Systematic Review Software, Veritas Health Innovation, Melbourne, Australia. Available at www.covidence.org). Prior to data screening, JC and MF will be familiarised and trained[142] by the lead author (KM) to conduct title/abstract screening using a screening tool hosted on Research Electronic Data Capture Platform (REDCap)[143] (Supplementary Material 4). Following the training session, inter-rater reliability of the inclusion and exclusion criteria for title/abstract screening will be assessed based on a random sample of 120 titles/abstracts. Percentage agreement and Cohen's Kappa (κ)[144] will be determined between all reviewers (KM - 100% of records, JC - 100%, MF - 100%). The inclusion and exclusion criteria will be deemed reliable if the agreement is 'strong' (percentage agreement >63% and κ >0.60),[145] and title/abstract screening will only begin once the agreement level is strong. Following training and provided 'strong' agreement between reviewers is achieved, title/abstract screening will begin. During this process reviewing pairs (KM - 100% of records, JC - 50%, MF - 50%) will independently screen all titles and abstracts to reduce quantity of errors.[146] If disagreements cannot be resolved within reviewing pairs, a consensus decision across the wider research team will be made following a discussion.

Following title and abstract screening, full text screening will be conducted by reviewing pairs (KM - 100% of records, JC - 50%, MF - 50%).[142] If disagreements cannot be resolved within reviewing pairs, a consensus decision across the research team will be made following a discussion. In line with common practice[147 148] and best-practice recommendations,[149] we will contact corresponding and co-authors via e-mail if details of a study are unclear during full text screening (Supplementary Material 5). A maximum of two attempted contacts will be made. The number of corresponding authors contacted, co-authors contacted, replies, and whether adequate information was provided, will be reported.

For peer review only - http://bmjopen.bmj.com/site/about/guidelines.xhtml

Quality and Critical Appraisal

In accordance with best-practice recommendations,[99] we will assess methodological quality before conducting data charting of included studies. As this review will include various study designs, we will employ JBI appraisal tools respective to each study design (Supplementary Material 6).[150-153] Individual items within each JBI appraisal tool will be assigned either a 'yes' (1), 'no' (0), or 'unclear' (seek clarification/further information) response to questions relating to the research quality. If study details are unclear, corresponding authors will be contacted following the same process as described in Stage 3 – Study Selection (Supplementary Material 5). A quality percentage score will be calculated, with smaller scores indicating lower quality and larger scores indicating higher quality. Identified studies will be included regardless of the quality appraisal score. The appraisals will be conducted by reviewing pairs (KM – 100%, JC – 50%, MF – 50%) using REDCap.[143]

Stage 4: Charting the Data

Data charting will be conducted in REDCap[143] by reviewing pairs (KM – 100%, JC – 50%, MF – 50%) to reduce the risk of errors in data extraction.[146] Data charting will include author(s), country of study, study year, study design, participant age and sex/gender distributions, injury type, surgery type, type of injury management, time since injury/surgery, sporting history before injury (type and level), aim of study, independent variables, supervision level (if intervention), quantity (if intervention), adherence rate (if intervention), drop out (if intervention), outcome variables, who assessed the measures, statistical methodology, and the strength of the treatment response or relationship between variables. This form (Supplementary Material 7) will be pilot tested by two reviewers and necessary amendments will be made if required (e.g., other relevant measurement tools or outcome variables). If study details are unclear, corresponding authors will be contacted following the same process as described in Stage 3 – Study Selection

Stage 5: Summarising the Data

For peer review only - http://bmjopen.bmj.com/site/about/guidelines.xhtml

Data will be summarised in three steps.[99] The first step will present the results as a descriptive numerical summary which will include: number of studies on each theme of the research question; frequency of study design; definitions and variables used for joint disease and illness; overview of methodological quality of studies. The second step will present the results as individual sections for each theme of the research question. Each theme will be structured as follows:

- Strength of the treatment response or relationship between variables[154-157] (Supplementary Material 8) for studies presented descriptively
- In accordance with a previous scoping review on ACL patients,[158] we will use the GRADE[89-91] (incorporates Bradford Hill Criteria[159-161]) for quality of evidence for either protective, degenerative, or unknown effect (Supplementary Material 9). GRADE will be conducted by the research panel (KM, JC, MF, DC, JPW, CM). Study-by-study results will be presented in supplementary material, whilst the overall summary of results for each theme will be displayed within the article in a summary of findings table

The third step will be to discuss and consider the meaning of the findings as they relate to the overall review purpose. We will also discuss implications for future PTOA research.

ETHICS AND DISSEMINATION

Ethical approval is not required as this review maps and synthesises data generated from published literature. This review will be submitted for publication in a peer-reviewed sports medicine journal, irrespective of findings which indicate a positive or negative association between PA and joint health. Knowledge synthesised by this review will be presented at scientific conferences and disseminated via social media platforms.

CONCLUSION

For peer review only - http://bmjopen.bmj.com/site/about/guidelines.xhtml

This study seeks to address a salient topic for people who experience a traumatic intra-articular knee injury and clinicians working with this population. Using a scoping review methodology to examine the known influence of PA on PTOA pathogenesis is novel and appropriate, considering the complexity of the topic and the multi-disciplinary evidence base. This research may inform future PTOA research by helping to guide research priorities and identifying outcome measures for clinical interventions.

CRediT (Contributor Roles Taxonomy)

Karl Morgan: Conceptualisation, Methodology, Writing - Original Draft, Writing - Review & Editing. **Dr James Cowburn:** Methodology, Writing - Review & Editing. **Dr Matthew Farrow:** Methodology, Writing - Review & Editing. **Dr Dario Cazzola:** Writing - Review & Editing, Supervision. **Dr Jean-Philippe Walhin:** Writing - Review & Editing, Supervision. **Dr Carly McKay:** Conceptualisation, Methodology, Writing - Review & Editing, Supervision.

Acknowledgements

We thank Peter Bradley, the University of Bath's Subject Librarian for Health and Social & Policy Sciences for his help in developing the search terms and his role when implementing the Peer Review of Electronic Search Strategy (PRESS)[118] guidelines. We also thank Dr David Bruce, Dr Nicos Haralabidis, Rosy Hyman, Charli Robertson, and Lee Page for their expert feedback developing the inclusion/exclusion criteria and outcome measures.

Funding statement

This work was funded by the University of Bath (funding refence: EA-FH1112) in affiliation with the Centre for Sport, Exercise and Osteoarthritis Research Versus Arthritis, University of Bath (grant reference: 21595).

For peer review only - http://bmjopen.bmj.com/site/about/guidelines.xhtml

For peer review only

Conflict of interest statement

None declared.

For peer review only

REFERENCES

1. Cochrane JL, Lloyd DG, Buttfield A, et al. Characteristics of anterior cruciate ligament injuries in Australian football. *J Sci Med Sport* 2007;10(2):96-104. doi: 10.1016/j.jsams.2006.05.015 [published Online First: 20060627]

2. Walden M, Krosshaug T, Bjorneboe J, et al. Three distinct mechanisms predominate in non-contact anterior cruciate ligament injuries in male professional football players: a systematic video analysis of 39 cases. *Br J Sports Med* 2015;49(22):1452-60. doi: 10.1136/bjsports-2014-094573 [published Online First: 2015/04/25]

3. Montgomery C, Blackburn J, Withers D, et al. Mechanisms of ACL injury in professional rugby union: a systematic video analysis of 36 cases. *Br J Sports Med* 2018;52(15):994-1001. doi: 10.1136/bjsports-2016-096425 [published Online First: 2017/01/01]

4. Boden BP, Dean GS, Feagin JA, et al. Mechanisms of anterior cruciate ligament injury. *Orthopedics* 2000;23(6):573-8.

5. Olsen OE, Myklebust G, Engebretsen L, et al. Injury mechanisms for anterior cruciate ligament injuries in team handball: a systematic video analysis. *Am J Sports Med* 2004;32(4):1002-12. doi: 10.1177/0363546503261724 [published Online First: 2004/05/20]

6. Koga H, Nakamae A, Shima Y, et al. Mechanisms for noncontact anterior cruciate ligament injuries: knee joint kinematics in 10 injury situations from female team handball and basketball. *Am J Sports Med* 2010;38(11):2218-25. doi: 10.1177/0363546510373570 [published Online First: 2010/07/03]

7. Ruedl G, Linortner I, Schranz A, et al. Distribution of injury mechanisms and related factors in ACL-injured female carving skiers. *Knee Surg Sports Traumatol Arthrosc* 2009;17(11):1393-8. doi: 10.1007/s00167-009-0860-7 [published Online First: 2009/07/11]

8. Ruedl G, Webhofer M, Linortner I, et al. ACL injury mechanisms and related factors in male and female carving skiers: a retrospective study. *Int J Sports Med* 2011;32(10):801-6. doi: 10.1055/s-0031-1279719 [published Online First: 2011/06/23]

9. Gabr A, De Medici A, Haddad F. The National Ligament Registry 2019 Annual Report. In: Spalding T, ed. The National Ligament Registry, 2020:1-14.

10. Moses B, Orchard J, Orchard J. Systematic review: Annual incidence of ACL injury and surgery in various populations. *Res Sports Med* 2012;20(3-4):157-79. doi: 10.1080/15438627.2012.680633 [published Online First: 2012/06/30]

11. Noyes FR, Barber-Westin SD. Treatment of meniscus tears during anterior cruciate ligament reconstruction. *Arthroscopy* 2012;28(1):123-30. doi: 10.1016/j.arthro.2011.08.292 [published Online First: 2011/11/15]

12. Ghodadra N, Mall NA, Karas V, et al. Articular and meniscal pathology associated with primary anterior cruciate ligament reconstruction. *J Knee Surg* 2013;26(3):185-93. doi: 10.1055/s-0032-1327450 [published Online First: 2013/01/05]

For peer review only - http://bmjopen.bmj.com/site/about/guidelines.xhtml

13. Mall NA, Chalmers PN, Moric M, et al. Incidence and trends of anterior cruciate ligament reconstruction in the United States. *Am J Sports Med* 2014;42(10):2363-70. doi: 10.1177/0363546514542796 [published Online First: 2014/08/03]

14. Everhart JS, DiBartola AC, Swank K, et al. Cartilage damage at the time of anterior cruciate ligament reconstruction is associated with weaker quadriceps function and lower risk of future ACL injury. *Knee Surg Sports Traumatol Arthrosc* 2020;28(2):576-83. doi: 10.1007/s00167-019-05739-1 [published Online First: 2019/10/11]

15. Duthon VB, Barea C, Abrassart S, et al. Anatomy of the anterior cruciate ligament. *Knee Surg Sports Traumatol Arthrosc* 2006;14(3):204-13. doi: 10.1007/s00167-005-0679-9 [published Online First: 2005/10/20]

16. Lohmander LS, Englund PM, Dahl LL, et al. The long-term consequence of anterior cruciate ligament and meniscus injuries: osteoarthritis. *Am J Sports Med* 2007;35(10):1756-69. doi: 10.1177/0363546507307396 [published Online First: 2007/09/01]

17. Abram SGF, Price AJ, Judge A, et al. Anterior cruciate ligament (ACL) reconstruction and meniscal repair rates have both increased in the past 20 years in England: hospital statistics from 1997 to 2017. *Br J Sports Med* 2020;54(5):286-91. doi: 10.1136/bjsports-2018-100195 [published Online First: 2019/01/21]

18. Domnick C, Garcia P, Raschke MJ, et al. Trends and incidences of ligament-surgeries and osteotomies of the knee: an analysis of German inpatient records 2005-2013. *Arch Orthop Trauma Surg* 2017;137(7):989-95. doi: 10.1007/s00402-017-2704-0 [published Online First: 2017/05/04]

19. Zbrojkiewicz D, Vertullo C, Grayson JE. Increasing rates of anterior cruciate ligament reconstruction in young Australians, 2000-2015. *Med J Aust* 2018;208(8):354-58. doi: doi:10.5694/mja17.00974 [published Online First: 2018/04/20]

20. Snoeker B, Turkiewicz A, Magnusson K, et al. Risk of knee osteoarthritis after different types of knee injuries in young adults: a population-based cohort study. *Br J Sports Med* 2020;54(12):725-30. doi: 10.1136/bjsports-2019-100959 [published Online First: 2019/12/13]

21. Morgan OJ, Hillstrom HJ, Ellis SJ, et al. Osteoarthritis in England: incidence trends from National Health Service Hospital Episode Statistics. *ACR Open Rheumatol* 2019;1(8):493-98. doi: 10.1002/acr2.11071 [published Online First: 2019/11/30]

22. Wallace IJ, Worthington S, Felson DT, et al. Knee osteoarthritis has doubled in prevalence since the mid-20th century. *Proc Natl Acad Sci U S A* 2017;114(35):9332-36. doi: 10.1073/pnas.1703856114 [published Online First: 2017/08/16]

23. Kiadaliri AA, Rinaldi G, Lohmander LS, et al. Temporal trend and regional disparity in osteoarthritis hospitalisations in Sweden 1998–2015. *Scand J Public Health* 2019;47(1):53-60. doi: 10.1177/1403494818766785

24. Kraus VB, Blanco FJ, Englund M, et al. Call for standardized definitions of osteoarthritis and risk stratification for clinical trials and clinical use. *Osteoarthritis Cartilage* 2015;23(8):1233-41. doi: 10.1016/j.joca.2015.03.036 [published Online First: 2015/04/14]

For peer review only

25. Illingworth KD, El Bitar Y, Siewert K, et al. Correlation of WOMAC and KOOS scores to tibiofemoral cartilage loss on plain radiography and 3 Tesla MRI: data from the osteoarthritis initiative. *Knee Surg Sports Traumatol Arthrosc* 2014;22(7):1649-58.

26. Bedson J, Croft PR. The discordance between clinical and radiographic knee osteoarthritis: a systematic search and summary of the literature. *BMC Musculoskelet Disord* 2008;9:116. doi: 10.1186/1471-2474-9-116 [published Online First: 2008/09/04]

27. Whittaker JL, Toomey CM, Woodhouse LJ, et al. Association between MRI-defined osteoarthritis, pain, function and strength 3-10 years following knee joint injury in youth sport. *Br J Sports Med* 2018;52(14):934-39. doi: 10.1136/bjsports-2017-097576 [published Online First: 2017/10/12]

28. Friel NA, Chu CR. The role of ACL injury in the development of posttraumatic knee osteoarthritis. *Clin Sports Med* 2013;32(1):1-12. doi: 10.1016/j.csm.2012.08.017 [published Online First: 2012/11/28]

29. Luc B, Gribble PA, Pietrosimone BG. Osteoarthritis prevalence following anterior cruciate ligament reconstruction: a systematic review and numbers-needed-to-treat analysis. *J Athl Train* 2014;49(6):806-19. doi: 10.4085/1062-6050-49.3.35 [published Online First: 2014/09/19]

30. Gelber AC, Hochberg MC, Mead LA, et al. Joint injury in young adults and risk for subsequent knee and hip osteoarthritis. *Ann Intern Med* 2000;133(5):321-8. doi: 10.7326/0003-4819-133-5-200009050-00007 [published Online First: 2000/09/09]

31. Roos EM. Joint injury causes knee osteoarthritis in young adults. *Curr Opin Rheumatol* 2005;17(2):195-200. doi: 10.1097/01.bor.0000151406.64393.00 [published Online First: 2005/02/16]

32. Chen T, Wang S, Li Y, et al. Radiographic osteoarthritis prevalence over ten years after anterior cruciate ligament reconstruction. *Int J Sports Med* 2019;40(11):683-95. doi: 10.1055/a-0902-8539 [published Online First: 2019/09/19]

33. Losina E, Weinstein AM, Reichmann WM, et al. Lifetime risk and age at diagnosis of symptomatic knee osteoarthritis in the US. *Arthritis Care Res (Hoboken)* 2013;65(5):703-11. doi: 10.1002/acr.21898 [published Online First: 2012/12/04]

34. Khan T, Alvand A, Prieto-Alhambra D, et al. ACL and meniscal injuries increase the risk of primary total knee replacement for osteoarthritis: a matched case-control study using the Clinical Practice Research Datalink (CPRD). *Br J Sports Med* 2019;53(15):965-68. doi: 10.1136/bjsports-2017-097762 [published Online First: 2018/01/15]

35. Price AJ, Alvand A, Troelsen A, et al. Knee replacement. *Lancet* 2018;392(10158):1672-82. doi: 10.1016/s0140-6736(18)32344-4

36. W-Dahl A, Robertsson O, Lidgren L. Surgery for knee osteoarthritis in younger patients. *Acta Orthop* 2010;81(2):161-64. doi: 10.3109/17453670903413186

37. Evans JT, Walker RW, Evans JP, et al. How long does a knee replacement last? A systematic review and meta-analysis of case series and national registry reports with more than 15 years of follow-

For peer review only - http://bmjopen.bmj.com/site/about/guidelines.xhtml

up. *Lancet* 2019;393(10172):655-63. doi: 10.1016/S0140-6736(18)32531-5 [published Online First: 2019/02/21]

38. Bayliss LE, Culliford D, Monk AP, et al. The effect of patient age at intervention on risk of implant revision after total replacement of the hip or knee: a population-based cohort study. *Lancet* 2017;389(10077):1424-30. doi: 10.1016/s0140-6736(17)30059-4

39. Wasserstein D, Huston LJ, Nwosu S, et al. KOOS pain as a marker for significant knee pain two and six years after primary ACL reconstruction: a Multicenter Orthopaedic Outcomes Network (MOON) prospective longitudinal cohort study. *Osteoarthritis Cartilage* 2015;23(10):1674-84. doi: 10.1016/j.joca.2015.05.025 [published Online First: 2015/06/15]

40. Antosh IJ, Svoboda SJ, Peck KY, et al. Change in KOOS and WOMAC scores in a young athletic population with and without anterior cruciate ligament injury. *Am J Sports Med* 2018;46(7):1606-16. doi: 10.1177/0363546518768753 [published Online First: 2018/05/08]

41. Whittaker JL, Woodhouse LJ, Nettel-Aguirre A, et al. Outcomes associated with early post-traumatic osteoarthritis and other negative health consequences 3-10 years following knee joint injury in youth sport. *Osteoarthritis Cartilage* 2015;23(7):1122-9. doi: 10.1016/j.joca.2015.02.021 [published Online First: 2015/03/01]

42. Englund M, Roos EM, Lohmander LS. Impact of type of meniscal tear on radiographic and symptomatic knee osteoarthritis: a sixteen-year followup of meniscectomy with matched controls. *Arthritis Rheum* 2003;48(8):2178-87. doi: 10.1002/art.11088 [published Online First: 2003/08/09]

43. Harkey MS, Baez S, Lewis J, et al. Prevalence of early knee osteoarthritis illness among various patient-reported classification criteria after anterior cruciate ligament reconstruction. *Arthritis Care Res (Hoboken)* 2022;74(3):377-85. doi: 10.1002/acr.24809

44. Arden N, Nevitt MC. Osteoarthritis: epidemiology. *Best Pract Res Clin Rheumatol* 2006;20(1):3-25. doi: 10.1016/j.berh.2005.09.007 [published Online First: 2006/02/18]

45. Rathleff MS, Roos EM, Olesen JL, et al. High prevalence of daily and multi-site pain--a cross-sectional population-based study among 3000 Danish adolescents. *BMC Pediatr* 2013;13:191. doi: 10.1186/1471-2431-13-191 [published Online First: 2013/11/21]

46. Peat G. Knee pain and osteoarthritis in older adults: a review of community burden and current use of primary health care. *Ann Rheum Dis* 2001;60(2):91-97. doi: 10.1136/ard.60.2.91

47. Turkiewicz A, Gerhardsson De Verdier M, Engstrom G, et al. Prevalence of knee pain and knee OA in southern Sweden and the proportion that seeks medical care. *Rheumatology (Oxford)* 2015;54(5):827-35. doi: 10.1093/rheumatology/keu409

48. Holla JF, Sanchez-Ramirez DC, van der Leeden M, et al. The avoidance model in knee and hip osteoarthritis: a systematic review of the evidence. *J Behav Med* 2014;37(6):1226-41. doi: 10.1007/s10865-014-9571-8 [published Online First: 2014/05/21]

For peer review only

49. Shortland A. Muscle deficits in cerebral palsy and early loss of mobility: can we learn something from our elders? *Dev Med Child Neurol* 2009;51:59-63. doi: 10.1111/j.1469-8749.2009.03434.x

50. Osteoarthritis Research Society International. Osteoarthritis: A Serious Disease: Osteoarthritis Research Society International, 2016.

51. Whittaker JL, Roos EM. A pragmatic approach to prevent post-traumatic osteoarthritis after sport or exercise-related joint injury. *Best Pract Res Clin Rheumatol* 2019;33(1):158-71. doi: 10.1016/j.berh.2019.02.008 [published Online First: 2019/08/23]

52. Kvist J, Ek A, Sporrstedt K, et al. Fear of re-injury: a hindrance for returning to sports after anterior cruciate ligament reconstruction. *Knee Surg Sports Traumatol Arthrosc* 2005;13(5):393-7. doi: 10.1007/s00167-004-0591-8 [published Online First: 2005/02/11]

53. Ardern CL, Osterberg A, Sonesson S, et al. Satisfaction with knee function after primary anterior cruciate ligament reconstruction is associated with self-efficacy, quality of life, and returning to the preinjury physical activity. *Arthroscopy* 2016;32(8):1631-38. doi: 10.1016/j.arthro.2016.01.035 [published Online First: 2016/03/30]

54. Toomey CM, Whittaker JL, Nettel-Aguirre A, et al. Higher fat mass is associated with a history of knee injury in youth sport. *J Orthop Sports Phys Ther* 2017;47(2):80-87. doi: 10.2519/jospt.2017.7101 [published Online First: 2017/02/02]

55. Bell DR, Pfeiffer KA, Cadmus-Bertram LA, et al. Objectively measured physical activity in patients after anterior cruciate ligament reconstruction. *Am J Sports Med* 2017;45(8):1893-900. doi: 10.1177/0363546517698940 [published Online First: 2017/04/19]

56. Toomey C, Whittaker JL, Doyle-Baker PK, et al. The association between physical activity and 3–15 year history of sport-related intra-articular knee injury: a matched cohort design [Abstract]. *Br J Sports Med* 2018; 52. https://bjsm.bmj.com/content/52/Suppl_1/A6.1 (accessed 01/04/2021).

57. Kuenze C, Cadmus-Bertram L, Pfieffer K, et al. Relationship between physical activity and clinical outcomes after ACL reconstruction. *J Sport Rehabil* 2019;28(2):180-87. doi: 10.1123/jsr.2017-0186 [published Online First: 2017/11/16]

58. Kuenze C, Lisee C, Pfeiffer KA, et al. Sex differences in physical activity engagement after ACL reconstruction. *Phys Ther Sport* 2019;35:12-17. doi: 10.1016/j.ptsp.2018.10.016 [published Online First: 2018/11/06]

59. Triplett AN, Kuenze CM. Characterizing body composition, cardiorespiratory fitness, and physical activity in women with anterior cruciate ligament reconstruction. *Phys Ther Sport* 2020;48:54-59. doi: 10.1016/j.ptsp.2020.12.014 [published Online First: 2020/12/30]

60. Baez SE, Hoch MC, Hoch JM. Psychological factors are associated with return to pre-injury levels of sport and physical activity after ACL reconstruction. *Knee Surg Sports Traumatol Arthrosc* 2020;28(2):495-501. doi: 10.1007/s00167-019-05696-9 [published Online First: 2019/09/06]

For peer review only - http://bmjopen.bmj.com/site/about/guidelines.xhtml

61. Webster KE, Feller JA. Expectations for return to preinjury sport before and after anterior cruciate ligament reconstruction. *Am J Sports Med* 2019;47(3):578-83. doi: 10.1177/0363546518819454 [published Online First: 2019/01/17]

62. Ardern CL, Webster KE, Taylor NF, et al. Return to sport following anterior cruciate ligament reconstruction surgery: a systematic review and meta-analysis of the state of play. *Br J Sports Med* 2011;45(7):596-606. doi: 10.1136/bjsm.2010.076364 [published Online First: 2011/03/15]

63. Ardern CL, Taylor NF, Feller JA, et al. Fifty-five per cent return to competitive sport following anterior cruciate ligament reconstruction surgery: an updated systematic review and meta-analysis including aspects of physical functioning and contextual factors. *Br J Sports Med* 2014;48(21):1543-52. doi: 10.1136/bjsports-2013-093398

64. National Institute for Health and Care Excellence. Osteoarthritis: care and management (update): National Institute for Health and Care Excellence; 2022 [Available from: https://www.nice.org.uk/guidance/indevelopment/gid-ng10127/consultation/html-content-2 accessed 31/05/2022 2022.

65. MacAlpine EM, Talwar D, Storey EP, et al. Weight gain after ACL reconstruction in pediatric and adolescent patients. *Sports Health* 2020;12(1):29-35. doi: 10.1177/1941738119870192 [published Online First: 2019/09/06]

66. Patterson BE, Culvenor AG, Barton CJ, et al. Worsening knee osteoarthritis features on magnetic resonance imaging 1 to 5 years after anterior cruciate ligament reconstruction. *Am J Sports Med* 2018;46(12):2873-83. doi: 10.1177/0363546518789685 [published Online First: 2018/09/05]

67. Ezzat AM, Whittaker JL, Toomey C, et al. Knee confidence in youth and young adults at risk of post-traumatic osteoarthritis 3-10 years following intra-articular knee injury. *J Sci Med Sport* 2018;21(7):671-75. doi: 10.1016/j.jsams.2017.11.012 [published Online First: 2017/12/14]

68. Tayfur B, Charuphongsa C, Morrissey D, et al. Neuromuscular function of the knee joint following knee injuries: does it ever get back to normal? A systematic review with meta-analyses. *Sports Med* 2021;51(2):321-38. doi: 10.1007/s40279-020-01386-6 [published Online First: 2020/11/29]

69. Hart JM, Pietrosimone B, Hertel J, et al. Quadriceps activation following knee injuries: a systematic review. *J Athl Train* 2010;45(1):87-97. doi: 10.4085/1062-6050-45.1.87 [published Online First: 2010/01/13]

70. Lisee C, Lepley AS, Birchmeier T, et al. Quadriceps strength and volitional activation after anterior cruciate ligament reconstruction: a systematic review and meta-analysis. *Sports Health* 2019;11(2):163-79. doi: 10.1177/1941738118822739 [published Online First: 2019/01/15]

71. Birchmeier T, Lisee C, Kane K, et al. Quadriceps muscle size following ACL injury and reconstruction: a systematic review. *J Orthop Res* 2020;38(3):598-608. doi: 10.1002/jor.24489 [published Online First: 2019/10/15]

For peer review only

72. Dutaillis B, Maniar N, Opar DA, et al. Lower limb muscle size after anterior cruciate ligament injury: a systematic review and meta-analysis. *Sports Med* 2021; in press. https://www.ncbi.nlm.nih.gov/pubmed/33492623

https://link.springer.com/content/pdf/10.1007/s40279-020-01419-0.pdf (accessed 15/03/2021).

73. Lindstrom M, Strandberg S, Wredmark T, et al. Functional and muscle morphometric effects of ACL reconstruction. A prospective CT study with 1 year follow-up. *Scand J Med Sci Sports* 2013;23(4):431-42. doi: 10.1111/j.1600-0838.2011.01417.x [published Online First: 2011/11/24]

74. Lopresti C, Kirkendall DT, Street GM, et al. Quadriceps insufficiency following repair of the anterior cruciate ligament. *J Orthop Sports Phys Ther* 1988;9(7):245-49.

75. Van Meer BL, Waarsing JH, Van Eijsden WA, et al. Bone mineral density changes in the knee following anterior cruciate ligament rupture. *Osteoarthritis Cartilage* 2014;22(1):154-61. doi: 10.1016/j.joca.2013.11.005

76. Fischer CP, Berntsen A, Perstrup LB, et al. Plasma levels of interleukin-6 and C-reactive protein are associated with physical inactivity independent of obesity. *Scand J Med Sci Sports* 2007;17(5):580-7. doi: 10.1111/j.1600-0838.2006.00602.x [published Online First: 2006/11/02]

77. Hamer M. The relative influences of fitness and fatness on inflammatory factors. *Prev Med* 2007;44(1):3-11. doi: 10.1016/j.ypmed.2006.09.005 [published Online First: 2006/10/27]

78. Lavin KM, Perkins RK, Jemiolo B, et al. Effects of aging and lifelong aerobic exercise on basal and exercise-induced inflammation. *J Appl Physiol (1985)* 2020;128(1):87-99. doi: 10.1152/japplphysiol.00495.2019 [published Online First: 2019/11/22]

79. Myers A, Gibbons C, Finlayson G, et al. Associations among sedentary and active behaviours, body fat and appetite dysregulation: investigating the myth of physical inactivity and obesity. *Br J Sports Med* 2017;51(21):1540-44. doi: 10.1136/bjsports-2015-095640 [published Online First: 2016/04/06]

80. Krogh-Madsen R, Thyfault JP, Broholm C, et al. A 2-wk reduction of ambulatory activity attenuates peripheral insulin sensitivity. *J Appl Physiol (1985)* 2010;108(5):1034-40. doi: 10.1152/japplphysiol.00977.2009 [published Online First: 2010/01/02]

81. Manini TM, Clark BC, Nalls MA, et al. Reduced physical activity increases intermuscular adipose tissue in healthy young adults. *Am J Clin Nutr* 2007;85(2):377-84. doi: 10.1093/ajcn/85.2.377 [published Online First: 2007/02/08]

82. Farr JN, Van Loan MD, Lohman TG, et al. Lower physical activity is associated with skeletal muscle fat content in girls. *Med Sci Sports Exerc* 2012;44(7):1375-81. doi: 10.1249/MSS.0b013e31824749b2 [published Online First: 2012/01/06]

83. Dimmick HL, Miller JD, Sterczala AJ, et al. Vastus lateralis muscle tissue composition and motor unit properties in chronically endurance-trained vs. sedentary women. *Eur J Appl Physiol* 2018;118(9):1789-800. doi: 10.1007/s00421-018-3909-9 [published Online First: 2018/06/28]

For peer review only - http://bmjopen.bmj.com/site/about/guidelines.xhtml

84. Pagano AF, Brioche T, Arc-Chagnaud C, et al. Short-term disuse promotes fatty acid infiltration into skeletal muscle. *J Cachexia Sarcopenia Muscle* 2018;9(2):335-47. doi: 10.1002/jcsm.12259 [published Online First: 2017/12/17]

85. MacLennan RJ, Sahebi M, Becker N, et al. Declines in skeletal muscle quality vs. size following two weeks of knee joint immobilization. *PeerJ* 2020; 8. https://www.ncbi.nlm.nih.gov/pmc/articles/PMC6964688/

https://www.ncbi.nlm.nih.gov/pmc/articles/PMC6964688/pdf/peerj-08-8224.pdf (accessed 18/04/2021).

86. Tuttle LJ, Sinacore DR, Cade WT, et al. Lower physical activity is associated with higher intermuscular adipose tissue in people with type 2 diabetes and peripheral neuropathy. *Phys Ther* 2011;91(6):923-30. doi: 10.2522/ptj.20100329 [published Online First: 2011/04/09]

87. Chastin SF, Mandrichenko O, Helbostadt J, et al. Associations between objectively-measured sedentary behaviour and physical activity with bone mineral density in adults and older adults, the NHANES study. *Bone* 2014;64:254-62.

88. Nordström A, Olsson T, Nordström P. Sustained Benefits from Previous Physical Activity on Bone Mineral Density in Males. *J Clin Endocrinol Metab* 2006;91(7):2600-04. doi: 10.1210/jc.2006-0151

89. Guyatt GH, Oxman AD, Kunz R, et al. What is “quality of evidence” and why is it important to clinicians? *BMJ* 2008;336(7651):995-98. doi: 10.1136/bmj.39490.551019.be

90. Guyatt GH, Oxman AD, Vist GE, et al. GRADE: an emerging consensus on rating quality of evidence and strength of recommendations. *BMJ* 2008;336(7650):924-26. doi: 10.1136/bmj.39489.470347.ad

91. Guyatt G, Oxman AD, Akl EA, et al. GRADE guidelines: 1. Introduction-GRADE evidence profiles and summary of findings tables. *J Clin Epidemiol* 2011;64(4):383-94. doi: 10.1016/j.jclinepi.2010.04.026 [published Online First: 20101231]

92. Grimshaw J. A Guide to Knowledge Synthesis: A Knowledge Synthesis Chapter: Canadian Institutes of Health Research, 2020.

93. Peters MDJ, Marnie C, Tricco AC, et al. Updated methodological guidance for the conduct of scoping reviews. *JBI Evid Synth* 2020;18(10):2119-26. doi: 10.11124/jbies-20-00167

94. Khalil H, Peters MD, Tricco AC, et al. Conducting high quality scoping reviews-challenges and solutions. *J Clin Epidemiol* 2021;130:156-60. doi: 10.1016/j.jclinepi.2020.10.009

95. Peters MDJ, Godfrey C, McInerney P, et al. Best practice guidance and reporting items for the development of scoping review protocols. *JBI Evid Synth* 2022;20(4)

96. Shamseer L, Moher D, Clarke M, et al. Preferred reporting items for systematic review and meta-analysis protocols (PRISMA-P) 2015: elaboration and explanation. *BMJ* 2015;349(jan02 1):g7647-g47. doi: 10.1136/bmj.g7647

For peer review only - http://bmjopen.bmj.com/site/about/guidelines.xhtml

97. Moher D, Shamseer L, Clarke M, et al. Preferred reporting items for systematic review and meta-analysis protocols (PRISMA-P) 2015 statement. *Syst Rev* 2015;4(1):1. doi: 10.1186/2046-4053-4-1

98. Tricco AC, Lillie E, Zarin W, et al. PRISMA Extension for Scoping Reviews (PRISMA-ScR): checklist and explanation. *Ann Intern Med* 2018;169(7):467-73. doi: 10.7326/m18-0850

99. Levac D, Colquhoun H, O'Brien KK. Scoping studies: advancing the methodology. *Implement Sci* 2010;5(1):69. doi: 10.1186/1748-5908-5-69

100. Pham MT, Rajić A, Greig JD, et al. A scoping review of scoping reviews: advancing the approach and enhancing the consistency. *Res Synth Methods* 2014;5(4):371-85. doi: 10.1002/jrsm.1123

101. Munn Z, Peters MDJ, Stern C, et al. Systematic review or scoping review? Guidance for authors when choosing between a systematic or scoping review approach. *BMC Med Res Methodol* 2018;18(1):143. doi: 10.1186/s12874-018-0611-x [published Online First: 2018/11/21]

102. Watkins R, Young G, Western M, et al. Nobody says to you "come back in six months and we'll see how you're doing": a qualitative interview study exploring young adults' experiences of sport-related knee injury. *BMC Musculoskelet Disord* 2020;21(1) doi: 10.1186/s12891-020-03428-6

103. Arksey H, O'Malley L. Scoping studies: towards a methodological framework. *Int J Soc Res Methodol* 2005;8(1):19-32. doi: 10.1080/1364557032000119616

104. Helman CG. Disease versus illness in general practice. *J R Coll Gen Pract* 1981;31(230):548-52.

105. Toomey CM, Whittaker JL, Ghali B, et al. Youth and young adults have increased abdominal and total fat mass 3-10 years following intra-articular knee injury. *Osteoarthritis Cartilage* 2015;23:A188-A88. doi: DOI 10.1016/j.joca.2015.02.966

106. Caspersen CJ, Powell KE, Christenson GM. Physical activity, exercise, and physical fitness: definitions and distinctions for health-related research. *Public Health Rep* 1985;100(2):126-31. [published Online First: 1985/03/01]

107. Bricca A, Struglics A, Larsson S, et al. Impact of exercise therapy on molecular biomarkers related to cartilage and inflammation in individuals at risk of, or with established, knee osteoarthritis: a systematic review and meta-analysis of randomized controlled trials. *Arthritis Care Res (Hoboken)* 2019;71(11):1504-15. doi: 10.1002/acr.23786

108. Stannus O, Jones G, Blizzard L, et al. Associations between serum levels of inflammatory markers and change in knee pain over 5 years in older adults: a prospective cohort study. *Ann Rheum Dis* 2013;72(4):535-40. doi: 10.1136/annrheumdis-2011-201047

109. Stannus O, Jones G, Cicuttini F, et al. Circulating levels of IL-6 and TNF-alpha are associated with knee radiographic osteoarthritis and knee cartilage loss in older adults. *Osteoarthritis Cartilage* 2010;18(11):1441-7. doi: 10.1016/j.joca.2010.08.016 [published Online First: 2010/09/08]

For peer review only

110. Ruan G, Xu J, Wang K, et al. Associations between knee structural measures, circulating inflammatory factors and MMP13 in patients with knee osteoarthritis. *Osteoarthritis Cartilage* 2018;26(8):1063-69. doi: 10.1016/j.joca.2018.05.003 [published Online First: 2018/05/14]

111. Kobayashi M, Squires GR, Mousa A, et al. Role of interleukin-1 and tumor necrosis factor α in matrix degradation of human osteoarthritic cartilage. *Arthritis Rheum* 2005;52(1):128-35. doi: 10.1002/art.20776

112. Wojdasiewicz P, Poniatowski LA, Szukiewicz D. The role of inflammatory and anti-inflammatory cytokines in the pathogenesis of osteoarthritis. *Mediators Inflamm* 2014; 2014. https://www.hindawi.com/journals/mi/2014/561459/

https://downloads.hindawi.com/journals/mi/2014/561459.pdf (accessed 03/02/2021).

113. Williams TD, Tolusso DV, Fedewa MV, et al. Comparison of Periodized and Non-Periodized Resistance Training on Maximal Strength: A Meta-Analysis. *Sports Med* 2017;47(10):2083-100. doi: 10.1007/s40279-017-0734-y

114. Shen W, Wang Z, Punyanita M, et al. Adipose tissue quantification by imaging methods: a proposed classification. *Obes Res* 2003;11(1):5-16. doi: 10.1038/oby.2003.3 [published Online First: 2003/01/17]

115. Thompson D, Karpe F, Lafontan M, et al. Physical Activity and Exercise in the Regulation of Human Adipose Tissue Physiology. *Physiol Rev* 2012;92(1):157-91. doi: 10.1152/physrev.00012.2011

116. Addison O, Marcus RL, Lastayo PC, et al. Intermuscular fat: a review of the consequences and causes. *Int J Endocrinol* 2014;2014:309570. doi: 10.1155/2014/309570 [published Online First: 2014/02/15]

117. Wisdom KM, Delp SL, Kuhl E. Use it or lose it: multiscale skeletal muscle adaptation to mechanical stimuli. *Biomech Model Mechanobiol* 2015;14(2):195-215. doi: 10.1007/s10237-014-0607-3 [published Online First: 2014/09/10]

118. McGowan J, Sampson M, Salzwedel DM, et al. PRESS Peer Review of Electronic Search Strategies: 2015 Guideline Statement. *J Clin Epidemiol* 2016;75:40-46. doi: 10.1016/j.jclinepi.2016.01.021

119. Chambat P, Guier C, Sonnery-Cottet B, et al. The evolution of ACL reconstruction over the last fifty years. *Int Orthop* 2013;37(2):181-86. doi: 10.1007/s00264-012-1759-3

120. Di Matteo B, Moran CJ, Tarabella V, et al. A history of meniscal surgery: from ancient times to the twenty-first century. *Knee Surg Sports Traumatol Arthrosc* 2016;24(5):1510-18. doi: 10.1007/s00167-015-3717-2

121. Peters MD, Godfrey CM, Khalil H, et al. Guidance for conducting systematic scoping reviews. *Int J Evid Based Healthc* 2015;13(3):141-46.

122. Paez A. Grey literature: An important resource in systematic reviews. *J Evid Based Med* 2017 doi: 10.1111/jebm.12265

For peer review only - http://bmjopen.bmj.com/site/about/guidelines.xhtml

123. Kellgren JH, Lawrence JS. Radiological Assessment of Osteo-Arthrosis. *Ann Rheum Dis* 1957;16(4):494-502. doi: 10.1136/ard.16.4.494

124. Altman RD, Gold GE. Atlas of individual radiographic features in osteoarthritis, revised. *Osteoarthritis Cartilage* 2007;15:A1-A56. doi: 10.1016/j.joca.2006.11.009

125. Hunter DJ, Guermazi A, Lo GH, et al. Evolution of semi-quantitative whole joint assessment of knee OA: MOAKS (MRI Osteoarthritis Knee Score). *Osteoarthritis Cartilage* 2011;19(8):990-1002. doi: 10.1016/j.joca.2011.05.004 [published Online First: 2011/06/08]

126. Peterfy CG, Guermazi A, Zaim S, et al. Whole-Organ Magnetic Resonance Imaging Score (WORMS) of the knee in osteoarthritis. *Osteoarthritis Cartilage* 2004;12(3):177-90. doi: 10.1016/j.joca.2003.11.003

127. Hunter DJ, Lo GH, Gale D, et al. The reliability of a new scoring system for knee osteoarthritis MRI and the validity of bone marrow lesion assessment: BLOKS (Boston Leeds Osteoarthritis Knee Score). *Ann Rheum Dis* 2008;67(2):206-11. doi: 10.1136/ard.2006.066183 [published Online First: 2007/05/03]

128. Eckstein F, Cicuttini F, Raynauld JP, et al. Magnetic resonance imaging (MRI) of articular cartilage in knee osteoarthritis (OA): morphological assessment. *Osteoarthritis Cartilage* 2006;14:46-75. doi: 10.1016/j.joca.2006.02.026

129. Dunn TC, Lu Y, Jin H, et al. T2 relaxation time of cartilage at MR imaging: comparison with severity of knee osteoarthritis. *Radiology* 2004;232(2):592-8. doi: 10.1148/radiol.2322030976 [published Online First: 20040623]

130. Burstein D, Velyvis J, Scott KT, et al. Protocol issues for delayed Gd(DTPA)2--enhanced MRI (dGEMRIC) for clinical evaluation of articular cartilage. *Magn Reson Med* 2001;45(1):36-41. doi: 10.1002/1522-2594(200101)45:1<36::aid-mrm1006>3.0.co;2-w

131. Bashir A, Gray ML, Boutin RD, et al. Glycosaminoglycan in articular cartilage: in vivo assessment with delayed Gd(DTPA)(2-)-enhanced MR imaging. *Radiology* 1997;205(2):551-8. doi: 10.1148/radiology.205.2.9356644

132. Moller I, Bong D, Naredo E, et al. Ultrasound in the study and monitoring of osteoarthritis. *Osteoarthritis Cartilage* 2008;16:S4-7. doi: 10.1016/j.joca.2008.06.005 [published Online First: 2008/09/02]

133. Naredo E, Acebes C, Moller I, et al. Ultrasound validity in the measurement of knee cartilage thickness. *Ann Rheum Dis* 2009;68(8):1322-7. doi: 10.1136/ard.2008.090738 [published Online First: 2008/08/08]

134. Schmitz RJ, Wang HM, Polprasert DR, et al. Evaluation of knee cartilage thickness: A comparison between ultrasound and magnetic resonance imaging methods. *The Knee* 2017;24(2):217-23. doi: 10.1016/j.knee.2016.10.004 [published Online First: 2016/12/05]

135. Kraus VB, Collins JE, Hargrove D, et al. Predictive validity of biochemical biomarkers in knee osteoarthritis: data from the FNIH OA Biomarkers Consortium. *Ann Rheum Dis*

For peer review only

For peer review only - http://bmjopen.bmj.com/site/about/guidelines.xhtml

2017;76(1):186-95. doi: 10.1136/annrheumdis-2016-209252 [published Online First: 2016/06/15]

136. Roberts HM, Law RJ, Thom JM. The time course and mechanisms of change in biomarkers of joint metabolism in response to acute exercise and chronic training in physiologic and pathological conditions. *Eur J Appl Physiol* 2019;119(11-12):2401-20. doi: 10.1007/s00421-019-04232-4 [published Online First: 2019/10/28]

137. Bay-Jensen AC, Mobasheri A, Thudium CS, et al. Blood and urine biomarkers in osteoarthritis–an update on cartilage associated type II collagen and aggrecan markers. *Curr Opin Rheumatol* 2022;34(1):54-60.

138. Luyten FP, Bierma-Zeinstra S, Dell'Accio F, et al. Toward classification criteria for early osteoarthritis of the knee. *Semin Arthritis Rheum* 2018;47(4):457-63. doi: 10.1016/j.semarthrit.2017.08.006 [published Online First: 2017/09/18]

139. Collins N, Prinsen C, Christensen R, et al. Knee Injury and Osteoarthritis Outcome Score (KOOS): systematic review and meta-analysis of measurement properties. *Osteoarthritis Cartilage* 2016;24(8):1317-29.

140. Collins NJ, Misra D, Felson DT, et al. Measures of knee function: International Knee Documentation Committee (IKDC) Subjective Knee Evaluation Form, Knee Injury and Osteoarthritis Outcome Score (KOOS), Knee Injury and Osteoarthritis Outcome Score Physical Function Short Form (KOOS-PS), Knee Outcome Survey Activities of Daily Living Scale (KOS-ADL), Lysholm Knee Scoring Scale, Oxford Knee Score (OKS), Western Ontario and McMaster Universities Osteoarthritis Index (WOMAC), Activity Rating Scale (ARS), and Tegner Activity Score (TAS). *Arthritis Care Res (Hoboken)* 2011;63 Suppl 11:S208-28. doi: 10.1002/acr.20632 [published Online First: 2012/05/25]

141. Hawker GA, Mian S, Kendzerska T, et al. Measures of adult pain: Visual Analog Scale for Pain (VAS Pain), Numeric Rating Scale for Pain (NRS Pain), McGill Pain Questionnaire (MPQ), Short-Form McGill Pain Questionnaire (SF-MPQ), Chronic Pain Grade Scale (CPGS), Short Form-36 Bodily Pain Scale (SF-36 BPS), and Measure of Intermittent and Constant Osteoarthritis Pain (ICOAP). *Arthritis Care Res (Hoboken)* 2011;63 Suppl 11:S240-52. doi: 10.1002/acr.20543 [published Online First: 2012/05/25]

142. Li T, Vedula SS, Hadar N, et al. Innovations in data collection, management, and archiving for systematic reviews. *Ann Intern Med* 2015;162(4):287-94.

143. Harris PA, Taylor R, Minor BL, et al. The REDCap consortium: Building an international community of software platform partners. *J Biomed Inform* 2019;95:103208. doi: 10.1016/j.jbi.2019.103208 [published Online First: 2019/05/13]

144. Cohen J. A coefficient of agreement for nominal scales. *Educational and psychological measurement* 1960;20(1):37-46.

145. McHugh ML. Interrater reliability: the kappa statistic. *Biochem Med (Zagreb)* 2012;22(3):276-82.

For peer review only

146. Buscemi N, Hartling L, Vandermeer B, et al. Single data extraction generated more errors than double data extraction in systematic reviews. *J Clin Epidemiol* 2006;59(7):697-703. doi: 10.1016/j.jclinepi.2005.11.010

147. Meursinge Reynders R, Ladu L, Di Girolamo N. Contacting of authors modified crucial outcomes of systematic reviews but was poorly reported, not systematic, and produced conflicting results. *J Clin Epidemiol* 2019;115:64-76. doi: 10.1016/j.jclinepi.2019.07.001

148. Mullan RJ, Flynn DN, Carlberg B, et al. Systematic reviewers commonly contact study authors but do so with limited rigor. *J Clin Epidemiol* 2009;62(2):138-42. doi: 10.1016/j.jclinepi.2008.08.002

149. Higgins JPT TJ, Chandler J, Cumpston M, Li T, Page MJ, Welch VA (editors). Chapter 5: Collecting data. In: Li T HJ, Deeks JJ (editors), ed. Cochrane Handbook for Systematic Reviews of Interventions version 63 (updated February 2022): Cochrane, 2022.

150. Aromataris E, Fernandez R, Godfrey CM, et al. Summarizing systematic reviews: methodological development, conduct and reporting of an umbrella review approach. *Int J Evid Based Healthc* 2015;13(3):132-40. doi: 10.1097/XEB.0000000000000055

151. Tufanaru C, Munn Z, Aromataris E, et al. Chapter 3: Systematic reviews of effectiveness. In: Aromataris E, Munn Z, eds. JBI Manual for Evidence Synthesis: JBI, 2020.

152. Munn Z, Moola S, Lisy K, et al. Methodological guidance for systematic reviews of observational epidemiological studies reporting prevalence and cumulative incidence data. *Int J Evid Based Healthc* 2015;13(3):147-53. doi: 10.1097/XEB.0000000000000054

153. Moola S, Munn Z, Tufanaru C, et al. Chapter 7: Systematic reviews of etiology and risk. In: Aromataris E, Munn Z, eds. JBI Manual for Evidence Synthesis: JBI, 2020.

154. Maher JM, Markey JC, Ebert-May D. The Other Half of the Story: Effect Size Analysis in Quantitative Research. *CBE Life Sci Educ* 2013;12(3):345-51. doi: 10.1187/cbe.13-04-0082

155. Rosenthal JA. Qualitative Descriptors of Strength of Association and Effect Size. *J Soc Serv Res* 1996;21(4):37-59. doi: 10.1300/j079v21n04_02

156. Cohen J. A power primer. *Psychological bulletin* 1992;112(1):155.

157. Cohen J. Statistical Power Analysis for the Behavioral Sciences. 2nd Edition ed. New York: Routledge 1988.

158. Sonnery-Cottet B, Saithna A, Quelard B, et al. Arthrogenic muscle inhibition after ACL reconstruction: a scoping review of the efficacy of interventions. *Br J Sports Med* 2019;53(5):289-98. doi: 10.1136/bjsports-2017-098401

159. Fedak KM, Bernal A, Capshaw ZA, et al. Applying the Bradford Hill criteria in the 21st century: how data integration has changed causal inference in molecular epidemiology. *Emerg Themes Epidemiol* 2015;12(1) doi: 10.1186/s12982-015-0037-4

For peer review only - http://bmjopen.bmj.com/site/about/guidelines.xhtml

160. Hill AB. The Enviroment and Disease: Association or Causation? *Proc R Soc Med* 1965;58(5):295-300.

161. Schünemann H, Hill S, Guyatt G, et al. The GRADE approach and Bradford Hill's criteria for causation. *J Epidemiol Community Health* 2011;65(5):392. doi: 10.1136/jech.2010.119933

For peer review only

For peer review only - http://bmjopen.bmj.com/site/about/guidelines.xhtml

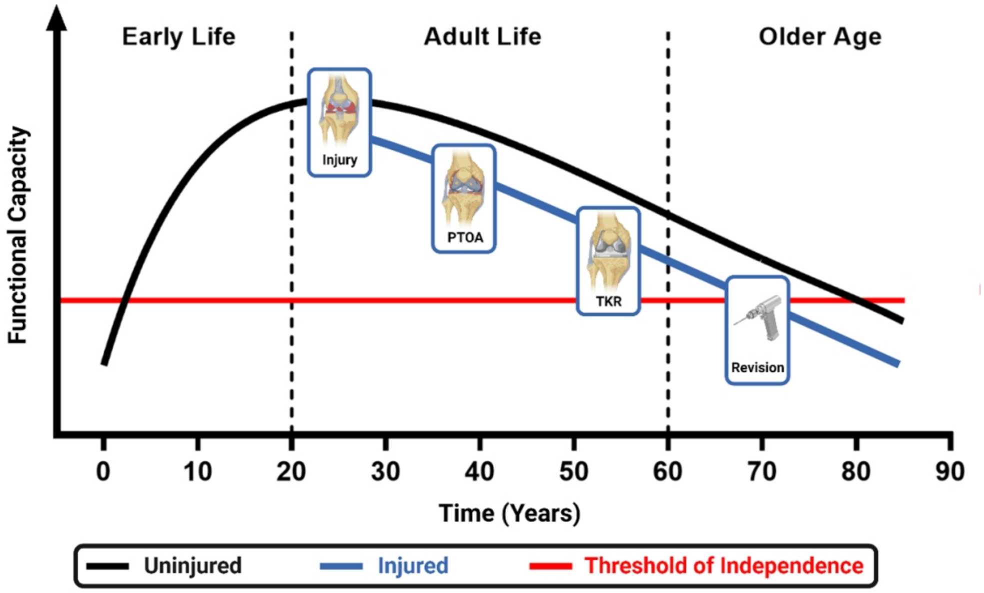

Figure 1. Hypothetical trajectory of injury to lifelong disease burden. Incidence of knee injury leads to post-traumatic osteoarthritis (PTOA),[28-31] which leads to earlier age for total knee replacement (TKR),[34] which increases the risk of revision surgery.[38] This accelerates the loss of knee functional capacity, which could impact the ability to complete self-sustaining tasks.

402x244mm (87 x 87 DPI)

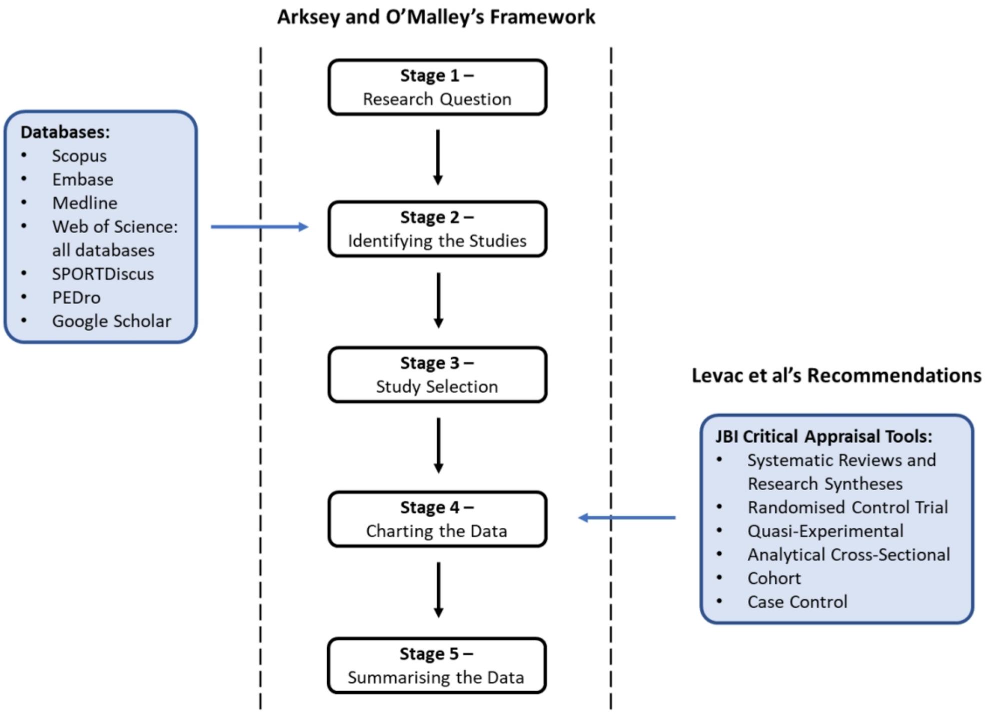

Figure 2. Outline of the study methodology for the present review incorporating Arksey and O'Malley's[103] framework and Levac's[99] recommendations (inclusion of methodological quality appraisal) for conducting a scoping review. JBI denotes the Joanna Briggs Institute.

638x462mm (59 x 59 DPI)

Supplementary Material 1. Preferred Reporting Items for Systematic review and Meta-Analysis Protocols 2015 Checklist[1,2] Adapted for Scoping Reviews[3]

Section and topic	Item No	Checklist item	
**ADMINISTRATIVE INFORMATION**			
Protocol title:			
Identification	1a	Identify the report as a protocol of a scoping review	✓
Update	1b	If the protocol is for an update of a previous scoping review, identify as such	✓
Registration	2	If registered, provide the name of the registry (such as Open Science Framework, Figshare or Research Gate) and registration number	✓
Authors:			
Contact	3a	Provide name, institutional affiliation, e-mail address of all protocol authors; provide physical mailing address of corresponding author	✓
Contributions	3b	Describe contributions of protocol authors and identify the guarantor of the review	✓
Amendments	4	If the protocol represents an amendment of a previously completed or published protocol, identify as such and list changes; otherwise, state plan for documenting important protocol amendments	✓
Support:			
Sources	5a	Indicate sources of financial or other support for the review	✓
Sponsor	5b	Provide name for the review funder and/or sponsor	✓
Role of sponsor or funder	5c	Describe roles of funder(s), sponsor(s), and/or institution(s), if any, in developing the protocol	✓
**INTRODUCTION**			
Rationale	6	Describe the rationale for the review in the context of what is already known	✓
Objectives	7	Provide an explicit statement of the question(s) the review will address with reference to the inclusion/exclusion criteria	✓

For peer review only

METHODS			
Eligibility criteria	8	Specify the study characteristics (such as PICO/PCC or other, study design, setting, time frame) and report characteristics (such as years considered, language, publication status) to be used as criteria for eligibility for the review	✓
Information sources	9	Describe all intended information sources (such as electronic databases, contact with study authors, trial registers or other grey literature sources) with planned dates of coverage	✓
Search strategy	10	Present draft of search strategy to be used for at least one electronic database, including planned limits, such that it could be repeated	✓
**Study records:**			
Data management	11a	Describe the mechanism(s) that will be used to manage records and data throughout the review	✓
Selection process	11b	State the process that will be used for selecting studies (such as two independent reviewers) through each phase of the review (that is, screening, eligibility, and inclusion)	✓
Data collection process	11c	Describe planned method of extracting data from reports (such as piloting forms, done independently, in duplicate), any processes for obtaining and confirming data from investigators	✓
Data items	12	List and define all variables for which data will be sought (such as PICO/PCC or other items, funding sources), any pre-planned data assumptions and simplifications	✓
Outcomes and prioritization	13	List and define all outcomes for which data will be sought, including prioritization of main and additional outcomes, with rationale (applicable if extracting outcomes)	✓
Risk of bias in individual studies	14	Describe anticipated methods for assessing risk of bias of individual studies, including whether this will be done at the outcome or study level, or both; state how this information will be used in data synthesis	✓
Data synthesis	15a	Describe criteria under which study data will be presented	✓
	15b	Describe the planned approach to how, extracted data will be presented (such as figures, tables, evidence gaps maps)	✓
	15c	Describe any proposed additional analyses (such as thematic analyses)	✓

	15d	If quantitative synthesis is not appropriate, describe the type of summary planned	✓
Meta-bias(es)	16	Specify any planned assessment of meta-bias(es) (such as publication bias across studies, selective reporting within studies)	✓
Confidence in cumulative evidence	17	Describe how the strength of the body of evidence will be assessed (such as GRADE)	✓

1. Moher D, Shamseer L, Clarke M, et al. Preferred reporting items for systematic review and meta-analysis protocols (PRISMA-P) 2015 statement. *Syst Rev* 2015;4(1):1. doi: 10.1186/2046-4053-4-1
2. Shamseer L, Moher D, Clarke M, et al. Preferred reporting items for systematic review and meta-analysis protocols (PRISMA-P) 2015: elaboration and explanation. *BMJ* 2015;349(jan02 1):g7647-g47. doi: 10.1136/bmj.g7647
3. Peters MDJ, Godfrey C, McInerney P, et al. Best practice guidance and reporting items for the development of scoping review protocols. *JBI Evid Synth* 2022;20(4)

For peer review only

Supplementary Material 2. Search Terms

((“knee injury” OR “knee surgery” OR “cruciate ligament injury” OR “cruciate ligament reconstruction” OR ACL OR MCL OR PCL OR “meniscus injury” OR meniscectomy OR “cartilage injury” OR “chondroplasty”) AND (“physical activity” OR “physical inactivity” OR exercise OR “blood” OR “urine” OR “synovial fluid” OR “serum” OR “plasma” OR biomarker* OR inflamma* OR cytokine* OR “interleukin” OR biomechanic* OR “reaction force” OR “contact force” OR “knee load” OR “cumulative load*” OR moment OR “ground reaction force” OR GRF OR “musculoskeletal” OR “inverse dynamics” OR simulation* OR “muscle strength” OR “muscle contraction” OR neurophysiolog* OR “neuromuscular” OR physiolog* OR “strength” OR “weakness” OR “body composition” OR “body mass index” OR BMI OR fat OR adipos* OR obes* OR “muscle quality” OR “echo intensity” OR echogenicity OR “inter-muscular adipose” OR “intermuscular adipose” OR “intermuscular fat” OR “intermuscular fat” OR “intra-muscular adipose” OR “intramuscular adipose” OR “intra-muscular fat” OR “intramuscular fat” OR “muscle attenuation” OR “muscle density” OR morpholog* OR mass OR volume OR “cross-sectional area” OR “cross sectional area” OR “thickness” OR atrophy OR “bone mineral” OR “bone density”) AND (osteoarthriti* OR OA OR PTOA OR t1rho OR t1 OR t2 OR pro-collagen OR procollagen OR collagen OR proteoglycan OR biomarker OR biomarker* OR biochemical OR “pain” OR “symptoms” OR “swelling” OR “stiffness” OR “function” OR “quality of life”))

For peer review only

Supplementary Material 3. PTOA Disease and Illness Outcomes

Table 1. Disease Outcome Measures

**Diagnoses of Knee PTOA Disease**	
**Radiography Diagnostic System**	**Scoring System**
Kellgren and Lawrence (K/L) system[1]	A K/L score of 1 indicates early knee OA[130], and an >1 K/L score indicates knee OA system[1]
Osteoarthritis Research Society International (OARSI) Atlas criteria[2]	OARSI Atlas scores for knee OA diagnosis are joint space narrowing ≥ grade 2, or grade 1 joint space narrowing occurred in combination with grade 1 osteophyte[2]
**Magnetic Resonance Imaging Diagnostic System**	**Scoring System**
Magnetic Resonance Imaging Osteoarthritis Knee Score (MOAKS)[3]	For brevity due to the multi-tissue assessment lower scores for each tissue inside the joint indicates degeneration.[3]
Whole Organ Magnetic Resonance Imaging Score (WORMS)[4]	For brevity due to the multi-tissue assessment, lower scores for each tissue inside the joint indicates degeneration.[4]
Boston Leeds OA Knee Score (BLOKS)[5]	For brevity due to the multi-tissue assessment, lower scores for each tissue inside the joint indicates degeneration.[5]
**Indicators of Structural Deterioration Preceding PTOA Disease**	
**Measurement Tool**	**Index**
Magnetic resonance imaging (MRI) quantified cartilage thickness and volume[6]	Lower MRI cartilage thickness and volume indicates degenerated cartilage[6 7]
T1rho mapping[4]	Higher signal intensity of T1rho mapping indicates lower collagen and/or proteoglycan content[7 8]
T2 mapping[9]	Higher signal intensity of T2 mapping indicates lower collagen and/or proteoglycan content[7 8]
Delayed contrast-enhanced MRI of cartilage (dGEMRIC)[125 126]	Higher signal intensity for dGEMRIC is indicative of low glycosaminoglycan (GAG) content[7]
Ultrasonography (US) quantified cartilage thickness[10-12]	Lower US cartilage thickness and volume indicates degenerated cartilage[10-12]
**Systemic Biomarkers Indicating Structural Deterioration of Knee PTOA Disease**	

For peer review only

**Substrate Biomarkers**	**Index**
Matrix Metalloprotease 3 (MMP-3)[13]	Serum MMP-3 concentration positively associated with knee OA and cartilage volume loss[14-16]
Matrix Metalloprotease 13 (MMP-13)[17-21]	Serum MMP-13 concentration negatively associated with knee cartilage volume and positively associated with knee OA diagnosis[22]
**Collagen Biomarker**	**Index**
N-Propeptide of Collagen IIA (PIIANP)[23]	Serum PIIANP concentration negatively associated with knee osteophytes[24] and knee OA[23 25]
N-Propeptide of Collagen IIB (PIIBNP)[26]	Serum and Plasma concentrations negatively associated with joint space narrowing[27]
C-Telopeptide of Type II Collagen (CTX-II)[28]	Urinary concentration of CTX-II positively associated with knee OA diagnosis and cartilage degeneration[15 29-33]
C-Propeptide of Type II Collagen (CP-II)[34]	Serum concentration of CP-II negatively associated with knee OA diagnosis[34 35]
CTX-II:CP-II[35]	Ratio of urinary concentration of CTX-II to serum concentrations of CP-II positively associated with knee OA[35]
Collagen Type II Cleavage Product (C2C)[36]	Serum and urine C2C concentration positively associated with cartilage degeneration and knee joint pain worsening and joint space narrowing[25 37 38]
Col2-3/4 C-terminal cleavage product of types I and II collagen (C1,2C)[17]	Serum C1,2C concentration positively associated with knee OA diagnosis[38]
Neoepitope of Collagen X (C10C or Col10Neo)[39]	Serum C10C concentration positively associated with knee OA diagnosis[39] and plasma C10C/Col10Neo concentration positively associated with knee OA diagnosis[40]
**Proteoglycan Biomarker**	**Index**
Hyaluronic Acid (HA)[41]	Serum HA concentration positively associated with knee OA diagnosis and joint space narrowing[15 25 42-44]
Chondroitin Sulphate 846 Epitope (CS846)[45]	Serum CS846 concentration positively associated with femur cartilage thickening[46 47]
Aggrecan[48] or Aggrecan Fragments (ARGS)[49 50]	Serum aggrecan concentration at baseline inversely associated with joint space narrowing in medial compartment after 10 years of follow-up[51] and synovial fluid ARGS concentration negatively associated with joint space narrowing[52]
Cartilage Oligomeric Matrix Protein (COMP)[53]	Serum COMP concentration positively associate with knee OA diagnosis and cartilage degeneration[15 30 32 33 38 51 54-58]

For peer review only

Table 2. Illness Outcome Measures

Diagnosis of Knee PTOA Illness

Illness Diagnostic System	Structure	Scoring System
Van Luyten Criteria[59]	Based on the Knee Injury and Osteoarthritis Outcome Score (KOOS).[60] Consists of five sub-scales: **1**) pain, **2**) other symptomology, **3**) function in daily living, **4**) function in sport and recreation, **5**) knee related quality of life	Scoring <85% of two of the following four sub-scales:<br>**1**) pain<br>**2**) other symptomology<br>**3**) function in daily living<br>**5**) knee related quality of life score
Englund Criteria[61]	Based on the Knee Injury and Osteoarthritis Outcome Score (KOOS).[60] Consists of five sub-scales: **1**) pain, **2**) other symptomology, **3**) function in daily living, **4**) function in sport and recreation, **5**) knee related quality of life	Scoring of any two subscales:<br>**1**) pain ≤ 86.1%,<br>**2**) other symptomology ≤ 85.7%,<br>**3**) function in daily living ≤ 86.8%,<br>**4**) function in sport and recreation ≤ 85.0%<br>In addition to:<br>**5**) knee related quality of life score ≤ 87.5%

Indicators of Knee PTOA Illness

Single Index for Knee Symptomology and Function

Measurement Tool	Structure	Scoring System
International Knee Documentation Committee (IKDC)[62]	Consist of three domains: **1**) symptoms, including pain, stiffness, swelling, locking/catching, and giving way; **2**) sports and daily activities; and **3**) current knee function	IKDC score of 0 = greatest limitation with daily or sporting activities and presence of symptoms.<br>IKDC score of 100 = no limitation with daily or sporting activities and the absence of symptoms.
Oxford Knee Score (OKS)[63]	Single index for knee pain and function during daily activities	OKS score of 12 = better outcome<br>OKS score of 60 = poorer outcome
Knee Outcome Survey Activities of Daily Living (KOS-ADL)[64]	Single index with two sections: **1**) symptoms; **2**) functional limitations	KOS-ADL score of 0 = worst symptoms/limitations<br>KOS-ADL score of 100 = no symptoms/limitations
Western Ontario and McMaster Universities Osteoarthritis Index (WOMAC)[65]	Consists of three sub-scales **1**) pain severity during various position or movement, **2**) severity of joint stiffness, and **3**) difficulty performing daily functioning activities	Higher scores indicate worse pain, stiffness, or physical function.
Lysholm Knee Scoring Scale[66]	Single scale measuring instability, symptoms,	Lysholm Scale score of 0 = worst symptoms or disability

For peer review only

	and function	Lysholm Scale score of 100 = no symptoms or disability<br>Scores are categorized as excellent (95–100), good (84–94), fair (65–83), and poor (≤64).

Knee Symptomology

Measurement Tool	Structure	Scoring System
Knee Injury and Osteoarthritis Outcome Score (KOOS)[60]	Subscale of the KOOS: **1**) Pain, **2**) Other symptomology	KOOS sub-scale score of 0 = extreme problems<br>KOOS sub-scale score of 100 = no problems
Visual Analogue Scale (VAS)[67-69]	A single scale consists of 10cm scale ranging from 0-100	VAS score of 0 = no pain<br>VAS score of 100 = worst pain/worst possible pain
Numerical Rating Scale (NRS)[67-70]	A single scale consisting of eleven items ranging from 0-10	NRS score of 0 = no pain<br>NRS score of 10 = pain as bad as you can imagine/worst pain imaginable/most pain
Verbal Rating Scale (VRS)[67 68]	Contains a list of descriptors that best indicates pain level	Four levels of pain: none, mild, moderate, and severe
Kujala Anterior Knee Pain Scale (AKPS)[71]	A single scale containing 13 items assessing patellofemoral pain	Responses are summed to provide an overall index in which 100 represents no symptomology and 0 represents worst possible symptomology
McGill Pain Questionnaire (MPQ)[72 73]	Contains seventy-eight pain descriptor items categorized into 20 subclasses, each containing 2–6 words that fall into 4 major sub-scales: sensory (subclasses 1–10), affective (subclasses 11–15), evaluative (subclass 16), and miscellaneous (subclasses 17–20). In addition, there is a one item pain intensity scale	A higher MPQ scores indicate more severe pain experienced
Short-Form McGill Pain Questionnaire (SF-MPQ)[74]	Fifteen words taken from the original MPQ which fall into the subclasses of sensory (eleven words) and affective (4 words)	A higher SF-MPQ score indicates a more severe pain experience
Chronic Pain Grade Scale (CPGS)[75]	Compromised of seven items which are scored on an eleven-point Likert scale (0–10)	Grade 0 = no pain<br>Grade I = low disability-low intensity<br>Grade II = low disability-high intensity<br>Grade III = high disability-moderately limiting<br>Grade IV = high disability-severely limiting
Measure of Intermittent and Constant Osteoarthritis Pain (ICOAP)[76]	Eleven items in two domains with two supplementary items on intermittent pain predictability	ICOAP score of 0 = no pain<br>ICOAP score of 100 = extreme pain

For peer review only

Short Form Health Survey Body Pain Scale (SF-36) Version 1 (v1) and 2 (v2)[77]	Two items: 'intensity of bodily pain' (6-point scale) and 'interference of pain with normal activities' (5-point scale)	SF-36 scale ranges between 0–100 a higher score indicates a lack of bodily pain
**Function/Disability**		
**Measurement Tool**	**Structure**	**Scoring System**
Knee Injury and Osteoarthritis Outcome Score (KOOS)[60]	Subscales of the KOOS: **1**) Function in daily living, **2**) function in sport and recreation	KOOS sub-scale score of 0 = extreme problems KOOS sub-scale score of 100 = no problems
**Quality of Life**		
**Measurement Tool**	**Structure**	**Scoring System**
Short Form Health Survey (SF-12)[78]	Consists of 12 items grouped in 8 subscales. **1**) Physical functioning, **2**) Role limitations due to physical health, **3**) role limitations due to emotional problems, **4**) Energy fatigue, **5**) emotional well-being, **6**) social functioning, **7**) pain, **8**) general health.	Scores range for each subscale from 0-100, with a higher score indicates a more favourable physical and mental health state
Short Form Health Survey (SF-36)[77]	Consists of 36 items grouped in 8 subscales. **1**) Physical functioning, **2**) Role limitations due to physical health, **3**) role limitations due to emotional problems, **4**) Energy fatigue, **5**) emotional well-being, **6**) social functioning, **7**) pain, **8**) general health.	Scores range for each subscale from 0-100, with a higher score indicates a more favourable physical and mental health state
Musculoskeletal Health Questionnaire (MSK-HQ)[79]	Consists of 14 items measuring symptomology, function, physical activity level, symptom interference, difficulty with sleep, level of fatigue/low energy levels, emotional well-being, understanding of diagnosis and treatment, confidence to self-manage, and independence, and these are summed to provide overall impact from symptoms.	Scores range from 0-56, with a score of 0 reflecting lowest overall musculoskeletal health status.
Knee Injury and Osteoarthritis Outcome Score (KOOS)[60]	Subscale of the KOOS: Knee related quality of life	KOOS sub-scale score of 0 = extreme problems KOOS sub-scale score of 100 = no problems

For peer review only

References

1. Kellgren JH, Lawrence JS. Radiological Assessment of Osteo-Arthrosis. *Ann Rheum Dis* 1957;16(4):494-502. doi: 10.1136/ard.16.4.494
2. Altman RD, Gold GE. Atlas of individual radiographic features in osteoarthritis, revised. *Osteoarthritis Cartilage* 2007;15:A1-A56. doi: 10.1016/j.joca.2006.11.009
3. Hunter DJ, Guermazi A, Lo GH, et al. Evolution of semi-quantitative whole joint assessment of knee OA: MOAKS (MRI Osteoarthritis Knee Score). *Osteoarthritis Cartilage* 2011;19(8):990-1002. doi: 10.1016/j.joca.2011.05.004 [published Online First: 2011/06/08]
4. Peterfy CG, Guermazi A, Zaim S, et al. Whole-Organ Magnetic Resonance Imaging Score (WORMS) of the knee in osteoarthritis. *Osteoarthritis Cartilage* 2004;12(3):177-90. doi: 10.1016/j.joca.2003.11.003
5. Hunter DJ, Lo GH, Gale D, et al. The reliability of a new scoring system for knee osteoarthritis MRI and the validity of bone marrow lesion assessment: BLOKS (Boston Leeds Osteoarthritis Knee Score). *Ann Rheum Dis* 2008;67(2):206-11. doi: 10.1136/ard.2006.066183 [published Online First: 2007/05/03]
6. Eckstein F, Cicuttini F, Raynauld JP, et al. Magnetic resonance imaging (MRI) of articular cartilage in knee osteoarthritis (OA): morphological assessment. *Osteoarthritis Cartilage* 2006;14:46-75. doi: 10.1016/j.joca.2006.02.026
7. Blumenkrantz G, Majumdar S. Quantitative magnetic resonance imaging of articular cartilage in osteoarthritis. *Eur Cell Mater* 2007; 13. http://europepmc.org/abstract/MED/17506024

https://doi.org/10.22203/ecm.v013a08 (accessed 2007/05//).

8. Atkinson HF, Birmingham TB, Moyer RF, et al. MRI T2 and T1ρ relaxation in patients at risk for knee osteoarthritis: a systematic review and meta-analysis. *BMC Musculoskelet Disord* 2019;20(1):1-18.
9. Dunn TC, Lu Y, Jin H, et al. T2 relaxation time of cartilage at MR imaging: comparison with severity of knee osteoarthritis. *Radiology* 2004;232(2):592-8. doi: 10.1148/radiol.2322030976 [published Online First: 20040623]
10. Naredo E, Acebes C, Moller I, et al. Ultrasound validity in the measurement of knee cartilage thickness. *Ann Rheum Dis* 2009;68(8):1322-7. doi: 10.1136/ard.2008.090738 [published Online First: 2008/08/08]
11. Abraham AM, Goff I, Pearce MS, et al. Reliability and validity of ultrasound imaging of features of knee osteoarthritis in the community. *BMC Musculoskelet Disord* 2011;12(1):70. doi: 10.1186/1471-2474-12-70
12. Schmitz RJ, Wang HM, Polprasert DR, et al. Evaluation of knee cartilage thickness: A comparison between ultrasound and magnetic resonance imaging methods. *The Knee* 2017;24(2):217-23. doi: 10.1016/j.knee.2016.10.004 [published Online First: 2016/12/05]
13. Okada Y, Shinmei M, Tanaka O, et al. Localization of matrix metalloproteinase 3 (stromelysin) in osteoarthritic cartilage and synovium. *Lab Invest* 1992;66(6):680-90. [published Online First: 1992/06/01]
14. Pelletier JP, Raynauld JP, Caron J, et al. Decrease in serum level of matrix metalloproteinases is predictive of the disease-modifying effect of osteoarthritis drugs assessed by quantitative MRI in patients with knee osteoarthritis. *Ann Rheum Dis* 2010;69(12):2095-101. doi: 10.1136/ard.2009.122002 [published Online First: 2010/06/24]
15. Joseph GB, Nevitt MC, McCulloch CE, et al. Associations between molecular biomarkers and MR-based cartilage composition and knee joint morphology: data from the Osteoarthritis Initiative. *Osteoarthritis Cartilage* 2018;26(8):1070-77. doi: 10.1016/j.joca.2018.04.019 [published Online First: 2018/05/29]
16. Pengas I, Eldridge S, Assiotis A, et al. MMP-3 in the peripheral serum as a biomarker of knee osteoarthritis, 40 years after open total knee meniscectomy. *J Exp Orthop* 2018;5(1):1-8.

For peer review only - http://bmjopen.bmj.com/site/about/guidelines.xhtml

17. Billinghurst RC, Dahlberg L, Ionescu M, et al. Enhanced cleavage of type II collagen by collagenases in osteoarthritic articular cartilage. *J Clin Invest* 1997;99(7):1534-45. doi: 10.1172/JCI119316 [published Online First: 1997/04/01]
18. Neuhold LA, Killar L, Zhao W, et al. Postnatal expression in hyaline cartilage of constitutively active human collagenase-3 (MMP-13) induces osteoarthritis in mice. *J Clin Invest* 2001;107(1):35-44. doi: 10.1172/JCI10564 [published Online First: 2001/01/03]
19. Shlopov BV, Lie WR, Mainardi CL, et al. Osteoarthritic lesions: involvement of three different collagenases. *Arthritis Rheum* 1997;40(11):2065-74. doi: 10.1002/art.1780401120 [published Online First: 1997/11/19]
20. Wang M, Sampson ER, Jin H, et al. MMP13 is a critical target gene during the progression of osteoarthritis. *Arthritis Res Ther* 2013;15(1):R5. doi: 10.1186/ar4133 [published Online First: 2013/01/10]
21. Hu Q, Ecker M. Overview of MMP-13 as a promising target for the treatment of osteoarthritis. *Int J Mol Sci* 2021;22(4):1742. doi: 10.3390/ijms22041742
22. Ruan G, Xu J, Wang K, et al. Associations between knee structural measures, circulating inflammatory factors and MMP13 in patients with knee osteoarthritis. *Osteoarthritis Cartilage* 2018;26(8):1063-69. doi: 10.1016/j.joca.2018.05.003 [published Online First: 2018/05/14]
23. Rousseau JC, Sandell LJ, Delmas PD, et al. Development and clinical application in arthritis of a new immunoassay for serum type IIA procollagen NH2 propeptide. *Methods Mol Med* 2004;101:25-37. doi: 10.1385/1-59259-821-8:025 [published Online First: 2004/08/10]
24. Daghestani HN, Jordan JM, Renner JB, et al. Serum N-propeptide of collagen IIA (PIIANP) as a marker of radiographic osteoarthritis burden. *PLoS One* 2017;12(12):e0190251. doi: 10.1371/journal.pone.0190251
25. Kraus VB, Collins JE, Hargrove D, et al. Predictive validity of biochemical biomarkers in knee osteoarthritis: data from the FNIH OA Biomarkers Consortium. *Ann Rheum Dis* 2017;76(1):186-95. doi: 10.1136/annrheumdis-2016-209252 [published Online First: 2016/06/15]
26. Luo Y, He Y, Reker D, et al. A Novel High Sensitivity Type II Collagen Blood-Based Biomarker, PRO-C2, for Assessment of Cartilage Formation. *International Journal of Molecular Sciences* 2018;19(11):3485. doi: 10.3390/ijms19113485
27. Luo Y, Samuels J, Krasnokutsky S, et al. A low cartilage formation and repair endotype predicts radiographic progression of symptomatic knee osteoarthritis. *J Orthop Traumatol* 2021;22(1) doi: 10.1186/s10195-021-00572-0
28. Christgau S, Garnero P, Fledelius C, et al. Collagen type II C-telopeptide fragments as an index of cartilage degradation. *Bone* 2001;29(3):209-15. doi: 10.1016/s8756-3282(01)00504-x [published Online First: 2001/09/15]
29. Cheng H, Hao B, Sun J, et al. C-terminal cross-linked telopeptides of type II collagen as biomarker for radiological knee osteoarthritis: a meta-analysis. *Cartilage* 2020;11(4):512-20. doi: 10.1177/1947603518798884 [published Online First: 2018/09/18]
30. Valdes AM, Meulenbelt I, Chassaing E, et al. Large scale meta-analysis of urinary C-terminal telopeptide, serum cartilage oligomeric protein and matrix metalloprotease degraded type II collagen and their role in prevalence, incidence and progression of osteoarthritis. *Osteoarthritis Cartilage* 2014;22(5):683-9. doi: 10.1016/j.joca.2014.02.007 [published Online First: 2014/03/01]
31. Dam EB, Byrjalsen I, Karsdal MA, et al. Increased urinary excretion of C-telopeptides of type II collagen (CTX-II) predicts cartilage loss over 21 months by MRI. *Osteoarthritis Cartilage* 2009;17(3):384-9. doi: 10.1016/j.joca.2008.07.009 [published Online First: 2008/09/05]
32. Sowers MF, Karvonen-Gutierrez CA, Yosef M, et al. Longitudinal changes of serum COMP and urinary CTX-II predict X-ray defined knee osteoarthritis severity and stiffness in women.

For peer review only

Osteoarthritis Cartilage 2009;17(12):1609-14. doi: 10.1016/j.joca.2009.06.001 [published Online First: 2009/07/01]

33. Hao HQ, Zhang JF, He QQ, et al. Cartilage oligomeric matrix protein, C-terminal cross-linking telopeptide of type II collagen, and matrix metalloproteinase-3 as biomarkers for knee and hip osteoarthritis (OA) diagnosis: a systematic review and meta-analysis. *Osteoarthritis Cartilage* 2019;27(5):726-36. doi: 10.1016/j.joca.2018.10.009 [published Online First: 2018/11/06]
34. Nelson F, Dahlberg L, Laverty S, et al. Evidence for altered synthesis of type II collagen in patients with osteoarthritis. *J Clin Invest* 1998;102(12):2115-25. doi: 10.1172/JCI4853 [published Online First: 1998/12/17]
35. Cibere J, Zhang H, Garnero P, et al. Association of biomarkers with pre-radiographically defined and radiographically defined knee osteoarthritis in a population-based study. *Arthritis Rheum* 2009;60(5):1372-80. doi: 10.1002/art.24473 [published Online First: 2009/05/01]
36. Poole AR, Ionescu M, Fitzcharles MA, et al. The assessment of cartilage degradation in vivo: development of an immunoassay for the measurement in body fluids of type II collagen cleaved by collagenases. *J Immunol Methods* 2004;294(1-2):145-53. doi: 10.1016/j.jim.2004.09.005 [published Online First: 2005/01/11]
37. King KB, Lindsey CT, Dunn TC, et al. A study of the relationship between molecular biomarkers of joint degeneration and the magnetic resonance-measured characteristics of cartilage in 16 symptomatic knees. *Magn Reson Imaging* 2004;22(8):1117-23. doi: 10.1016/j.mri.2004.08.001
38. Kong SY, Stabler TV, Criscione LG, et al. Diurnal variation of serum and urine biomarkers in patients with radiographic knee osteoarthritis. *Arthritis Rheum* 2006;54(8):2496-504. doi: 10.1002/art.21977 [published Online First: 2006/07/27]
39. He Y, Siebuhr AS, Brandt-Hansen NU, et al. Type X collagen levels are elevated in serum from human osteoarthritis patients and associated with biomarkers of cartilage degradation and inflammation. *BMC Musculoskelet Disord* 2014;15(1):309. doi: 10.1186/1471-2474-15-309
40. He Y, Manon-Jensen T, Arendt-Nielsen L, et al. Potential diagnostic value of a type X collagen neo-epitope biomarker for knee osteoarthritis. *Osteoarthritis Cartilage* 2019;27(4):611-20. doi: 10.1016/j.joca.2019.01.001
41. Laurent TC, Laurent UB, Fraser JRE. Serum hyaluronan as a disease marker. *Ann Med* 1996;28(3):241-53.
42. Georges C, Vigneron H, Ayral X, et al. Serum biologic markers as predictors of disease progression in osteoarthritis of the knee. *Arthritis Rheum* 1997;40(3):590-1. doi: 10.1002/art.1780400333 [published Online First: 1997/03/01]
43. Elliott AL, Kraus VB, Luta G, et al. Serum hyaluronan levels and radiographic knee and hip osteoarthritis in African Americans and Caucasians in the Johnston County Osteoarthritis Project. *Arthritis Rheum* 2005;52(1):105-11. doi: 10.1002/art.20724 [published Online First: 2005/01/11]
44. Sharif M, George E, Shepstone L, et al. Serum hyaluronic acid level as a predictor of disease progression in osteoarthritis of the knee. *Arthritis Rheum* 1995;38(6):760-7. doi: 10.1002/art.1780380608 [published Online First: 1995/06/01]
45. Rizkalla G, Reiner A, Bogoch E, et al. Studies of the articular cartilage proteoglycan aggrecan in health and osteoarthritis. Evidence for molecular heterogeneity and extensive molecular changes in disease. *J Clin Invest* 1992;90(6):2268-77. doi: 10.1172/JCI116113 [published Online First: 1992/12/01]
46. Chu CR, Sheth S, Erhart-Hledik JC, et al. Mechanically stimulated biomarkers signal cartilage changes over 5 years consistent with disease progression in medial knee osteoarthritis patients. *J Orthop Res* 2018;36(3):891-97. doi: 10.1002/jor.23720 [published Online First: 2017/09/02]

For peer review only

47. Mazzuca SA, Poole AR, Brandt KD, et al. Associations between joint space narrowing and molecular markers of collagen and proteoglycan turnover in patients with knee osteoarthritis. *J Rheumatol* 2006;33(6):1147-51.
48. Lohmander SL, Neame PJ, Sandy JD. The structure of aggrecan fragments in human synovial fluid. evidence that aggrecanase mediates cartilage degradation in inflammatory joint disease, joint injury, and osteoarthritis. *Arthritis Rheum* 1993;36(9):1214-22. doi: https://doi.org/10.1002/art.1780360906
49. Larsson S, Lohmander LS, Struglics A. An ARGS-aggrecan assay for analysis in blood and synovial fluid. *Osteoarthritis Cartilage* 2014;22(2):242-9. doi: 10.1016/j.joca.2013.12.010 [published Online First: 2013/12/24]
50. Larsson S, Lohmander LS, Struglics A. Synovial fluid level of aggrecan ARGS fragments is a more sensitive marker of joint disease than glycosaminoglycan or aggrecan levels: a cross-sectional study. *Arthritis Res Ther* 2009;11(3):R92. doi: 10.1186/ar2735
51. Blumenfeld O, Williams FMK, Hart DJ, et al. Association between cartilage and bone biomarkers and incidence of radiographic knee osteoarthritis (RKOA) in UK females: a prospective study. *Osteoarthritis Cartilage* 2013;21(7):923-29. doi: 10.1016/j.joca.2013.04.009
52. Larsson S, Englund M, Struglics A, et al. Association between synovial fluid levels of aggrecan ARGS fragments and radiographic progression in knee osteoarthritis. *Arthritis Res Ther* 2010;12(6):R230. doi: 10.1186/ar3217
53. Saxne T, Heinegård D. Cartilage oligomeric matrix protein: a novel marker of cartilage turnover detectable in synovial fluid and blood. *Rheumatology* 1992;31(9):583-91. doi: 10.1093/rheumatology/31.9.583
54. Bi X. Correlation of serum cartilage oligomeric matrix protein with knee osteoarthritis diagnosis: a meta-analysis. *J Orthop Surg Res* 2018;13(1):262. doi: 10.1186/s13018-018-0959-y [published Online First: 2018/10/21]
55. Vilim V, Olejarova M, Machacek S, et al. Serum levels of cartilage oligomeric matrix protein (COMP) correlate with radiographic progression of knee osteoarthritis. *Osteoarthritis Cartilage* 2002;10(9):707-13. doi: 10.1053/joca.2002.0819 [published Online First: 2002/08/31]
56. Verma P, Dalal K. Serum cartilage oligomeric matrix protein (COMP) in knee osteoarthritis: A novel diagnostic and prognostic biomarker. *J Orthop Res* 2013;31(7):999-1006. doi: 10.1002/jor.22324
57. Hoch JM, Mattacola CG, Medina McKeon JM, et al. Serum cartilage oligomeric matrix protein (sCOMP) is elevated in patients with knee osteoarthritis: a systematic review and meta-analysis. *Osteoarthritis Cartilage* 2011;19(12):1396-404. doi: 10.1016/j.joca.2011.09.005 [published Online First: 2011/10/18]
58. Clark AG, Jordan JM, Vilim V, et al. Serum cartilage oligomeric matrix protein reflects osteoarthritis presence and severity: The Johnston county osteoarthritis project. *Arthritis Rheum* 1999;42(11):2356-64. doi: 10.1002/1529-0131(199911)42:11<2356::aid-anr14>3.0.co;2-r
59. Luyten FP, Bierma-Zeinstra S, Dell'Accio F, et al. Toward classification criteria for early osteoarthritis of the knee. *Semin Arthritis Rheum* 2018;47(4):457-63. doi: 10.1016/j.semarthrit.2017.08.006 [published Online First: 2017/09/18]
60. Roos EM, Roos HP, Lohmander LS, et al. Knee Injury and Osteoarthritis Outcome Score (KOOS)—development of a self-administered outcome measure. *J Orthop Sports Phys Ther* 1998;28(2):88-96.
61. Englund M, Roos EM, Lohmander LS. Impact of type of meniscal tear on radiographic and symptomatic knee osteoarthritis: a sixteen-year followup of meniscectomy with matched controls. *Arthritis Rheum* 2003;48(8):2178-87. doi: 10.1002/art.11088 [published Online First: 2003/08/09]

62. Anderson AF, Irrgang JJ, Kocher MS, et al. The International Knee Documentation Committee subjective knee evaluation form. *Am J Sports Med* 2006;34(1):128-35. doi: 10.1177/0363546505280214
63. Dawson J, Fitzpatrick R, Murray D, et al. Questionnaire on the perceptions of patients about total knee replacement. *J Bone Joint Surg Br* 1998;80(1):63-9. doi: 10.1302/0301-620x.80b1.7859
64. Irrgang JJ, Snyder-Mackler L, Wainner RS, et al. Development of a patient-reported measure of function of the knee. *J Bone Joint Surg Am* 1998;80(8):1132-45. doi: 10.2106/00004623-199808000-00006
65. Bellamy N, Buchanan WW, Goldsmith CH, et al. Validation study of WOMAC: a health status instrument for measuring clinically important patient relevant outcomes to antirheumatic drug therapy in patients with osteoarthritis of the hip or knee. *J Rheumatol* 1988;15(12):1833-40.
66. Lysholm J, Gillquist J. Evaluation of knee ligament surgery results with special emphasis on use of a scoring scale. *Am J Sports Med* 1982;10(3):150-54. doi: 10.1177/036354658201000306
67. Alghadir AH, Anwer S, Iqbal A, et al. Test-retest reliability, validity, and minimum detectable change of visual analog, numerical rating, and verbal rating scales for measurement of osteoarthritic knee pain. *J Pain Res* 2018;11:851-56. doi: 10.2147/JPR.S158847 [published Online First: 2018/05/08]
68. Ferraz MB, Quaresma MR, Aquino LR, et al. Reliability of pain scales in the assessment of literate and illiterate patients with rheumatoid arthritis. *J Rheumatol* 1990;17(8):1022-4. [published Online First: 1990/08/01]
69. Johnson C. Measuring pain. Visual analog scale versus numeric pain scale: what is the difference? *J Chiropr Med* 2005;4(1):43.
70. Farrar JT, Young Jr JP, LaMoreaux L, et al. Clinical importance of changes in chronic pain intensity measured on an 11-point numerical pain rating scale. *Pain* 2001;94(2):149-58.
71. Kujala UM, Jaakkola LH, Koskinen SK, et al. Scoring of patellofemoral disorders. *Arthroscopy* 1993;9(2):159-63. doi: 10.1016/s0749-8063(05)80366-4
72. Burckhardt CS. The use of the McGill Pain Questionnaire in assessing arthritis pain. *Pain* 1984;19(3):305-14.
73. Melzack R. The McGill Pain Questionnaire: major properties and scoring methods. *Pain* 1975;1(3):277-99.
74. Melzack R. The short-form McGill pain questionnaire. *Pain* 1987;30(2):191-97.
75. Von Korff M, Ormel J, Keefe FJ, et al. Grading the severity of chronic pain. *Pain* 1992;50(2):133-49.
76. Hawker G, Davis A, French M, et al. Development and preliminary psychometric testing of a new OA pain measure–an OARSI/OMERACT initiative. *Osteoarthritis Cartilage* 2008;16(4):409-14.
77. Ware JE, Kosinski M, Keller S. SF-36 physical and mental health summary scales: a user's manual: Health Assessment Lab. 1994.
78. Ware J, Jr., Kosinski M, Keller SD. A 12-Item Short-Form Health Survey: construction of scales and preliminary tests of reliability and validity. *Med Care* 1996;34(3):220-33. doi: 10.1097/00005650-199603000-00003
79. Hill JC, Kang S, Benedetto E, et al. Development and initial cohort validation of the Arthritis Research UK Musculoskeletal Health Questionnaire (MSK-HQ) for use across musculoskeletal care pathways. *BMJ Open* 2016;6(8):e012331. doi: 10.1136/bmjopen-2016-012331

For peer review only

Supplementary Material 4. Title/Abstract Screening Tool

Screening Tool - Title/Abstract

Codebook

Data Dictionary Codebook

2022-08-02 19:40:25

Collapse all instruments

	#	Variable / Field Name	Field Label<br>*Field Note*	Field Attributes (Field Type, Validation, Choices, Calculations, etc.)
Instrument: **Screening Tool** (screening_tool)				Collapse
	1	record_id	Record ID	text
	2	reviewer_screening	Section Header: *Reviewer Details and Study Information*<br>Reviewer	radio, Required<br>1 Karl Morgan<br>2 James Cowburn<br>3 Mathew Farrow
	3	reviewer_date_screening	Date<br>*Just press 'Today'*	text (date_dmy), Required
	4	record_number_screening	Record number	text (number), Required
	5	narrative_review	Is the study a:<br><br>Critical review?Literature review?Mapping review?Mixed studies review/mixed method review?Overview?Qualitative systematic review/qualitative evidence synthesis?Rapid review?Scoping review?State of the art review?Systematic review?Systematic search and review?Systematic review? Umbrella review?<br>Grant and Booth (2009)<br>https://onlinelibrary.wiley.com/doi/full/10.1111/j.1471-1842.2009.00848.x	checkbox, Required<br>1 narrative_review___1 Yes<br>2 narrative_review___2 No<br><br>Stop actions on 1
	6	animal	Is the study an animal model?	checkbox, Required<br>1 animal___1 Yes<br>2 animal___2 No<br><br>Stop actions on 1
	7	in_silico	Is the study purely in silico?	checkbox, Required<br>1 in_silico___1 Yes<br>2 in_silico___2 No<br><br>Stop actions on 1
	8	cadavers	Is the study working with cadavers?	checkbox, Required<br>1 cadavers___1 Yes<br>2 cadavers___2 No<br><br>Stop actions on 1
	9	knee_injury	Section Header: *1. Population*<br>Do the participants have either:<br><br>An undefined intra-articular knee injury?<br><br>Or<br><br>Injuries and/or reparative surgeries for the following knee joint tissues?<br><br>LigamentMeniscusOr<br><br>At the distal end of femur or proximal end of tibia, was there a:<br><br>FractureDislocationContusion<br>*Please select one option*	checkbox, Required<br>1 knee_injury___1 Yes<br>2 knee_injury___2 No<br>3 knee_injury___3 Maybe<br><br>Stop actions on 2

	10	age	Are the participants aged 15-49?<br>*Please select one option*	checkbox, Required<br>1 | age___1 | Yes<br>2 | age___2 | No<br>3 | age___3 | Maybe<br><br>Stop actions on 2
	11	independent_variable	Section Header: *2. Independent Variable*<br>Does the study have an independent variable which measures:<br><br>Physical activityExerciseSystemic inflammation (e.g., IL-6 concentrations)Knee joint load (moments or MSK modelling)Adipose tissueMuscle strengthMuscle sizeIntra-muscular adipose tissueBone mineral content or density<br>Or does the study have an intervention involving:<br><br>Physical activity or exerciseManipulation of joint loadWeight lossDisuse or bedrest<br>*Please select one option*	checkbox, Required<br>1 | independent_variable___1 | Yes<br>2 | independent_variable___2 | No<br>3 | independent_variable___3 | Maybe<br><br>Stop actions on 2
	12	disease	Section Header: *3. Outcome Variable*<br>Does the study have a disease outcome variable of:<br><br>PTOA Diagnosis<br>Kellgren and Lawrence (K/L)Osteoarthritis Research Society International (OARSI) AtlasMRI Osteoarthritis Knee Score (MOAKS)Whole Organ Magnetic Resonance Imaging Score (WORMS)Boston Leeds OA Knee Score (BLOKS)<br>Imaging IndicatorMRI quantified cartilage thickness and volumeT1rho mappingT2 mappingDelayed contrast-enhanced MRI of cartilage (dGEMRIC)Ultrasound<br>BiomarkersMatrix Metalloprotease 3 (MMP-3)Matrix Metalloprotease 13 (MMP-13)N-Propeptide of Collagen IIA (PIIANP)C-Telopeptide of Type II Collagen (CTX-II)C-Propeptide of Type II Collagen (CP-II)Collagen Type II Cleavage Product (C2C)Col2-3/4 C-terminal cleavage product of types I and II collagen (C1,2C)Hyaluronic Acid (HA)Chondroitin Sulphate 846 Epitope (CS846)Aggrecan or Aggrecan Fragments (ARGS)Cartilage Oligomeric Matrix Protein (COMP)<br>Does the study have a illness outcome variable of:<br><br>Illness Diagnosis:Van Luyten CriteriaEnglund Criteria<br>Illness Indicator:International Knee Documentation Committee (IKDC)Oxford Knee Score (OKS)Knee Outcome Survey Activities of Daily Living (KOS-ADL)Western Ontario and McMaster Universities Osteoarthritis Index (WOMAC)Lysholm Knee Scoring ScaleKnee Injury and Osteoarthritis Outcome Score (KOOS)Visual Analogue Scale (VAS)Numerical Rating Scale (NRS)Verbal Rating Scale (VRS)McGill Pain Questionnaire (MPQ)Short-Form McGill Pain Questionnaire (SF-MPQ)Chronic Pain Grade Scale (CPGS)Measure of Intermittent and Constant Osteoarthritis Pain (ICOAP)Short Form Health Survey Body Pain Scale (BPI-SF-36)Modified Brief Pain Inventory Short Form for Patients with Osteoarthritis (BPI-SF-OA)Short Form Health Survey (SF-12)Short Form Health Survey (SF-36)Musculoskeletal Health Questionnaire (MSK-HQ)<br>*Please select one option*	checkbox, Required<br>1 | disease___1 | Yes<br>2 | disease___2 | No<br>3 | disease___3 | Maybe
	13	all_yes<br>Show the field ONLY if:<br>[knee_injury(1)]= 1 and [age (1)] = 1 and [independent_variable(1)] = 1 and [disease(1)] = 1	Section Header: *Summary*	descriptive<br>1 |
	14	knee_inj_maybe<br>Show the field ONLY if:<br>[knee_injury(3)]= 1 and [age (1)] = 1 and [independent_variable(1)] = 1 and [disease(1)] = 1		descriptive<br>1 |

	15	age_maybe<br>Show the field ONLY if: [knee_injury(1)]= 1 and [age(3)] = 1 and [independent_variable(1)] = 1 and [disease(1)] = 1		descriptive<br>1
	16	iv_maybe<br>Show the field ONLY if: [knee_injury(1)]= 1 and [age(1)] = 1 and [independent_variable(3)] = 1 and [disease(1)] = 1		descriptive<br>1
	17	ov_maybe<br>Show the field ONLY if: [knee_injury(1)]= 1 and [age(1)] = 1 and [independent_variable(1)] = 1 and [disease(3)] = 1		descriptive<br>1
	18	knee_inj_age_maybe<br>Show the field ONLY if: [knee_injury(3)]= 1 and [age(3)] = 1 and [independent_variable(1)] = 1 and [disease(1)] = 1		descriptive<br>1
	19	age_iv_maybe<br>Show the field ONLY if: [knee_injury(1)]= 1 and [age(3)] = 1 and [independent_variable(3)] = 1 and [disease(1)] = 1		descriptive<br>1
	20	iv_ov_maybe<br>Show the field ONLY if: [knee_injury(1)]= 1 and [age(1)] = 1 and [independent_variable(3)] = 1 and [disease(3)] = 1		descriptive<br>1
	21	knee_inj_ov_maybe<br>Show the field ONLY if: [knee_injury(3)]= 1 and [age(1)] = 1 and [independent_variable(1)] = 1 and [disease(3)] = 1		descriptive<br>1
	22	age_ov_maybe<br>Show the field ONLY if: [knee_injury(1)]= 1 and [age(3)] = 1 and [independent_variable(1)] = 1 and [disease(3)] = 1		descriptive<br>1
	23	all_maybe<br>Show the field ONLY if: [knee_injury(3)]= 1 and [age(3)] = 1 and [independent_variable(3)] = 1 and [disease(3)] = 1		descriptive<br>1
	24	age_iv_ov_maybe<br>Show the field ONLY if: [knee_injury(1)]= 1 and [age(3)] = 1 and [independent_variable(3)] = 1 and [disease(3)] = 1		descriptive<br>1
	25	knee_inj_iv_ov_maybe<br>Show the field ONLY if: [knee_injury(1)]= 1 and [age(3)] = 1 and [independent_variable(1)] = 1 and [disease(1)] = 1		descriptive<br>1

	26	knee_inj_age_ov_maybe<br>Show the field ONLY if: [knee_injury(1)]= 1 and [age (1)] = 1 and [independent_variable(3)] = 1 and [disease(1)] = 1		descriptive<br>1
	27	knee_inj_age_iv_maybe<br>Show the field ONLY if: [knee_injury(3)]= 1 and [age (3)] = 1 and [independent_variable(3)] = 1 and [disease(1)] = 1		descriptive<br>1
	28	decision	Decision	descriptive
	29	all_yes_dec<br>Show the field ONLY if: [knee_injury(1)]= 1 and [age (1)] = 1 and [independent_variable(1)] = 1 and [disease(1)] = 1	Select 'Yes' in Covidence	descriptive
	30	knee_inj_maybe_dec<br>Show the field ONLY if: [knee_injury(3)]= 1 and [age (1)] = 1 and [independent_variable(1)] = 1 and [disease(1)] = 1	Select 'Yes' in Covidence	descriptive
	31	age_maybe_dec<br>Show the field ONLY if: [knee_injury(1)]= 1 and [age (3)] = 1 and [independent_variable(1)] = 1 and [disease(1)] = 1	Select 'Yes' in Covidence	descriptive
	32	iv_maybe_dec<br>Show the field ONLY if: [knee_injury(1)]= 1 and [age (1)] = 1 and [independent_variable(3)] = 1 and [disease(1)] = 1	Select 'Yes' in Covidence	descriptive
	33	ov_maybe_dec<br>Show the field ONLY if: [knee_injury(1)]= 1 and [age (1)] = 1 and [independent_variable(1)] = 1 and [disease(3)] = 1	Select 'Yes' in Covidence	descriptive
	34	knee_inj_age_maybe_dec<br>Show the field ONLY if: [knee_injury(3)]= 1 and [age (3)] = 1 and [independent_variable(1)] = 1 and [disease(1)] = 1	Select 'Yes' in Covidence	descriptive
	35	knee_inj_iv_maybe_dec<br>Show the field ONLY if: [knee_injury(3)]= 1 and [age (1)] = 1 and [independent_variable(3)] = 1 and [disease(1)] = 1	Select 'Yes' in Covidence	descriptive
	36	age_iv_maybe_dec<br>Show the field ONLY if: [knee_injury(1)]= 1 and [age (3)] = 1 and [independent_variable(3)] = 1 and [disease(1)] = 1	Select 'Yes' in Covidence	descriptive
	37	iv_ov_maybe_dec<br>Show the field ONLY if: [knee_injury(1)]= 1 and [age (1)] = 1 and [independent_variable(3)] = 1 and [disease(3)] = 1	Select 'Yes' in Covidence	descriptive

	38	age_ov_maybe_dec<br>Show the field ONLY if: [knee_injury(1)]= 1 and [age(3)] = 1 and [independent_variable(1)] = 1 and [disease(3)] = 1	Select 'Yes' in Covidence	descriptive
	39	knee_inj_ov_maybe_dec<br>Show the field ONLY if: [knee_injury(1)]= 1 and [age(1)] = 1 and [independent_variable(1)] = 1 and [disease(3)] = 1	Select 'Yes' in Covidence	descriptive
	40	all_maybe_dec<br>Show the field ONLY if: [knee_injury(3)]= 1 and [age(3)] = 1 and [independent_variable(3)] = 1 and [disease(3)] = 1	Select 'Maybe' in Covidence	descriptive
	41	age_iv_ov_maybe_dec<br>Show the field ONLY if: [knee_injury(1)]= 1 and [age(3)] = 1 and [independent_variable(3)] = 1 and [disease(3)] = 1	Select 'Maybe' in Covidence	descriptive
	42	knee_inj_iv_ov_maybe_dec<br>Show the field ONLY if: [knee_injury(3)]= 1 and [age(1)] = 1 and [independent_variable(3)] = 1 and [disease(3)] = 1	Select 'Maybe' in Covidence	descriptive
	43	knee_inj_age_ov_maybe_dec<br>Show the field ONLY if: [knee_injury(3)]= 1 and [age(3)] = 1 and [independent_variable(1)] = 1 and [disease(3)] = 1	Select 'Maybe' in Covidence	descriptive
	44	knee_inj_age_iv_maybe_dec<br>Show the field ONLY if: [knee_injury(3)]= 1 and [age(3)] = 1 and [independent_variable(3)] = 1 and [disease(1)] = 1	Select 'Maybe' in Covidence	descriptive
	45	screening_tool_complete	Section Header: *Form Status*<br>Complete?	dropdown<br>0 Incomplete<br>1 Unverified<br>2 Complete

Supplementary Material 5. Protocol for Contacting Authors

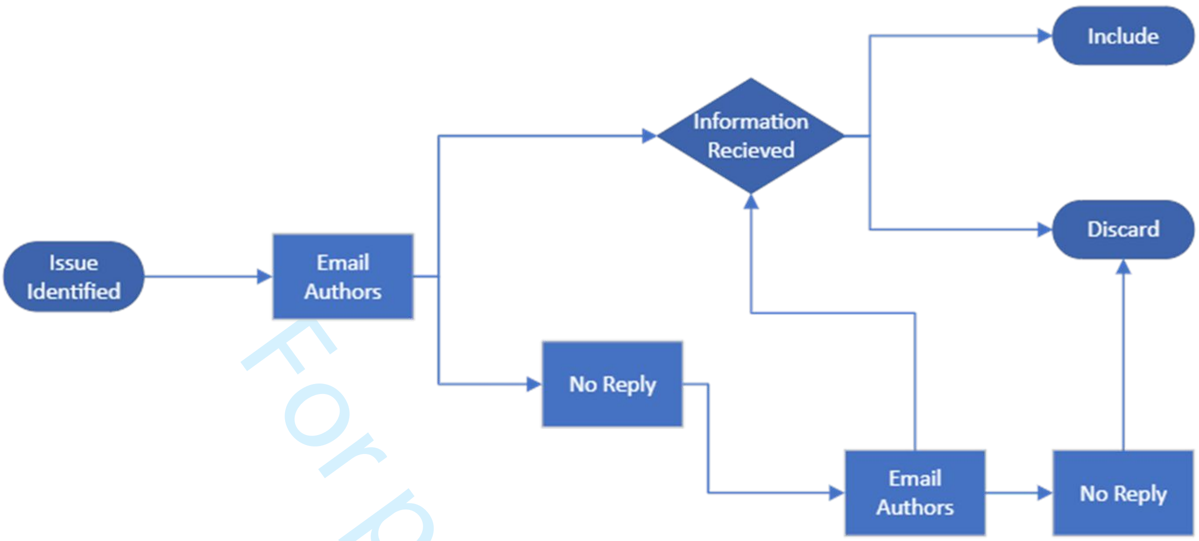

For peer review only

Supplementary Material 6. Johanna Brigg's Institute (JBI) Appraisal Criteria

REDCap Flow Diagram for Critical Appraisal

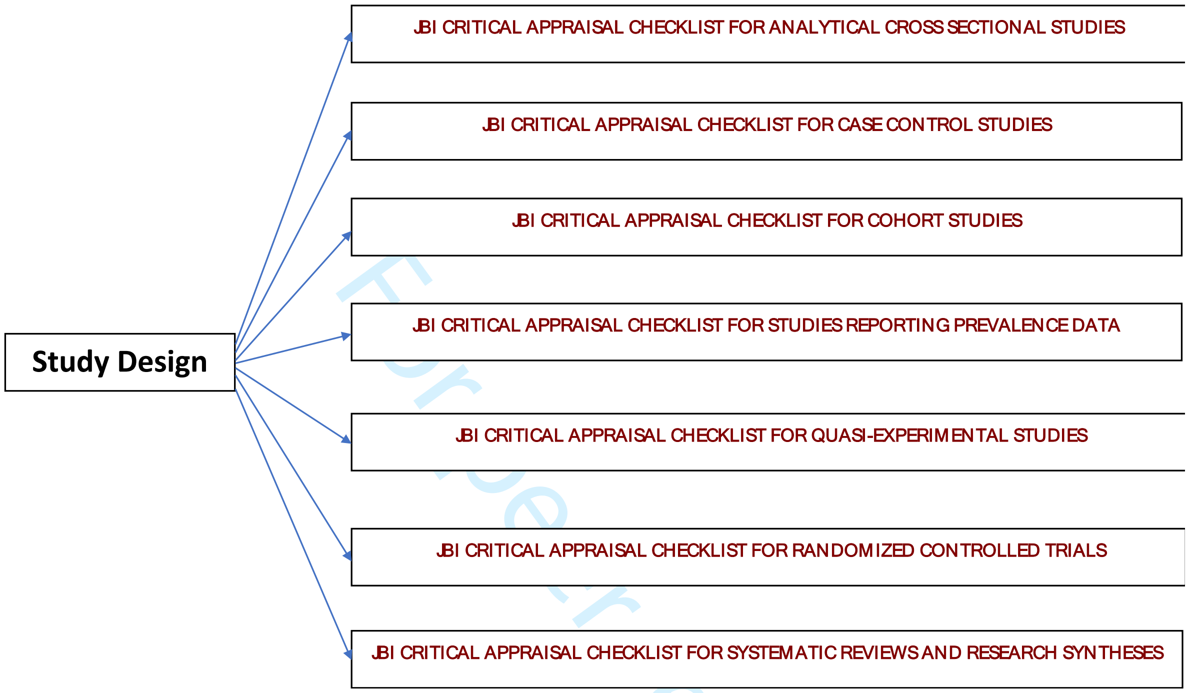

For peer review only

JBI Quality Appraisal

Codebook

Data Dictionary Codebook

2022-08-02 19:46:29

Collapse all instruments

	#	Variable / Field Name	Field Label<br>*Field Note*	Field Attributes (Field Type, Validation, Choices, Calculations, etc.)
Instrument: **Study Design** (study_design) Collapse				
	1	record_id	Record ID	text
	2	study_design	Please select the study design	radio<br>1 Analytical Cross-Sectional Study<br>2 Case Control Study<br>3 Cohort Study<br>4 Prevalence Study<br>5 Quasi-Experimental Study<br>6 Randomised Control Trial<br>7 Systematic Review and Research Synthesis<br>8 Unclear
	3	study_design_complete	Section Header: *Form Status*<br>Complete?	dropdown<br>0 Incomplete<br>1 Unverified<br>2 Complete
Instrument: **Study Design Unclear** (study_design_unclear) Collapse				
	4	reviewer_name_unclear	Section Header: *Reviewer Details*<br>Reviewer	dropdown<br>1 Karl Morgan<br>2 James Cowburn<br>3 Matthew Farrow
	5	reviewer_date_unclear	Date reviewed<br>*Just press 'Today'*	text (date_dmy)
	6	authors_unclear	Section Header: *Study Information*<br>Authors	text
	7	year_unclear	Publication year	text
	8	record_number_unclear	Record number	text
	9	study_design_unclear_complete	Section Header: *Form Status*<br>Complete?	dropdown<br>0 Incomplete<br>1 Unverified<br>2 Complete
Instrument: **JBI Critical Appraisal Checklist for Analytical Cross-Sectional Studies** (jbi_critical_appraisal_checklist_for_analytical_cr) Collapse				
	10	reviewer_name_cross	Section Header: *Reviewer Details*<br>Reviewer	dropdown<br>1 Karl Morgan<br>2 James Cowburn<br>3 Matthew Farrow
	11	reviewer_date_cross	Date reviewed<br>*Just press 'Today'*	text (date_dmy)
	12	authors_cross	Section Header: *Study Information*<br>Authors	text
	13	year_cross	Publication year	text
	14	record_number_cross	Record number	text

	15	cross_section_01	Section Header: *JBI Critical Appraisal Checklist for Analytical Cross-Sectional Studies*<br>1. Were the criteria for inclusion in the sample clearly defined?	radio (Matrix)<br>1 Yes<br>2 No<br>3 Unclear<br>4 Not Applicable
	16	cross_section_02	2. Were the study subjects and the setting described in detail?	radio (Matrix)<br>1 Yes<br>2 No<br>3 Unclear<br>4 Not Applicable
	17	cross_section_03	3. Was the exposure measured in a valid and reliable way?	radio (Matrix)<br>1 Yes<br>2 No<br>3 Unclear<br>4 Not Applicable
	18	cross_section_04	4. Were objective, standard criteria used for measurement of the condition?	radio (Matrix)<br>1 Yes<br>2 No<br>3 Unclear<br>4 Not Applicable
	19	cross_section_05	5. Were confounding factors identified?	radio (Matrix)<br>1 Yes<br>2 No<br>3 Unclear<br>4 Not Applicable
	20	cross_section_06	6. Were strategies to deal with confounding factors stated?	radio (Matrix)<br>1 Yes<br>2 No<br>3 Unclear<br>4 Not Applicable
	21	cross_section_07	7. Were the outcomes measured in a valid and reliable way?	radio (Matrix)<br>1 Yes<br>2 No<br>3 Unclear<br>4 Not Applicable
	22	cross_section_08	8. Was appropriate statistical analysis used?	radio (Matrix)<br>1 Yes<br>2 No<br>3 Unclear<br>4 Not Applicable
	23	jbi_critical_appraisal_checklist_for_analytical_cr_complete	Section Header: *Form Status*<br>Complete?	dropdown<br>0 Incomplete<br>1 Unverified<br>2 Complete
Instrument: **JBI Critical Appraisal Checklist for Case Control Studies** (jbi_critical_appraisal_checklist_for_case_control) Collapse				
	24	reviewer_name_case	Section Header: *Reviewer Details*<br>Reviewer	dropdown<br>1 Karl Morgan<br>2 James Cowburn<br>3 Matthew Farrow
	25	reviewer_date_case	Date reviewed<br>*Just press 'Today'*	text (date_dmy)
	26	authors_case	Section Header: *Study Information*<br>Authors	text

	27	year_case	Publication year	text
	28	record_number_case	Record number	text
	29	case_control_01	Section Header: *JBI Critical Appraisal Checklist for Case Control Studies*<br>1. Were the groups comparable other than the presence of disease in cases or the absence of disease in controls?	radio (Matrix)<br>1 Yes<br>2 No<br>3 Unclear<br>4 Not Applicable
	30	case_control_02	2. Were cases and controls matched appropriately?	radio (Matrix)<br>1 Yes<br>2 No<br>3 Unclear<br>4 Not Applicable
	31	case_control_03	3. Were the same criteria used for identification of cases and controls?	radio (Matrix)<br>1 Yes<br>2 No<br>3 Unclear<br>4 Not Applicable
	32	case_control_04	4. Was exposure measured in a standard, valid and reliable way?	radio (Matrix)<br>1 Yes<br>2 No<br>3 Unclear<br>4 Not Applicable
	33	case_control_05	5. Was exposure measured in the same way for cases and controls?	radio (Matrix)<br>1 Yes<br>2 No<br>3 Unclear<br>4 Not Applicable
	34	case_control_06	6. Were confounding factors identified?	radio (Matrix)<br>1 Yes<br>2 No<br>3 Unclear<br>4 Not Applicable
	35	case_control_07	7. Were strategies to deal with confounding factors stated?	radio (Matrix)<br>1 Yes<br>2 No<br>3 Unclear<br>4 Not Applicable
	36	case_control_08	8. Were outcomes assessed in a standard, valid and reliable way for cases and controls?	radio (Matrix)<br>1 Yes<br>2 No<br>3 Unclear<br>4 Not Applicable
	37	case_control_09	9. Was the exposure period of interest long enough to be meaningful?	radio (Matrix)<br>1 Yes<br>2 No<br>3 Unclear<br>4 Not Applicable
	38	case_control_10	10. Was appropriate statistical analysis used?	radio (Matrix)<br>1 Yes<br>2 No<br>3 Unclear<br>4 Not Applicable

	39	jbi_critical_appraisal_checklist_for_case_control_complete	Section Header: *Form Status*<br>Complete?	dropdown<br>0 Incomplete<br>1 Unverified<br>2 Complete
Instrument: **JBI Critical Appraisal Checklist for Cohort Studies** (jbi_critical_appraisal_checklist_for_cohort_studie) Collapse				
	40	reviewer_name_cohort	Section Header: *Reviewer Details*<br>Reviewer	dropdown<br>1 Karl Morgan<br>2 James Cowburn<br>3 Matthew Farrow
	41	reviewer_date_cohort	Date reviewed<br>*Just press 'Today'*	text (date_dmy)
	42	authors_cohort	Section Header: *Study Information*<br>Authors<br>*Surname*	text
	43	year_cohort	Publication year<br>*e.g., 2010*	text
	44	record_number_cohort	Record number	text
	45	cohort_01	Section Header: *JBI Critical Appraisal Checklist for Cohort Studies*<br>1. Were the two groups similar and recruited from the same population?	radio (Matrix)<br>1 Yes<br>2 No<br>3 Unclear<br>4 Not Applicable
	46	cohort_02	2. Were the exposures measured similarly to assign people to both exposed and unexposed groups?	radio (Matrix)<br>1 Yes<br>2 No<br>3 Unclear<br>4 Not Applicable
	47	cohort_03	3. Was the exposure measured in a valid and reliable way?	radio (Matrix)<br>1 Yes<br>2 No<br>3 Unclear<br>4 Not Applicable
	48	cohort_04	4. Were confounding factors identified?	radio (Matrix)<br>1 Yes<br>2 No<br>3 Unclear<br>4 Not Applicable
	49	cohort_05	5. Were strategies to deal with confounding factors stated?	radio (Matrix)<br>1 Yes<br>2 No<br>3 Unclear<br>4 Not Applicable
	50	cohort_06	6. Were the groups/participants free of the outcome at the start of the study (or at the moment of exposure)?	radio (Matrix)<br>1 Yes<br>2 No<br>3 Unclear<br>4 Not Applicable
	51	cohort_07	7. Were the outcomes measured in a valid and reliable way?	radio (Matrix)<br>1 Yes<br>2 No<br>3 Unclear<br>4 Not Applicable

	52	cohort_08	8. Was the follow up time reported and sufficient to be long enough for outcomes to occur?	radio (Matrix)<br>1 Yes<br>2 No<br>3 Unclear<br>4 Not Applicable
	53	cohort_09	9. Was follow up complete, and if not, were the reasons to loss to follow up described and explored?	radio (Matrix)<br>1 Yes<br>2 No<br>3 Unclear<br>4 Not Applicable
	54	cohort_10	10. Were strategies to address incomplete follow up utilized?	radio (Matrix)<br>1 Yes<br>2 No<br>3 Unclear<br>4 Not Applicable
	55	cohort_11	11. Was appropriate statistical analysis used?	radio (Matrix)<br>1 Yes<br>2 No<br>3 Unclear<br>4 Not Applicable
	56	jbi_critical_appraisal_checklist_for_cohort_studie_complete	Section Header: *Form Status*<br>Complete?	dropdown<br>0 Incomplete<br>1 Unverified<br>2 Complete
Instrument: **JBI Critical Appraisal Checklist for Studies Reporting Prevalence Data** (jbi_critical_appraisal_checklist_for_studies_repor) Collapse				
	57	reviewer_name_prevalence	Section Header: *Reviewer Details*<br>Reviewer	dropdown<br>1 Karl Morgan<br>2 James Cowburn<br>3 Matthew Farrow
	58	reviewer_date_prevalence	Date reviewed<br>*Just press 'Today'*	text (date_dmy)
	59	authors_prevalence	Section Header: *Study Information*<br>Authors	text
	60	year_prevalence	Publication year	text
	61	record_number_prevalence	Record number	text
	62	prevalence_01	Section Header: *JBI Critical Appraisal Checklist for Studies Reporting Prevalence Data*<br>1. Was the sample frame appropriate to address the target population?	radio (Matrix)<br>1 Yes<br>2 No<br>3 Unclear<br>4 Not Applicable
	63	prevalence_02	2. Were study participants sampled in an appropriate way?	radio (Matrix)<br>1 Yes<br>2 No<br>3 Unclear<br>4 Not Applicable
	64	prevalence_03	3. Was the sample size adequate?	radio (Matrix)<br>1 Yes<br>2 No<br>3 Unclear<br>4 Not Applicable

	65	prevalence_04	4. Were the study subjects and the setting described in detail?	radio (Matrix)<br>1 Yes<br>2 No<br>3 Unclear<br>4 Not Applicable
	66	prevalence_05	5. Was the data analysis conducted with sufficient coverage of the identified sample?	radio (Matrix)<br>1 Yes<br>2 No<br>3 Unclear<br>4 Not Applicable
	67	prevalence_06	6. Were valid methods used for the identification of the condition?	radio (Matrix)<br>1 Yes<br>2 No<br>3 Unclear<br>4 Not Applicable
	68	prevalence_07	7. Was the condition measured in a standard, reliable way for all participants?	radio (Matrix)<br>1 Yes<br>2 No<br>3 Unclear<br>4 Not Applicable
	69	prevalence_08	8. Was there appropriate statistical analysis?	radio (Matrix)<br>1 Yes<br>2 No<br>3 Unclear<br>4 Not Applicable
	70	prevalence_09	9. Was the response rate adequate, and if not, was the low response rate managed appropriately?	radio (Matrix)<br>1 Yes<br>2 No<br>3 Unclear<br>4 Not Applicable
	71	jbi_critical_appraisal_checklist_for_studies_repor_complete	Section Header: *Form Status*<br>Complete?	dropdown<br>0 Incomplete<br>1 Unverified<br>2 Complete
Instrument: **JBI Critical Appraisal Checklist for Quasi-Experimental Studies** (jbi_critical_appraisal_checklist_for_quasiexperime) Collapse				
	72	reviewer_name_quasi	Section Header: *Reviewer Details*<br>Reviewer	dropdown<br>1 Karl Morgan<br>2 James Cowburn<br>3 Matthew Farrow
	73	reviewer_date_quasi	Date reviewed<br>*Just press 'Today'*	text (date_dmy)
	74	author_quasi	Section Header: *Study Information*<br>Authors	text
	75	year_quasi	Publication year	text
	76	record_number_quasi	Record number	text
	77	quasi_experimental_01	Section Header: *JBI Critical Appraisal Checklist for Quasi-Experimental Studies*<br>1. Is it clear in the study what is the 'cause' and what is the 'effect' (i.e. there is no confusion about which variable comes first)?	radio (Matrix)<br>1 Yes<br>2 No<br>3 Unclear<br>4 Not Applicable

	78	quasi_experimental_02	2. Were the participants included in any comparisons similar?	radio (Matrix)<br>1 Yes<br>2 No<br>3 Unclear<br>4 Not Applicable
	79	quasi_experimental_03	3. Were the participants included in any comparisons receiving similar treatment/care, other than the exposure or intervention of interest?	radio (Matrix)<br>1 Yes<br>2 No<br>3 Unclear<br>4 Not Applicable
	80	quasi_experimental_04	4. Was there a control group?	radio (Matrix)<br>1 Yes<br>2 No<br>3 Unclear<br>4 Not Applicable
	81	quasi_experimental_05	5. Were there multiple measurements of the outcome both pre and post the intervention/exposure?	radio (Matrix)<br>1 Yes<br>2 No<br>3 Unclear<br>4 Not Applicable
	82	quasi_experimental_06	6. Was follow up complete and if not, were differences between groups in terms of their follow up adequately described and analyzed?	radio (Matrix)<br>1 Yes<br>2 No<br>3 Unclear<br>4 Not Applicable
	83	quasi_experimental_07	7. Were the outcomes of participants included in any comparisons measured in the same way?	radio (Matrix)<br>1 Yes<br>2 No<br>3 Unclear<br>4 Not Applicable
	84	quasi_experimental_08	8. Were outcomes measured in a reliable way?	radio (Matrix)<br>1 Yes<br>2 No<br>3 Unclear<br>4 Not Applicable
	85	quasi_experimental_09	9. Was appropriate statistical analysis used?	radio (Matrix)<br>1 Yes<br>2 No<br>3 Unclear<br>4 Not Applicable
	86	jbi_critical_appraisal_checklist_for_quasiexperime_complete	Section Header: *Form Status*<br>Complete?	dropdown<br>0 Incomplete<br>1 Unverified<br>2 Complete

Instrument: **JBI Critical Appraisal Checklist for Randomised Controlled Trials** (jbi_critical_appraisal_checklist_for_randomised_co)

Collapse

	87	reviewer_name_rct	Section Header: *Reviewer Details*<br>Reviewer	dropdown<br>1 Karl Morgan<br>2 James Cowburn<br>3 Matthew Farrow
	88	reviewer_date_rct	Date reviewed<br>*Just press 'Today'*	text (date_dmy)

	89	authors_rct	Section Header: *Study Information*<br>Authors	text
	90	year_rct	Publication year	text
	91	record_number_rct	Record number	text
	92	rct_01	Section Header: *JBI Critical Appraisal Checklist for Randomised Controlled Trials*<br>1. Was true randomization used for assignment of participants to treatment groups?	radio (Matrix)<br>1 Yes<br>2 No<br>3 Unclear<br>4 Not Applicable
	93	rct_02	2. Was allocation to treatment groups concealed?	radio (Matrix)<br>1 Yes<br>2 No<br>3 Unclear<br>4 Not Applicable
	94	rct_03	3. Were treatment groups similar at the baseline?	radio (Matrix)<br>1 Yes<br>2 No<br>3 Unclear<br>4 Not Applicable
	95	rct_04	4. Were participants blind to treatment assignment?	radio (Matrix)<br>1 Yes<br>2 No<br>3 Unclear<br>4 Not Applicable
	96	rct_05	5. Were those delivering treatment blind to treatment assignment?	radio (Matrix)<br>1 Yes<br>2 No<br>3 Unclear<br>4 Not Applicable
	97	rct_06	6. Were outcomes assessors blind to treatment assignment?	radio (Matrix)<br>1 Yes<br>2 No<br>3 Unclear<br>4 Not Applicable
	98	rct_07	7. Were treatment groups treated identically other than the intervention of interest?	radio (Matrix)<br>1 Yes<br>2 No<br>3 Unclear<br>4 Not Applicable
	99	rct_08	8. Was follow up complete and if not, were differences between groups in terms of their follow up adequately described and analyzed?	radio (Matrix)<br>1 Yes<br>2 No<br>3 Unclear<br>4 Not Applicable
	100	rct_09	9. Were participants analyzed in the groups to which they were randomized?	radio (Matrix)<br>1 Yes<br>2 No<br>3 Unclear<br>4 Not Applicable

#	Variable / Field Name	Field Label	Field Attributes
101	rct_10	10. Were outcomes measured in the same way for treatment groups?	radio (Matrix)<br>1 Yes<br>2 No<br>3 Unclear<br>4 Not Applicable
102	rct_11	11. Were outcomes measured in a reliable way?	radio (Matrix)<br>1 Yes<br>2 No<br>3 Unclear<br>4 Not Applicable
103	rct_12	12. Was appropriate statistical analysis used?	radio (Matrix)<br>1 Yes<br>2 No<br>3 Unclear<br>4 Not Applicable
104	rct_13	13. Was the trial design appropriate, and any deviations from the standard RCT design (individual randomization, parallel groups) accounted for in the conduct and analysis of the trial?	radio (Matrix)<br>1 Yes<br>2 No<br>3 Unclear<br>4 Not Applicable
105	jbi_critical_appraisal_checklist_for_randomised_co_complete	Section Header: *Form Status*<br>Complete?	dropdown<br>0 Incomplete<br>1 Unverified<br>2 Complete
Instrument: **JBI Critical Appraisal Checklist for Systematic Reviews and Research Syntheses** (jbi_critical_appraisal_checklist_for_systematic_re) Collapse			
106	reviewer_name_review	Section Header: *Study Information*<br>Reviewer	dropdown<br>1 Karl Morgan<br>2 James Cowburn<br>3 Matthew Farrow
107	reviewer_date_review	Date reviewed<br>*Just press 'Today'*	text (date_dmy)
108	authors_review	Section Header: *Study Information*<br>Authors	text
109	year_review	Publication year	text
110	record_number_review	Record number	text
111	reviews_01	Section Header: *JBI Critical Appraisal Checklist for Systematic Reviews and Research Syntheses*<br>1. Is the review question clearly and explicitly stated?	radio (Matrix)<br>1 Yes<br>2 No<br>3 Unclear<br>4 Not Applicable
112	reviews_02	2. Were the inclusion criteria appropriate for the review question?	radio (Matrix)<br>1 Yes<br>2 No<br>3 Unclear<br>4 Not Applicable
113	reviews_03	3. Was the search strategy appropriate?	radio (Matrix)<br>1 Yes<br>2 No<br>3 Unclear<br>4 Not Applicable

	114	reviews_04	4. Were the sources and resources used to search for studies adequate?	radio (Matrix)<br>1 Yes<br>2 No<br>3 Unclear<br>4 Not Applicable
	115	reviews_05	5. Were the criteria for appraising studies appropriate?	radio (Matrix)<br>1 Yes<br>2 No<br>3 Unclear<br>4 Not Applicable
	116	reviews_06	6. Was critical appraisal conducted by two or more reviewers independently?	radio (Matrix)<br>1 Yes<br>2 No<br>3 Unclear<br>4 Not Applicable
	117	reviews_07	7. Were there methods to minimize errors in data extraction?	radio (Matrix)<br>1 Yes<br>2 No<br>3 Unclear<br>4 Not Applicable
	118	reviews_08	8. Were the methods used to combine studies appropriate?	radio (Matrix)<br>1 Yes<br>2 No<br>3 Unclear<br>4 Not Applicable
	119	reviews_09	9. Was the likelihood of publication bias assessed?	radio (Matrix)<br>1 Yes<br>2 No<br>3 Unclear<br>4 Not Applicable
	120	reviews_10	10. Were recommendations for policy and/or practice supported by the reported data?	radio (Matrix)<br>1 Yes<br>2 No<br>3 Unclear<br>4 Not Applicable
	121	reviews_11	11. Were the specific directives for new research appropriate?	radio (Matrix)<br>1 Yes<br>2 No<br>3 Unclear<br>4 Not Applicable
	122	jbi_critical_appraisal_checklist_for_systematic_re_complete	Section Header: *Form Status*<br>Complete?	dropdown<br>0 Incomplete<br>1 Unverified<br>2 Complete

Supplementary Material 7. Draft Data Charting Template

Data Charting

Codebook

Data Dictionary Codebook

2022-08-02 19:49:46

Collapse all instruments

#	Variable / Field Name	Field Label<br>*Field Note*	Field Attributes (Field Type, Validation, Choices, Calculations, etc.)
Instrument: **Data Extraction** (data_extraction)			Collapse
1	record_id	Record ID	text
2	reviewer_name_extraction	Reviewer	dropdown, Required<br>1 Karl Morgan<br>2 James Cowburn<br>3 Mathew Farrow
3	reviewer_date_extraction	Date of extraction<br>*Just press 'Today'*	text (date_dmy), Required
4	record_number_extraction	Section Header: *Study information*<br>Record number	text, Required
5	first_author	First author<br>*Format as: second name, first name initial (e.g., Morgan, K.).*	text, Required
6	co_authors	Co-authors<br>*Format as: Initial followed by surname (e.g., Morgan, K.). Write n/a if none.*	notes, Required
7	publication_year	Publication year<br>*Please insert as number (e.g., 2006).*	text (number), Required
8	country_research_origin	Country of research origin<br><br>ISO Alpha 3 codes<br>*Please use ISO Alpha 3 codes (e.g., USA, GBR, FRA, DNK, CAN). Write unclear if unknown.*	text, Required
9	study_aim_1	Section Header: *Study aims*<br>Study aim 1<br>*Write unclear if unknown.*	notes, Required
10	study_aim_2	Study aim 2<br>*Write unclear if unknown. If no secondary aim write n/a.*	notes, Required
11	study_aim_3	Study aim 3<br>*Write unclear if unknown. If no tertiary aim write n/a.*	notes, Required
12	study_design	Section Header: *Methodology - Study Design*<br>Study design	radio, Required<br>1 Analytical cross-sectional<br>2 Case Control<br>3 Cohort<br>4 Prevalence data<br>5 Quasi-experimental<br>6 Randomised control trial<br>7 Review and research syntheses<br>8 Unclear
13	number_of_participants	Section Header: *Methodology - Experimental Population Size*<br>Total number of participants<br>*Report as: number*	text (number), Required
14	number_of_males	Number of males<br>*Report as: number*	text, Required
15	number_of_females	Number of females<br>*Report as: number*	text, Required
16	age_heading	Section Header: *Methodology - Experimental Group Demographic Information*<br>Experimental Group Age	descriptive
17	total_population_age	Total Population	descriptive

	18	participant_age_total	How was the age of the total study population reported?<br>*Select all relevant answers*	checkbox, Required<br>1 participant_age_total___1 Mean<br>2 participant_age_total___2 Standard deviation<br>3 participant_age_total___3 Median<br>4 participant_age_total___4 Range
	19	age_mean<br>Show the field ONLY if:<br>[participant_age_total(1)] = '1'	Mean<br>*Years*	text, Required
	20	age_stdev<br>Show the field ONLY if:<br>[participant_age_total(2)] = '1'	Standard deviation<br>*Years*	text, Required
	21	age_median<br>Show the field ONLY if:<br>[participant_age_total(3)] = '1'	Median<br>*Years*	text, Required
	22	age_range<br>Show the field ONLY if:<br>[participant_age_total(4)] = '1'	Range<br>*Years*	text, Required
	23	male_population_age	Male Population	descriptive
	24	participant_age_male	How was the age of the male study population reported?<br>*Select all relevant answers*	checkbox, Required<br>1 participant_age_male___1 Mean<br>2 participant_age_male___2 Standard deviation<br>3 participant_age_male___3 Median<br>4 participant_age_male___4 Range
	25	age_mean_male<br>Show the field ONLY if:<br>[participant_age_male(1)] = '1'	Mean<br>*Years*	text, Required
	26	age_stdev_male<br>Show the field ONLY if:<br>[participant_age_male(2)] = '1'	Standard deviation<br>*Years*	text, Required
	27	age_median_male<br>Show the field ONLY if:<br>[participant_age_male(3)] = '1'	Median<br>*Years*	text, Required
	28	age_range_male<br>Show the field ONLY if:<br>[participant_age_male(4)] = '1'	Range<br>*Years*	text, Required
	29	population_age_female	Female Population	descriptive
	30	participant_age_female	How was the age of the female study population reported?<br>*Select all relevant answers*	checkbox, Required<br>1 participant_age_female___1 Mean<br>2 participant_age_female___2 Standard deviation<br>3 participant_age_female___3 Median<br>4 participant_age_female___4 Range
	31	age_mean_female<br>Show the field ONLY if:<br>[participant_age_female(1)] = '1'	Mean<br>*Years*	text, Required
	32	age_stdev_female<br>Show the field ONLY if:<br>[participant_age_female(2)] = '1'	Standard deviation<br>*Years*	text, Required
	33	age_median_female<br>Show the field ONLY if:<br>[participant_age_female(3)] = '1'	Median<br>*Years*	text, Required
	34	age_range_female<br>Show the field ONLY if:<br>[participant_age_female(4)] = '1'	Range<br>*Years*	text, Required
	35	participant_anth_info	Participant Anthropometric Information	descriptive

	36	total_population_anthro	Total Population	descriptive
	37	participant_height	How was participant height reported?<br>*Meters*	checkbox, Required<br>1 participant_height___1 Mean<br>2 participant_height___2 Standard deviation<br>3 participant_height___3 Median<br>4 participant_height___4 Range
	38	participant_height_mean<br>Show the field ONLY if:<br>[participant_height(1)] = '1'	Mean	text, Required
	39	participant_height_stdev<br>Show the field ONLY if:<br>[participant_height(2)] = '1'	Standard deviation	text, Required
	40	participant_height_median<br>Show the field ONLY if:<br>[participant_height(3)] = '1'	Median	text, Required
	41	participant_height_range<br>Show the field ONLY if:<br>[participant_height(4)] = '1'	Range	text, Required
	42	participant_mass	Participant mass<br>*Kilograms*	checkbox, Required<br>1 participant_mass___1 Mean<br>2 participant_mass___2 Standard deviation<br>3 participant_mass___3 Median<br>4 participant_mass___4 Range
	43	participant_mass_mean<br>Show the field ONLY if:<br>[participant_mass(1)] = '1'	Mean	text, Required
	44	participant_mass_stdev<br>Show the field ONLY if:<br>[participant_mass(2)] = '1'	Standard deviation	text, Required
	45	participant_mass_median<br>Show the field ONLY if:<br>[participant_mass(3)] = '1'	Median	text, Required
	46	participant_mass_range<br>Show the field ONLY if:<br>[participant_mass(4)] = '1'	Range	text, Required
	47	participant_bmi	Participant BMI<br>*Kilograms/meters^2*	checkbox, Required<br>1 participant_bmi___1 Mean<br>2 participant_bmi___2 Standard deviation<br>3 participant_bmi___3 Median<br>4 participant_bmi___4 Range
	48	participant_bmi_mean<br>Show the field ONLY if:<br>[participant_bmi(1)] = '1'	Mean	text, Required
	49	participant_bmi_stdev<br>Show the field ONLY if:<br>[participant_bmi(2)] = '1'	Standard deviation	text, Required
	50	participant_bmi_median<br>Show the field ONLY if:<br>[participant_bmi(3)] = '1'	Median	text, Required
	51	participant_bmi_range<br>Show the field ONLY if:<br>[participant_bmi(4)] = '1'	Range	text, Required
	52	male_population_anthro	Male Population	descriptive

	53	male_exp_pp_height	How was participant height reported?<br>*Meters*	checkbox, Required<br>1 male_exp_pp_height___1 Mean<br>2 male_exp_pp_height___2 Standard deviation<br>3 male_exp_pp_height___3 Median<br>4 male_exp_pp_height___4 Range
	54	male_exp_mean_height<br>Show the field ONLY if:<br>[male_exp_pp_height(1)] = '1'	Mean	text, Required
	55	male_exp_stdev_height<br>Show the field ONLY if:<br>[male_exp_pp_height(2)] = '1'	Standard deviation	text, Required
	56	male_exp_median_height<br>Show the field ONLY if:<br>[male_exp_pp_height(3)] = '1'	Median	text, Required
	57	male_exp_range_height<br>Show the field ONLY if:<br>[male_exp_pp_height(4)] = '1'	Range	text, Required
	58	male_exp_pp_mass	How was participant mass reported?<br>*Meters*	checkbox, Required<br>1 male_exp_pp_mass___1 Mean<br>2 male_exp_pp_mass___2 Standard deviation<br>3 male_exp_pp_mass___3 Median<br>4 male_exp_pp_mass___4 Range
	59	male_exp_mean_mass<br>Show the field ONLY if:<br>[male_exp_pp_mass(1)] = '1'	Mean	text, Required
	60	male_exp_stdev_mass<br>Show the field ONLY if:<br>[male_exp_pp_mass(2)] = '1'	Standard deviation	text, Required
	61	male_exp_median_mass<br>Show the field ONLY if:<br>[male_exp_pp_mass(3)] = '1'	Median	text, Required
	62	male_exp_range_mass<br>Show the field ONLY if:<br>[male_exp_pp_mass(4)] = '1'	Range	text, Required
	63	male_exp_pp_bmi	How was participant body mass index reported?<br>*Meters*	checkbox, Required<br>1 male_exp_pp_bmi___1 Mean<br>2 male_exp_pp_bmi___2 Standard deviation<br>3 male_exp_pp_bmi___3 Median<br>4 male_exp_pp_bmi___4 Range
	64	male_exp_mean_bmi<br>Show the field ONLY if:<br>[male_exp_pp_bmi(1)] = '1'	Mean	text, Required
	65	male_exp_stdev_bmi<br>Show the field ONLY if:<br>[male_exp_pp_bmi(2)] = '1'	Standard deviation	text, Required
	66	male_exp_median_bmi<br>Show the field ONLY if:<br>[male_exp_pp_bmi(3)] = '1'	Median	text, Required
	67	male_exp_range_bmi<br>Show the field ONLY if:<br>[male_exp_pp_bmi(4)] = '1'	Range	text, Required
	68	female_population_anthro	Female Population	descriptive
	69	female_exp_pp_height	How was participant height reported?<br>*Meters*	checkbox, Required<br>1 female_exp_pp_height___1 Mean<br>2 female_exp_pp_height___2 Standard deviation<br>3 female_exp_pp_height___3 Median<br>4 female_exp_pp_height___4 Range

	70	female_exp_mean_height<br>Show the field ONLY if: [female_exp_pp_height(1)] = '1'	Mean	text, Required
	71	female_exp_sdtev_height<br>Show the field ONLY if: [female_exp_pp_height(2)] = '1'	Standard deviation	text, Required
	72	female_exp_median_height<br>Show the field ONLY if: [female_exp_pp_height(3)] = '1'	Median	text, Required
	73	female_exp_range_height<br>Show the field ONLY if: [female_exp_pp_height(4)] = '1'	Range	text, Required
	74	female_exp_pp_mass	How was participant mass reported?<br>*Meters*	checkbox, Required<br>1 female_exp_pp_mass___1 Mean<br>2 female_exp_pp_mass___2 Standard deviation<br>3 female_exp_pp_mass___3 Median<br>4 female_exp_pp_mass___4 Range
	75	female_exp_mean_mass<br>Show the field ONLY if: [female_exp_pp_mass(1)] = '1'	Mean	text, Required
	76	female_exp_stdev_mass<br>Show the field ONLY if: [female_exp_pp_mass(2)] = '1'	Standard deviation	text, Required
	77	female_exp_median_mass<br>Show the field ONLY if: [female_exp_pp_mass(3)] = '1'	Median	text, Required
	78	female_exp_range_mass<br>Show the field ONLY if: [female_exp_pp_mass(4)] = '1'	Range	text, Required
	79	female_exp_pp_bmi	How was participant body mass index reported?<br>*Meters*	checkbox, Required<br>1 female_exp_pp_bmi___1 Mean<br>2 female_exp_pp_bmi___2 Standard deviation<br>3 female_exp_pp_bmi___3 Median<br>4 female_exp_pp_bmi___4 Range
	80	female_exp_mean_bmi<br>Show the field ONLY if: [female_exp_pp_bmi(1)] = '1'	Mean	text, Required
	81	female_exp_stdev_bmi<br>Show the field ONLY if: [female_exp_pp_bmi(2)] = '1'	Standard deviation	text, Required
	82	female_exp_median_bmi<br>Show the field ONLY if: [female_exp_pp_bmi(3)] = '1'	Median	text, Required
	83	female_exp_range_bmi<br>Show the field ONLY if: [female_exp_pp_bmi(4)] = '1'	Range	text, Required
	84	number_of_controls<br>Show the field ONLY if: [cntrl_participants_included] = 1	Section Header: *Methodology - Control Population Size*<br>Number of controls<br>*Report as: number*	text (number), Required
	85	number_of_males_control<br>Show the field ONLY if: [cntrl_participants_included] = 1	Number of control males<br>*Report as: number*	text, Required

	86	number_of_females_control<br>Show the field ONLY if:<br>[number_of_controls] = 1	Number of control females<br>*Report as: number*	text, Required
	87	cntrl_group_age	Section Header: *Methodology - Control Demographic Information*<br>Control Group Age	descriptive
	88	total_cntrl_age	Total Population	descriptive
	89	participant_cntrl_age_type<br>Show the field ONLY if:<br>[number_of_controls] = 1	How was the age of control participants reported?<br>*Select all relevant answers*	checkbox, Required<br>1 participant_cntrl_age_type___1 Mean<br>2 participant_cntrl_age_type___2 Standard deviation<br>3 participant_cntrl_age_type___3 Median<br>4 participant_cntrl_age_type___4 Range
	90	age_cntrl_mean<br>Show the field ONLY if:<br>[participant_cntrl_age_type(1)] = '1'	Mean	text, Required
	91	age_cntrl_stdev<br>Show the field ONLY if:<br>[participant_cntrl_age_type(2)] = '1'	Standard deviation	text, Required
	92	age_cntrl_median<br>Show the field ONLY if:<br>[participant_cntrl_age_type(3)] = '1'	Median	text, Required
	93	age_cntrl_range<br>Show the field ONLY if:<br>[participant_cntrl_age_type(4)] = '1'	Range	text, Required
	94	male_cntrl_age	Male Population	descriptive
	95	male_pp_cntrl_age_type	How was the age of control participants reported?<br>*Select all relevant answers*	checkbox, Required<br>1 male_pp_cntrl_age_type___1 Mean<br>2 male_pp_cntrl_age_type___2 Standard deviation<br>3 male_pp_cntrl_age_type___3 Median<br>4 male_pp_cntrl_age_type___4 Range
	96	age_cntrl_male_mean<br>Show the field ONLY if:<br>[male_pp_cntrl_age_type(1)] = '1'	Mean	text
	97	age_cntrl_male_stdev<br>Show the field ONLY if:<br>[male_pp_cntrl_age_type(2)] = '1'	Standard deviation	text
	98	age_cntrl_male_median<br>Show the field ONLY if:<br>[male_pp_cntrl_age_type(3)] = '1'	Median	text
	99	age_cntrl_male_range<br>Show the field ONLY if:<br>[male_pp_cntrl_age_type(4)] = '1'	Range	text
	100	female_cntrl_age	Female Population	descriptive
	101	female_pp_cntrl_age_type	How was the age of control participants reported?<br>*Select all relevant answers*	checkbox, Required<br>1 female_pp_cntrl_age_type___1 Mean<br>2 female_pp_cntrl_age_type___2 Standard deviation<br>3 female_pp_cntrl_age_type___3 Median<br>4 female_pp_cntrl_age_type___4 Range

	102	age_cntrl_female_mean<br>Show the field ONLY if:<br>[female_pp_cntrl_age_type(1)] = '1'	Mean	text
	103	age_cntrl_female_stdev<br>Show the field ONLY if:<br>[female_pp_cntrl_age_type(2)] = '1'	Standard deviation	text
	104	age_cntrl_female_median<br>Show the field ONLY if:<br>[female_pp_cntrl_age_type(3)] = '1'	Median	text
	105	age_cntrl_female_range<br>Show the field ONLY if:<br>[female_pp_cntrl_age_type(4)] = '1'	Range	text
	106	cntrl_anth_info	Control Anthropometric Information	descriptive
	107	cntrl_anth_info_2	Total Population	descriptive
	108	control_height<br>Show the field ONLY if:<br>[number_of_controls] = 1	Control height<br>*Meters*	text, Required
	109	control_mass<br>Show the field ONLY if:<br>[number_of_controls] = 1	Control mass<br>*Kilograms*	text, Required
	110	control_bmi<br>Show the field ONLY if:<br>[number_of_controls] = 1	Control body mass index<br>*Kilograms/meters^2*	text, Required
	111	male_cntrl_anth_info	Male Population	descriptive
	112	female_cntrl_anth_info	Female Population	descriptive
	113	injury_type	Section Header: *Injury History*<br>Type of knee injury<br>*Select all relevant answers*	checkbox, Required<br>1 | injury_type___1 | Intra-articular (general unspecified)<br>2 | injury_type___2 | Ligament<br>3 | injury_type___3 | Meniscus<br>4 | injury_type___4 | Fracture<br>5 | injury_type___5 | Dislocation<br>6 | injury_type___6 | Contusion<br>7 | injury_type___7 | Unclear
	114	time_since_injury_type	How was time between injury incidence and observation/follow up reported?<br>*Select all relevant answers*	checkbox, Required<br>1 | time_since_injury_type___1 | Mean<br>2 | time_since_injury_type___2 | Standard deviation<br>3 | time_since_injury_type___3 | Median<br>4 | time_since_injury_type___4 | Range<br>5 | time_since_injury_type___5 | Not reported
	115	time_since_injury_mean<br>Show the field ONLY if:<br>[time_since_injury_type(1)] = '1'	Mean	text (number), Required
	116	time_since_injury_stdev<br>Show the field ONLY if:<br>[time_since_injury_type(2)] = '1'	Standard deviation	text (number), Required
	117	time_since_injury_median<br>Show the field ONLY if:<br>[time_since_injury_type(3)] = '1'	Median	text (number), Required

	118	time_since_injury_range<br>Show the field ONLY if: [time_since_injury_type(4)] = '1'	Range	text (number), Required
	119	knee_surgery	Section Header: *Surgery History*<br>Did the participants have knee surgery?	yesno, Required<br>1 Yes<br>0 No
	120	surgery_type<br>Show the field ONLY if: [knee_surgery] = '1'	Type of knee surgery<br>*Select all relevant answers*	checkbox, Required<br>1 surgery_type___1 Tendon graft<br>2 surgery_type___2 Meniscectomy<br>3 surgery_type___3 Chondroplasty<br>7 surgery_type___7 Unclear
	121	time_since_surgery_type<br>Show the field ONLY if: [knee_surgery] = '1'	How was time between between surgery and observation/follow up reported?<br>*Select all relevant answers*	checkbox, Required<br>1 time_since_surgery_type___1 Mean<br>2 time_since_surgery_type___2 Standard deviation<br>3 time_since_surgery_type___3 Median<br>4 time_since_surgery_type___4 Range<br>5 time_since_surgery_type___5 Not reported
	122	time_since_surgery_mean<br>Show the field ONLY if: [time_since_surgery_type(1)] = 1	Mean	text, Required
	123	time_since_surgery_stdev<br>Show the field ONLY if: [time_since_surgery_type(2)] = 1	Standard deviation	text, Required
	124	time_since_surgery_median<br>Show the field ONLY if: [time_since_surgery_type(3)] = 1	Median	text, Required
	125	time_since_surgery_range<br>Show the field ONLY if: [time_since_surgery_type(4)] = 1	Range	text, Required
	126	sport_type	Section Header: *Sporting History*<br>What sport did the participants engage in at the time of injury?<br>*If not detailed state N/A*	text, Required
	127	sport_level	What was the competition level at the time of injury?<br>*If not detailed state N/A*	text, Required
	128	was_there_intervention	Section Header: *Intervention Details*<br>Was there an intervention?	yesno<br>1 Yes<br>0 No
	129	intervention_type<br>Show the field ONLY if: [was_there_intervention] = 1	Intervention type<br>*Please detail the type of intervention*	notes, Required
	130	intervention_setting<br>Show the field ONLY if: [was_there_intervention] = 1	Intervention setting<br>*E.g., home, hospital, physio clinic*	text, Required
	131	duration_of_intervention<br>Show the field ONLY if: [was_there_intervention] = 1	Duration of intervention<br>*Months*	text (number), Required
	132	adherence<br>Show the field ONLY if: [was_there_intervention] = 1	Adherence rate	text (number), Required
	133	drop_out<br>Show the field ONLY if: [was_there_intervention] = 1	Drop out rate	text (number), Required

	134	independent_variable	Independent Variables	descriptive
	135	what_did_the_study_measure	What was the independent variable measuring?<br>*Select all relevant answers*	checkbox, Required<br>1 what_did_the_study_measure___1 Physical activity<br>2 what_did_the_study_measure___2 Systemic inflammation<br>3 what_did_the_study_measure___3 Knee joint loading<br>4 what_did_the_study_measure___4 Adiposity<br>5 what_did_the_study_measure___5 Muscle size<br>6 what_did_the_study_measure___6 Muscle strength<br>7 what_did_the_study_measure___7 Intramuscular fat<br>8 what_did_the_study_measure___8 Bone mineral density<br>9 what_did_the_study_measure___9 Unclear
	136	physical_activity_tool<br>Show the field ONLY if:<br>[what_did_the_study_measure(1)] = 1	Please select the tool used to measure physical activity<br>*Select all relevant answers*	checkbox, Required<br>1 physical_activity_tool___1 Doubly labelled water<br>2 physical_activity_tool___2 Accelerometers<br>3 physical_activity_tool___3 Pedometers<br>4 physical_activity_tool___4 Subjective questionnaires<br>5 physical_activity_tool___5 Diaries
	137	subjective_pa_tool_type<br>Show the field ONLY if:<br>[physical_activity_tool(4)] = '1'	What was the name of the subjective assessment tool?<br>*Please use full name, not abbreviation. E.g., International Physical Activity Questionnaire*	text, Required
	138	inflammation_tool<br>Show the field ONLY if:<br>[what_did_the_study_measure(2)] = '1'	Which cytokine/protein(s) were used to measure systemic inflammation?<br>*Select all relevant answers*	checkbox, Required<br>1 inflammation_tool___1 Interleukin 6 (IL-6)<br>2 inflammation_tool___2 Interleukin 1 Beta (IL-1β)<br>3 inflammation_tool___3 Tumor Necrosis Factor Alpha (TNF-α)<br>6 inflammation_tool___6 C-Reactive Protein (CRP)
	139	knee_load_tool<br>Show the field ONLY if:<br>[what_did_the_study_measure(3)] = '1'	What was the tool(s) used to measure knee joint loading?<br>*Select all relevant answers*	checkbox, Required<br>1 knee_load_tool___1 Ground reaction force<br>2 knee_load_tool___2 Moments<br>3 knee_load_tool___3 Inverse dynamics<br>4 knee_load_tool___4 Forward dynamics
	140	adiposity_tool<br>Show the field ONLY if:<br>[what_did_the_study_measure(4)] = '1'	What was the tool to measure adiposity?	checkbox, Required<br>1 adiposity_tool___1 Body Mass Index (BMI)<br>2 adiposity_tool___2 Air displacement plethysmograph (ADP)<br>3 adiposity_tool___3 Bioelectrical impedance (BI)<br>4 adiposity_tool___4 Dual energy x-ray absorptiometry (DEXA or DXA)<br>5 adiposity_tool___5 Computerised tomography (CT)<br>6 adiposity_tool___6 Peripheral computerised tomography (pQCT)

	141	muslce_tool	What was the tool used to measure muscle size?	checkbox<br>1 | muslce_tool___1 | Ultrasound (US)<br>2 | muslce_tool___2 | Dual energy x-ray absorptiometry (DEXA or DXA)<br>3 | muslce_tool___3 | Peripheral quantitative computerised tomography (pQCT)<br>4 | muslce_tool___4 | Computerised tomography (CT)<br>5 | muslce_tool___5 | Magnetic resonance imaging (MRI)
	142	muscle_strength_tool<br>Show the field ONLY if:<br>[what_did_the_study_measure(5)] = '1'	What was the tool to measure muscle strength?	checkbox, Required<br>1 | muscle_strength_tool___1 | Isokinetic dynamometry<br>2 | muscle_strength_tool___2 | Hand-held dynamometer
	143	intra_muscular_fat_tool<br>Show the field ONLY if:<br>[what_did_the_study_measure(6)] = '1'	What was the tool to measure intra-muscular fat?	checkbox, Required<br>1 | intra_muscular_fat_tool___1 | Ultrasound (US)<br>2 | intra_muscular_fat_tool___2 | Computerised tomography (CT)<br>3 | intra_muscular_fat_tool___3 | Peripheral quantitative computerised tomography (pQCT)<br>4 | intra_muscular_fat_tool___4 | Magnetic resonance imaging (MRI)
	144	bone_mineral_density_tool<br>Show the field ONLY if:<br>[what_did_the_study_measure(7)] = '1'	What was the tool to measure bone mineral density?	checkbox, Required<br>1 | bone_mineral_density_tool___1 | Dual energy x-ray absorptiometry (DEXA or DXA)<br>2 | bone_mineral_density_tool___2 | Computerised tomography (CT)<br>3 | bone_mineral_density_tool___3 | Peripheral quantitative computerised tomography (pQCT)
	145	ptoa_disease_head	Section Header: *Outcome Variables*<br>PTOA Disease	descriptive
	146	ptoa_disease	Does the study measure PTOA disease?	yesno<br>1 | Yes<br>0 | No
	147	radiography_diagnostic	Radiography diagnostic system	checkbox, Required<br>1 | radiography_diagnostic___1 | Kellgren and Lawrence (K/L) system<br>2 | radiography_diagnostic___2 | Osteoarthritis Research Society International (OARSI) Atlas criteria
	148	mri_diagnostic	Magnetic resonance imaging diagnostic system	checkbox, Required<br>1 | mri_diagnostic___1 | MRI Osteoarthritis Knee Score (MOAKS)<br>2 | mri_diagnostic___2 | Whole Organ Magnetic Resonance Imaging Score (WORMS)<br>3 | mri_diagnostic___3 | Boston Leeds OA Knee Score (BLOKS)

	149	indicators_of_disease	Indicators of structural deterioration preceding or exacerbating PTOA disease	checkbox, Required<br>1 indicators_of_disease___1 MRI quantified cartilage thickness and volume<br>2 indicators_of_disease___2 T1rho mapping<br>3 indicators_of_disease___3 T2 mapping<br>4 indicators_of_disease___4 Delayed contrast-enhanced MRI of cartilage (dGEMRIC)
	150	systemic_biomarkers	Systemic biomarkers indicating structural deterioration of knee PTOA disease	checkbox<br>1 systemic_biomarkers___1 Matrix Metalloprotease 3 (MMP-3)<br>2 systemic_biomarkers___2 Matrix Metalloprotease 13 (MMP-13)<br>3 systemic_biomarkers___3 N-Propeptide of Collagen IIA (PIIANP)<br>4 systemic_biomarkers___4 C-Telopeptide of Type II Collagen (CTX)<br>5 systemic_biomarkers___5 C-Propeptide of Type II Collagen (CP-II)<br>6 systemic_biomarkers___6 CTX:CP-II<br>7 systemic_biomarkers___7 Collagen Type II Cleavage Product (C2C)<br>8 systemic_biomarkers___8 Col2-3/4 C-terminal cleavage product of types I and II collagen (C1,2C)<br>9 systemic_biomarkers___9 Hyaluronic Acid (HA)<br>10 systemic_biomarkers___10 Chondroitin Sulphate 846 Epitope (CS846)<br>11 systemic_biomarkers___11 Aggrecan164 or Aggrecan Fragments (ARGS)<br>12 systemic_biomarkers___12 Cartilage Oligomeric Matrix Protein (COMP)
	151	illness_diagnostic_system	Diagnosis of knee PTOA illness	checkbox<br>1 illness_diagnostic_system___1 Van Luyten Criteria
	152	independent_variablertgrt	PTOA Illness	descriptive
	153	ptoa_illness	Did the study measure PTOA illness?	yesno<br>1 Yes<br>0 No
	154	illness_measure_type	What type of aspect of illness did they measure?	checkbox<br>1 illness_measure_type___1 Combined pain/function<br>2 illness_measure_type___2 Pain<br>3 illness_measure_type___3 Other symptomology (swelling, stiffness, crepitus)<br>4 illness_measure_type___4 Function/disability<br>5 illness_measure_type___5 Quality of Life

	155	combined_pain_function	Please report the combined pain/function scale(s) used	checkbox<br>1 combined_pain_function___1 International Knee Documentation Committee (IKDC)<br>2 combined_pain_function___2 Oxford Knee Score (OKS)<br>3 combined_pain_function___3 Knee Outcome Survey Activities of Daily Living (KOS-ADL)<br>4 combined_pain_function___4 Western Ontario and McMaster Universities Osteoarthritis Index (WOMAC)<br>5 combined_pain_function___5 Lysholm Knee Scoring Scale
	156	pain	Please report the combined pain/function scale(s) used	checkbox<br>1 pain___1 Knee Injury and Osteoarthritis Outcome Score (KOOS)<br>2 pain___2 Visual Analogue Scale (VAS)<br>3 pain___3 Numerical Rating Scale (NRS)<br>4 pain___4 Verbal Rating Scale (VRS)<br>5 pain___5 McGill Pain Questionnaire (MPQ)<br>6 pain___6 Short-Form McGill Pain Questionnaire (SF-MPQ)<br>7 pain___7 Chronic Pain Grade Scale (CPGS)<br>8 pain___8 Measure of Intermittent and Constant Osteoarthritis Pain (ICOAP)<br>9 pain___9 Brief Pain Inventory Short Form (BPI-SF)<br>10 pain___10 West Haven-Yale Multidimensional Pain Inventory (WHYMPI)<br>11 pain___11 Treatment Outcomes of Pain Survey (TOPS)
	157	illness_indicators	Indicators of knee PTOA illness	checkbox<br>1 illness_indicators___1 Knee Injury and Osteoarthritis Outcome Score (KOOS)<br>2 illness_indicators___2 Western Ontario and McMaster Universities Osteoarthritis Index (WOMAC)<br>3 illness_indicators___3 International Knee Documentation Committee (IKDC)<br>4 illness_indicators___4 Oxford Knee Score (OKS)<br>5 illness_indicators___5 Knee Outcome Survey Activities of Daily Living (KOS-ADL)<br>6 illness_indicators___6 Lysholm Knee Scoring Scale<br>7 illness_indicators___7 Tegner Activity Scale (TAS)<br>8 illness_indicators___8 Marx Activity Score (MAS)
	158	who_assessed_measures	Who assessed the measures	text
	159	results	Section Header: *Results*<br>Please detail the results	notes

	160	data_extraction_complete	Section Header: *Form Status*<br>Complete?	dropdown<br>0 Incomplete<br>1 Unverified<br>2 Complete

Supplementary Material 8. Strength of the treatment response or relationship between variables

Interpreting effect size values[1,2,3,4]

Effect Size Measure	Effect Size			
	Small	Medium	Large	Very Large
Odds Ratio	1.5	2.5	4	10
Cohen's *d*	0.2	0.5	0.8	1.3
r	0.1	0.3	0.5	0.7
Cohen's *f*	0.1	0.25	0.4	-
Eta-squared	0.01	0.06	0.14	-

1. Maher JM, Markey JC, Ebert-May D. The Other Half of the Story: Effect Size Analysis in Quantitative Research. *CBE Life Sci Educ* 2013;12(3):345-51. doi: 10.1187/cbe.13-04-0082

2. Rosenthal JA. Qualitative Descriptors of Strength of Association and Effect Size. J Soc Serv Res 1996;21(4):37-59. doi: 10.1300/j079v21n04_02

3. Cohen J. A power primer. Psychological bulletin 1992;112(1):155.

4. Cohen J. Statistical Power Analysis for the Behavioral Sciences. 2nd Edition ed. New York: Routledge 1988.

For peer review only

Supplementary Material 9. Adapted Grading of Recommendations, Assessment, Development and Evaluations (GRADE) Guidelines for Scoping Review

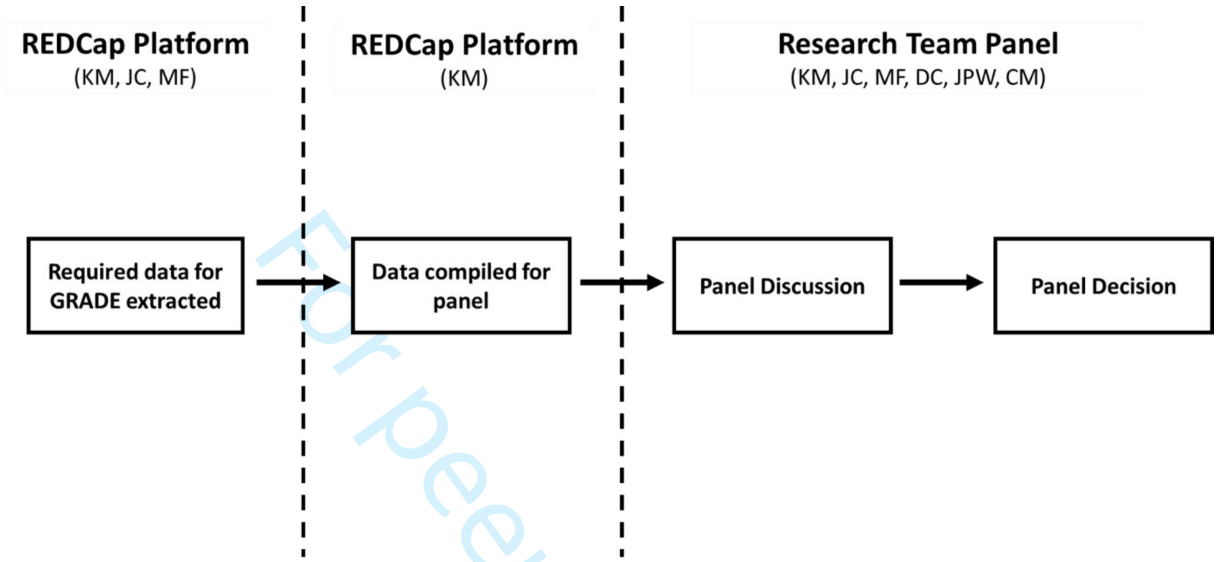

For peer review only

Draft - Adapted Grading of Recommendations, Assessment, Development and Evaluations (GRADE) Guidelines for Scoping Review

Overview:

Grading of Recommendations, Assessment, Development and Evaluations (GRADE)[1-3] is structured as:

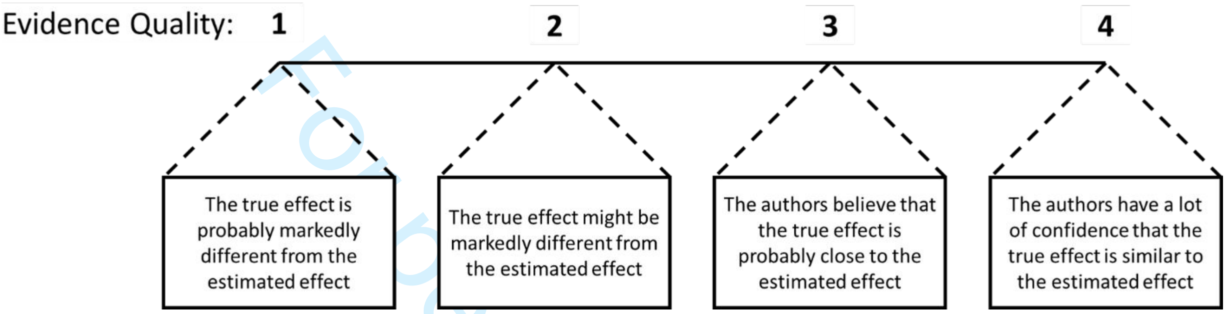

In GRADE, randomised control trials (RCTs) are the highest quality of research evidence (4), whilst observational studies (e.g., analytical cross-sectional studies) are the lowest form of evidence (1).[1-3] From there, individual studies will either be downgraded or upgraded for quality of evidence. For example, if an RCT is not downgraded it will provide the highest quality of evidence (4).[1-3]

In this review, the GRADE criteria will be applied to the research question '***what is the role of physical activity in the trajectory from intra-articular knee injury to post-traumatic osteoarthritis in young men and women?***' and will be considered through each of the following themes:

- Physical activity
- Systemic inflammation
- Adiposity
- Knee joint loading
- Lower body muscle strength
- Muscle size
- Intra-muscular fat
- Bone mineral density

Downgrade quality of evidence

1. Risk of Bias (RoB)

According to Ma *et al.*,[4] "Quality includes internal and external validity, while methodological quality usually refers to internal validity. Internal validity is also recommended as "risk of bias (RoB)" by the Cochrane Collaboration."

In this review, we will use the Joanna Briggs Institute (JBI) Critical Appraisal Checklists (Supplementary Material 6) to assess the RoB of each individual study. The Critical Appraisal Checklists apply to:

- Analytical Cross Sectional Studies[5]
- Case Control Studies[5]
- Cohort Studies[5]
- Studies Reporting Prevalence Data[6]
- Quasi-Experimental Studies[7]
- Randomised Control Trials[7]
- Systematic Review and Research Synthesis[8]

For the purpose of this review, we will classify the scores generated from JBI Critical Appraisal Checklists as either:

- Very low quality (0 – 25.0%)
- Low quality (>25.0 – 50.0%)
- Moderate quality (>50.0 – 75.0%)
- High quality (75.0 – 100%)

This scoring system will be applied to studies on a case-by-case basis.

Following application of the scoring system to all studies, we will adopt GRADE guidelines by downgrading studies if, collectively for the outcome, there is either a serious risk of bias or a very serious risk of bias. The scoring criteria for this review will be as follows:

- (**0**) No serious risk of bias overall = all studies scored moderate to high quality
- (**-1**) Serious risk of bias overall = more than 25% of studies considered low or very low quality
- (**-2**) Very serious risk of bias overall = More than 50% of studies considered low or very low quality

2. Inconsistency

Study results will be considered consistent when directions (e.g., positive or negative association), are considered similar enough to draw uniform conclusions. In this review, consistency in direction will be defined as >75% of the included studies for each theme demonstrating the same positive effect only or negative effect only.

For peer review only

- (**-1**) Serious risk of inconsistency when ≤75% of the included studies have the same direction of effect.
- (**-2**) Very serious risk of inconsistency when there is only one or no studies.

3. Indirectness

GRADE distinguishes several types of indirectness[9]. In this review, indirectness may be due to any of the factors detailed below:

Independent variable (*applicability*)[9]

- (**-1**) Serious risk of indirectness when the independent variable typically used by studies is deemed less valid than the gold standard (e.g., adiposity inferred from body mass index rather than dual-energy x-ray absorptiometry)
- (**-1**) Serious risk of indirectness an intervention cannot be completed in multiple settings (e.g., hospital and home based)

If no studies test a relevant intervention to the research question, or there is no research investigating any of the identified themes, we will conclude that the effects are uncertain.

Differences in outcome measures (*surrogate outcomes*)[9]

- (**-1**) Serious risk of indirectness when research typically uses of a surrogate measure which is associated with knee PTOA (e.g., systemic concentrations of type-II collagen biomarker), rather than directly diagnosing PTOA
- (**-1)** Serious risk of indirectness when studies do not provide information on full time course of PTOA development (e.g., only short-term effects of a physical activity intervention on a surrogate provide, and no studies investigating the long-term impact of PTOA diagnosis)

Indirect comparison[9]

- (**-1**) Serious risk of indirectness by lack of comparisons between physical activity types or exercise modalities
- (**-1**) Serious risk of indirectness by lack of comparison to alternative approaches (e.g., pharmaceutical driven pain relief)

4. Imprecision

As the data will not be combined in a meta-analysis, the following qualitative assessment will be applied to determine imprecision[10]:

- (**-1**) Serious risk of imprecision when the lower threshold of 95% CI reduces the effect to negligible

For peer review only

- (**-1**) Serious risk of imprecision when effect of estimate presented in only one or two studies or estimate comes from one or two events
- (**-2**) Very serious risk of imprecision if the lower threshold of 95% CI is consistent with conflicting conclusions
- (**-2**) Very serious risk of imprecision if CIs were not provided in any of the studies

5. Publication Bias

Assessment of publication bias using statistical methods or funnel plots will not be possible due to heterogeneity of the studies in this review. Publication bias will be screened for qualitatively as detailed in Chapter 7 of the Cochrane Handbook.

For peer review only

Upgrade quality of evidence

1. Large Magnitude of Effect

In this review, when a very large magnitude of effect is observed (assessed using Supplementary Material 8), there is greater certainty that there is at least a small effect.[11]

- (**+1**) when the effect size is large
- (**+2**) when the effect size is very large

The time-course of outcomes will also be considered in this review[12] when upgrading due to large magnitude of effect and devised into:

- (**+1**) Short-term (e.g., response to single exercise session/alteration of joint loading)
  - Systemic biomarkers in synovial fluid/serum/plasma/urine
  - MRI or US indicates deterioration
  - Symptoms or function

- (**+1**) Medium-term (e.g., response to 12-week activity intervention)
  - Systemic biomarkers in synovial fluid/serum/plasma/urine
  - MRI or US indicates deterioration
  - Symptoms or function

- (**+2**) Long-term (e.g., Body Mass Index at one-year post-injury and PTOA development in the years following)
  - X-Ray or MRI PTOA Diagnosis

This is because biomarkers concentrations changes can occur rapidly due to mechanical compression,[13-16] or an exercise-based intervention.[17] Furthermore, the basal protein synthesis rates in various musculoskeletal tissues (including cartilage, meniscus, bone) are within the same range of skeletal muscle protein synthesis rates[18] which may indicate pathology much faster than it takes for PTOA to fully develop.

Lastly, if there are major concerns regarding risk of bias, consistency, precision, or publication bias, in addition to being downgraded, the quality of evidence will not be upgraded via magnitude of effect.[12]

2. Dose-Response Gradient

This increases our confidence in the findings of observational studies and thus enhance the assigned quality of evidence.[12] This is best represented by RCTs and quasi-experimental trials.

- (**+1**) when there is evidence of a dose (e.g., physical activity-based intervention) and response (e.g., increased concentrations of aggrecan

For peer review only

synthesis, lower concentrations of type-II collagen breakdown, slower MRI defined joint tissue degeneration)

3. **All residual confounding would decrease magnitude of effect (in situations with an effect)**

All plausible residual confounders or biases would reduce a demonstrated effect, or suggest a spurious effect when results show no effect.[12]

REFERENCES

1. Guyatt GH, Oxman AD, Kunz R, et al. What is “quality of evidence” and why is it important to clinicians? *BMJ* 2008;336(7651):995-98. doi: 10.1136/bmj.39490.551019.be
2. Guyatt GH, Oxman AD, Vist GE, et al. GRADE: an emerging consensus on rating quality of evidence and strength of recommendations. *BMJ* 2008;336(7650):924-26. doi: 10.1136/bmj.39489.470347.ad
3. Guyatt G, Oxman AD, Akl EA, et al. GRADE guidelines: 1. Introduction-GRADE evidence profiles and summary of findings tables. *J Clin Epidemiol* 2011;64(4):383-94. doi: 10.1016/j.jclinepi.2010.04.026 [published Online First: 20101231]
4. Ma L-L, Wang Y-Y, Yang Z-H, et al. Methodological quality (risk of bias) assessment tools for primary and secondary medical studies: what are they and which is better? *Mil Med Res* 2020;7(1) doi: 10.1186/s40779-020-00238-8
5. Moola S, Munn Z, Tufanaru C, et al. Chapter 7: Systematic reviews of etiology and risk. In: Aromataris E, Munn Z, eds. JBI Manual for Evidence Synthesis: JBI, 2020.
6. Munn Z, Moola S, Lisy K, et al. Methodological guidance for systematic reviews of observational epidemiological studies reporting prevalence and cumulative incidence data. *Int J Evid Based Healthc* 2015;13(3):147-53. doi: 10.1097/XEB.0000000000000054
7. Tufanaru C, Munn Z, Aromataris E, et al. Chapter 3: Systematic reviews of effectiveness. In: Aromataris E, Munn Z, eds. JBI Manual for Evidence Synthesis: JBI, 2020.
8. Aromataris E, Fernandez R, Godfrey CM, et al. Summarizing systematic reviews: methodological development, conduct and reporting of an umbrella review approach. *Int J Evid Based Healthc* 2015;13(3):132-40. doi: 10.1097/XEB.0000000000000055
9. Guyatt GH, Oxman AD, Kunz R, et al. GRADE guidelines: 8. Rating the quality of evidence—indirectness. *J Clin Epidemiol* 2011;64(12):1303-10. doi: 10.1016/j.jclinepi.2011.04.014
10. Guyatt GH, Oxman AD, Kunz R, et al. GRADE guidelines 6. Rating the quality of evidence—imprecision. *J Clin Epidemiol* 2011;64(12):1283-93. doi: 10.1016/j.jclinepi.2011.01.012

For peer review only

11. Reed S, Guyatt G. What is GRADE? BMJ: BMJ; 2022 [Available from: https://bestpractice.bmj.com/info/toolkit/learn-ebm/what-is-grade/ accessed 20/06/2022 2022.
12. Guyatt GH, Oxman AD, Sultan S, et al. GRADE guidelines: 9. Rating up the quality of evidence. *J Clin Epidemiol* 2011;64(12):1311-16. doi: 10.1016/j.jclinepi.2011.06.004
13. Mundermann A, Dyrby CO, Andriacchi TP, et al. Serum concentration of cartilage oligomeric matrix protein (COMP) is sensitive to physiological cyclic loading in healthy 9adults. *Osteoarthritis Cartilage* 2005;13(1):34-38. doi: 10.1016/j.joca.2004.09.007 [published Online First: 2005/01/11]
14. Mündermann A, King KB, Smith RL, et al. Change in serum COMP concentration due to ambulatory load is not related to knee OA status. *J Orthop Res* 2009;27(11):1408-13. doi: 10.1002/jor.20908 [published Online First: 2009/05/08]
15. Mundermann A, Klenk C, Billich C, et al. Changes in Cartilage Biomarker Levels During a Transcontinental Multistage Footrace Over 4486 km. *Am J Sports Med* 2017;45(11):2630-36. doi: 10.1177/0363546517712945 [published Online First: 2017/06/27]
16. Roberts HM, Law RJ, Thom JM. The time course and mechanisms of change in biomarkers of joint metabolism in response to acute exercise and chronic training in physiologic and pathological conditions. *Eur J Appl Physiol* 2019;119(11-12):2401-20. doi: 10.1007/s00421-019-04232-4 [published Online First: 2019/10/28]
17. Azukizawa M, Ito H, Hamamoto Y, et al. The effects of well-rounded exercise program on systemic biomarkers related to cartilage metabolism. *Cartilage* 2019;10(4):451-58. doi: 10.1177/1947603518767998 [published Online First: 2018/04/13]
18. Smeets JSJ, Horstman AMH, Vles GF, et al. Protein synthesis rates of muscle, tendon, ligament, cartilage, and bone tissue in vivo in humans. *PLoS One* 2019;14(11):e0224745. doi: 10.1371/journal.pone.0224745 [published Online First: 2019/11/08]

For peer review only

Understanding the Role of Physical Activity on the Pathway from Intra-Articular Knee Injury to Post-Traumatic Osteoarthritis Disease in Young People: A Scoping Review Protocol

Journal:	*BMJ Open*
Manuscript ID	bmjopen-2022-067147.R1
Article Type:	Protocol
Date Submitted by the Author:	05-Jan-2023
Complete List of Authors:	Morgan, Karl; University of Bath, Department for Health; Centre for Health and Injury and Illness Prevention in Sport (CHI2PS), University of Bath<br>Cowburn, James; University of Bath, Department for Health<br>Farrow, Matthew; University of Bath, Department for Health; Centre for Nutrition and Exercise Metabolism (CNEM), University of Bath<br>Carter, Josh; University of Bath, Department for Health<br>Cazzola, Dario; University of Bath, Department for Health; Centre for the Analysis of Motion, Entertainment Research and Applications (CAMERA), University of Bath<br>Walhin, Jean-Philippe; University of Bath, Department for Health; Centre for Nutrition and Exercise Metabolism (CNEM), University of Bath<br>McKay, Carly; University of Bath, Department for Health; Centre for Health and Injury and Illness Prevention in Sport (CHI2PS), University of Bath
**Primary Subject Heading**:	Sports and exercise medicine
Secondary Subject Heading:	Rheumatology
Keywords:	Knee < ORTHOPAEDIC & TRAUMA SURGERY, Orthopaedic sports trauma < ORTHOPAEDIC & TRAUMA SURGERY, SPORTS MEDICINE, RHEUMATOLOGY, Musculoskeletal disorders < ORTHOPAEDIC & TRAUMA SURGERY

Title Page

Title: Understanding the Role of Physical Activity on the Pathway from Intra-Articular Knee Injury to Post-Traumatic Osteoarthritis Disease in Young People: A Scoping Review Protocol

Authors: Karl Morgan*[1 2 3 5], Dr James Cowburn[1 2], Dr Matthew Farrow[1 3 5], Josh Carter[1], Dr Dario Cazzola[1 2 4], Dr Jean-Philippe Walhin[1 3], Dr Carly McKay[1 2 5]

Department for Health, University of Bath, Bath, UK[1]

Centre for Health and Injury and Illness Prevention in Sport (CHI[2]PS), Department for Health, University of Bath, Bath, UK[2]

Centre for Nutrition and Exercise Metabolism (CNEM), Department for Health, University of Bath, Bath, UK[3]

Centre for the Analysis of Motion, Entertainment Research and Applications (CAMERA), University of Bath, Bath, UK[4]

Centre for Sport, Exercise and Osteoarthritis Versus Arthritis, University of Bath, Bath, UK[5]

***Corresponding Author:** kdm40@bath.ac.uk, Department for Health, Claverton Down, Bath, UK, BA2 7AY

Word Count: 2, 614

For peer review only

For peer review only - http://bmjopen.bmj.com/site/about/guidelines.xhtml

ABSTRACT

Introduction

The prevalence of intra-articular knee injuries and reparative surgeries is increasing in many countries. Alarmingly, there is a risk of developing post-traumatic osteoarthritis (PTOA) after sustaining a serious intra-articular knee injury. Although physical inactivity is suggested as a risk factor contributing to the high prevalence of the condition, there is a paucity of research characterising the association between physical activity and joint health. Consequently, the primary aim of this review will be to identify and present available empirical evidence regarding the association between physical activity and joint degeneration after intra-articular knee injury and summarise the evidence using an adapted Grading of Recommendations, Assessment, Development and Evaluations. The secondary aim will be to identify potential mechanistic pathways through which physical activity could influence PTOA pathogenesis. The tertiary aim will be to highlight gaps in current understanding of the association between PA and joint degeneration following joint injury.

Methods

A scoping review will be conducted using the Preferred Reporting Items for Systematic reviews and Meta-Analyses extension for Scoping Reviews checklist and best-practice recommendations. The review will be guided by the following research question: what is the role of physical activity in the trajectory from intra-articular knee injury to post-traumatic osteoarthritis in young men and women? We will identify primary research studies and grey literature by searching the electronic databases Scopus, Embase: Elsevier, Medline, Web of Science: all databases, and Google Scholar. Reviewing pairs will screen abstracts, full texts, and will extract data. Data will be presented descriptively using charts, graphs, plots, and tables.

Ethics and dissemination

For peer review only - http://bmjopen.bmj.com/site/about/guidelines.xhtml

This research does not require ethical approval due to the data being published and publicly available. This review will be submitted for publication in a peer-reviewed sports medicine journal irrespective of discoveries and disseminated through scientific conference presentations and social media.

Registration

This scoping review is registered on the Open Science Framework (https://osf.io/84pnh/)

Strengths and limitations of this study

- A scoping review is the most appropriate approach to identify and describe current evidence on the complex relationship between PA and post-traumatic osteoarthritis due to the multi-faceted aetiology underpinning the condition.
- This novel research lays an important foundation for further empirical research by mapping current evidence in the literature, examining potential mechanistic pathways using a multi-disciplinary approach, and identifying gaps in current knowledge.
- Due to the broad nature of a scoping review, this approach may limit the ability to comprehensively elucidate pathological mechanisms.

For peer review only

INTRODUCTION

Knee injuries, resulting from both contact and non-contact mechanisms,[1-9] are a common orthopaedic complaint in young, sporting populations worldwide[10] and often involve damage to multiple tissues inside the joint.[11-14] Reconstruction surgery is commonly used to treat the injured tissue,[9 11 13 15 16] with hundreds of thousands of knee injuries and reparative reconstruction procedures performed worldwide every year. [13 17-19] Current data suggests rates of intra-articular injury and tissue repair are rising in several countries,[17-19] with knee injuries predominantly affecting people under 40 years of age, [13 17-19] which is alarming considering injury greatly increases the risk of developing knee osteoarthritis (OA).[20] Knee OA is a highly prevalent disorder[21] which is becoming more common,[21-23] and is composed of both a disease and an illness component.[24] OA disease is associated with illness,[25] and the threshold for disease inducing illness is low.[24] However, the presence of illness does not always indicate structural deterioration and disease does not always induce symptomology,[26 27] with history of knee injury alone enough to induce illness.[27]

Knee OA affecting people with a history of intra-articular injury is defined as post-traumatic osteoarthritis (PTOA).[28-31] There is 10-fold increased odds of experiencing PTOA disease between 3-10 years following a knee injury.[27] Further, the prevalence of the disease 11-17 years following knee trauma (48-52%)[29 32] is concerning when considering average age at diagnosis for idiopathic knee OA is 55 years,[33] which indicates a typical case of PTOA would increase disease burden duration. After disease diagnosis, the joint may deteriorate to end stage OA, resulting in increased odds for total knee replacement (TKR), which occurs at a noticeably younger age in populations with an injury history than those without.[34] Knee OA is the primary cause for TKR[35] and the number of TKRs in people aged under 55 is increasing.[36] As TKRs typically last 25 years,[37] these younger patients experiencing PTOA may sustain subsequent prosthesis failure within their lifetime.[38]

In terms of PTOA illness, after intra-articular knee injury or anterior cruciate ligament reconstruction there is a high prevalence of knee symptomology (e.g., pain, stiffness, swelling, crepitus) and loss of function.[39-43] Knee pain is described as the cardinal symptom of OA,[44] and often drives people to seek medical care.[45-47] When applying the pain-avoidance model,[48] developed from idiopathic

For peer review only - http://bmjopen.bmj.com/site/about/guidelines.xhtml

knee OA populations, to PTOA, experiencing disease and illness due to PTOA may exacerbate loss of function across the lifespan, thereby accelerating the decline below the threshold of independence (Figure 1).[46 49] Consequently, understanding joint deterioration and characterising risk factors for PTOA in knee injury populations, with a view to prevent or slow condition progression, is paramount.

FIGURE 1. HYPOTHETICAL TRAJECOTRY OF INJURY TO LIFELONG DISEASE BURDEN

Physical inactivity is postulated as one of several risk factors that may compound joint degeneration following intra-articular knee injury.[50 51] Subjective and objective measurements of physical activity (PA) levels indicate reduced participation following knee injury[52-60] and many individuals do not return to the same level of sports participation or return to sport at all after anterior cruciate ligament reconstruction.[61-63] Therapeutic exercise is a core treatment for knee OA, and is considered beneficial to joint health,[64] whilst physical inactivity has been touted as a key contributor to joint deterioration in idiopathic OA.[22 50] However, a recent narrative review suggested there is little minimal robust scientific evidence linking PA level and joint health following injury.[51] Furthermore, although PA often involves knee joint loading (e.g., walking or running), there is limited research investigating the association between cumulative joint loading (dictated by PA) and joint health after knee joint trauma. Lastly, there are several physiological maladaptations that can occur after a traumatic knee injury, such as; increased adiposity,[41 54 59 65-67] knee extensor and flexor weakness,[68-70] muscle atrophy,[71 72] intra-muscular fat accumulation,[73 74] and bone mineral density reduction,[75] which could influence joint health. However, these associations with PTOA are yet to be fully understood. Considering PA is causally associated with systemic inflammation,[76-78] adiposity,[79] muscle size[80 81] and quality,[81-86] and bone mineral density[87 88] in uninjured populations, exploring the associations between these factors in injured populations may help to explain the complex relationship between PA and joint health following intra-articular knee injury.

For peer review only

For peer review only - http://bmjopen.bmj.com/site/about/guidelines.xhtml

Consequently, this protocol documents the procedure for conducting the review. The primary aim of the scoping review will be to identify and present available empirical evidence regarding the association between PA and joint degeneration after intra-articular knee injury and summarise the evidence using an adapted Grading of Recommendations, Assessment, Development, and Evaluations (GRADE).[89-91] The secondary aim will be to establish potential mechanistic pathways through which PA could influence PTOA pathogenesis. The tertiary aim will be to highlight gaps in current understanding of the association between PA and joint health following injury.

METHODS

This scoping review is an exploratory project which will systematically map the literature relevant to the research question, identifies key concepts, theories and sources of evidence, and gaps in the research.[92 93] This protocol has been prepared to ensure the transparency of the review process and will be used as a guide when conducting the review and reporting the outcomes to minimise the risk of reporting bias.[94 95] Further, this protocol has been developed in accordance with the adapted Preferred Reporting Items for Systematic Review and Meta-Analysis Protocol (PRISMA-P)[96 97] for scoping reviews[95] (Supplementary Material 1). This review is registered on Open Science Framework (https://osf.io/84pnh/) and the final output will be underpinned both by the Preferred Reporting Items for Systematic reviews and Meta-Analyses extension for Scoping Reviews (PRISMA-ScR)[98] and best practice recommendations.[99-101] Deviations from this protocol when conducting the review will be documented and published in supplementary material of the final output. This scoping review is currently in progress, with the first search completed in August 2022 and has a projected end date of May 2023.

Patient and Public Involvement

During protocol design and planning, there was no patient or public involvement. However, previous research conducted by our group has highlighted people with history of a knee injury can

For peer review only

For peer review only - http://bmjopen.bmj.com/site/about/guidelines.xhtml

experience a lack of long-term support and often alter their -PA participation because of their previous knee injury.[102] Furthermore, although a recent narrative review[51] highlighted the need to characterise the relationship between PA and joint health following injury, there is no existing synthesis that has been conducted systematically or rigorously which highlights a lack of an objective information source and a clear consensus. Considering the complexity and multifaceted relationship between PA and joint health, and the heterogenous study designs used by researchers in the topic, the research team concluded a scoping review would provide a coherent and informative assessment of current knowledge regarding PA and joint health in those at risk of experiencing post-traumatic osteoarthritis.

Framework

This review will follow the Arksey and O'Malley framework and adhere to the Joanna Briggs Institute quality assessment recommendations (Figure 2).[99 103]

FIGURE 2. OUTLINE OF THE STUDY METHODOLOGY

Stage 1: Identifying the Research Question

The review will be guided by the following research question: What is the role of PA on the trajectory from intra-articular knee injury to post-traumatic osteoarthritis in young men and women? As PTOA is a joint condition with a 'disease' component and an 'illness' component,[24] we will only describe the association between PA, and the factors physical activity underpins, with PTOA disease in this review. Disease will be defined as: 'abnormalities of the structure and function of body organs and systems that can be specifically identified and described by reference to certain biological, chemical or other evidence'.[104] Additionally, we will only include young participants (aged 18-40 years old) in this review. Within the research question, the following themes have been identified *a priori*: PA, systemic inflammation, knee joint load, adiposity, strength (lower body), intra-muscular adipose tissue (lower

For peer review only - http://bmjopen.bmj.com/site/about/guidelines.xhtml

body), muscle size (lower body), and bone mineral content. For this review, we will use consensus definitions for intra-articular knee injury, PA, the themes underpinned by PA, and PTOA (Table 1). If there is no agreed or clear definition, then best practices used by previous literature or consensus among the research team will be adopted.

Table 1. Review Definitions

Term	Definition
**Post-traumatic osteoarthritis**	Joint disorder characterised by extracellular matrix degradation initiated by micro- and macro-injury resulting in the manifestation of molecular derangement (e.g., abnormal joint tissue metabolism) followed by anatomic, and/or physiologic derangements (e.g., cartilage degradation), that can culminate in illness.[24] In this review, we will focus on the disease component of the disorder.
**Intra-articular knee injury**	Previous research has identified cruciate ligament and meniscus injuries have the highest risk of developing into post-traumatic osteoarthritis.[20] Considering these are also the most common types of intra-articular knee injury, our review will focus on both ligament and meniscus injuries only.
**Physical activity**	Any bodily movement produced by skeletal muscles that results in energy expenditure.[105] Further, we will include the term 'exercise' within this definition of physical activity, as although they describe different concepts, they are often used as synonyms.[105] Exercise is defined as a subset of physical activity that is planned, structured, and repetitive and has as a final or an immediate objective the improvement or maintenance of physical fitness.[105]
**Systemic inflammation**	A consensus definition on the type, number, and value of inflammatory biomarkers for systemic inflammation diagnosis has not been devised. In this review, we will use concentrations of key circulating proteins and cytokines identified in previous research as being involved with inflammation processes and OA.[106-111]
**Knee joint load**	The research team defines knee joint load as quantification of the forces and/or moments applied to the knee joint specifically.
**Strength (lower body)**	Strength is the ability to produce a maximal voluntary muscular contraction against an external resistance.[112] We consider the following movements as relevant to this review:<br>- Hip extension/flexion/abduction/adduction<br>- Knee extension/flexion/abduction/adduction<br>- Ankle dorsi/plantar flexion, supination/pronation
**Adipose tissue**	Specialised loose connective tissue that is extensively laden with adipocytes.[113] Adipose tissue is arranged in discrete depots,[114] and we consider the following depots at any body location relevent to this review:<br>- subcutaneous<br>- intraperitoneal/viseral<br>- inter-muscular (considered to be similar to intraperitoneal/visceral[115]
**Intra-muscular adipose tissue (lower body)**	Intra-muscular adipose tissue is the visible fat found within a muscle.[115] We consider the intra-muscular adipose content in any of the following muscle groups/muscles relevant to this review:<br>- Gluteal<br>- Hamstring<br>○ Bicep femoris

For peer review only - http://bmjopen.bmj.com/site/about/guidelines.xhtml

	o Semitendinosus<br>o Semimembranosus<br>- Quadriceps<br>o Vastus Lateralis<br>o Rectus Femoris<br>o Vastus Medialis<br>o Vastus Intermedius<br>- Tibialis anterior<br>- Gastrocnemius<br>- Soleus
**Muscle size (lower body)**	Muscle size if the dimension of skeletal muscle, which can be devised into a multi-scale[116] consisting of;<br>- organ<br>- tissue<br>- cellular<br>- sub-cellular<br>- molecular<br><br>We consider the muscle size of muscle groups/muscles relevant to this review:<br>- Gluteal<br>- Hamstring<br>o Bicep Femoris<br>o Semitendinosus<br>o Semimembranosus<br>- Quadriceps<br>o Vastus Lateralis<br>o Rectus Femoris<br>o Vastus Medialis<br>o Vastus Intermedius<br>- Tibialis anterior<br>- Gastrocnemius<br>- Soleus
**Bone mineral content**	The research team defines bone mineral content as the amount of minerals (mostly calcium and phosphorous) contained in bone.<br><br>Total bone mineral content of the body is considered relevant to this review. Additionally, the mineral content of the following bones is also considered relevant to this review:<br>- Femur<br>- Tibia<br>- Fibular

For peer review only

Stage 2: Identifying Relevant Studies

The search terms include population, independent variable, and outcome (Supplementary Material 2). Using the Peer Review of Electronic Search Strategy (PRESS)[117] guidelines, a professional health science librarian was consulted to develop and tailor search strategies for each database. Electronic databases Scopus, Embase: Elsevier, Medline, Web of Science: all databases, and Google Scholar will be searched. Literature published between 1970 and 2022 will be included, covering the

period during which clinical knee injury diagnosis was formalised and surgical reparative techniques were popularised.[118 119] Primary research studies and grey literature will be included.[93 120 121]

Stage 3: Study Selection

Inclusion and exclusion criteria are presented in Table 2.

Table 2. Review Inclusion and Exclusion Criteria

**Inclusion**	**Exclusion**
Includes participants with a history of experiencing intra-articular injury or reparative surgery for the injury.	Reporting of any other medical condition (disease, illness, or disorder):<br>- Musculoskeletal<br>- Immunological<br>- Cardiological<br>- Respiratory<br>- Neurological<br>- Metabolic
Measures any association between PA or factors determined by PA (systemic markers of inflammation, joint loading, adiposity, muscle strength, lower body muscle size, inter-muscular fat, bone density), and either post-traumatic osteoarthritis diagnosis or indicators of joint degeneration.	Evidence of intra-articular injury incidence to any of the following joints prior to or following intra-articular knee injury:<br>- Ankle<br>- Knee<br>- Hip<br>- Shoulder<br>- Elbow
18-40 years of age	Revision surgery if undergone initial reparative surgery
Primary research studies, grey literature	Animal models, *in vitro* studies, purely *in silico* studies with no human component (i.e., simulated data with no data capture element working with humans)
Human research	
Published in English	
Literature published between 1970 and 2022	

Outcome measures for PTOA disease must include medical imaging or molecular indicators of joint health (e.g., metabolic, proteomic, genomic, transcriptomic).[24] Imaging diagnosis of disease may include radiography[122 123] or magnetic resonance imaging (MRI).[124-126] Imaging indicators for disease processes include MRI[127-130] and ultrasound (US) techniques[131-133]. For molecular indicators of disease processes, OsteoArthritis Research Society International (OARSI) recommended biomarkers[134] will be included alongside other biomarkers of cartilage metabolism which have been deemed of interest in

For peer review only - http://bmjopen.bmj.com/site/about/guidelines.xhtml

previous reviews due to their association with joint health (Supplementary Material 3).[135-138] This list of outcome measures is not exhaustive and will be added to if other relevant outcomes are identified during Stage 2 - Identifying Relevant Studies.

To establish the face and content validity of the inclusion and exclusion criteria, and the selected outcome measures, feedback was sought from a surgeon, an osteopath, a physiotherapist, a physiologist, and a biomechanist independent from the research team, with experience ranging from clinical practice to sport research. These inclusion and exclusion criteria will be applied to title/abstract screening, which will be conducted using Covidence (Covidence Systematic Review Software, Veritas Health Innovation, Melbourne, Australia. Available at www.covidence.org). Prior to screening start, JC, JC, and MF will be familiarised and trained[139] by the lead author (KM) to conduct title/abstract screening using a screening tool hosted on Research Electronic Data Capture Platform (REDCap)[140] (Supplementary Material 4). Following the training session, inter-rater reliability of the inclusion and exclusion criteria for title/abstract screening will be assessed based on a random sample of 120 titles/abstracts. Percentage agreement and Cohen's Kappa (κ)[141] will be determined between all reviewers (KM - 100% of records, JC - 33%, JC - 33%, MF - 33%). The inclusion and exclusion criteria will be deemed reliable if the agreement is 'strong' (percentage agreement >63% and κ >0.60),[142] and title/abstract screening will only begin once the agreement level is strong. Following training and provided 'strong' agreement between reviewers is achieved, title/abstract screening will begin. During this process reviewing pairs (KM - 100% of records, JC - 33%, JC - 33%, MF - 33%) will independently screen all titles and abstracts to reduce quantity of errors.[143] If disagreements cannot be resolved within reviewing pairs, a consensus decision across the wider research team will be made following a discussion. Duplicate results returned during title/abstract screening will be removed using Covidence.

Following title and abstract screening, full text screening will be conducted by reviewing pairs (KM - 100% of records, JC - 33%, JC - 33%, MF - 33%).[139] Prior to full text screening, JC, JC and MF will be familiarised and trained[139] by the lead author (KM) to conduct full text screening using a screening tool hosted on Research Electronic Data Capture Platform (REDCap)[140] (Supplementary

For peer review only - http://bmjopen.bmj.com/site/about/guidelines.xhtml

Material 5). Following the training session, inter-rater reliability of the inclusion and exclusion criteria for the full text screening will be assessed based on a random sample of 20 full texts. Once 'strong' agreement between reviewers is achieved, full text screening will begin. All full texts will be independently screened (KM - 100% of records, JC - 33%, JC - 33%, MF - 33%) to reduce quantity of errors.[143] If disagreements cannot be resolved within reviewing pairs, a consensus decision across the research team will be made following a discussion. In line with common practice[144 145] and best-practice recommendations,[146] we will contact corresponding and co-authors via e-mail if details of a study are unclear during title/abstract or full text screening (Supplementary Material 6). A maximum of two attempted contacts will be made. The number of corresponding authors contacted, co-authors contacted, replies, and whether adequate information was provided, will be reported. Following full text screening, the remaining relevant studies to the review will undergo forward/backward screening.

Quality and Critical Appraisal

In accordance with best-practice recommendations,[99] we will assess methodological quality before conducting data charting of included studies. As this review will include various study designs, we will employ Joanna Briggs Institute (JBI) appraisal tools respective to each study design (Supplementary Material 7).[147-150] Individual items within each JBI appraisal tool will be assigned either a 'yes' (1), 'no' (0), or 'unclear' (seek clarification/further information) response to questions relating to the research quality. If study details are unclear, corresponding authors will be contacted following the same process as described in Stage 3 – Study Selection (Supplementary Material 5). A quality percentage score will be calculated, with smaller scores indicating lower quality and larger scores indicating higher quality. Identified studies will be included regardless of the quality appraisal score. The appraisals will be conducted by reviewing pairs (KM - 100% of records, JC - 33%, JC - 33%, MF - 33%) using REDCap.[140]

Stage 4: Charting the Data

For peer review only - http://bmjopen.bmj.com/site/about/guidelines.xhtml

180 Data charting will be conducted in REDCap[140] by reviewing pairs (KM - 100% of records, JC
181 - 33%, JC - 33%, MF - 33%) to reduce the risk of errors in data extraction.[143] Data charting will include
182 author(s), country of study, study year, study design, participant age and sex/gender distributions, injury
183 type, surgery type, type of injury management, time since injury/surgery, sporting history before injury
184 (type and level), aim of study, independent variables, supervision level (if intervention), quantity (if
185 intervention), adherence rate (if intervention), drop out (if intervention), outcome variables, who
186 assessed the measures, statistical methodology, and the strength of the treatment response or
187 relationship between variables. This form (Supplementary Material 8) will be pilot tested by two
188 reviewers and necessary amendments will be made if required (e.g., other relevant measurement tools
189 or outcome variables). If study details are unclear, corresponding authors will be contacted following
190 the same process as described in Stage 3 – Study Selection.

191

192 Stage 5: Summarising the Data

193 Data will be summarised in three steps.[99] The first step will present the results as a descriptive
194 numerical summary which will include: number of studies on each theme of the research question;
195 frequency of study design; definitions and variables used for joint disease; overview of methodological
196 quality of studies. The second step will present the results as individual sections for each theme of the
197 research question. Each theme will be structured as follows:

198 - Strength of the treatment response or relationship between variables[151-154] (Supplementary
199 Material 9) for studies presented descriptively
200 - In accordance with a previous scoping review on ACL patients,[155] we will use the
201 GRADE[89-91] (incorporates Bradford Hill Criteria[156-158]) for quality of evidence for either a
202 protective or degenerative, or if there is an unknown effect (Supplementary Material 10).
203 Study-by-study results will be presented in supplementary material, whilst the overall
204 summary of results for each theme will be displayed within the article in a summary of
205 findings table

For peer review only - http://bmjopen.bmj.com/site/about/guidelines.xhtml

The third step will be to discuss and consider the meaning of the findings as they relate to the overall review purpose. We will also discuss implications for future PTOA research.

ETHICS AND DISSEMINATION

Ethical approval is not required as this review maps and synthesises data generated from published literature. This review will be submitted for publication in a peer-reviewed sports medicine journal, irrespective of findings which indicate a positive or negative association between PA and joint health. Knowledge synthesised by this review will be presented at scientific conferences and disseminated via social media platforms.

CONCLUSION

This study seeks to address a salient topic for people who experience a traumatic intra-articular knee injury and clinicians working with this population. Using a scoping review methodology to examine the known influence of PA on PTOA pathogenesis is novel and appropriate, considering the complexity of the topic and the multi-disciplinary evidence base. This research may inform future PTOA research by helping to guide research priorities and identifying outcome measures for clinical interventions.

CRediT (Contributor Roles Taxonomy)

Karl Morgan: Conceptualisation, Methodology, Writing - Original Draft, Writing - Review & Editing. **Dr James Cowburn:** Methodology, Writing - Review & Editing. **Dr Matthew Farrow:** Methodology, Writing - Review & Editing. **Josh Carter :** Methodology, Writing - Review & Editing. **Dr Dario Cazzola:** Writing - Review & Editing, Supervision. **Dr Jean-Philippe Walhin:** Writing - Review & Editing, Supervision. **Dr Carly McKay:** Conceptualisation, Methodology, Writing - Review & Editing, Supervision.

For peer review only - http://bmjopen.bmj.com/site/about/guidelines.xhtml

Acknowledgements

We thank Peter Bradley, the University of Bath's Subject Librarian for Health and Social & Policy Sciences for his help in developing the search terms and his role when implementing the Peer Review of Electronic Search Strategy (PRESS)[117] guidelines. We also thank Dr David Bruce, Dr Nicos Haralabidis, Rosy Hyman, Charli Robertson, and Lee Page for their expert feedback developing the inclusion/exclusion criteria and outcome measures.

Funding statement

This work was funded by the University of Bath (funding refence: EA-FH1112) in affiliation with the Centre for Sport, Exercise and Osteoarthritis Research Versus Arthritis, University of Bath (grant reference: 21595).

Conflict of interest statement

None declared.

For peer review only

REFERENCES

1. Cochrane JL, Lloyd DG, Buttfield A, et al. Characteristics of anterior cruciate ligament injuries in Australian football. *J Sci Med Sport* 2007;10(2):96-104. doi: 10.1016/j.jsams.2006.05.015 [published Online First: 20060627]

2. Walden M, Krosshaug T, Bjorneboe J, et al. Three distinct mechanisms predominate in non-contact anterior cruciate ligament injuries in male professional football players: a systematic video analysis of 39 cases. *Br J Sports Med* 2015;49(22):1452-60. doi: 10.1136/bjsports-2014-094573 [published Online First: 2015/04/25]

3. Montgomery C, Blackburn J, Withers D, et al. Mechanisms of ACL injury in professional rugby union: a systematic video analysis of 36 cases. *Br J Sports Med* 2018;52(15):994-1001. doi: 10.1136/bjsports-2016-096425 [published Online First: 2017/01/01]

4. Boden BP, Dean GS, Feagin JA, et al. Mechanisms of anterior cruciate ligament injury. *Orthopedics* 2000;23(6):573-8.

5. Olsen OE, Myklebust G, Engebretsen L, et al. Injury mechanisms for anterior cruciate ligament injuries in team handball: a systematic video analysis. *Am J Sports Med* 2004;32(4):1002-12. doi: 10.1177/0363546503261724 [published Online First: 2004/05/20]

6. Koga H, Nakamae A, Shima Y, et al. Mechanisms for noncontact anterior cruciate ligament injuries: knee joint kinematics in 10 injury situations from female team handball and basketball. *Am J Sports Med* 2010;38(11):2218-25. doi: 10.1177/0363546510373570 [published Online First: 2010/07/03]

7. Ruedl G, Linortner I, Schranz A, et al. Distribution of injury mechanisms and related factors in ACL-injured female carving skiers. *Knee Surg Sports Traumatol Arthrosc* 2009;17(11):1393-8. doi: 10.1007/s00167-009-0860-7 [published Online First: 2009/07/11]

8. Ruedl G, Webhofer M, Linortner I, et al. ACL injury mechanisms and related factors in male and female carving skiers: a retrospective study. *Int J Sports Med* 2011;32(10):801-6. doi: 10.1055/s-0031-1279719 [published Online First: 2011/06/23]

9. Gabr A, De Medici A, Haddad F. The National Ligament Registry 2019 Annual Report. In: Spalding T, ed. The National Ligament Registry, 2020:1-14.

10. Moses B, Orchard J, Orchard J. Systematic review: Annual incidence of ACL injury and surgery in various populations. *Res Sports Med* 2012;20(3-4):157-79. doi: 10.1080/15438627.2012.680633 [published Online First: 2012/06/30]

11. Noyes FR, Barber-Westin SD. Treatment of meniscus tears during anterior cruciate ligament reconstruction. *Arthroscopy* 2012;28(1):123-30. doi: 10.1016/j.arthro.2011.08.292 [published Online First: 2011/11/15]

12. Ghodadra N, Mall NA, Karas V, et al. Articular and meniscal pathology associated with primary anterior cruciate ligament reconstruction. *J Knee Surg* 2013;26(3):185-93. doi: 10.1055/s-0032-1327450 [published Online First: 2013/01/05]

For peer review only - http://bmjopen.bmj.com/site/about/guidelines.xhtml

13. Mall NA, Chalmers PN, Moric M, et al. Incidence and trends of anterior cruciate ligament reconstruction in the United States. *Am J Sports Med* 2014;42(10):2363-70. doi: 10.1177/0363546514542796 [published Online First: 2014/08/03]

14. Everhart JS, DiBartola AC, Swank K, et al. Cartilage damage at the time of anterior cruciate ligament reconstruction is associated with weaker quadriceps function and lower risk of future ACL injury. *Knee Surg Sports Traumatol Arthrosc* 2020;28(2):576-83. doi: 10.1007/s00167-019-05739-1 [published Online First: 2019/10/11]

15. Duthon VB, Barea C, Abrassart S, et al. Anatomy of the anterior cruciate ligament. *Knee Surg Sports Traumatol Arthrosc* 2006;14(3):204-13. doi: 10.1007/s00167-005-0679-9 [published Online First: 2005/10/20]

16. Lohmander LS, Englund PM, Dahl LL, et al. The long-term consequence of anterior cruciate ligament and meniscus injuries: osteoarthritis. *Am J Sports Med* 2007;35(10):1756-69. doi: 10.1177/0363546507307396 [published Online First: 2007/09/01]

17. Abram SGF, Price AJ, Judge A, et al. Anterior cruciate ligament (ACL) reconstruction and meniscal repair rates have both increased in the past 20 years in England: hospital statistics from 1997 to 2017. *Br J Sports Med* 2020;54(5):286-91. doi: 10.1136/bjsports-2018-100195 [published Online First: 2019/01/21]

18. Domnick C, Garcia P, Raschke MJ, et al. Trends and incidences of ligament-surgeries and osteotomies of the knee: an analysis of German inpatient records 2005-2013. *Arch Orthop Trauma Surg* 2017;137(7):989-95. doi: 10.1007/s00402-017-2704-0 [published Online First: 2017/05/04]

19. Zbrojkiewicz D, Vertullo C, Grayson JE. Increasing rates of anterior cruciate ligament reconstruction in young Australians, 2000-2015. *Med J Aust* 2018;208(8):354-58. doi: doi:10.5694/mja17.00974 [published Online First: 2018/04/20]

20. Snoeker B, Turkiewicz A, Magnusson K, et al. Risk of knee osteoarthritis after different types of knee injuries in young adults: a population-based cohort study. *Br J Sports Med* 2020;54(12):725-30. doi: 10.1136/bjsports-2019-100959 [published Online First: 2019/12/13]

21. Morgan OJ, Hillstrom HJ, Ellis SJ, et al. Osteoarthritis in England: incidence trends from National Health Service Hospital Episode Statistics. *ACR Open Rheumatol* 2019;1(8):493-98. doi: 10.1002/acr2.11071 [published Online First: 2019/11/30]

22. Wallace IJ, Worthington S, Felson DT, et al. Knee osteoarthritis has doubled in prevalence since the mid-20th century. *Proc Natl Acad Sci U S A* 2017;114(35):9332-36. doi: 10.1073/pnas.1703856114 [published Online First: 2017/08/16]

23. Kiadaliri AA, Rinaldi G, Lohmander LS, et al. Temporal trend and regional disparity in osteoarthritis hospitalisations in Sweden 1998–2015. *Scand J Public Health* 2019;47(1):53-60. doi: 10.1177/1403494818766785

24. Kraus VB, Blanco FJ, Englund M, et al. Call for standardized definitions of osteoarthritis and risk stratification for clinical trials and clinical use. *Osteoarthritis Cartilage* 2015;23(8):1233-41. doi: 10.1016/j.joca.2015.03.036 [published Online First: 2015/04/14]

For peer review only - http://bmjopen.bmj.com/site/about/guidelines.xhtml

25. Illingworth KD, El Bitar Y, Siewert K, et al. Correlation of WOMAC and KOOS scores to tibiofemoral cartilage loss on plain radiography and 3 Tesla MRI: data from the osteoarthritis initiative. *Knee Surg Sports Traumatol Arthrosc* 2014;22(7):1649-58.

26. Bedson J, Croft PR. The discordance between clinical and radiographic knee osteoarthritis: a systematic search and summary of the literature. *BMC Musculoskelet Disord* 2008;9:116. doi: 10.1186/1471-2474-9-116 [published Online First: 2008/09/04]

27. Whittaker JL, Toomey CM, Woodhouse LJ, et al. Association between MRI-defined osteoarthritis, pain, function and strength 3-10 years following knee joint injury in youth sport. *Br J Sports Med* 2018;52(14):934-39. doi: 10.1136/bjsports-2017-097576 [published Online First: 2017/10/12]

28. Friel NA, Chu CR. The role of ACL injury in the development of posttraumatic knee osteoarthritis. *Clin Sports Med* 2013;32(1):1-12. doi: 10.1016/j.csm.2012.08.017 [published Online First: 2012/11/28]

29. Luc B, Gribble PA, Pietrosimone BG. Osteoarthritis prevalence following anterior cruciate ligament reconstruction: a systematic review and numbers-needed-to-treat analysis. *J Athl Train* 2014;49(6):806-19. doi: 10.4085/1062-6050-49.3.35 [published Online First: 2014/09/19]

30. Gelber AC, Hochberg MC, Mead LA, et al. Joint injury in young adults and risk for subsequent knee and hip osteoarthritis. *Ann Intern Med* 2000;133(5):321-8. doi: 10.7326/0003-4819-133-5-200009050-00007 [published Online First: 2000/09/09]

31. Roos EM. Joint injury causes knee osteoarthritis in young adults. *Curr Opin Rheumatol* 2005;17(2):195-200. doi: 10.1097/01.bor.0000151406.64393.00 [published Online First: 2005/02/16]

32. Chen T, Wang S, Li Y, et al. Radiographic osteoarthritis prevalence over ten years after anterior cruciate ligament reconstruction. *Int J Sports Med* 2019;40(11):683-95. doi: 10.1055/a-0902-8539 [published Online First: 2019/09/19]

33. Losina E, Weinstein AM, Reichmann WM, et al. Lifetime risk and age at diagnosis of symptomatic knee osteoarthritis in the US. *Arthritis Care Res (Hoboken)* 2013;65(5):703-11. doi: 10.1002/acr.21898 [published Online First: 2012/12/04]

34. Khan T, Alvand A, Prieto-Alhambra D, et al. ACL and meniscal injuries increase the risk of primary total knee replacement for osteoarthritis: a matched case-control study using the Clinical Practice Research Datalink (CPRD). *Br J Sports Med* 2019;53(15):965-68. doi: 10.1136/bjsports-2017-097762 [published Online First: 2018/01/15]

35. Price AJ, Alvand A, Troelsen A, et al. Knee replacement. *Lancet* 2018;392(10158):1672-82. doi: 10.1016/s0140-6736(18)32344-4

36. W-Dahl A, Robertsson O, Lidgren L. Surgery for knee osteoarthritis in younger patients. *Acta Orthop* 2010;81(2):161-64. doi: 10.3109/17453670903413186

37. Evans JT, Walker RW, Evans JP, et al. How long does a knee replacement last? A systematic review and meta-analysis of case series and national registry reports with more than 15 years of follow-

For peer review only - http://bmjopen.bmj.com/site/about/guidelines.xhtml

up. *Lancet* 2019;393(10172):655-63. doi: 10.1016/S0140-6736(18)32531-5 [published Online First: 2019/02/21]

38. Bayliss LE, Culliford D, Monk AP, et al. The effect of patient age at intervention on risk of implant revision after total replacement of the hip or knee: a population-based cohort study. *Lancet* 2017;389(10077):1424-30. doi: 10.1016/s0140-6736(17)30059-4

39. Wasserstein D, Huston LJ, Nwosu S, et al. KOOS pain as a marker for significant knee pain two and six years after primary ACL reconstruction: a Multicenter Orthopaedic Outcomes Network (MOON) prospective longitudinal cohort study. *Osteoarthritis Cartilage* 2015;23(10):1674-84. doi: 10.1016/j.joca.2015.05.025 [published Online First: 2015/06/15]

40. Antosh IJ, Svoboda SJ, Peck KY, et al. Change in KOOS and WOMAC scores in a young athletic population with and without anterior cruciate ligament injury. *Am J Sports Med* 2018;46(7):1606-16. doi: 10.1177/0363546518768753 [published Online First: 2018/05/08]

41. Whittaker JL, Woodhouse LJ, Nettel-Aguirre A, et al. Outcomes associated with early post-traumatic osteoarthritis and other negative health consequences 3-10 years following knee joint injury in youth sport. *Osteoarthritis Cartilage* 2015;23(7):1122-9. doi: 10.1016/j.joca.2015.02.021 [published Online First: 2015/03/01]

42. Englund M, Roos EM, Lohmander LS. Impact of type of meniscal tear on radiographic and symptomatic knee osteoarthritis: a sixteen-year followup of meniscectomy with matched controls. *Arthritis Rheum* 2003;48(8):2178-87. doi: 10.1002/art.11088 [published Online First: 2003/08/09]

43. Harkey MS, Baez S, Lewis J, et al. Prevalence of early knee osteoarthritis illness among various patient-reported classification criteria after anterior cruciate ligament reconstruction. *Arthritis Care Res (Hoboken)* 2022;74(3):377-85. doi: 10.1002/acr.24809

44. Arden N, Nevitt MC. Osteoarthritis: epidemiology. *Best Pract Res Clin Rheumatol* 2006;20(1):3-25. doi: 10.1016/j.berh.2005.09.007 [published Online First: 2006/02/18]

45. Rathleff MS, Roos EM, Olesen JL, et al. High prevalence of daily and multi-site pain--a cross-sectional population-based study among 3000 Danish adolescents. *BMC Pediatr* 2013;13:191. doi: 10.1186/1471-2431-13-191 [published Online First: 2013/11/21]

46. Peat G. Knee pain and osteoarthritis in older adults: a review of community burden and current use of primary health care. *Ann Rheum Dis* 2001;60(2):91-97. doi: 10.1136/ard.60.2.91

47. Turkiewicz A, Gerhardsson De Verdier M, Engstrom G, et al. Prevalence of knee pain and knee OA in southern Sweden and the proportion that seeks medical care. *Rheumatology (Oxford)* 2015;54(5):827-35. doi: 10.1093/rheumatology/keu409

48. Holla JF, Sanchez-Ramirez DC, van der Leeden M, et al. The avoidance model in knee and hip osteoarthritis: a systematic review of the evidence. *J Behav Med* 2014;37(6):1226-41. doi: 10.1007/s10865-014-9571-8 [published Online First: 2014/05/21]

For peer review only - http://bmjopen.bmj.com/site/about/guidelines.xhtml

49. Shortland A. Muscle deficits in cerebral palsy and early loss of mobility: can we learn something from our elders? *Dev Med Child Neurol* 2009;51:59-63. doi: 10.1111/j.1469-8749.2009.03434.x

50. Osteoarthritis Research Society International. Osteoarthritis: A Serious Disease: Osteoarthritis Research Society International, 2016.

51. Whittaker JL, Roos EM. A pragmatic approach to prevent post-traumatic osteoarthritis after sport or exercise-related joint injury. *Best Pract Res Clin Rheumatol* 2019;33(1):158-71. doi: 10.1016/j.berh.2019.02.008 [published Online First: 2019/08/23]

52. Kvist J, Ek A, Sporrstedt K, et al. Fear of re-injury: a hindrance for returning to sports after anterior cruciate ligament reconstruction. *Knee Surg Sports Traumatol Arthrosc* 2005;13(5):393-7. doi: 10.1007/s00167-004-0591-8 [published Online First: 2005/02/11]

53. Ardern CL, Osterberg A, Sonesson S, et al. Satisfaction with knee function after primary anterior cruciate ligament reconstruction is associated with self-efficacy, quality of life, and returning to the preinjury physical activity. *Arthroscopy* 2016;32(8):1631-38. doi: 10.1016/j.arthro.2016.01.035 [published Online First: 2016/03/30]

54. Toomey CM, Whittaker JL, Nettel-Aguirre A, et al. Higher fat mass is associated with a history of knee injury in youth sport. *J Orthop Sports Phys Ther* 2017;47(2):80-87. doi: 10.2519/jospt.2017.7101 [published Online First: 2017/02/02]

55. Bell DR, Pfeiffer KA, Cadmus-Bertram LA, et al. Objectively measured physical activity in patients after anterior cruciate ligament reconstruction. *Am J Sports Med* 2017;45(8):1893-900. doi: 10.1177/0363546517698940 [published Online First: 2017/04/19]

56. Toomey C, Whittaker JL, Doyle-Baker PK, et al. The association between physical activity and 3–15 year history of sport-related intra-articular knee injury: a matched cohort design [Abstract]. *Br J Sports Med* 2018; 52. https://bjsm.bmj.com/content/52/Suppl_1/A6.1 (accessed 01/04/2021).

57. Kuenze C, Cadmus-Bertram L, Pfieffer K, et al. Relationship between physical activity and clinical outcomes after ACL reconstruction. *J Sport Rehabil* 2019;28(2):180-87. doi: 10.1123/jsr.2017-0186 [published Online First: 2017/11/16]

58. Kuenze C, Lisee C, Pfeiffer KA, et al. Sex differences in physical activity engagement after ACL reconstruction. *Phys Ther Sport* 2019;35:12-17. doi: 10.1016/j.ptsp.2018.10.016 [published Online First: 2018/11/06]

59. Triplett AN, Kuenze CM. Characterizing body composition, cardiorespiratory fitness, and physical activity in women with anterior cruciate ligament reconstruction. *Phys Ther Sport* 2020;48:54-59. doi: 10.1016/j.ptsp.2020.12.014 [published Online First: 2020/12/30]

60. Baez SE, Hoch MC, Hoch JM. Psychological factors are associated with return to pre-injury levels of sport and physical activity after ACL reconstruction. *Knee Surg Sports Traumatol Arthrosc* 2020;28(2):495-501. doi: 10.1007/s00167-019-05696-9 [published Online First: 2019/09/06]

For peer review only - http://bmjopen.bmj.com/site/about/guidelines.xhtml

61. Webster KE, Feller JA. Expectations for return to preinjury sport before and after anterior cruciate ligament reconstruction. *Am J Sports Med* 2019;47(3):578-83. doi: 10.1177/0363546518819454 [published Online First: 2019/01/17]

62. Ardern CL, Webster KE, Taylor NF, et al. Return to sport following anterior cruciate ligament reconstruction surgery: a systematic review and meta-analysis of the state of play. *Br J Sports Med* 2011;45(7):596-606. doi: 10.1136/bjsm.2010.076364 [published Online First: 2011/03/15]

63. Ardern CL, Taylor NF, Feller JA, et al. Fifty-five per cent return to competitive sport following anterior cruciate ligament reconstruction surgery: an updated systematic review and meta-analysis including aspects of physical functioning and contextual factors. *Br J Sports Med* 2014;48(21):1543-52. doi: 10.1136/bjsports-2013-093398

64. National Institute for Health and Care Excellence. Osteoarthritis: care and management (update): National Institute for Health and Care Excellence; 2022 [Available from: https://www.nice.org.uk/guidance/indevelopment/gid-ng10127/consultation/html-content-2 accessed 31/05/2022 2022.

65. MacAlpine EM, Talwar D, Storey EP, et al. Weight gain after ACL reconstruction in pediatric and adolescent patients. *Sports Health* 2020;12(1):29-35. doi: 10.1177/1941738119870192 [published Online First: 2019/09/06]

66. Patterson BE, Culvenor AG, Barton CJ, et al. Worsening knee osteoarthritis features on magnetic resonance imaging 1 to 5 years after anterior cruciate ligament reconstruction. *Am J Sports Med* 2018;46(12):2873-83. doi: 10.1177/0363546518789685 [published Online First: 2018/09/05]

67. Ezzat AM, Whittaker JL, Toomey C, et al. Knee confidence in youth and young adults at risk of post-traumatic osteoarthritis 3-10 years following intra-articular knee injury. *J Sci Med Sport* 2018;21(7):671-75. doi: 10.1016/j.jsams.2017.11.012 [published Online First: 2017/12/14]

68. Tayfur B, Charuphongsa C, Morrissey D, et al. Neuromuscular function of the knee joint following knee injuries: does it ever get back to normal? A systematic review with meta-analyses. *Sports Med* 2021;51(2):321-38. doi: 10.1007/s40279-020-01386-6 [published Online First: 2020/11/29]

69. Hart JM, Pietrosimone B, Hertel J, et al. Quadriceps activation following knee injuries: a systematic review. *J Athl Train* 2010;45(1):87-97. doi: 10.4085/1062-6050-45.1.87 [published Online First: 2010/01/13]

70. Lisee C, Lepley AS, Birchmeier T, et al. Quadriceps strength and volitional activation after anterior cruciate ligament reconstruction: a systematic review and meta-analysis. *Sports Health* 2019;11(2):163-79. doi: 10.1177/1941738118822739 [published Online First: 2019/01/15]

71. Birchmeier T, Lisee C, Kane K, et al. Quadriceps muscle size following ACL injury and reconstruction: a systematic review. *J Orthop Res* 2020;38(3):598-608. doi: 10.1002/jor.24489 [published Online First: 2019/10/15]

For peer review only

72. Dutaillis B, Maniar N, Opar DA, et al. Lower limb muscle size after anterior cruciate ligament injury: a systematic review and meta-analysis. *Sports Med* 2021; in press. https://www.ncbi.nlm.nih.gov/pubmed/33492623

https://link.springer.com/content/pdf/10.1007/s40279-020-01419-0.pdf (accessed 15/03/2021).

73. Lindstrom M, Strandberg S, Wredmark T, et al. Functional and muscle morphometric effects of ACL reconstruction. A prospective CT study with 1 year follow-up. *Scand J Med Sci Sports* 2013;23(4):431-42. doi: 10.1111/j.1600-0838.2011.01417.x [published Online First: 2011/11/24]

74. Lopresti C, Kirkendall DT, Street GM, et al. Quadriceps insufficiency following repair of the anterior cruciate ligament. *J Orthop Sports Phys Ther* 1988;9(7):245-49.

75. Van Meer BL, Waarsing JH, Van Eijsden WA, et al. Bone mineral density changes in the knee following anterior cruciate ligament rupture. *Osteoarthritis Cartilage* 2014;22(1):154-61. doi: 10.1016/j.joca.2013.11.005

76. Fischer CP, Berntsen A, Perstrup LB, et al. Plasma levels of interleukin-6 and C-reactive protein are associated with physical inactivity independent of obesity. *Scand J Med Sci Sports* 2007;17(5):580-7. doi: 10.1111/j.1600-0838.2006.00602.x [published Online First: 2006/11/02]

77. Hamer M. The relative influences of fitness and fatness on inflammatory factors. *Prev Med* 2007;44(1):3-11. doi: 10.1016/j.ypmed.2006.09.005 [published Online First: 2006/10/27]

78. Lavin KM, Perkins RK, Jemiolo B, et al. Effects of aging and lifelong aerobic exercise on basal and exercise-induced inflammation. *J Appl Physiol (1985)* 2020;128(1):87-99. doi: 10.1152/japplphysiol.00495.2019 [published Online First: 2019/11/22]

79. Myers A, Gibbons C, Finlayson G, et al. Associations among sedentary and active behaviours, body fat and appetite dysregulation: investigating the myth of physical inactivity and obesity. *Br J Sports Med* 2017;51(21):1540-44. doi: 10.1136/bjsports-2015-095640 [published Online First: 2016/04/06]

80. Krogh-Madsen R, Thyfault JP, Broholm C, et al. A 2-wk reduction of ambulatory activity attenuates peripheral insulin sensitivity. *J Appl Physiol (1985)* 2010;108(5):1034-40. doi: 10.1152/japplphysiol.00977.2009 [published Online First: 2010/01/02]

81. Manini TM, Clark BC, Nalls MA, et al. Reduced physical activity increases intermuscular adipose tissue in healthy young adults. *Am J Clin Nutr* 2007;85(2):377-84. doi: 10.1093/ajcn/85.2.377 [published Online First: 2007/02/08]

82. Farr JN, Van Loan MD, Lohman TG, et al. Lower physical activity is associated with skeletal muscle fat content in girls. *Med Sci Sports Exerc* 2012;44(7):1375-81. doi: 10.1249/MSS.0b013e31824749b2 [published Online First: 2012/01/06]

83. Dimmick HL, Miller JD, Sterczala AJ, et al. Vastus lateralis muscle tissue composition and motor unit properties in chronically endurance-trained vs. sedentary women. *Eur J Appl Physiol* 2018;118(9):1789-800. doi: 10.1007/s00421-018-3909-9 [published Online First: 2018/06/28]

For peer review only - http://bmjopen.bmj.com/site/about/guidelines.xhtml

84. Pagano AF, Brioche T, Arc-Chagnaud C, et al. Short-term disuse promotes fatty acid infiltration into skeletal muscle. *J Cachexia Sarcopenia Muscle* 2018;9(2):335-47. doi: 10.1002/jcsm.12259 [published Online First: 2017/12/17]

85. MacLennan RJ, Sahebi M, Becker N, et al. Declines in skeletal muscle quality vs. size following two weeks of knee joint immobilization. *PeerJ* 2020; 8. https://www.ncbi.nlm.nih.gov/pmc/articles/PMC6964688/

https://www.ncbi.nlm.nih.gov/pmc/articles/PMC6964688/pdf/peerj-08-8224.pdf (accessed 18/04/2021).

86. Tuttle LJ, Sinacore DR, Cade WT, et al. Lower physical activity is associated with higher intermuscular adipose tissue in people with type 2 diabetes and peripheral neuropathy. *Phys Ther* 2011;91(6):923-30. doi: 10.2522/ptj.20100329 [published Online First: 2011/04/09]

87. Chastin SF, Mandrichenko O, Helbostadt J, et al. Associations between objectively-measured sedentary behaviour and physical activity with bone mineral density in adults and older adults, the NHANES study. *Bone* 2014;64:254-62.

88. Nordström A, Olsson T, Nordström P. Sustained Benefits from Previous Physical Activity on Bone Mineral Density in Males. *J Clin Endocrinol Metab* 2006;91(7):2600-04. doi: 10.1210/jc.2006-0151

89. Guyatt GH, Oxman AD, Kunz R, et al. What is "quality of evidence" and why is it important to clinicians? *BMJ* 2008;336(7651):995-98. doi: 10.1136/bmj.39490.551019.be

90. Guyatt GH, Oxman AD, Vist GE, et al. GRADE: an emerging consensus on rating quality of evidence and strength of recommendations. *BMJ* 2008;336(7650):924-26. doi: 10.1136/bmj.39489.470347.ad

91. Guyatt G, Oxman AD, Akl EA, et al. GRADE guidelines: 1. Introduction-GRADE evidence profiles and summary of findings tables. *J Clin Epidemiol* 2011;64(4):383-94. doi: 10.1016/j.jclinepi.2010.04.026 [published Online First: 20101231]

92. Grimshaw J. A Guide to Knowledge Synthesis: A Knowledge Synthesis Chapter: Canadian Institutes of Health Research, 2020.

93. Peters MDJ, Marnie C, Tricco AC, et al. Updated methodological guidance for the conduct of scoping reviews. *JBI Evid Synth* 2020;18(10):2119-26. doi: 10.11124/jbies-20-00167

94. Khalil H, Peters MD, Tricco AC, et al. Conducting high quality scoping reviews-challenges and solutions. *J Clin Epidemiol* 2021;130:156-60. doi: 10.1016/j.jclinepi.2020.10.009

95. Peters MDJ, Godfrey C, McInerney P, et al. Best practice guidance and reporting items for the development of scoping review protocols. *JBI Evid Synth* 2022;20(4)

96. Shamseer L, Moher D, Clarke M, et al. Preferred reporting items for systematic review and meta-analysis protocols (PRISMA-P) 2015: elaboration and explanation. *BMJ* 2015;349(jan02 1):g7647-g47. doi: 10.1136/bmj.g7647

For peer review only - http://bmjopen.bmj.com/site/about/guidelines.xhtml

97. Moher D, Shamseer L, Clarke M, et al. Preferred reporting items for systematic review and meta-analysis protocols (PRISMA-P) 2015 statement. *Syst Rev* 2015;4(1):1. doi: 10.1186/2046-4053-4-1

98. Tricco AC, Lillie E, Zarin W, et al. PRISMA Extension for Scoping Reviews (PRISMA-ScR): checklist and explanation. *Ann Intern Med* 2018;169(7):467-73. doi: 10.7326/m18-0850

99. Levac D, Colquhoun H, O'Brien KK. Scoping studies: advancing the methodology. *Implement Sci* 2010;5(1):69. doi: 10.1186/1748-5908-5-69

100. Pham MT, Rajić A, Greig JD, et al. A scoping review of scoping reviews: advancing the approach and enhancing the consistency. *Res Synth Methods* 2014;5(4):371-85. doi: 10.1002/jrsm.1123

101. Munn Z, Peters MDJ, Stern C, et al. Systematic review or scoping review? Guidance for authors when choosing between a systematic or scoping review approach. *BMC Med Res Methodol* 2018;18(1):143. doi: 10.1186/s12874-018-0611-x [published Online First: 2018/11/21]

102. Watkins R, Young G, Western M, et al. Nobody says to you "come back in six months and we'll see how you're doing": a qualitative interview study exploring young adults' experiences of sport-related knee injury. *BMC Musculoskelet Disord* 2020;21(1) doi: 10.1186/s12891-020-03428-6

103. Arksey H, O'Malley L. Scoping studies: towards a methodological framework. *Int J Soc Res Methodol* 2005;8(1):19-32. doi: 10.1080/1364557032000119616

104. Helman CG. Disease versus illness in general practice. *J R Coll Gen Pract* 1981;31(230):548-52.

105. Caspersen CJ, Powell KE, Christenson GM. Physical activity, exercise, and physical fitness: definitions and distinctions for health-related research. *Public Health Rep* 1985;100(2):126-31. [published Online First: 1985/03/01]

106. Bricca A, Struglics A, Larsson S, et al. Impact of exercise therapy on molecular biomarkers related to cartilage and inflammation in individuals at risk of, or with established, knee osteoarthritis: a systematic review and meta-analysis of randomized controlled trials. *Arthritis Care Res (Hoboken)* 2019;71(11):1504-15. doi: 10.1002/acr.23786

107. Stannus O, Jones G, Blizzard L, et al. Associations between serum levels of inflammatory markers and change in knee pain over 5 years in older adults: a prospective cohort study. *Ann Rheum Dis* 2013;72(4):535-40. doi: 10.1136/annrheumdis-2011-201047

108. Stannus O, Jones G, Cicuttini F, et al. Circulating levels of IL-6 and TNF-alpha are associated with knee radiographic osteoarthritis and knee cartilage loss in older adults. *Osteoarthritis Cartilage* 2010;18(11):1441-7. doi: 10.1016/j.joca.2010.08.016 [published Online First: 2010/09/08]

109. Ruan G, Xu J, Wang K, et al. Associations between knee structural measures, circulating inflammatory factors and MMP13 in patients with knee osteoarthritis. *Osteoarthritis Cartilage* 2018;26(8):1063-69. doi: 10.1016/j.joca.2018.05.003 [published Online First: 2018/05/14]

For peer review only - http://bmjopen.bmj.com/site/about/guidelines.xhtml

110. Kobayashi M, Squires GR, Mousa A, et al. Role of interleukin-1 and tumor necrosis factor α in matrix degradation of human osteoarthritic cartilage. *Arthritis Rheum* 2005;52(1):128-35. doi: 10.1002/art.20776

111. Wojdasiewicz P, Poniatowski LA, Szukiewicz D. The role of inflammatory and anti-inflammatory cytokines in the pathogenesis of osteoarthritis. *Mediators Inflamm* 2014; 2014. https://www.hindawi.com/journals/mi/2014/561459/

https://downloads.hindawi.com/journals/mi/2014/561459.pdf (accessed 03/02/2021).

112. Williams TD, Tolusso DV, Fedewa MV, et al. Comparison of Periodized and Non-Periodized Resistance Training on Maximal Strength: A Meta-Analysis. *Sports Med* 2017;47(10):2083-100. doi: 10.1007/s40279-017-0734-y

113. Shen W, Wang Z, Punyanita M, et al. Adipose tissue quantification by imaging methods: a proposed classification. *Obes Res* 2003;11(1):5-16. doi: 10.1038/oby.2003.3 [published Online First: 2003/01/17]

114. Thompson D, Karpe F, Lafontan M, et al. Physical Activity and Exercise in the Regulation of Human Adipose Tissue Physiology. *Physiol Rev* 2012;92(1):157-91. doi: 10.1152/physrev.00012.2011

115. Addison O, Marcus RL, Lastayo PC, et al. Intermuscular fat: a review of the consequences and causes. *Int J Endocrinol* 2014;2014:309570. doi: 10.1155/2014/309570 [published Online First: 2014/02/15]

116. Wisdom KM, Delp SL, Kuhl E. Use it or lose it: multiscale skeletal muscle adaptation to mechanical stimuli. *Biomech Model Mechanobiol* 2015;14(2):195-215. doi: 10.1007/s10237-014-0607-3 [published Online First: 2014/09/10]

117. McGowan J, Sampson M, Salzwedel DM, et al. PRESS Peer Review of Electronic Search Strategies: 2015 Guideline Statement. *J Clin Epidemiol* 2016;75:40-46. doi: 10.1016/j.jclinepi.2016.01.021

118. Chambat P, Guier C, Sonnery-Cottet B, et al. The evolution of ACL reconstruction over the last fifty years. *Int Orthop* 2013;37(2):181-86. doi: 10.1007/s00264-012-1759-3

119. Di Matteo B, Moran CJ, Tarabella V, et al. A history of meniscal surgery: from ancient times to the twenty-first century. *Knee Surg Sports Traumatol Arthrosc* 2016;24(5):1510-18. doi: 10.1007/s00167-015-3717-2

120. Peters MD, Godfrey CM, Khalil H, et al. Guidance for conducting systematic scoping reviews. *Int J Evid Based Healthc* 2015;13(3):141-46.

121. Paez A. Grey literature: An important resource in systematic reviews. *J Evid Based Med* 2017 doi: 10.1111/jebm.12265

122. Kellgren JH, Lawrence JS. Radiological Assessment of Osteo-Arthrosis. *Ann Rheum Dis* 1957;16(4):494-502. doi: 10.1136/ard.16.4.494

For peer review only

123. Altman RD, Gold GE. Atlas of individual radiographic features in osteoarthritis, revised. *Osteoarthritis Cartilage* 2007;15:A1-A56. doi: 10.1016/j.joca.2006.11.009

124. Hunter DJ, Guermazi A, Lo GH, et al. Evolution of semi-quantitative whole joint assessment of knee OA: MOAKS (MRI Osteoarthritis Knee Score). *Osteoarthritis Cartilage* 2011;19(8):990-1002. doi: 10.1016/j.joca.2011.05.004 [published Online First: 2011/06/08]

125. Peterfy CG, Guermazi A, Zaim S, et al. Whole-Organ Magnetic Resonance Imaging Score (WORMS) of the knee in osteoarthritis. *Osteoarthritis Cartilage* 2004;12(3):177-90. doi: 10.1016/j.joca.2003.11.003

126. Hunter DJ, Lo GH, Gale D, et al. The reliability of a new scoring system for knee osteoarthritis MRI and the validity of bone marrow lesion assessment: BLOKS (Boston Leeds Osteoarthritis Knee Score). *Ann Rheum Dis* 2008;67(2):206-11. doi: 10.1136/ard.2006.066183 [published Online First: 2007/05/03]

127. Eckstein F, Cicuttini F, Raynauld JP, et al. Magnetic resonance imaging (MRI) of articular cartilage in knee osteoarthritis (OA): morphological assessment. *Osteoarthritis Cartilage* 2006;14:46-75. doi: 10.1016/j.joca.2006.02.026

128. Dunn TC, Lu Y, Jin H, et al. T2 relaxation time of cartilage at MR imaging: comparison with severity of knee osteoarthritis. *Radiology* 2004;232(2):592-8. doi: 10.1148/radiol.2322030976 [published Online First: 20040623]

129. Burstein D, Velyvis J, Scott KT, et al. Protocol issues for delayed Gd(DTPA)2--enhanced MRI (dGEMRIC) for clinical evaluation of articular cartilage. *Magn Reson Med* 2001;45(1):36-41. doi: 10.1002/1522-2594(200101)45:1<36::aid-mrm1006>3.0.co;2-w

130. Bashir A, Gray ML, Boutin RD, et al. Glycosaminoglycan in articular cartilage: in vivo assessment with delayed Gd(DTPA)(2-)-enhanced MR imaging. *Radiology* 1997;205(2):551-8. doi: 10.1148/radiology.205.2.9356644

131. Moller I, Bong D, Naredo E, et al. Ultrasound in the study and monitoring of osteoarthritis. *Osteoarthritis Cartilage* 2008;16:S4-7. doi: 10.1016/j.joca.2008.06.005 [published Online First: 2008/09/02]

132. Naredo E, Acebes C, Moller I, et al. Ultrasound validity in the measurement of knee cartilage thickness. *Ann Rheum Dis* 2009;68(8):1322-7. doi: 10.1136/ard.2008.090738 [published Online First: 2008/08/08]

133. Schmitz RJ, Wang HM, Polprasert DR, et al. Evaluation of knee cartilage thickness: A comparison between ultrasound and magnetic resonance imaging methods. *The Knee* 2017;24(2):217-23. doi: 10.1016/j.knee.2016.10.004 [published Online First: 2016/12/05]

134. Kraus VB, Collins JE, Hargrove D, et al. Predictive validity of biochemical biomarkers in knee osteoarthritis: data from the FNIH OA Biomarkers Consortium. *Ann Rheum Dis* 2017;76(1):186-95. doi: 10.1136/annrheumdis-2016-209252 [published Online First: 2016/06/15]

For peer review only - http://bmjopen.bmj.com/site/about/guidelines.xhtml

135. Roberts HM, Law RJ, Thom JM. The time course and mechanisms of change in biomarkers of joint metabolism in response to acute exercise and chronic training in physiologic and pathological conditions. *Eur J Appl Physiol* 2019;119(11-12):2401-20. doi: 10.1007/s00421-019-04232-4 [published Online First: 2019/10/28]

136. Bay-Jensen AC, Mobasheri A, Thudium CS, et al. Blood and urine biomarkers in osteoarthritis–an update on cartilage associated type II collagen and aggrecan markers. *Curr Opin Rheumatol* 2022;34(1):54-60.

137. Engbersen MP, Huang Z, Kraus VB. Bone Biomarkers Related to Osteoarthritis: Springer Netherlands 2017:993-1021.

138. Van Spil WE, Degroot J, Lems WF, et al. Serum and urinary biochemical markers for knee and hip-osteoarthritis: a systematic review applying the consensus BIPED criteria. *Osteoarthritis and cartilage* 2010;18(5):605-12. doi: 10.1016/j.joca.2010.01.012

139. Li T, Vedula SS, Hadar N, et al. Innovations in data collection, management, and archiving for systematic reviews. *Ann Intern Med* 2015;162(4):287-94.

140. Harris PA, Taylor R, Minor BL, et al. The REDCap consortium: Building an international community of software platform partners. *J Biomed Inform* 2019;95:103208. doi: 10.1016/j.jbi.2019.103208 [published Online First: 2019/05/13]

141. Cohen J. A coefficient of agreement for nominal scales. *Educational and psychological measurement* 1960;20(1):37-46.

142. McHugh ML. Interrater reliability: the kappa statistic. *Biochem Med (Zagreb)* 2012;22(3):276-82.

143. Buscemi N, Hartling L, Vandermeer B, et al. Single data extraction generated more errors than double data extraction in systematic reviews. *J Clin Epidemiol* 2006;59(7):697-703. doi: 10.1016/j.jclinepi.2005.11.010

144. Meursinge Reynders R, Ladu L, Di Girolamo N. Contacting of authors modified crucial outcomes of systematic reviews but was poorly reported, not systematic, and produced conflicting results. *J Clin Epidemiol* 2019;115:64-76. doi: 10.1016/j.jclinepi.2019.07.001

145. Mullan RJ, Flynn DN, Carlberg B, et al. Systematic reviewers commonly contact study authors but do so with limited rigor. *J Clin Epidemiol* 2009;62(2):138-42. doi: 10.1016/j.jclinepi.2008.08.002

146. Higgins JPT TJ, Chandler J, Cumpston M, Li T, Page MJ, Welch VA (editors). Chapter 5: Collecting data. In: Li T HJ, Deeks JJ (editors), ed. Cochrane Handbook for Systematic Reviews of Interventions version 63 (updated February 2022): Cochrane, 2022.

147. Aromataris E, Fernandez R, Godfrey CM, et al. Summarizing systematic reviews: methodological development, conduct and reporting of an umbrella review approach. *Int J Evid Based Healthc* 2015;13(3):132-40. doi: 10.1097/XEB.0000000000000055

For peer review only

For peer review only - http://bmjopen.bmj.com/site/about/guidelines.xhtml

148. Tufanaru C, Munn Z, Aromataris E, et al. Chapter 3: Systematic reviews of effectiveness. In: Aromataris E, Munn Z, eds. JBI Manual for Evidence Synthesis: JBI, 2020.

149. Munn Z, Moola S, Lisy K, et al. Methodological guidance for systematic reviews of observational epidemiological studies reporting prevalence and cumulative incidence data. *Int J Evid Based Healthc* 2015;13(3):147-53. doi: 10.1097/XEB.0000000000000054

150. Moola S, Munn Z, Tufanaru C, et al. Chapter 7: Systematic reviews of etiology and risk. In: Aromataris E, Munn Z, eds. JBI Manual for Evidence Synthesis: JBI, 2020.

151. Maher JM, Markey JC, Ebert-May D. The Other Half of the Story: Effect Size Analysis in Quantitative Research. *CBE Life Sci Educ* 2013;12(3):345-51. doi: 10.1187/cbe.13-04-0082

152. Rosenthal JA. Qualitative Descriptors of Strength of Association and Effect Size. *J Soc Serv Res* 1996;21(4):37-59. doi: 10.1300/j079v21n04_02

153. Cohen J. A power primer. *Psychological bulletin* 1992;112(1):155.

154. Cohen J. Statistical Power Analysis for the Behavioral Sciences. 2nd Edition ed. New York: Routledge 1988.

155. Sonnery-Cottet B, Saithna A, Quelard B, et al. Arthrogenic muscle inhibition after ACL reconstruction: a scoping review of the efficacy of interventions. *Br J Sports Med* 2019;53(5):289-98. doi: 10.1136/bjsports-2017-098401

156. Fedak KM, Bernal A, Capshaw ZA, et al. Applying the Bradford Hill criteria in the 21st century: how data integration has changed causal inference in molecular epidemiology. *Emerg Themes Epidemiol* 2015;12(1) doi: 10.1186/s12982-015-0037-4

157. Hill AB. The Enviroment and Disease: Association or Causation? *Proc R Soc Med* 1965;58(5):295-300.

158. Schünemann H, Hill S, Guyatt G, et al. The GRADE approach and Bradford Hill's criteria for causation. *J Epidemiol Community Health* 2011;65(5):392. doi: 10.1136/jech.2010.119933

For peer review only

For peer review only - http://bmjopen.bmj.com/site/about/guidelines.xhtml

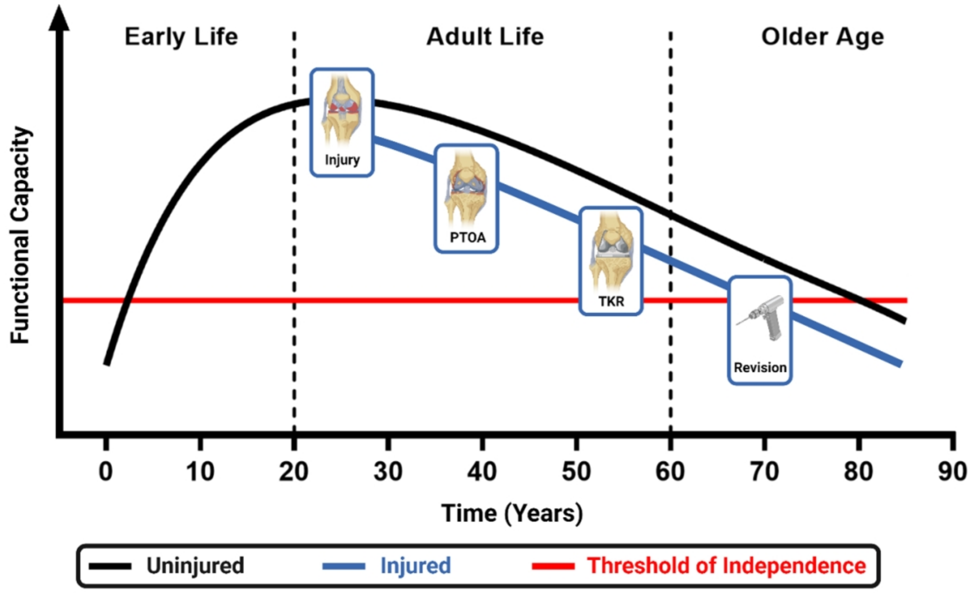

Figure 1. Hypothetical trajectory of injury to lifelong disease burden. Incidence of knee injury leads to post-traumatic osteoarthritis (PTOA),[28-31] which leads to earlier age for total knee replacement (TKR),[34] which increases the risk of revision surgery.[38] This accelerates the loss of knee functional capacity, which could impact the ability to complete self-sustaining tasks.

402x244mm (87 x 87 DPI)

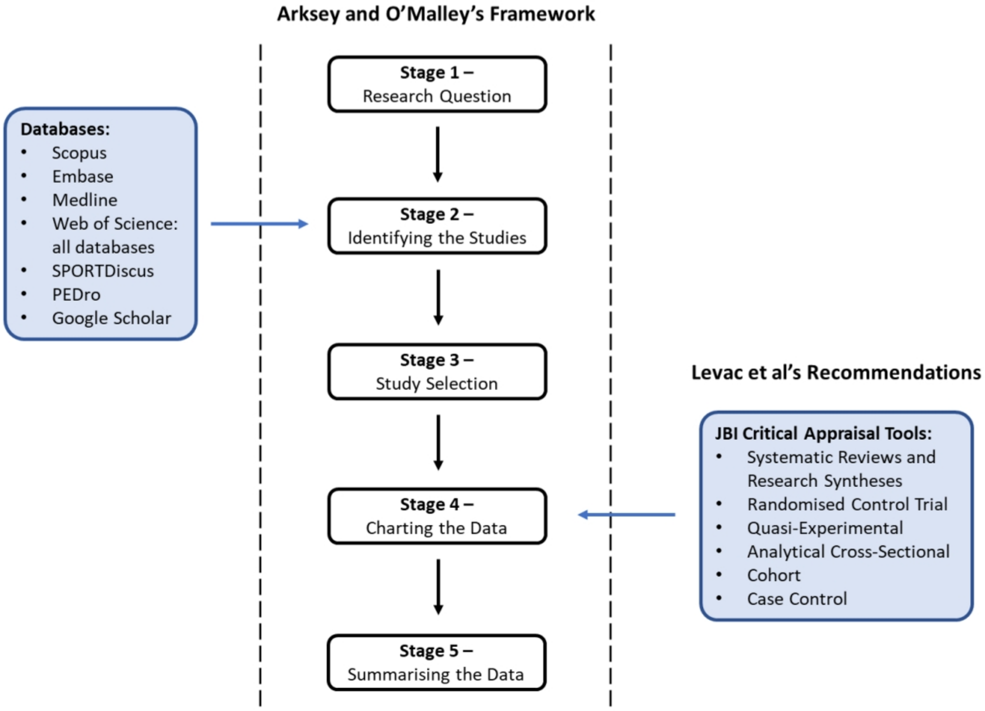

Figure 2. Outline of the study methodology for the present review incorporating Arksey and O'Malley's[103] framework and Levac's[99] recommendations (inclusion of methodological quality appraisal) for conducting a scoping review. JBI denotes the Joanna Briggs Institute.

638x462mm (59 x 59 DPI)

Supplementary Material 1. Preferred Reporting Items for Systematic review and Meta-Analysis Protocols 2015 Checklist[1 2] Adapted for Scoping Reviews[3]

Section and topic	Item No	Checklist item	
**ADMINISTRATIVE INFORMATION**			
Protocol title:			
Identification	1a	Identify the report as a protocol of a scoping review	- Study title<br>- Page 6 of main manuscript
Update	1b	If the protocol is for an update of a previous scoping review, identify as such	Not applicable
Registration	2	If registered, provide the name of the registry (such as Open Science Framework, Figshare or Research Gate) and registration number	- Abstract, page 3 of main manuscript<br>- Methods, page 6 of the main manuscript
Authors:			
Contact	3a	Provide name, institutional affiliation, e-mail address of all protocol authors; provide physical mailing address of corresponding author	Title page, page 1 of main manuscript
Contributions	3b	Describe contributions of protocol authors and identify the guarantor of the review	CRediT statement, page 14 of main manuscript
Amendments	4	If the protocol represents an amendment of a previously completed or published protocol, identify as such and list changes; otherwise, state plan for documenting important protocol amendments	Not applicable
Support:			
Sources	5a	Indicate sources of financial or other support for the review	- Funding statement, page 15 of main manuscript<br>- Acknowledgements, page 15 of main manuscript
Sponsor	5b	Provide name for the review funder and/or sponsor	Funding statement, page 15 of main manuscript
Role of sponsor or funder	5c	Describe roles of funder(s), sponsor(s), and/or institution(s), if any, in developing the protocol	Not applicable

For peer review only

**INTRODUCTION**			
Rationale	6	Describe the rationale for the review in the context of what is already known	Introduction, pages 4-6 of the main manuscript
Objectives	7	Provide an explicit statement of the question(s) the review will address with reference to the inclusion/exclusion criteria	- Introduction, review aims, page 6 of the main manuscript<br>- Methods, framework, stage 1, pages 7-8 of the main manuscript
**METHODS**			
Eligibility criteria	8	Specify the study characteristics (such as PICO/PCC or other, study design, setting, time frame) and report characteristics (such as years considered, language, publication status) to be used as criteria for eligibility for the review	- Table 1., review definitions, page 8-9 of main manuscript<br>- Table 2., review inclusion and exclusion criteria, page 10 of main manuscript
Information sources	9	Describe all intended information sources (such as electronic databases, contact with study authors, trial registers or other grey literature sources) with planned dates of coverage	- Abstract, methods, page 2 of main manuscript<br>- Methods, framework, stage 2, page 9 of main manuscript
Search strategy	10	Present draft of search strategy to be used for at least one electronic database, including planned limits, such that it could be repeated	Supplementary material 2
Study records:			
Data management	11a	Describe the mechanism(s) that will be used to manage records and data throughout the review	- Supplementary material 4, 5, 7, 8<br>- Pages 11-13 of main manuscript
Selection process	11b	State the process that will be used for selecting studies (such as two independent reviewers) through each phase of the review (that is, screening, eligibility, and inclusion)	Pages 11-13 of main manuscript
Data collection process	11c	Describe planned method of extracting data from reports (such as piloting forms, done independently, in duplicate), any processes for obtaining and confirming data from investigators	Pages 11-13 of main manuscript
Data items	12	List and define all variables for which data will be sought (such as PICO/PCC or other items, funding sources), any pre-planned data assumptions and simplifications	Supplementary material 3

For peer review only

Outcomes and prioritization	13	List and define all outcomes for which data will be sought, including prioritization of main and additional outcomes, with rationale (applicable if extracting outcomes)	Supplementary material 3
Risk of bias in individual studies	14	Describe anticipated methods for assessing risk of bias of individual studies, including whether this will be done at the outcome or study level, or both; state how this information will be used in data synthesis	Supplementary material 7
Data synthesis	15a	Describe criteria under which study data will be presented	Methods, framework, stage 4, page 13 of main manuscript
	15b	Describe the planned approach to how, extracted data will be presented (such as figures, tables, evidence gaps maps)	Methods, framework, stage 4 and stage 5, page 13 of main manuscript
	15c	Describe any proposed additional analyses (such as thematic analyses)	Methods, framework, stage 5, pages 13-14 of main manuscript
	15d	If quantitative synthesis is not appropriate, describe the type of summary planned	Methods, framework, stage 5, pages 13-14 of main manuscript
Meta-bias(es)	16	Specify any planned assessment of meta-bias(es) (such as publication bias across studies, selective reporting within studies)	Not applicable
Confidence in cumulative evidence	17	Describe how the strength of the body of evidence will be assessed (such as GRADE)	Methods, framework, stage 5, pages 13-14 of main manuscript

1. Moher D, Shamseer L, Clarke M, et al. Preferred reporting items for systematic review and meta-analysis protocols (PRISMA-P) 2015 statement. *Syst Rev* 2015;4(1):1. doi: 10.1186/2046-4053-4-1
2. Shamseer L, Moher D, Clarke M, et al. Preferred reporting items for systematic review and meta-analysis protocols (PRISMA-P) 2015: elaboration and explanation. *BMJ* 2015;349(jan02 1):g7647-g47. doi: 10.1136/bmj.g7647
3. Peters MDJ, Godfrey C, McInerney P, et al. Best practice guidance and reporting items for the development of scoping review protocols. *JBI Evid Synth* 2022;20(4)

For peer review only

PubMed Search Strategy

The search terms are structured as follows:

Population AND independent variables (e.g., physical activity, adiposity, knee joint loading) AND outcome variables (e.g., osteoarthritis, biomarkers of cartilage or bone metabolism)

Search Terms

(("knee injur*" OR "cruciate ligament injur*" OR "cruciate ligament tear" OR "cruciate ligament damage*" OR "cruciate ligament rupture*" OR “injured cruciate ligament” OR "torn cruciate ligament" OR “damaged cruciate ligament” OR “ruptured cruciate ligament” OR “ACL injury” OR “ACL tear” OR “ACL damage” OR “ACL rupture” OR “injured ACL” OR “torn ACL” OR “damaged ACL” OR “ruptured ACL” OR “PCL injury” OR “PCL tear” OR “PCL damage” OR “PCL rupture” OR “injured PCL” OR “torn PCL” OR “PCL damage” OR “ruptured PCL” OR "cruciate ligament reconstruct*" OR "cruciate ligament surger*" OR “cruciate ligament repair” OR “ACL reconstr*” OR “ACL surger*” OR “PCL reconstr*” OR “PCL surger*” OR "meniscus injur*" OR "meniscal injur*" OR "meniscus tear" OR "meniscal tear" OR "meniscus damage" OR "meniscal damage" OR "meniscus rupture" OR "meniscal rupture" OR “injured menisc*” OR “torn menisc*” OR "meniscus repair" OR “repaired menis*” OR "meniscus surger*" OR Anterior Cruciate Ligament Injuries[MeSH Terms] OR Anterior Cruciate Ligament Reconstruction[MeSH Terms] OR Posterior Cruciate Ligament Reconstruction[MeSH Terms] OR Tibial Meniscus Injuries[MeSH Terms] OR Meniscectomy[MeSH Terms]) AND ("physical activit*" OR "physical inactivit*" OR exercis* OR "rehab*" OR train* OR inflamma* OR "c reactive protein" OR cytokine* OR interleukin OR "reaction force*" OR "contact force*" OR "knee load*" OR "cumulative load*" OR "knee adduction moment" OR "knee abduction moment" OR "knee flexion moment" OR "knee extension moment" OR "contact pressure" OR "ground reaction force*" OR "musculoskeletal model*" OR "inverse dynamics" OR simulat* OR "muscle strength" OR "muscle contract*" OR neurophysiolog* OR neuromuscular OR "body composition" OR "body mass inde*" OR adipos* OR obes* OR fat OR "muscle quality" OR "echo intensity" OR echogenicity OR "inter-muscular adipose" OR "intermuscular adipose" OR "intermuscular fat" OR "intra-muscular adipose" OR "intramuscular adipose" OR "intra-muscular fat" OR "intramuscular fat" OR "muscle attenuation" OR "muscle density" OR "muscle mass" OR "muscle volume" OR "muscle cross-sectional area" OR "muscle thickness" OR "muscle atrophy" OR "bone mineral" OR "bone density" OR Exercise[MeSH Terms] OR Sedentary Behaviour[MeSH Terms] OR Rehabilitation[MeSH Terms] OR Inflammation[MeSH Terms] OR Cytokines[MeSH Terms] OR Interleukins[MeSH Terms] OR Muscle Strength[MeSH Terms] OR Muscle Contraction[MeSH Terms] OR Muscle Weakness[MeSH Terms] OR Body Composition[MeSH Terms] OR Body Mass Index[MeSH Terms] OR Subcutaneous Fat[MeSH Terms] OR Adipose Tissue[MeSH Terms] OR Obesity[MeSH Terms] OR Leptin[MeSH Terms] OR Adiponectin[MeSH Terms] OR Muscular Atrophy[MeSH Terms] OR Bone Density[MeSH Terms]) AND (osteoarthr* OR chondrosis OR “degenerative arthritis” OR "t1 relaxation time" OR t1rho OR "t2 relaxation time" OR t2 OR "delayed gadolinium-enhanced magnetic resonance imaging of cartilage" OR dGEMRIC OR “cartilage volume” OR "cartilage thickness" OR matrix OR MMP OR collagen OR “pro-collagen” OR procollagen OR collagenase OR gelatinase OR proteoglycan OR hyaluronic OR “keratin

For peer review only - http://bmjopen.bmj.com/site/about/guidelines.xhtml

sulphate" OR chondroitin OR pyridinoline OR "alkaline phosphatase bone isoenzyme" OR "bone alkaline phosphatase" OR "tartrate-resistant acid phosphatase 5b" OR osteocalcin OR deoxypyridinoline OR osteophyte* OR "bone remodel*" OR "bone regenerat*" OR 'insulin growth factor 1' OR 'transforming growth factor beta' OR gene* OR allele OR polymorphism OR variant OR expression OR proteom* OR "mass spectrometry" OR stromelysin OR 'a disintegrin and metalloproteinase with thrombospondin motifs' OR ADAMTS OR metaboli* OR biomarker* OR "blood marker" OR "urine marker" OR "synovial fluid marker" OR biochemical OR Osteoarthritis, Knee[MeSH Terms] OR Fibrillar Collagens[MeSH Terms] OR Proteoglycans[MeSH Terms] OR Bone Remodelling[MeSH Terms] OR Bone Regeneration[MeSH Terms] OR Osteogenesis[MeSH Terms] OR Osteophytes[MeSH Terms] OR Biomarkers[MeSH Terms]) NOT (Review[Publication Type] OR Systematic Review[Publication Type] OR Meta Analysis[Publication Type] OR Scoping Review[Publication Type] OR Aged[MeSH Terms] OR Arthroplasty, Replacement, Knee[MeSH Terms] OR (animals[mh] NOT humans[mh])))

Filters Applied: Results by Year: 1970-2023, Species: Human, Language: English

Number of Records: 2,810

For peer review only - http://bmjopen.bmj.com/site/about/guidelines.xhtml

Supplementary Material 3. PTOA Disease Outcomes

This list of outcome measures is not exhaustive and will be added to if other relevant outcomes are identified during Stage 2 - Identifying Relevant Studies.

Table 1. Disease Outcome Measures

**Diagnoses of Knee PTOA Disease**	
**Radiographic Diagnostic System**	**Scoring System**
Kellgren and Lawrence (K/L) system[1]	A K/L score of 1 indicates early knee OA and an >1 K/L score indicates knee OA[1]
Osteoarthritis Research Society International (OARSI) Atlas criteria[2]	OARSI Atlas scores for knee OA diagnosis are joint space narrowing ≥ grade 2, or grade 1 joint space narrowing occurred in combination with grade 1 osteophyte[2]
International Knee Documentation Committee (IKDC) System[3]	An IKDC score from C-D indicates reduction in joint space, development of small osteophytes, slight sclerosis, or femoral condyle flattening[3]
Ahlback System[4]	An Ahlback score of >0 indicates development of osteoarthritic features[4]
Fairbank Classification[5]	A Fairbank classification of >0 indicates development of osteoarthritic features[5]
Brandt Grading Scale[6]	
Jager-Wirth Classification[7]	A Jager-Wirth Classification of >0 indicates development of osteoarthritic features[7]
**Magnetic Resonance Imaging Diagnostic System**	**Scoring System**
Magnetic Resonance Imaging Osteoarthritis Knee Score (MOAKS)[8]	For brevity due to the multi-tissue assessment lower scores for each tissue inside the joint indicates degeneration.[8]
Whole Organ Magnetic Resonance Imaging Score (WORMS)[9]	For brevity due to the multi-tissue assessment, lower scores for each tissue inside the joint indicates degeneration.[9]
Boston Leeds OA Knee Score (BLOKS)[10]	For brevity due to the multi-tissue assessment, lower scores for each tissue inside the joint indicates degeneration.[10]

For peer review only

Indicators of Structural Deterioration Preceding PTOA Disease

Measurement Tool	Index
Magnetic resonance imaging (MRI) quantified cartilage thickness and volume[11]	Lower MRI cartilage thickness and volume indicates degenerated cartilage[11 12]
T1rho mapping[12]	Higher signal intensity of T1rho mapping indicates lower collagen and/or proteoglycan content[12 13]
T2 mapping[14]	Higher signal intensity of T2 mapping indicates lower collagen and/or proteoglycan content[12 13]
Delayed contrast-enhanced MRI of cartilage (dGEMRIC)[15]	Higher signal intensity for dGEMRIC is indicative of low glycosaminoglycan (GAG) content[12 15]
Ultrasonography (US) quantified cartilage thickness[16-18]	Lower US cartilage thickness and volume indicates degenerated cartilage[16-18]

Systemic Biomarkers Indicating Structural Deterioration of Knee PTOA Disease

Substrate Biomarkers	Index
Matrix Metalloprotease 3 (MMP-3)[19]	Serum MMP-3 concentration positively associated with knee OA and cartilage volume loss[20-22]
Matrix Metalloprotease 9 (MMP-9)[23]	Serum and synovial fluid MMP-9 concentration positively associated with OA diagnosis[24]
Matrix Metalloprotease 13 (MMP-13) [25-29]	Serum MMP-13 concentration negatively associated with knee cartilage volume and positively associated with knee OA diagnosis[22 30]
A Disintegrin and Metalloproteinase with Thrombospondin Motifs 4 (ADAMTS-4)[31]	Serum ADAMTS-4 concentration positively associated with knee OA diagnosis[32]
Tissue Inhibitor of Metalloproteinase 1 (TIMP-1)[33]	Plasma TIMP-1 concentration positively associated with OA severity[34]
**Collagen Biomarker**	**Index**
N-Propeptide of Collagen IIA (PIIANP)[35]	Serum PIIANP concentration negatively associated with knee osteophytes[24] and knee OA[35 36]
N-Propeptide of Collagen IIB (PIIBNP)[37]	Serum and Plasma concentrations negatively associated with joint space narrowing[38]
C-Telopeptide of Type II Collagen (CTX-II)[39]	Urinary concentration of CTX-II positively associated with knee OA diagnosis and cartilage degeneration[21 40-44]
C-Propeptide of Type II Collagen (CP-II) [45]	Serum concentration of CP-II negatively associated with knee OA diagnosis[45 46]

For peer review only

For peer review only - http://bmjopen.bmj.com/site/about/guidelines.xhtml

CTX-II:CP-II[46]	Ratio of urinary concentration of CTX-II to serum concentrations of CP-II positively associated with knee OA[35 46]
Collagen Type II Cleavage Product (C2C)[47]	Serum and urine C2C concentration positively associated with cartilage degeneration and knee joint pain worsening and joint space narrowing[48-50]
Col2-3/4 C-terminal cleavage product of types I and II collagen (C1,2C)[25]	Serum C1,2C concentration positively associated with knee OA diagnosis[50]
Neoepitope of Collagen X (C10C or Col10Neo)[50]	Serum C10C concentration positively associated with knee OA diagnosis[51] and plasma C10C/Col10Neo concentration positively associated with knee OA diagnosis[52]
**Proteoglycan Biomarker**	**Index**
Hyaluronic Acid (HA)[53]	Serum HA concentration positively associated with knee OA diagnosis and joint space narrowing[21 48 54-56]
Chondroitin Sulphate 846 Epitope (CS846)[57]	Serum CS846 concentration positively associated with femur cartilage thickening [58 59]
Aggrecan[60] or Aggrecan Fragments (ARGS)[61 62]	Serum aggrecan concentration at baseline inversely associated with joint space narrowing in medial compartment after 10 years of follow-up[63] and synovial fluid ARGS concentration negatively associated with joint space narrowing[52 64]
Cartilage Oligomeric Matrix Protein (COMP)[53 65]	Serum COMP concentration positively associate with knee OA diagnosis and cartilage degeneration[21 41 43 44 50 63 66-70]
**Bone Formation**	**Index**
Osteocalcin (OC)[71]	Serum increase in OC concentration over a year positively associated with knee OA severity progression over three years[72]
Bone Specific Alkaline Phosphate (BAP) or alkaline phosphatase (ALP)[73]	Serum ALP concentrations positively associated with knee OA severity[74]
Procollagen Type I N-Terminal Propeptide (PINP)[75]	Baseline PINP serum concentration predicts OA progression[76]
**Bone Resorption**	**Index**
Pyridinoline (PYD)[71 77]	Urinary PYD concentration negatively associated with the joint space width and OA severity[34]
Deoxypyridinoline (DPD)[71]	Synovial fluid DPD concentration positively associated with disease severity[78]
N-Telopeptide of Type I Collagen (NTX-I)[79 80]	Urinary and serum NTX-I concentration positively associated with knee OA severity progression[48 81]
C-Telopeptide of Type I Collagen (CTX-I)[79]	Urinary and serum CTX-I concentration positively associated with knee OA severity progression[48]

For peer review only

For peer review only

References

1. Kellgren JH, Lawrence JS. Radiological Assessment of Osteo-Arthrosis. *Ann Rheum Dis* 1957;16(4):494-502. doi: 10.1136/ard.16.4.494
2. Altman RD, Gold GE. Atlas of individual radiographic features in osteoarthritis, revised. *Osteoarthritis Cartilage* 2007;15:A1-A56. doi: 10.1016/j.joca.2006.11.009
3. Hefti E, Müller W, Jakob RP, et al. Evaluation of knee ligament injuries with the IKDC form. *Knee Surgery, Sports Traumatology, Arthroscopy* 1993;1(3-4):226-34. doi: 10.1007/bf01560215
4. Ahlbäck S. Osteoarthrosis of the knee. A radiographic investigation. *Acta Radiol Diagn (Stockh)* 1968:Suppl 277:7-72.
5. Fairbank TJ. KNEE JOINT CHANGES AFTER MENISCECTOMY. *The Journal of Bone and Joint Surgery British volume* 1948;30-B(4):664-70. doi: 10.1302/0301-620X.30B4.664
6. Brandt KD, Fife RS, Braunstein EM, et al. Radiographic grading of the severity of knee osteoarthritis: Relation of the kellgren and lawrence grade to a grade based on joint space narrowing, and correlation with arthroscopic evidence of articular cartilage degeneration. *Arthritis & Rheumatism* 2010;34(11):1381-86. doi: 10.1002/art.1780341106
7. Scheller G, Sobau C, Bülow JU. Arthroscopic partial lateral meniscectomy in an otherwise normal knee: Clinical, functional, and radiographic results of a long-term follow-up study. *Arthroscopy* 2001;17(9):946-52. doi: 10.1053/jars.2001.28952
8. Hunter DJ, Guermazi A, Lo GH, et al. Evolution of semi-quantitative whole joint assessment of knee OA: MOAKS (MRI Osteoarthritis Knee Score). *Osteoarthritis Cartilage* 2011;19(8):990-1002. doi: 10.1016/j.joca.2011.05.004 [published Online First: 2011/06/08]
9. Peterfy CG, Guermazi A, Zaim S, et al. Whole-Organ Magnetic Resonance Imaging Score (WORMS) of the knee in osteoarthritis. *Osteoarthritis Cartilage* 2004;12(3):177-90. doi: 10.1016/j.joca.2003.11.003
10. Hunter DJ, Lo GH, Gale D, et al. The reliability of a new scoring system for knee osteoarthritis MRI and the validity of bone marrow lesion assessment: BLOKS (Boston Leeds Osteoarthritis Knee Score). *Ann Rheum Dis* 2008;67(2):206-11. doi: 10.1136/ard.2006.066183 [published Online First: 2007/05/03]
11. Eckstein F, Cicuttini F, Raynauld JP, et al. Magnetic resonance imaging (MRI) of articular cartilage in knee osteoarthritis (OA): morphological assessment. *Osteoarthritis Cartilage* 2006;14:46-75. doi: 10.1016/j.joca.2006.02.026
12. Blumenkrantz G, Majumdar S. Quantitative magnetic resonance imaging of articular cartilage in osteoarthritis. *Eur Cell Mater* 2007; 13. http://europepmc.org/abstract/MED/17506024

https://doi.org/10.22203/ecm.v013a08 (accessed 2007/05//).

13. Atkinson HF, Birmingham TB, Moyer RF, et al. MRI T2 and T1ρ relaxation in patients at risk for knee osteoarthritis: a systematic review and meta-analysis. *BMC Musculoskelet Disord* 2019;20(1):1-18.
14. Dunn TC, Lu Y, Jin H, et al. T2 relaxation time of cartilage at MR imaging: comparison with severity of knee osteoarthritis. *Radiology* 2004;232(2):592-8. doi: 10.1148/radiol.2322030976 [published Online First: 20040623]
15. Bashir A, Gray ML, Boutin RD, et al. Glycosaminoglycan in articular cartilage: in vivo assessment with delayed Gd(DTPA)(2-)-enhanced MR imaging. *Radiology* 1997;205(2):551-8. doi: 10.1148/radiology.205.2.9356644
16. Naredo E, Acebes C, Moller I, et al. Ultrasound validity in the measurement of knee cartilage thickness. *Ann Rheum Dis* 2009;68(8):1322-7. doi: 10.1136/ard.2008.090738 [published Online First: 2008/08/08]
17. Abraham AM, Goff I, Pearce MS, et al. Reliability and validity of ultrasound imaging of features of knee osteoarthritis in the community. *BMC Musculoskelet Disord* 2011;12(1):70. doi: 10.1186/1471-2474-12-70

For peer review only

18. Schmitz RJ, Wang HM, Polprasert DR, et al. Evaluation of knee cartilage thickness: A comparison between ultrasound and magnetic resonance imaging methods. *The Knee* 2017;24(2):217-23. doi: 10.1016/j.knee.2016.10.004 [published Online First: 2016/12/05]
19. Okada Y, Shinmei M, Tanaka O, et al. Localization of matrix metalloproteinase 3 (stromelysin) in osteoarthritic cartilage and synovium. *Lab Invest* 1992;66(6):680-90. [published Online First: 1992/06/01]
20. Pelletier JP, Raynauld JP, Caron J, et al. Decrease in serum level of matrix metalloproteinases is predictive of the disease-modifying effect of osteoarthritis drugs assessed by quantitative MRI in patients with knee osteoarthritis. *Ann Rheum Dis* 2010;69(12):2095-101. doi: 10.1136/ard.2009.122002 [published Online First: 2010/06/24]
21. Joseph GB, Nevitt MC, McCulloch CE, et al. Associations between molecular biomarkers and MR-based cartilage composition and knee joint morphology: data from the Osteoarthritis Initiative. *Osteoarthritis Cartilage* 2018;26(8):1070-77. doi: 10.1016/j.joca.2018.04.019 [published Online First: 2018/05/29]
22. Pengas I, Eldridge S, Assiotis A, et al. MMP-3 in the peripheral serum as a biomarker of knee osteoarthritis, 40 years after open total knee meniscectomy. *J Exp Orthop* 2018;5(1):1-8.
23. Fosang AJ, Neame PJ, Last K, et al. The interglobular domain of cartilage aggrecan is cleaved by PUMP, gelatinases, and cathepsin B. *Journal of Biological Chemistry* 1992;267(27):19470-74. doi: https://doi.org/10.1016/S0021-9258(18)41799-1
24. Zeng GQ, Chen AB, Li W, et al. High MMP-1, MMP-2, and MMP-9 protein levels in osteoarthritis. *Genetics and Molecular Research* 2015;14(4):14811-22. doi: 10.4238/2015.november.18.46
25. Billinghurst RC, Dahlberg L, Ionescu M, et al. Enhanced cleavage of type II collagen by collagenases in osteoarthritic articular cartilage. *J Clin Invest* 1997;99(7):1534-45. doi: 10.1172/JCI119316 [published Online First: 1997/04/01]
26. Neuhold LA, Killar L, Zhao W, et al. Postnatal expression in hyaline cartilage of constitutively active human collagenase-3 (MMP-13) induces osteoarthritis in mice. *J Clin Invest* 2001;107(1):35-44. doi: 10.1172/JCI10564 [published Online First: 2001/01/03]
27. Shlopov BV, Lie WR, Mainardi CL, et al. Osteoarthritic lesions: involvement of three different collagenases. *Arthritis Rheum* 1997;40(11):2065-74. doi: 10.1002/art.1780401120 [published Online First: 1997/11/19]
28. Wang M, Sampson ER, Jin H, et al. MMP13 is a critical target gene during the progression of osteoarthritis. *Arthritis Res Ther* 2013;15(1):R5. doi: 10.1186/ar4133 [published Online First: 2013/01/10]
29. Hu Q, Ecker M. Overview of MMP-13 as a promising target for the treatment of osteoarthritis. *Int J Mol Sci* 2021;22(4):1742. doi: 10.3390/ijms22041742
30. Ruan G, Xu J, Wang K, et al. Associations between knee structural measures, circulating inflammatory factors and MMP13 in patients with knee osteoarthritis. *Osteoarthritis Cartilage* 2018;26(8):1063-69. doi: 10.1016/j.joca.2018.05.003 [published Online First: 2018/05/14]
31. Tortorella M, Pratta M, Liu R-Q, et al. The Thrombospondin Motif of Aggrecanase-1 (ADAMTS-4) Is Critical for Aggrecan Substrate Recognition and Cleavage. *Journal of Biological Chemistry* 2000;275(33):25791-97. doi: 10.1074/jbc.m001065200
32. Li W, Du C, Wang H, et al. Increased serum ADAMTS-4 in knee osteoarthritis: a potential indicator for the diagnosis of osteoarthritis in early stages. *Genetics and Molecular Research* 2014;13(4):9642-49. doi: 10.4238/2014.november.14.9
33. Woolley DE, Roberts DR, Evanson JM. Inhibition of human collagenase activity by a small molecular weight serum protein. *Biochem Biophys Res Commun* 1975;66(2):747-54. doi: 10.1016/0006-291x(75)90573-2
34. Takahashi M, Naito K, Abe M, et al. *Arthritis Research & Therapy* 2004;6(3):R208. doi: 10.1186/ar1166

35. Rousseau JC, Sandell LJ, Delmas PD, et al. Development and clinical application in arthritis of a new immunoassay for serum type IIA procollagen NH2 propeptide. *Methods Mol Med* 2004;101:25-37. doi: 10.1385/1-59259-821-8:025 [published Online First: 2004/08/10]
36. Daghestani HN, Jordan JM, Renner JB, et al. Serum N-propeptide of collagen IIA (PIIANP) as a marker of radiographic osteoarthritis burden. *PLoS One* 2017;12(12):e0190251. doi: 10.1371/journal.pone.0190251
37. Luo Y, He Y, Reker D, et al. A Novel High Sensitivity Type II Collagen Blood-Based Biomarker, PRO-C2, for Assessment of Cartilage Formation. *International Journal of Molecular Sciences* 2018;19(11):3485. doi: 10.3390/ijms19113485
38. Luo Y, Samuels J, Krasnokutsky S, et al. A low cartilage formation and repair endotype predicts radiographic progression of symptomatic knee osteoarthritis. *J Orthop Traumatol* 2021;22(1) doi: 10.1186/s10195-021-00572-0
39. Christgau S, Garnero P, Fledelius C, et al. Collagen type II C-telopeptide fragments as an index of cartilage degradation. *Bone* 2001;29(3):209-15. doi: 10.1016/s8756-3282(01)00504-x [published Online First: 2001/09/15]
40. Cheng H, Hao B, Sun J, et al. C-terminal cross-linked telopeptides of type II collagen as biomarker for radiological knee osteoarthritis: a meta-analysis. *Cartilage* 2020;11(4):512-20. doi: 10.1177/1947603518798884 [published Online First: 2018/09/18]
41. Valdes AM, Meulenbelt I, Chassaing E, et al. Large scale meta-analysis of urinary C-terminal telopeptide, serum cartilage oligomeric protein and matrix metalloprotease degraded type II collagen and their role in prevalence, incidence and progression of osteoarthritis. *Osteoarthritis Cartilage* 2014;22(5):683-9. doi: 10.1016/j.joca.2014.02.007 [published Online First: 2014/03/01]
42. Dam EB, Byrjalsen I, Karsdal MA, et al. Increased urinary excretion of C-telopeptides of type II collagen (CTX-II) predicts cartilage loss over 21 months by MRI. *Osteoarthritis Cartilage* 2009;17(3):384-9. doi: 10.1016/j.joca.2008.07.009 [published Online First: 2008/09/05]
43. Sowers MF, Karvonen-Gutierrez CA, Yosef M, et al. Longitudinal changes of serum COMP and urinary CTX-II predict X-ray defined knee osteoarthritis severity and stiffness in women. *Osteoarthritis Cartilage* 2009;17(12):1609-14. doi: 10.1016/j.joca.2009.06.001 [published Online First: 2009/07/01]
44. Hao HQ, Zhang JF, He QQ, et al. Cartilage oligomeric matrix protein, C-terminal cross-linking telopeptide of type II collagen, and matrix metalloproteinase-3 as biomarkers for knee and hip osteoarthritis (OA) diagnosis: a systematic review and meta-analysis. *Osteoarthritis Cartilage* 2019;27(5):726-36. doi: 10.1016/j.joca.2018.10.009 [published Online First: 2018/11/06]
45. Nelson F, Dahlberg L, Laverty S, et al. Evidence for altered synthesis of type II collagen in patients with osteoarthritis. *J Clin Invest* 1998;102(12):2115-25. doi: 10.1172/JCI4853 [published Online First: 1998/12/17]
46. Cibere J, Zhang H, Garnero P, et al. Association of biomarkers with pre-radiographically defined and radiographically defined knee osteoarthritis in a population-based study. *Arthritis Rheum* 2009;60(5):1372-80. doi: 10.1002/art.24473 [published Online First: 2009/05/01]
47. Poole AR, Ionescu M, Fitzcharles MA, et al. The assessment of cartilage degradation in vivo: development of an immunoassay for the measurement in body fluids of type II collagen cleaved by collagenases. *J Immunol Methods* 2004;294(1-2):145-53. doi: 10.1016/j.jim.2004.09.005 [published Online First: 2005/01/11]
48. Kraus VB, Collins JE, Hargrove D, et al. Predictive validity of biochemical biomarkers in knee osteoarthritis: data from the FNIH OA Biomarkers Consortium. *Ann Rheum Dis* 2017;76(1):186-95. doi: 10.1136/annrheumdis-2016-209252 [published Online First: 2016/06/15]
49. King KB, Lindsey CT, Dunn TC, et al. A study of the relationship between molecular biomarkers of joint degeneration and the magnetic resonance-measured characteristics of cartilage in 16

For peer review only

symptomatic knees. *Magn Reson Imaging* 2004;22(8):1117-23. doi: 10.1016/j.mri.2004.08.001
50. Kong SY, Stabler TV, Criscione LG, et al. Diurnal variation of serum and urine biomarkers in patients with radiographic knee osteoarthritis. *Arthritis Rheum* 2006;54(8):2496-504. doi: 10.1002/art.21977 [published Online First: 2006/07/27]
51. He Y, Siebuhr AS, Brandt-Hansen NU, et al. Type X collagen levels are elevated in serum from human osteoarthritis patients and associated with biomarkers of cartilage degradation and inflammation. *BMC Musculoskelet Disord* 2014;15(1):309. doi: 10.1186/1471-2474-15-309
52. He Y, Manon-Jensen T, Arendt-Nielsen L, et al. Potential diagnostic value of a type X collagen neo-epitope biomarker for knee osteoarthritis. *Osteoarthritis Cartilage* 2019;27(4):611-20. doi: 10.1016/j.joca.2019.01.001
53. Laurent TC, Laurent UB, Fraser JRE. Serum hyaluronan as a disease marker. *Ann Med* 1996;28(3):241-53.
54. Georges C, Vigneron H, Ayral X, et al. Serum biologic markers as predictors of disease progression in osteoarthritis of the knee. *Arthritis Rheum* 1997;40(3):590-1. doi: 10.1002/art.1780400333 [published Online First: 1997/03/01]
55. Elliott AL, Kraus VB, Luta G, et al. Serum hyaluronan levels and radiographic knee and hip osteoarthritis in African Americans and Caucasians in the Johnston County Osteoarthritis Project. *Arthritis Rheum* 2005;52(1):105-11. doi: 10.1002/art.20724 [published Online First: 2005/01/11]
56. Sharif M, George E, Shepstone L, et al. Serum hyaluronic acid level as a predictor of disease progression in osteoarthritis of the knee. *Arthritis Rheum* 1995;38(6):760-7. doi: 10.1002/art.1780380608 [published Online First: 1995/06/01]
57. Rizkalla G, Reiner A, Bogoch E, et al. Studies of the articular cartilage proteoglycan aggrecan in health and osteoarthritis. Evidence for molecular heterogeneity and extensive molecular changes in disease. *J Clin Invest* 1992;90(6):2268-77. doi: 10.1172/JCI116113 [published Online First: 1992/12/01]
58. Chu CR, Sheth S, Erhart-Hledik JC, et al. Mechanically stimulated biomarkers signal cartilage changes over 5 years consistent with disease progression in medial knee osteoarthritis patients. *J Orthop Res* 2018;36(3):891-97. doi: 10.1002/jor.23720 [published Online First: 2017/09/02]
59. Mazzuca SA, Poole AR, Brandt KD, et al. Associations between joint space narrowing and molecular markers of collagen and proteoglycan turnover in patients with knee osteoarthritis. *J Rheumatol* 2006;33(6):1147-51.
60. Lohmander SL, Neame PJ, Sandy JD. The structure of aggrecan fragments in human synovial fluid. evidence that aggrecanase mediates cartilage degradation in inflammatory joint disease, joint injury, and osteoarthritis. *Arthritis Rheum* 1993;36(9):1214-22. doi: https://doi.org/10.1002/art.1780360906
61. Larsson S, Lohmander LS, Struglics A. Synovial fluid level of aggrecan ARGS fragments is a more sensitive marker of joint disease than glycosaminoglycan or aggrecan levels: a cross-sectional study. *Arthritis Res Ther* 2009;11(3):R92. doi: 10.1186/ar2735
62. Larsson S, Lohmander LS, Struglics A. An ARGS-aggrecan assay for analysis in blood and synovial fluid. *Osteoarthritis Cartilage* 2014;22(2):242-9. doi: 10.1016/j.joca.2013.12.010 [published Online First: 2013/12/24]
63. Blumenfeld O, Williams FMK, Hart DJ, et al. Association between cartilage and bone biomarkers and incidence of radiographic knee osteoarthritis (RKOA) in UK females: a prospective study. *Osteoarthritis Cartilage* 2013;21(7):923-29. doi: 10.1016/j.joca.2013.04.009
64. Larsson S, Englund M, Struglics A, et al. Association between synovial fluid levels of aggrecan ARGS fragments and radiographic progression in knee osteoarthritis. *Arthritis Res Ther* 2010;12(6):R230. doi: 10.1186/ar3217

For peer review only

65. Saxne T, Heinegård D. Cartilage oligomeric matrix protein: a novel marker of cartilage turnover detectable in synovial fluid and blood. *Rheumatology* 1992;31(9):583-91. doi: 10.1093/rheumatology/31.9.583
66. Bi X. Correlation of serum cartilage oligomeric matrix protein with knee osteoarthritis diagnosis: a meta-analysis. *J Orthop Surg Res* 2018;13(1):262. doi: 10.1186/s13018-018-0959-y [published Online First: 2018/10/21]
67. Vilim V, Olejarova M, Machacek S, et al. Serum levels of cartilage oligomeric matrix protein (COMP) correlate with radiographic progression of knee osteoarthritis. *Osteoarthritis Cartilage* 2002;10(9):707-13. doi: 10.1053/joca.2002.0819 [published Online First: 2002/08/31]
68. Verma P, Dalal K. Serum cartilage oligomeric matrix protein (COMP) in knee osteoarthritis: A novel diagnostic and prognostic biomarker. *J Orthop Res* 2013;31(7):999-1006. doi: 10.1002/jor.22324
69. Hoch JM, Mattacola CG, Medina McKeon JM, et al. Serum cartilage oligomeric matrix protein (sCOMP) is elevated in patients with knee osteoarthritis: a systematic review and meta-analysis. *Osteoarthritis Cartilage* 2011;19(12):1396-404. doi: 10.1016/j.joca.2011.09.005 [published Online First: 2011/10/18]
70. Clark AG, Jordan JM, Vilim V, et al. Serum cartilage oligomeric matrix protein reflects osteoarthritis presence and severity: The Johnston county osteoarthritis project. *Arthritis Rheum* 1999;42(11):2356-64. doi: 10.1002/1529-0131(199911)42:11<2356::aid-anr14>3.0.co;2-r
71. Delmas PD. Biochemical markers of bone turnover. *J Bone Miner Res* 1993;8 Suppl 2:S549-55. doi: 10.1002/jbmr.5650081323
72. Bruyere O, Collette JH, Ethgen O, et al. Biochemical markers of bone and cartilage remodeling in prediction of longterm progression of knee osteoarthritis. *J Rheumatol* 2003;30(5):1043-50.
73. Gomez B, Jr., Ardakani S, Ju J, et al. Monoclonal antibody assay for measuring bone-specific alkaline phosphatase activity in serum. *Clinical Chemistry* 1995;41(11):1560-66. doi: 10.1093/clinchem/41.11.1560
74. Park H-M, Lee J-H, Lee Y-J. Positive Association of Serum Alkaline Phosphatase Level with Severe Knee Osteoarthritis: A Nationwide Population-Based Study. *Diagnostics* 2020;10(12):1016. doi: 10.3390/diagnostics10121016
75. Orum O, Hansen M, Jensen CH, et al. Procollagen type I N-terminal propeptide (PINP) as an indicator of type I collagen metabolism: ELISA development, reference interval, and hypovitaminosis D induced hyperparathyroidism. *Bone* 1996;19(2):157-63. doi: 10.1016/8756-3282(96)00165-2
76. Kumm J, Tamm A, Lintrop M, et al. Diagnostic and prognostic value of bone biomarkers in progressive knee osteoarthritis: a 6-year follow-up study in middle-aged subjects. *Osteoarthritis and cartilage* 2013;21(6):815-22. doi: 10.1016/j.joca.2013.03.008
77. Delmas PD, Schlemmer A, Gineyts E, et al. Urinary excretion of pyridinoline crosslinks correlates with bone turnover measured on iliac crest biopsy in patients with vertebral osteoporosis. *J Bone Miner Res* 1991;6(6):639-44. doi: 10.1002/jbmr.5650060615
78. Schmidt-Rohlfing B, Thomsen M, Niedhart C, et al. Correlation of bone and cartilage markers in the synovial fluid with the degree of osteoarthritis. *Rheumatology International* 2002;21(5):193-99. doi: 10.1007/s00296-001-0170-y
79. Rosen HN, Moses AC, Garber J, et al. Serum CTX: A New Marker of Bone Resorption That Shows Treatment Effect More Often Than Other Markers Because of Low Coefficient of Variability and Large Changes with Bisphosphonate Therapy. *Calcified Tissue International* 2000;66(2):100-03. doi: 10.1007/pl00005830
80. Rosen HN, Dresner-Pollak R, Moses AC, et al. Specificity of urinary excretion of cross-linked N-telopeptides of type I collagen as a marker of bone turnover. *Calcified Tissue International* 1994;54(1):26-29. doi: 10.1007/bf00316285

For peer review only

81. Bettica P, Cline G, Hart DJ, et al. Evidence for increased bone resorption in patients with progressive knee osteoarthritis: Longitudinal results from the Chingford study. *Arthritis & Rheumatism* 2002;46(12):3178-84. doi: 10.1002/art.10630

For peer review only

Screening Tool: Title/Abstract

Codebook

Data Dictionary Codebook

2022-12-22 21:32:53

Collapse all instruments

	#	Variable / Field Name	**Field Label**<br>***Field Note***	Field Attributes (Field Type, Validation, Choices, Calculations, etc.)
Instrument: **Screening Tool** (screening_tool)				Collapse
	1	record_id	Record ID	text
	2	instructions	Yes - There is explicit evidence in the title or abstract of the research including this.	descriptive
	3	maybe	Maybe - It is unclear whether there is evidence of this in the title/abstract. It may or may not be relevant but I feel this needs further investigation.	descriptive
	4	no	No - There is explicit evidence in the title or abstract of the research not including this.	descriptive
	5	reviewer_screening	Section Header: *Reviewer Details*<br>Reviewer	radio, Required<br>1 Karl Morgan<br>2 James Cowburn<br>3 Mathew Farrow<br>4 Josh Carter
	6	reviewer_date_screening	Date<br>*Just press 'Today'*	text (date_dmy), Required
	7	record_number_screening	Record number	text (number), Required
	8	review_no_meta	Section Header: *1. Study Information*<br>Is this a review, meta-analysis, book, protocol or commentary?	checkbox<br>1 review_no_meta___1 No<br>2 review_no_meta___2 Maybe<br>3 review_no_meta___3 Yes
	9	animal<br>Show the field ONLY if:<br>[review_no_meta(3)] <> 1	Is the study an animal model?	checkbox<br>1 animal___1 No<br>2 animal___2 Maybe<br>3 animal___3 Yes
	10	in_silico<br>Show the field ONLY if:<br>[review_no_meta(3)] <> 1 and [animal(3)] <> 1	Is the study purely in silico?	checkbox<br>1 in_silico___1 No<br>2 in_silico___2 Maybe<br>3 in_silico___3 Yes
	11	in_vitro<br>Show the field ONLY if:<br>[review_no_meta(3)] <> 1 and [animal(3)] <> 1 and [in_silico (3)] <> 1	Is the study purely in vitro (i.e. no human participants)?	checkbox<br>1 in_vitro___1 No<br>2 in_vitro___2 Maybe<br>3 in_vitro___3 Yes
	12	cadavers<br>Show the field ONLY if:<br>[review_no_meta(3)] <> 1 and [animal(3)] <> 1 and [in_silico (3)] <> 1 and [in_vitro(3)] <> 1	Is the study only working with cadavers?	checkbox<br>1 cadavers___1 No<br>2 cadavers___2 Maybe<br>3 cadavers___3 Yes
	13	knee_oa<br>Show the field ONLY if:<br>[review_no_meta(3)] <> 1 and [animal(3)] <> 1 and [in_silico (3)] <> 1 and [in_vitro(3)] <> 1 and [cadavers(3)] <> 1	Do the participants already have knee osteoarthritis (e.g., a cross-sectional study looking at the effects of exercise in people who already have knee OA disease) or another type of arthritis/disease?	checkbox<br>1 knee_oa___1 No<br>2 knee_oa___2 Maybe<br>3 knee_oa___3 Yes

	14	knee_injury<br>Show the field ONLY if: [review_no_meta(3)] <> 1 and [animal(3)] <> 1 and [in_silico (3)] <> 1 and [in_vitro(3)] <> 1 and [cadavers(3)] <> 1 and [k nee_oa(3)] <> 1	Section Header: *2. Population*<br>Could this study involve those at risk of knee post-traumatic osteoarthritis (PTOA) such as after a knee injury? Cruciate ligament injuryCruciate ligament reconstructionMeniscus injuryMeniscectomy<br><br>This does NOT include:<br>Total knee replacementKnee arthroplastyosteotomy<br>*Please select one option*	checkbox, Required<br>1 knee_injury___1 No<br>2 knee_injury___2 Maybe<br>3 knee_injury___3 Yes
	15	age<br>Show the field ONLY if: [review_no_meta(3)] <> 1 and [animal(3)] <> 1 and [in_silico (3)] <> 1 and [in_vitro(3)] <> 1 and [cadavers(3)] <> 1 and [k nee_oa(3)] <> 1 and [knee_inj ury(1)] <> 1	Could the study participants be aged 18-40?<br>At least 50% of the population must be within 18-40 years old.<br><br>If the study is longitudinal, use the baseline age.<br>*Please select one option.*	checkbox, Required<br>1 age___1 No<br>2 age___2 Maybe<br>3 age___3 Yes
	16	independent_variable<br>Show the field ONLY if: [review_no_meta(3)] <> 1 and [animal(3)] <> 1 and [in_silico (3)] <> 1 and [in_vitro(3)] <> 1 and [cadavers(3)] <> 1 and [k nee_oa(3)] <> 1 and [knee_inj ury(1)] <> 1 and [age(1)] <> 1	Section Header: *3. Independent Variable*<br>Could the study have an independent variable which measures:<br>Physical activity (including Tegner)ExerciseSystemic inflammation (e.g., IL-6 concentrations)Knee joint load (moments or MSK modelling)Adipose tissueMuscle strengthMuscle sizeIntra-muscular adipose tissueBone mineral content or density<br>Or may the study have an intervention involving:<br><br>Physical activity or exerciseManipulation of joint loadWeight lossDisuse or bedrest<br>*Please select one option*	checkbox, Required<br>1 independent_variable___1 No<br>2 independent_variable___2 Maybe<br>3 independent_variable___3 Yes
	17	diagnosis_header<br>Show the field ONLY if: [review_no_meta(3)] <> 1 and [animal(3)] <> 1 and [in_silico (3)] <> 1 and [in_vitro(3)] <> 1 and [cadavers(3)] <> 1 and [k nee_oa(3)] <> 1 and [knee_inj ury(1)] <> 1 and [age(1)] <> 1 and [independent_variable (1)] <> 1	Section Header: *4. Outcome Variable*<br>Knee Osteoarthritis (OA) Diagnosis	descriptive
	18	disease_diagnoses<br>Show the field ONLY if: [review_no_meta(3)] <> 1 and [animal(3)] <> 1 and [in_silico (3)] <> 1 and [in_vitro(3)] <> 1 and [cadavers(3)] <> 1 and [k nee_oa(3)] <> 1 and [knee_inj ury(1)] <> 1 and [age(1)] <> 1 and [independent_variable (1)] <> 1	Could the study investigate knee OA diagnosis including the following scales:<br>Kellgren and Lawrence (K/L)Osteoarthritis Research Society International (OARSI) AtlasInternational Knee Documentation Committee (IKDC) SystemAhlbäck SystemFairbank ClassificationBrandt Grading ScaleJäger-Wirth ClassificationMerchant SystemMRI Osteoarthritis Knee Score (MOAKS)Whole Organ Magnetic Resonance Imaging Score (WORMS)Boston Leeds OA Knee Score (BLOKS)<br><br>P.s if there is another system mentioned that your are unsure of then please send me a message.<br>*Please select one option*	checkbox, Required<br>1 disease_diagnoses___1 No<br>2 disease_diagnoses___2 Maybe<br>3 disease_diagnoses___3 Yes
	19	imaging_indicator_header<br>Show the field ONLY if: [review_no_meta(3)] <> 1 and [animal(3)] <> 1 and [in_silico (3)] <> 1 and [in_vitro(3)] <> 1 and [cadavers(3)] <> 1 and [k nee_oa(3)] <> 1 and [knee_inj ury(1)] <> 1 and [age(1)] <> 1 and [independent_variable (1)] <> 1	Medical Imaging Indicator	descriptive
	20	medical_imaging_indicators<br>Show the field ONLY if: [review_no_meta(3)] <> 1 and [animal(3)] <> 1 and [in_silico (3)] <> 1 and [in_vitro(3)] <> 1 and [cadavers(3)] <> 1 and [k nee_oa(3)] <> 1 and [knee_inj ury(1)] <> 1 and [age(1)] <> 1 and [independent_variable (1)] <> 1	Could the study investigate medical imaging indicators of tissue health including the following techniques:<br>MRI quantified cartilage thickness and volumeT1rho mappingT2 mappingDelayed contrast-enhanced MRI of cartilage (dGEMRIC)Ultrasound quantified cartilage thickness<br>*Please select one option*	checkbox, Required<br>1 medical_imaging_indicators___1 No<br>2 medical_imaging_indicators___2 Maybe<br>3 medical_imaging_indicators___3 Yes

For peer review only - http://bmjopen.bmj.com/site/about/guidelines.xhtml

	21	biomarkers_header<br><br>Show the field ONLY if:<br>[review_no_meta(3)] <> 1 and [animal(3)] <> 1 and [in_silico(3)] <> 1 and [in_vitro(3)] <> 1 and [cadavers(3)] <> 1 and [knee_oa(3)] <> 1 and [knee_injury(1)] <> 1 and [age(1)] <> 1 and [independent_variable(1)] <> 1	Biomarkers	descriptive
	22	biomarkers<br><br>Show the field ONLY if:<br>[review_no_meta(3)] <> 1 and [animal(3)] <> 1 and [in_silico(3)] <> 1 and [in_vitro(3)] <> 1 and [cadavers(3)] <> 1 and [knee_oa(3)] <> 1 and [knee_injury(1)] <> 1 and [age(1)] <> 1 and [independent_variable(1)] <> 1	Could the study investigate biomarkers including the following:<br>Cartilage synthesisC-propeptide of type II collagen (CP-II)Chondroitin sulphate 846 epitope (CS846)N-Propeptide of Collagen IIA (PIIANP)N-Propeptide of Collagen IIB (PIIBNP)<br>Cartilage degradationMatrix metalloproteases (e.g., MMP-3, MMP-9, MMP-13)A disintegrin and metalloproteinase with thrombospondin motifs (e.g., ADAMTS-4, ADAMTS-5)Collagen type II cleavage product (C2C)Col2-3/4 C-terminal cleavage product of types I and II collagen (C1,2C)Neoepitope of collagen X (C10C or Col10Neo)Hyaluronic acid (HA) Aggrecan or aggrecan fragments (ARGS)Keratin sulphate (KS)Cartilage oligomeric matrix protein (COMP)<br>Bone formationOsteocalcin (OC)Bone alkaline phosphatase (BAP)Procollagen type I N-terminal propeptide (PINP)Procollagen type I C-terminal propeptide (PICP)<br>Bone resorptionPyridinoline (PYD)Deoxypyridinoline (DPD)Tartrate-resistant acid phosphatase 5b (TRAP-5)N-telopeptide if type I collagen (NTX-I)C-telopeptide of type I collagen (CTX-I)Insulin-like growth factor 1 (IGF-1)Transforming growth factor beta (TGFB)<br>*Please select one option*	checkbox, Required<br>1 | biomarkers___1 | No<br>2 | biomarkers___2 | Maybe<br>3 | biomarkers___3 | Yes
	23	all_yes_dec<br><br>Show the field ONLY if:<br>[knee_injury(3)]= 1 and [age(3)] = 1 and [independent_variable(3)] = 1 and [review_no_meta(3)] <> 1 and [animal(3)] <> 1 and [in_silico(3)] <> 1 and [cadavers(3)] <> 1 or [disease_diagnoses(3)] = 1 or [medical_imaging_indicators(3)] = 1 or [biomarkers(3)] = 1	Section Header: *Decision*<br><br>Select 'Yes' in Covidence	descriptive
	24	all_maybe_dec<br><br>Show the field ONLY if:<br>[knee_injury(2)]= 1 and [age(2)] = 1 and [independent_variable(2)] = 1 and [review_no_meta(1)] <> 1 and [animal(1)] <> 1 and [in_silico(1)] <> 1 and [cadavers(1)] <> 1	Select 'Maybe' in Covidence	descriptive
	25	knee_inj_maybe<br><br>Show the field ONLY if:<br>[knee_injury(2)]= 1 and [age(1)] = 1 and [independent_variable(1)] = 1 and [disease(1)] = 1	Select 'Maybe' in Covidence	descriptive
	26	age_maybe<br><br>Show the field ONLY if:<br>[knee_injury(1)]= 1 and [age(2)] = 1 and [independent_variable(1)] = 1 and [disease(1)] = 1 and [review_no_meta(1)] <> 1 and [animal(1)] <> 1 and [in_silico(1)] <> 1 and [cadavers(1)] <> 1	Select 'Maybe' in Covidence	descriptive

	27	iv_maybe<br>Show the field ONLY if: [knee_injury(1)]= 1 and [age(1)] = 1 and [independent_variable(2)] = 1 and [disease(1)] = 1 and [review_no_meta(1)] <> 1 and [animal(1)] <> 1 and [in_silico(1)] <> 1 and [cadavers(1)] <> 1	Select 'Maybe' in Covidence	descriptive
	28	ov_maybe<br>Show the field ONLY if: [knee_injury(1)]= 1 and [age(1)] = 1 and [independent_variable(1)] = 1 and [disease(2)] = 1 and [review_no_meta(1)] <> 1 and [animal(1)] <> 1 and [in_silico(1)] <> 1 and [cadavers(1)] <> 1	Select 'Maybe' in Covidence	descriptive
	29	age_iv_ov_maybe<br>Show the field ONLY if: [knee_injury(1)]= 1 and [age(2)] = 1 and [independent_variable(2)] = 1 and [disease(2)] = 1 and [review_no_meta(1)] <> 1 and [animal(1)] <> 1 and [in_silico(1)] <> 1 and [cadavers(1)] <> 1	Select 'Maybe' in Covidence	descriptive
	30	knee_inj_iv_ov_maybe<br>Show the field ONLY if: [knee_injury(2)]= 1 and [age(1)] = 1 and [independent_variable(2)] = 1 and [disease(2)] = 1 and [review_no_meta(1)] <> 1 and [animal(1)] <> 1 and [in_silico(1)] <> 1 and [cadavers(1)] <> 1	Select 'Maybe' in Covidence	descriptive
	31	knee_inj_age_ov_maybe<br>Show the field ONLY if: [knee_injury(2)]= 1 and [age(2)] = 1 and [independent_variable(1)] = 1 and [disease(2)] = 1 and [review_no_meta(1)] <> 1 and [animal(1)] <> 1 and [in_silico(1)] <> 1 and [cadavers(1)] <> 1	Select 'Maybe' in Covidence	descriptive
	32	knee_inj_age_iv_maybe<br>Show the field ONLY if: [knee_injury(2)]= 1 and [age(2)] = 1 and [independent_variable(2)] = 1 and [review_no_meta(1)] <> 1 and [animal(1)] <> 1 and [in_silico(1)] <> 1 and [cadavers(1)] <> 1	Select 'Maybe' in Covidence	descriptive
	33	knee_inj_age_iv_maybe_2<br>Show the field ONLY if: [disease_diagnoses(2)] = 1 AND [medical_imaging_indicators(2)] = 1 AND [biomarkers(2)] = 1	Select 'Maybe' in Covidence	descriptive
	34	select_no<br>Show the field ONLY if: [review_no_meta(3)] = 1 or [animal(3)] = 1 or [in_silico(3)] = 1 or [in_vitro(3)] = 1 or [cadavers(3)] = 1 or [knee_oa(3)] =1 or [knee_injury(1)] = 1 or [age(1)] = 1 or [independent_variable(1)] = 1	Select 'No' in Covidence	descriptive

	35	select_no_2<br><br>Show the field ONLY if: [disease_diagnoses(1)] = 1 AND [medical_imaging_indicators(1)] = 1 AND [biomarkers(1)] = 1	Select 'No' in Covidence	descriptive
	36	screening_tool_complete	Section Header: *Form Status*<br><br>Complete?	dropdown<br>0 Incomplete<br>1 Unverified<br>2 Complete

For peer review only

Screening Tool: Full Text

Codebook

Data Dictionary Codebook

2022-12-22 21:35:11

Collapse all instruments

	#	Variable / Field Name	Field Label<br>*Field Note*	Field Attributes (Field Type, Validation, Choices, Calculations, etc.)
Instrument: **Screening Tool** (screening_tool) Collapse				
	1	record_id	Record ID	text
	2	instructions	Yes - There is explicit evidence in the title or abstract of the research including this.	descriptive
	3	maybe	Maybe - It is unclear whether there is evidence of this in the title/abstract. It may or may not be relevant but I feel this needs further investigation.	descriptive
	4	no	No - There is explicit evidence in the title or abstract of the research not including this.	descriptive
	5	reviewer_screening	Section Header: *Reviewer Details*<br>Reviewer	radio, Required<br>1 Karl Morgan<br>2 James Cowburn<br>3 Mathew Farrow<br>4 Josh Carter
	6	reviewer_date_screening	Date<br>*Just press 'Today'*	text (date_dmy), Required
	7	record_number_screening	Record number	text (number), Required
	8	review_no_meta	Section Header: *1. Study Information*<br>Is this a review, meta-analysis, book, protocol or commentary?	checkbox<br>1 review_no_meta___1 No<br>2 review_no_meta___2 Maybe<br>3 review_no_meta___3 Yes
	9	animal<br>Show the field ONLY if:<br>[review_no_meta(3)] <> 1	Is the study an animal model?	checkbox<br>1 animal___1 No<br>2 animal___2 Maybe<br>3 animal___3 Yes
	10	in_silico<br>Show the field ONLY if:<br>[review_no_meta(3)] <> 1 and [animal(3)] <> 1	Is the study purely in silico?	checkbox<br>1 in_silico___1 No<br>2 in_silico___2 Maybe<br>3 in_silico___3 Yes
	11	in_vitro<br>Show the field ONLY if:<br>[review_no_meta(3)] <> 1 and [animal(3)] <> 1 and [in_silico (3)] <> 1	Is the study purely in vitro (i.e. no human participants)?	checkbox<br>1 in_vitro___1 No<br>2 in_vitro___2 Maybe<br>3 in_vitro___3 Yes
	12	cadavers<br>Show the field ONLY if:<br>[review_no_meta(3)] <> 1 and [animal(3)] <> 1 and [in_silico (3)] <> 1 and [in_vitro(3)] <> 1	Is the study only working with cadavers?	checkbox<br>1 cadavers___1 No<br>2 cadavers___2 Maybe<br>3 cadavers___3 Yes
	13	knee_oa<br>Show the field ONLY if:<br>[review_no_meta(3)] <> 1 and [animal(3)] <> 1 and [in_silico (3)] <> 1 and [in_vitro(3)] <> 1 and [cadavers(3)] <> 1	Do the participants already have knee osteoarthritis (e.g., a cross-sectional study looking at the effects of exercise in people who already have knee OA disease) or another type of arthritis/disease?	checkbox<br>1 knee_oa___1 No<br>2 knee_oa___2 Maybe<br>3 knee_oa___3 Yes

	14	knee_injury<br><br>Show the field ONLY if:<br>[review_no_meta(3)] <> 1 and [animal(3)] <> 1 and [in_silico(3)] <> 1 and [in_vitro(3)] <> 1 and [cadavers(3)] <> 1 and [knee_oa(3)] <> 1	Section Header: *2. Population*<br><br>Could this study involve those at risk of knee post-traumatic osteoarthritis (PTOA) such as after a knee injury? Cruciate ligament injuryCruciate ligament reconstructionMeniscus injuryMeniscectomy<br><br>This does NOT include:<br>Total knee replacementKnee arthroplastyosteotomy<br>*Please select one option*	checkbox, Required<br>1 knee_injury___1 No<br>2 knee_injury___2 Maybe<br>3 knee_injury___3 Yes
	15	age<br><br>Show the field ONLY if:<br>[review_no_meta(3)] <> 1 and [animal(3)] <> 1 and [in_silico(3)] <> 1 and [in_vitro(3)] <> 1 and [cadavers(3)] <> 1 and [knee_oa(3)] <> 1 and [knee_injury(1)] <> 1	Could the study participants be aged 18-40?<br>At least 50% of the population must be within 18-40 years old.<br><br>If the study is longitudinal, use the baseline age.<br>*Please select one option.*	checkbox, Required<br>1 age___1 No<br>2 age___2 Maybe<br>3 age___3 Yes
	16	independent_variable<br><br>Show the field ONLY if:<br>[review_no_meta(3)] <> 1 and [animal(3)] <> 1 and [in_silico(3)] <> 1 and [in_vitro(3)] <> 1 and [cadavers(3)] <> 1 and [knee_oa(3)] <> 1 and [knee_injury(1)] <> 1 and [age(1)] <> 1	Section Header: *3. Independent Variable*<br><br>Could the study have an independent variable which measures:<br>Physical activity (including Tegner)ExerciseSystemic inflammation (e.g., IL-6 concentrations)Knee joint load (moments or MSK modelling)Adipose tissueMuscle strengthMuscle sizeIntra-muscular adipose tissueBone mineral content or density<br>Or may the study have an intervention involving:<br><br>Physical activity or exerciseManipulation of joint loadWeight lossDisuse or bedrest<br>*Please select one option*	checkbox, Required<br>1 independent_variable___1 No<br>2 independent_variable___2 Maybe<br>3 independent_variable___3 Yes
	17	diagnosis_header<br><br>Show the field ONLY if:<br>[review_no_meta(3)] <> 1 and [animal(3)] <> 1 and [in_silico(3)] <> 1 and [in_vitro(3)] <> 1 and [cadavers(3)] <> 1 and [knee_oa(3)] <> 1 and [knee_injury(1)] <> 1 and [age(1)] <> 1 and [independent_variable(1)] <> 1	Section Header: *4. Outcome Variable*<br><br>Knee Osteoarthritis (OA) Diagnosis	descriptive
	18	disease_diagnoses<br><br>Show the field ONLY if:<br>[review_no_meta(3)] <> 1 and [animal(3)] <> 1 and [in_silico(3)] <> 1 and [in_vitro(3)] <> 1 and [cadavers(3)] <> 1 and [knee_oa(3)] <> 1 and [knee_injury(1)] <> 1 and [age(1)] <> 1 and [independent_variable(1)] <> 1	Could the study investigate knee OA diagnosis including the following scales:<br>Kellgren and Lawrence (K/L)Osteoarthritis Research Society International (OARSI) AtlasInternational Knee Documentation Committee (IKDC) SystemAhlbäck SystemFairbank ClassificationBrandt Grading ScaleJäger-Wirth ClassificationMerchant SystemMRI Osteoarthritis Knee Score (MOAKS)Whole Organ Magnetic Resonance Imaging Score (WORMS)Boston Leeds OA Knee Score (BLOKS)<br><br>P.s if there is another system mentioned that your are unsure of then please send me a message.<br>*Please select one option*	checkbox, Required<br>1 disease_diagnoses___1 No<br>2 disease_diagnoses___2 Maybe<br>3 disease_diagnoses___3 Yes
	19	imaging_indicator_header<br><br>Show the field ONLY if:<br>[review_no_meta(3)] <> 1 and [animal(3)] <> 1 and [in_silico(3)] <> 1 and [in_vitro(3)] <> 1 and [cadavers(3)] <> 1 and [knee_oa(3)] <> 1 and [knee_injury(1)] <> 1 and [age(1)] <> 1 and [independent_variable(1)] <> 1	Medical Imaging Indicator	descriptive
	20	medical_imaging_indicators<br><br>Show the field ONLY if:<br>[review_no_meta(3)] <> 1 and [animal(3)] <> 1 and [in_silico(3)] <> 1 and [in_vitro(3)] <> 1 and [cadavers(3)] <> 1 and [knee_oa(3)] <> 1 and [knee_injury(1)] <> 1 and [age(1)] <> 1 and [independent_variable(1)] <> 1	Could the study investigate medical imaging indicators of tissue health including the following techniques:<br>MRI quantified cartilage thickness and volumeT1rho mappingT2 mappingDelayed contrast-enhanced MRI of cartilage (dGEMRIC)Ultrasound quantified cartilage thickness<br>*Please select one option*	checkbox, Required<br>1 medical_imaging_indicators___1 No<br>2 medical_imaging_indicators___2 Maybe<br>3 medical_imaging_indicators___3 Yes

For peer review only - http://bmjopen.bmj.com/site/about/guidelines.xhtml

	21	biomarkers_header<br><br>Show the field ONLY if:<br>[review_no_meta(3)] <> 1 and [animal(3)] <> 1 and [in_silico(3)] <> 1 and [in_vitro(3)] <> 1 and [cadavers(3)] <> 1 and [knee_oa(3)] <> 1 and [knee_injury(1)] <> 1 and [age(1)] <> 1 and [independent_variable(1)] <> 1	Biomarkers	descriptive
	22	biomarkers<br><br>Show the field ONLY if:<br>[review_no_meta(3)] <> 1 and [animal(3)] <> 1 and [in_silico(3)] <> 1 and [in_vitro(3)] <> 1 and [cadavers(3)] <> 1 and [knee_oa(3)] <> 1 and [knee_injury(1)] <> 1 and [age(1)] <> 1 and [independent_variable(1)] <> 1	Could the study investigate biomarkers including the following:<br>Cartilage synthesisC-propeptide of type II collagen (CP-II)Chondroitin sulphate 846 epitope (CS846)N-Propeptide of Collagen IIA (PIIANP)N-Propeptide of Collagen IIB (PIIBNP)<br>Cartilage degradationMatrix metalloproteases (e.g., MMP-3, MMP-9, MMP-13)A disintegrin and metalloproteinase with thrombospondin motifs (e.g., ADAMTS-4, ADAMTS-5)Collagen type II cleavage product (C2C)Col2-3/4 C-terminal cleavage product of types I and II collagen (C1,2C)Neoepitope of collagen X (C10C or Col10Neo)Hyaluronic acid (HA) Aggrecan or aggrecan fragments (ARGS)Keratin sulphate (KS)Cartilage oligomeric matrix protein (COMP)<br>Bone formationOsteocalcin (OC)Bone alkaline phosphatase (BAP)Procollagen type I N-terminal propeptide (PINP)Procollagen type I C-terminal propeptide (PICP)<br>Bone resorptionPyridinoline (PYD)Deoxypyridinoline (DPD)Tartrate-resistant acid phosphatase 5b (TRAP-5)N-telopeptide if type I collagen (NTX-I)C-telopeptide of type I collagen (CTX-I)Insulin-like growth factor 1 (IGF-1)Transforming growth factor beta (TGFB)<br>*Please select one option*	checkbox, Required<br>1 | biomarkers___1 | No<br>2 | biomarkers___2 | Maybe<br>3 | biomarkers___3 | Yes
	23	all_yes_dec<br><br>Show the field ONLY if:<br>[knee_injury(3)]= 1 and [age(3)] = 1 and [independent_variable(3)] = 1 and [review_no_meta(3)] <> 1 and [animal(3)] <> 1 and [in_silico(3)] <> 1 and [cadavers(3)] <> 1 or [disease_diagnoses(3)] = 1 or [medical_imaging_indicators(3)] = 1 or [biomarkers(3)] = 1	Section Header: *Decision*<br>Select 'Yes' in Covidence	descriptive
	24	all_maybe_dec<br><br>Show the field ONLY if:<br>[knee_injury(2)]= 1 and [age(2)] = 1 and [independent_variable(2)] = 1 and [review_no_meta(1)] <> 1 and [animal(1)] <> 1 and [in_silico(1)] <> 1 and [cadavers(1)] <> 1	Select 'Maybe' in Covidence	descriptive
	25	knee_inj_maybe<br><br>Show the field ONLY if:<br>[knee_injury(2)]= 1 and [age(1)] = 1 and [independent_variable(1)] = 1 and [disease(1)] = 1	Select 'Maybe' in Covidence	descriptive
	26	age_maybe<br><br>Show the field ONLY if:<br>[knee_injury(1)]= 1 and [age(2)] = 1 and [independent_variable(1)] = 1 and [disease(1)] = 1 and [review_no_meta(1)] <> 1 and [animal(1)] <> 1 and [in_silico(1)] <> 1 and [cadavers(1)] <> 1	Select 'Maybe' in Covidence	descriptive

	27	iv_maybe<br>Show the field ONLY if: [knee_injury(1)]= 1 and [age(1)] = 1 and [independent_variable(2)] = 1 and [disease(1)] = 1 and [review_no_meta(1)] <> 1 and [animal(1)] <> 1 and [in_silico(1)] <> 1 and [cadavers(1)] <> 1	Select 'Maybe' in Covidence	descriptive
	28	ov_maybe<br>Show the field ONLY if: [knee_injury(1)]= 1 and [age(1)] = 1 and [independent_variable(1)] = 1 and [disease(2)] = 1 and [review_no_meta(1)] <> 1 and [animal(1)] <> 1 and [in_silico(1)] <> 1 and [cadavers(1)] <> 1	Select 'Maybe' in Covidence	descriptive
	29	age_iv_ov_maybe<br>Show the field ONLY if: [knee_injury(1)]= 1 and [age(2)] = 1 and [independent_variable(2)] = 1 and [disease(2)] = 1 and [review_no_meta(1)] <> 1 and [animal(1)] <> 1 and [in_silico(1)] <> 1 and [cadavers(1)] <> 1	Select 'Maybe' in Covidence	descriptive
	30	knee_inj_iv_ov_maybe<br>Show the field ONLY if: [knee_injury(2)]= 1 and [age(1)] = 1 and [independent_variable(2)] = 1 and [disease(2)] = 1 and [review_no_meta(1)] <> 1 and [animal(1)] <> 1 and [in_silico(1)] <> 1 and [cadavers(1)] <> 1	Select 'Maybe' in Covidence	descriptive
	31	knee_inj_age_ov_maybe<br>Show the field ONLY if: [knee_injury(2)]= 1 and [age(2)] = 1 and [independent_variable(1)] = 1 and [disease(2)] = 1 and [review_no_meta(1)] <> 1 and [animal(1)] <> 1 and [in_silico(1)] <> 1 and [cadavers(1)] <> 1	Select 'Maybe' in Covidence	descriptive
	32	knee_inj_age_iv_maybe<br>Show the field ONLY if: [knee_injury(2)]= 1 and [age(2)] = 1 and [independent_variable(2)] = 1 and [review_no_meta(1)] <> 1 and [animal(1)] <> 1 and [in_silico(1)] <> 1 and [cadavers(1)] <> 1	Select 'Maybe' in Covidence	descriptive
	33	knee_inj_age_iv_maybe_2<br>Show the field ONLY if: [disease_diagnoses(2)] = 1 AND [medical_imaging_indicators(2)] = 1 AND [biomarkers(2)] = 1	Select 'Maybe' in Covidence	descriptive
	34	select_no<br>Show the field ONLY if: [review_no_meta(3)] = 1 or [animal(3)] = 1 or [in_silico(3)] = 1 or [in_vitro(3)] = 1 or [cadavers(3)] = 1 or [knee_oa(3)] =1 or [knee_injury(1)] = 1 or [age(1)] = 1 or [independent_variable(1)] = 1	Select 'No' in Covidence	descriptive

	35	select_no_2<br><br>Show the field ONLY if:<br>[disease_diagnoses(1)] = 1 AND [medical_imaging_indicators(1)] = 1 AND [biomarkers(1)] = 1	Select 'No' in Covidence	descriptive
	36	screening_tool_complete	Section Header: *Form Status*<br><br>Complete?	dropdown<br>0 Incomplete<br>1 Unverified<br>2 Complete

For peer review only

Supplementary Material 5. Protocol for Contacting Authors

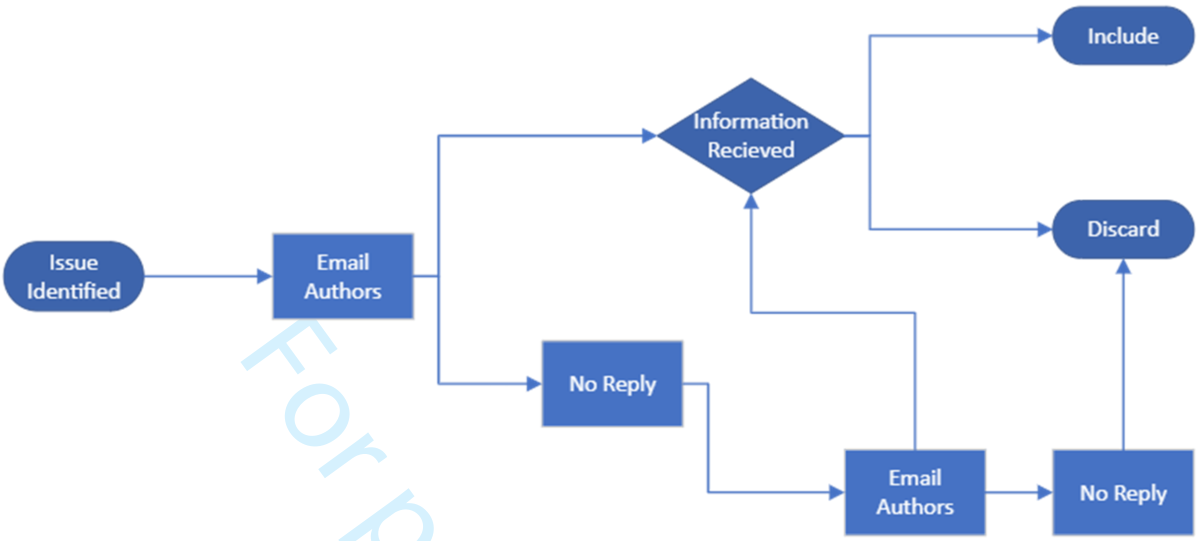

For peer review only

Supplementary Material 6. Johanna Brigg's Institute (JBI) Appraisal Criteria

REDCap Flow Diagram for Critical Appraisal

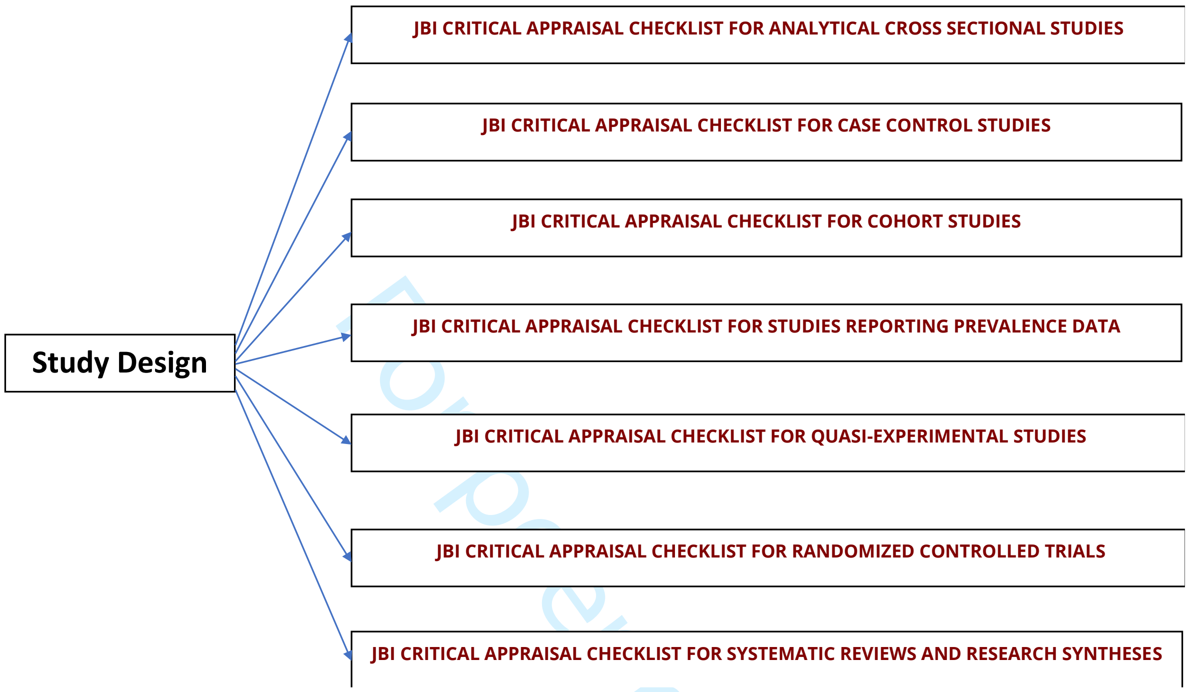

For peer review only

JBI Quality Appraisal

Codebook

Data Dictionary Codebook

2022-08-02 19:46:29

Collapse all instruments

	#	Variable / Field Name	Field Label<br>*Field Note*	Field Attributes (Field Type, Validation, Choices, Calculations, etc.)
Instrument: **Study Design** (study_design) Collapse				
	1	record_id	Record ID	text
	2	study_design	Please select the study design	radio<br>1 Analytical Cross-Sectional Study<br>2 Case Control Study<br>3 Cohort Study<br>4 Prevalence Study<br>5 Quasi-Experimental Study<br>6 Randomised Control Trial<br>7 Systematic Review and Research Synthesis<br>8 Unclear
	3	study_design_complete	Section Header: *Form Status*<br>Complete?	dropdown<br>0 Incomplete<br>1 Unverified<br>2 Complete
Instrument: **Study Design Unclear** (study_design_unclear) Collapse				
	4	reviewer_name_unclear	Section Header: *Reviewer Details*<br>Reviewer	dropdown<br>1 Karl Morgan<br>2 James Cowburn<br>3 Matthew Farrow
	5	reviewer_date_unclear	Date reviewed<br>*Just press 'Today'*	text (date_dmy)
	6	authors_unclear	Section Header: *Study Information*<br>Authors	text
	7	year_unclear	Publication year	text
	8	record_number_unclear	Record number	text
	9	study_design_unclear_complete	Section Header: *Form Status*<br>Complete?	dropdown<br>0 Incomplete<br>1 Unverified<br>2 Complete
Instrument: **JBI Critical Appraisal Checklist for Analytical Cross-Sectional Studies** (jbi_critical_appraisal_checklist_for_analytical_cr) Collapse				
	10	reviewer_name_cross	Section Header: *Reviewer Details*<br>Reviewer	dropdown<br>1 Karl Morgan<br>2 James Cowburn<br>3 Matthew Farrow
	11	reviewer_date_cross	Date reviewed<br>*Just press 'Today'*	text (date_dmy)
	12	authors_cross	Section Header: *Study Information*<br>Authors	text
	13	year_cross	Publication year	text
	14	record_number_cross	Record number	text

	15	cross_section_01	Section Header: *JBI Critical Appraisal Checklist for Analytical Cross-Sectional Studies*<br>1. Were the criteria for inclusion in the sample clearly defined?	radio (Matrix)<br>1 Yes<br>2 No<br>3 Unclear<br>4 Not Applicable
	16	cross_section_02	2. Were the study subjects and the setting described in detail?	radio (Matrix)<br>1 Yes<br>2 No<br>3 Unclear<br>4 Not Applicable
	17	cross_section_03	3. Was the exposure measured in a valid and reliable way?	radio (Matrix)<br>1 Yes<br>2 No<br>3 Unclear<br>4 Not Applicable
	18	cross_section_04	4. Were objective, standard criteria used for measurement of the condition?	radio (Matrix)<br>1 Yes<br>2 No<br>3 Unclear<br>4 Not Applicable
	19	cross_section_05	5. Were confounding factors identified?	radio (Matrix)<br>1 Yes<br>2 No<br>3 Unclear<br>4 Not Applicable
	20	cross_section_06	6. Were strategies to deal with confounding factors stated?	radio (Matrix)<br>1 Yes<br>2 No<br>3 Unclear<br>4 Not Applicable
	21	cross_section_07	7. Were the outcomes measured in a valid and reliable way?	radio (Matrix)<br>1 Yes<br>2 No<br>3 Unclear<br>4 Not Applicable
	22	cross_section_08	8. Was appropriate statistical analysis used?	radio (Matrix)<br>1 Yes<br>2 No<br>3 Unclear<br>4 Not Applicable
	23	jbi_critical_appraisal_checklist_for_analytical_cr_complete	Section Header: *Form Status*<br>Complete?	dropdown<br>0 Incomplete<br>1 Unverified<br>2 Complete
Instrument: **JBI Critical Appraisal Checklist for Case Control Studies** (jbi_critical_appraisal_checklist_for_case_control) Collapse				
	24	reviewer_name_case	Section Header: *Reviewer Details*<br>Reviewer	dropdown<br>1 Karl Morgan<br>2 James Cowburn<br>3 Matthew Farrow
	25	reviewer_date_case	Date reviewed<br>*Just press 'Today'*	text (date_dmy)
	26	authors_case	Section Header: *Study Information*<br>Authors	text

	27	year_case	Publication year	text
	28	record_number_case	Record number	text
	29	case_control_01	Section Header: *JBI Critical Appraisal Checklist for Case Control Studies*<br>1. Were the groups comparable other than the presence of disease in cases or the absence of disease in controls?	radio (Matrix)<br>1 Yes<br>2 No<br>3 Unclear<br>4 Not Applicable
	30	case_control_02	2. Were cases and controls matched appropriately?	radio (Matrix)<br>1 Yes<br>2 No<br>3 Unclear<br>4 Not Applicable
	31	case_control_03	3. Were the same criteria used for identification of cases and controls?	radio (Matrix)<br>1 Yes<br>2 No<br>3 Unclear<br>4 Not Applicable
	32	case_control_04	4. Was exposure measured in a standard, valid and reliable way?	radio (Matrix)<br>1 Yes<br>2 No<br>3 Unclear<br>4 Not Applicable
	33	case_control_05	5. Was exposure measured in the same way for cases and controls?	radio (Matrix)<br>1 Yes<br>2 No<br>3 Unclear<br>4 Not Applicable
	34	case_control_06	6. Were confounding factors identified?	radio (Matrix)<br>1 Yes<br>2 No<br>3 Unclear<br>4 Not Applicable
	35	case_control_07	7. Were strategies to deal with confounding factors stated?	radio (Matrix)<br>1 Yes<br>2 No<br>3 Unclear<br>4 Not Applicable
	36	case_control_08	8. Were outcomes assessed in a standard, valid and reliable way for cases and controls?	radio (Matrix)<br>1 Yes<br>2 No<br>3 Unclear<br>4 Not Applicable
	37	case_control_09	9. Was the exposure period of interest long enough to be meaningful?	radio (Matrix)<br>1 Yes<br>2 No<br>3 Unclear<br>4 Not Applicable
	38	case_control_10	10. Was appropriate statistical analysis used?	radio (Matrix)<br>1 Yes<br>2 No<br>3 Unclear<br>4 Not Applicable

	39	jbi_critical_appraisal_checklist_for_case_control_complete	Section Header: *Form Status*<br>Complete?	dropdown<br>0 Incomplete<br>1 Unverified<br>2 Complete
Instrument: **JBI Critical Appraisal Checklist for Cohort Studies** (jbi_critical_appraisal_checklist_for_cohort_studie) Collapse				
	40	reviewer_name_cohort	Section Header: *Reviewer Details*<br>Reviewer	dropdown<br>1 Karl Morgan<br>2 James Cowburn<br>3 Matthew Farrow
	41	reviewer_date_cohort	Date reviewed<br>*Just press 'Today'*	text (date_dmy)
	42	authors_cohort	Section Header: *Study Information*<br>Authors<br>*Surname*	text
	43	year_cohort	Publication year<br>*e.g., 2010*	text
	44	record_number_cohort	Record number	text
	45	cohort_01	Section Header: *JBI Critical Appraisal Checklist for Cohort Studies*<br>1. Were the two groups similar and recruited from the same population?	radio (Matrix)<br>1 Yes<br>2 No<br>3 Unclear<br>4 Not Applicable
	46	cohort_02	2. Were the exposures measured similarly to assign people to both exposed and unexposed groups?	radio (Matrix)<br>1 Yes<br>2 No<br>3 Unclear<br>4 Not Applicable
	47	cohort_03	3. Was the exposure measured in a valid and reliable way?	radio (Matrix)<br>1 Yes<br>2 No<br>3 Unclear<br>4 Not Applicable
	48	cohort_04	4. Were confounding factors identified?	radio (Matrix)<br>1 Yes<br>2 No<br>3 Unclear<br>4 Not Applicable
	49	cohort_05	5. Were strategies to deal with confounding factors stated?	radio (Matrix)<br>1 Yes<br>2 No<br>3 Unclear<br>4 Not Applicable
	50	cohort_06	6. Were the groups/participants free of the outcome at the start of the study (or at the moment of exposure)?	radio (Matrix)<br>1 Yes<br>2 No<br>3 Unclear<br>4 Not Applicable
	51	cohort_07	7. Were the outcomes measured in a valid and reliable way?	radio (Matrix)<br>1 Yes<br>2 No<br>3 Unclear<br>4 Not Applicable

	52	cohort_08	8. Was the follow up time reported and sufficient to be long enough for outcomes to occur?	radio (Matrix)<br>1 Yes<br>2 No<br>3 Unclear<br>4 Not Applicable
	53	cohort_09	9. Was follow up complete, and if not, were the reasons to loss to follow up described and explored?	radio (Matrix)<br>1 Yes<br>2 No<br>3 Unclear<br>4 Not Applicable
	54	cohort_10	10. Were strategies to address incomplete follow up utilized?	radio (Matrix)<br>1 Yes<br>2 No<br>3 Unclear<br>4 Not Applicable
	55	cohort_11	11. Was appropriate statistical analysis used?	radio (Matrix)<br>1 Yes<br>2 No<br>3 Unclear<br>4 Not Applicable
	56	jbi_critical_appraisal_checklist_for_cohort_studie_complete	Section Header: *Form Status*<br>Complete?	dropdown<br>0 Incomplete<br>1 Unverified<br>2 Complete
Instrument: **JBI Critical Appraisal Checklist for Studies Reporting Prevalence Data** (jbi_critical_appraisal_checklist_for_studies_repor) Collapse				
	57	reviewer_name_prevalence	Section Header: *Reviewer Details*<br>Reviewer	dropdown<br>1 Karl Morgan<br>2 James Cowburn<br>3 Matthew Farrow
	58	reviewer_date_prevalence	Date reviewed<br>*Just press 'Today'*	text (date_dmy)
	59	authors_prevalence	Section Header: *Study Information*<br>Authors	text
	60	year_prevalence	Publication year	text
	61	record_number_prevalence	Record number	text
	62	prevalence_01	Section Header: *JBI Critical Appraisal Checklist for Studies Reporting Prevalence Data*<br>1. Was the sample frame appropriate to address the target population?	radio (Matrix)<br>1 Yes<br>2 No<br>3 Unclear<br>4 Not Applicable
	63	prevalence_02	2. Were study participants sampled in an appropriate way?	radio (Matrix)<br>1 Yes<br>2 No<br>3 Unclear<br>4 Not Applicable
	64	prevalence_03	3. Was the sample size adequate?	radio (Matrix)<br>1 Yes<br>2 No<br>3 Unclear<br>4 Not Applicable

	65	prevalence_04	4. Were the study subjects and the setting described in detail?	radio (Matrix)<br>1 Yes<br>2 No<br>3 Unclear<br>4 Not Applicable
	66	prevalence_05	5. Was the data analysis conducted with sufficient coverage of the identified sample?	radio (Matrix)<br>1 Yes<br>2 No<br>3 Unclear<br>4 Not Applicable
	67	prevalence_06	6. Were valid methods used for the identification of the condition?	radio (Matrix)<br>1 Yes<br>2 No<br>3 Unclear<br>4 Not Applicable
	68	prevalence_07	7. Was the condition measured in a standard, reliable way for all participants?	radio (Matrix)<br>1 Yes<br>2 No<br>3 Unclear<br>4 Not Applicable
	69	prevalence_08	8. Was there appropriate statistical analysis?	radio (Matrix)<br>1 Yes<br>2 No<br>3 Unclear<br>4 Not Applicable
	70	prevalence_09	9. Was the response rate adequate, and if not, was the low response rate managed appropriately?	radio (Matrix)<br>1 Yes<br>2 No<br>3 Unclear<br>4 Not Applicable
	71	jbi_critical_appraisal_checklist_for_studies_repor_complete	Section Header: *Form Status*<br>Complete?	dropdown<br>0 Incomplete<br>1 Unverified<br>2 Complete
Instrument: **JBI Critical Appraisal Checklist for Quasi-Experimental Studies** (jbi_critical_appraisal_checklist_for_quasiexperime) Collapse				
	72	reviewer_name_quasi	Section Header: *Reviewer Details*<br>Reviewer	dropdown<br>1 Karl Morgan<br>2 James Cowburn<br>3 Matthew Farrow
	73	reviewer_date_quasi	Date reviewed<br>*Just press 'Today'*	text (date_dmy)
	74	author_quasi	Section Header: *Study Information*<br>Authors	text
	75	year_quasi	Publication year	text
	76	record_number_quasi	Record number	text
	77	quasi_experimental_01	Section Header: *JBI Critical Appraisal Checklist for Quasi-Experimental Studies*<br>1. Is it clear in the study what is the 'cause' and what is the 'effect' (i.e. there is no confusion about which variable comes first)?	radio (Matrix)<br>1 Yes<br>2 No<br>3 Unclear<br>4 Not Applicable

	78	quasi_experimental_02	2. Were the participants included in any comparisons similar?	radio (Matrix)<br>1 Yes<br>2 No<br>3 Unclear<br>4 Not Applicable
	79	quasi_experimental_03	3. Were the participants included in any comparisons receiving similar treatment/care, other than the exposure or intervention of interest?	radio (Matrix)<br>1 Yes<br>2 No<br>3 Unclear<br>4 Not Applicable
	80	quasi_experimental_04	4. Was there a control group?	radio (Matrix)<br>1 Yes<br>2 No<br>3 Unclear<br>4 Not Applicable
	81	quasi_experimental_05	5. Were there multiple measurements of the outcome both pre and post the intervention/exposure?	radio (Matrix)<br>1 Yes<br>2 No<br>3 Unclear<br>4 Not Applicable
	82	quasi_experimental_06	6. Was follow up complete and if not, were differences between groups in terms of their follow up adequately described and analyzed?	radio (Matrix)<br>1 Yes<br>2 No<br>3 Unclear<br>4 Not Applicable
	83	quasi_experimental_07	7. Were the outcomes of participants included in any comparisons measured in the same way?	radio (Matrix)<br>1 Yes<br>2 No<br>3 Unclear<br>4 Not Applicable
	84	quasi_experimental_08	8. Were outcomes measured in a reliable way?	radio (Matrix)<br>1 Yes<br>2 No<br>3 Unclear<br>4 Not Applicable
	85	quasi_experimental_09	9. Was appropriate statistical analysis used?	radio (Matrix)<br>1 Yes<br>2 No<br>3 Unclear<br>4 Not Applicable
	86	jbi_critical_appraisal_checklist_for_quasiexperime_complete	Section Header: *Form Status*<br>Complete?	dropdown<br>0 Incomplete<br>1 Unverified<br>2 Complete
Instrument: **JBI Critical Appraisal Checklist for Randomised Controlled Trials** (jbi_critical_appraisal_checklist_for_randomised_co) Collapse				
	87	reviewer_name_rct	Section Header: *Reviewer Details*<br>Reviewer	dropdown<br>1 Karl Morgan<br>2 James Cowburn<br>3 Matthew Farrow
	88	reviewer_date_rct	Date reviewed<br>*Just press 'Today'*	text (date_dmy)

	89	authors_rct	Section Header: *Study Information*<br>Authors	text
	90	year_rct	Publication year	text
	91	record_number_rct	Record number	text
	92	rct_01	Section Header: *JBI Critical Appraisal Checklist for Randomised Controlled Trials*<br>1. Was true randomization used for assignment of participants to treatment groups?	radio (Matrix)<br>1 Yes<br>2 No<br>3 Unclear<br>4 Not Applicable
	93	rct_02	2. Was allocation to treatment groups concealed?	radio (Matrix)<br>1 Yes<br>2 No<br>3 Unclear<br>4 Not Applicable
	94	rct_03	3. Were treatment groups similar at the baseline?	radio (Matrix)<br>1 Yes<br>2 No<br>3 Unclear<br>4 Not Applicable
	95	rct_04	4. Were participants blind to treatment assignment?	radio (Matrix)<br>1 Yes<br>2 No<br>3 Unclear<br>4 Not Applicable
	96	rct_05	5. Were those delivering treatment blind to treatment assignment?	radio (Matrix)<br>1 Yes<br>2 No<br>3 Unclear<br>4 Not Applicable
	97	rct_06	6. Were outcomes assessors blind to treatment assignment?	radio (Matrix)<br>1 Yes<br>2 No<br>3 Unclear<br>4 Not Applicable
	98	rct_07	7. Were treatment groups treated identically other than the intervention of interest?	radio (Matrix)<br>1 Yes<br>2 No<br>3 Unclear<br>4 Not Applicable
	99	rct_08	8. Was follow up complete and if not, were differences between groups in terms of their follow up adequately described and analyzed?	radio (Matrix)<br>1 Yes<br>2 No<br>3 Unclear<br>4 Not Applicable
	100	rct_09	9. Were participants analyzed in the groups to which they were randomized?	radio (Matrix)<br>1 Yes<br>2 No<br>3 Unclear<br>4 Not Applicable

	101	rct_10	10. Were outcomes measured in the same way for treatment groups?	radio (Matrix)<br>1 Yes<br>2 No<br>3 Unclear<br>4 Not Applicable
	102	rct_11	11. Were outcomes measured in a reliable way?	radio (Matrix)<br>1 Yes<br>2 No<br>3 Unclear<br>4 Not Applicable
	103	rct_12	12. Was appropriate statistical analysis used?	radio (Matrix)<br>1 Yes<br>2 No<br>3 Unclear<br>4 Not Applicable
	104	rct_13	13. Was the trial design appropriate, and any deviations from the standard RCT design (individual randomization, parallel groups) accounted for in the conduct and analysis of the trial?	radio (Matrix)<br>1 Yes<br>2 No<br>3 Unclear<br>4 Not Applicable
	105	jbi_critical_appraisal_checklist_for_randomised_co_complete	Section Header: *Form Status*<br>Complete?	dropdown<br>0 Incomplete<br>1 Unverified<br>2 Complete
Instrument: **JBI Critical Appraisal Checklist for Systematic Reviews and Research Syntheses** (jbi_critical_appraisal_checklist_for_systematic_re) Collapse				
	106	reviewer_name_review	Section Header: *Study Information*<br>Reviewer	dropdown<br>1 Karl Morgan<br>2 James Cowburn<br>3 Matthew Farrow
	107	reviewer_date_review	Date reviewed<br>*Just press 'Today'*	text (date_dmy)
	108	authors_review	Section Header: *Study Information*<br>Authors	text
	109	year_review	Publication year	text
	110	record_number_review	Record number	text
	111	reviews_01	Section Header: *JBI Critical Appraisal Checklist for Systematic Reviews and Research Syntheses*<br>1. Is the review question clearly and explicitly stated?	radio (Matrix)<br>1 Yes<br>2 No<br>3 Unclear<br>4 Not Applicable
	112	reviews_02	2. Were the inclusion criteria appropriate for the review question?	radio (Matrix)<br>1 Yes<br>2 No<br>3 Unclear<br>4 Not Applicable
	113	reviews_03	3. Was the search strategy appropriate?	radio (Matrix)<br>1 Yes<br>2 No<br>3 Unclear<br>4 Not Applicable

	114	reviews_04	4. Were the sources and resources used to search for studies adequate?	radio (Matrix)<br>1 Yes<br>2 No<br>3 Unclear<br>4 Not Applicable
	115	reviews_05	5. Were the criteria for appraising studies appropriate?	radio (Matrix)<br>1 Yes<br>2 No<br>3 Unclear<br>4 Not Applicable
	116	reviews_06	6. Was critical appraisal conducted by two or more reviewers independently?	radio (Matrix)<br>1 Yes<br>2 No<br>3 Unclear<br>4 Not Applicable
	117	reviews_07	7. Were there methods to minimize errors in data extraction?	radio (Matrix)<br>1 Yes<br>2 No<br>3 Unclear<br>4 Not Applicable
	118	reviews_08	8. Were the methods used to combine studies appropriate?	radio (Matrix)<br>1 Yes<br>2 No<br>3 Unclear<br>4 Not Applicable
	119	reviews_09	9. Was the likelihood of publication bias assessed?	radio (Matrix)<br>1 Yes<br>2 No<br>3 Unclear<br>4 Not Applicable
	120	reviews_10	10. Were recommendations for policy and/or practice supported by the reported data?	radio (Matrix)<br>1 Yes<br>2 No<br>3 Unclear<br>4 Not Applicable
	121	reviews_11	11. Were the specific directives for new research appropriate?	radio (Matrix)<br>1 Yes<br>2 No<br>3 Unclear<br>4 Not Applicable
	122	jbi_critical_appraisal_checklist_for_systematic_re_complete	Section Header: *Form Status*<br>Complete?	dropdown<br>0 Incomplete<br>1 Unverified<br>2 Complete

Data Charting Draft

📖 Codebook

📖 Data Dictionary Codebook

2022-12-22 21:44:12

Collapse all instruments

	#	Variable / Field Name	Field Label<br>*Field Note*	Field Attributes (Field Type, Validation, Choices, Calculations, etc.)
Instrument: **Data Extraction 2** (data_extraction_2) Collapse				
	1	record_id	Record ID	text
	2	reviewer_name_extraction_v2	Reviewer	dropdown, Required<br>1 Karl Morgan<br>2 James Cowburn<br>3 Mathew Farrow<br>4 Josh Carter
	3	reviewer_date_extraction_v2	Date of extraction<br>*Just press 'Today'*	text (date_dmy), Required
	4	record_number_extraction_v2	Section Header: *Study Information*<br>Record number	text, Required
	5	first_author_v2	First author<br>*Format as: second name, first name initial (e.g., Morgan, K.).*	text, Required
	6	co_authors_v2	Co-authors<br>*Format as: Initial followed by surname (e.g., Morgan, K.). Write n/a if none.*	notes, Required
	7	publication_year_v2	Publication year<br>*Please insert as number (e.g., 2006).*	text (number), Required
	8	country_research_origin_v2	Country of research origin<br><br>ISO Alpha 3 codes<br>*Please use ISO Alpha 3 codes (e.g., USA, GBR, FRA, DNK, CAN). Write unclear if unknown.*	text, Required
	9	study_aim_1_v2	Section Header: *Study aims*<br>Study aim 1<br>*Write unclear if unknown.*	notes, Required
	10	study_aim_2_v2	Study aim 2<br>*Write unclear if unknown. If no secondary aim write n/a.*	notes, Required
	11	study_aim_3_v2	Study aim 3<br>*Write unclear if unknown. If no tertiary aim write n/a.*	notes, Required
	12	study_design_v2	Section Header: *Methodology - Study Design*<br>Study design	radio, Required<br>1 Analytical cross-sectional<br>2 Case Control<br>3 Cohort<br>4 Prevalence data<br>5 Quasi-experimental<br>6 Randomised control trial<br>7 Unclear
	13	number_of_participants_v2	Section Header: *Methodology - Experimental Population Size*<br>Total number of participants<br>*Report as: number*	text (number), Required
	14	number_of_males_v2	Number of males<br>*Report as: number*	text, Required
	15	number_of_females_v2	Number of females<br>*Report as: number*	text, Required
	16	age_heading_v2	Section Header: *Methodology - Experimental Group Demographic Information*<br>Experimental Group Age	descriptive
	17	total_population_age_v2	Total Population	descriptive

	18	participant_age_total_v2	How was the age of the total study population reported?<br>*Select all relevant answers*	checkbox, Required<br>1 participant_age_total_v2___1 Mean<br>2 participant_age_total_v2___2 Standard deviation<br>3 participant_age_total_v2___3 Median<br>4 participant_age_total_v2___4 Range
	19	age_mean_v2<br>Show the field ONLY if: [participant_age_total_v2(1)] = '1'	Mean<br>*Years*	text, Required
	20	age_stdev_v2<br>Show the field ONLY if: [participant_age_total_v2(2)] = '1'	Standard deviation<br>*Years*	text, Required
	21	age_median_v2<br>Show the field ONLY if: [participant_age_total_v2(3)] = '1'	Median<br>*Years*	text, Required
	22	age_range_v2<br>Show the field ONLY if: [participant_age_total_v2(4)] = '1'	Range<br>*Years*	text, Required
	23	male_population_age_v2	Male Population	descriptive
	24	participant_age_male_v2	How was the age of the male study population reported?<br>*Select all relevant answers*	checkbox, Required<br>1 participant_age_male_v2___1 Mean<br>2 participant_age_male_v2___2 Standard deviation<br>3 participant_age_male_v2___3 Median<br>4 participant_age_male_v2___4 Range
	25	age_mean_male_v2<br>Show the field ONLY if: [participant_age_male_v2(1)] = '1'	Mean<br>*Years*	text, Required
	26	age_stdev_male_v2<br>Show the field ONLY if: [participant_age_male_v2(2)] = '1'	Standard deviation<br>*Years*	text, Required
	27	age_median_male_v2<br>Show the field ONLY if: [participant_age_male_v2(3)] = '1'	Median<br>*Years*	text, Required
	28	age_range_male_v2<br>Show the field ONLY if: [participant_age_male_v2(4)] = '1'	Range<br>*Years*	text, Required
	29	population_age_female_v2	Female Population	descriptive
	30	participant_age_female_v2	How was the age of the female study population reported?<br>*Select all relevant answers*	checkbox, Required<br>1 participant_age_female_v2___1 Mean<br>2 participant_age_female_v2___2 Standard deviation<br>3 participant_age_female_v2___3 Median<br>4 participant_age_female_v2___4 Range
	31	age_mean_female_v2<br>Show the field ONLY if: [participant_age_female_v2 (1)] = '1'	Mean<br>*Years*	text, Required
	32	age_stdev_female_v2<br>Show the field ONLY if: [participant_age_female_v2 (2)] = '1'	Standard deviation<br>*Years*	text, Required

	33	age_median_female_v2<br>Show the field ONLY if:<br>[participant_age_female_v2(3)] = '1'	Median<br>*Years*	text, Required
	34	age_range_female_v2<br>Show the field ONLY if:<br>[participant_age_female_v2(4)] = '1'	Range<br>*Years*	text, Required
	35	participant_anth_info_v2	Participant Anthropometric Information	descriptive
	36	total_population_anthro_v2	Total Population	descriptive
	37	participant_height_v2	How was participant height reported?<br>*Meters*	checkbox, Required<br>1 participant_height_v2___1 Mean<br>2 participant_height_v2___2 Standard deviation<br>3 participant_height_v2___3 Median<br>4 participant_height_v2___4 Range
	38	participant_height_mean_v2<br>Show the field ONLY if:<br>[participant_height_v2(1)] = '1'	Mean	text, Required
	39	participant_height_stdev_v2<br>Show the field ONLY if:<br>[participant_height_v2(2)] = '1'	Standard deviation	text, Required
	40	participant_height_median_v2<br>Show the field ONLY if:<br>[participant_height_v2(3)] = '1'	Median	text, Required
	41	participant_height_range_v2<br>Show the field ONLY if:<br>[participant_height_v2(4)] = '1'	Range	text, Required
	42	participant_mass_v2	Participant mass<br>*Kilograms*	checkbox, Required<br>1 participant_mass_v2___1 Mean<br>2 participant_mass_v2___2 Standard deviation<br>3 participant_mass_v2___3 Median<br>4 participant_mass_v2___4 Range
	43	participant_mass_mean_v2<br>Show the field ONLY if:<br>[participant_mass_v2(1)] = '1'	Mean	text, Required
	44	participant_mass_stdev_v2<br>Show the field ONLY if:<br>[participant_mass_v2(2)] = '1'	Standard deviation	text, Required
	45	participant_mass_median_v2<br>Show the field ONLY if:<br>[participant_mass_v2(3)] = '1'	Median	text, Required
	46	participant_mass_range_v2<br>Show the field ONLY if:<br>[participant_mass_v2(4)] = '1'	Range	text, Required
	47	participant_bmi_v2	Participant BMI<br>*Kilograms/meters^2*	checkbox, Required<br>1 participant_bmi_v2___1 Mean<br>2 participant_bmi_v2___2 Standard deviation<br>3 participant_bmi_v2___3 Median<br>4 participant_bmi_v2___4 Range
	48	participant_bmi_mean_v2<br>Show the field ONLY if:<br>[participant_bmi_v2(1)] = '1'	Mean	text, Required
	49	participant_bmi_stdev_v2<br>Show the field ONLY if:<br>[participant_bmi_v2(2)] = '1'	Standard deviation	text, Required
	50	participant_bmi_median_v2<br>Show the field ONLY if:<br>[participant_bmi_v2(3)] = '1'	Median	text, Required

For peer review only - http://bmjopen.bmj.com/site/about/guidelines.xhtml

	51	participant_bmi_range_v2<br>Show the field ONLY if:<br>[participant_bmi_v2(4)] = '1'	Range	text, Required
	52	male_population_anthro_v2	Male Population	descriptive
	53	male_exp_pp_height_v2	How was participant height reported?<br>*Meters*	checkbox, Required<br>1 male_exp_pp_height_v2___1 Mean<br>2 male_exp_pp_height_v2___2 Standard deviation<br>3 male_exp_pp_height_v2___3 Median<br>4 male_exp_pp_height_v2___4 Range
	54	male_exp_mean_height_v2<br>Show the field ONLY if:<br>[male_exp_pp_height_v2(1)] = '1'	Mean	text, Required
	55	male_exp_stdev_height_v2<br>Show the field ONLY if:<br>[male_exp_pp_height_v2(2)] = '1'	Standard deviation	text, Required
	56	male_exp_median_height_v2<br>Show the field ONLY if:<br>[male_exp_pp_height_v2(3)] = '1'	Median	text, Required
	57	male_exp_range_height_v2<br>Show the field ONLY if:<br>[male_exp_pp_height_v2(4)] = '1'	Range	text, Required
	58	male_exp_pp_mass_v2	How was participant mass reported?<br>*Meters*	checkbox, Required<br>1 male_exp_pp_mass_v2___1 Mean<br>2 male_exp_pp_mass_v2___2 Standard deviation<br>3 male_exp_pp_mass_v2___3 Median<br>4 male_exp_pp_mass_v2___4 Range
	59	male_exp_mean_mass_v2<br>Show the field ONLY if:<br>[male_exp_pp_mass_v2(1)] = '1'	Mean	text, Required
	60	male_exp_stdev_mass_v2<br>Show the field ONLY if:<br>[male_exp_pp_mass_v2(2)] = '1'	Standard deviation	text, Required
	61	male_exp_median_mass_v2<br>Show the field ONLY if:<br>[male_exp_pp_mass_v2(3)] = '1'	Median	text, Required
	62	male_exp_range_mass_v2<br>Show the field ONLY if:<br>[male_exp_pp_mass_v2(4)] = '1'	Range	text, Required
	63	male_exp_pp_bmi_v2	How was participant body mass index reported?<br>*Meters*	checkbox, Required<br>1 male_exp_pp_bmi_v2___1 Mean<br>2 male_exp_pp_bmi_v2___2 Standard deviation<br>3 male_exp_pp_bmi_v2___3 Median<br>4 male_exp_pp_bmi_v2___4 Range
	64	male_exp_mean_bmi_v2<br>Show the field ONLY if:<br>[male_exp_pp_bmi_v2(1)] = '1'	Mean	text, Required
	65	male_exp_stdev_bmi_v2<br>Show the field ONLY if:<br>[male_exp_pp_bmi_v2(2)] = '1'	Standard deviation	text, Required
	66	male_exp_median_bmi_v2<br>Show the field ONLY if:<br>[male_exp_pp_bmi_v2(3)] = '1'	Median	text, Required

For peer review only - http://bmjopen.bmj.com/site/about/guidelines.xhtml

	67	male_exp_range_bmi_v2<br>Show the field ONLY if:<br>[male_exp_pp_bmi_v2(4)] = '1'	Range	text, Required
	68	female_population_anthro_v2	Female Population	descriptive
	69	female_exp_pp_height_v2	How was participant height reported?<br>*Meters*	checkbox, Required<br>1 female_exp_pp_height_v2___1 Mean<br>2 female_exp_pp_height_v2___2 Standard deviation<br>3 female_exp_pp_height_v2___3 Median<br>4 female_exp_pp_height_v2___4 Range
	70	female_exp_mean_height_v2<br>Show the field ONLY if:<br>[female_exp_pp_height_v2(1)] = '1'	Mean	text, Required
	71	female_exp_sdtev_height_v2<br>Show the field ONLY if:<br>[female_exp_pp_height_v2(2)] = '1'	Standard deviation	text, Required
	72	female_exp_median_height_v2<br>Show the field ONLY if:<br>[female_exp_pp_height_v2(3)] = '1'	Median	text, Required
	73	female_exp_range_height_v2<br>Show the field ONLY if:<br>[female_exp_pp_height_v2(4)] = '1'	Range	text, Required
	74	female_exp_pp_mass_v2	How was participant mass reported?<br>*Meters*	checkbox, Required<br>1 female_exp_pp_mass_v2___1 Mean<br>2 female_exp_pp_mass_v2___2 Standard deviation<br>3 female_exp_pp_mass_v2___3 Median<br>4 female_exp_pp_mass_v2___4 Range
	75	female_exp_mean_mass_v2<br>Show the field ONLY if:<br>[female_exp_pp_mass_v2(1)] = '1'	Mean	text, Required
	76	female_exp_stdev_mass_v2<br>Show the field ONLY if:<br>[female_exp_pp_mass_v2(2)] = '1'	Standard deviation	text, Required
	77	female_exp_median_mass_v2<br>Show the field ONLY if:<br>[female_exp_pp_mass_v2(3)] = '1'	Median	text, Required
	78	female_exp_range_mass_v2<br>Show the field ONLY if:<br>[female_exp_pp_mass_v2(4)] = '1'	Range	text, Required
	79	female_exp_pp_bmi_v2	How was participant body mass index reported?<br>*Meters*	checkbox, Required<br>1 female_exp_pp_bmi_v2___1 Mean<br>2 female_exp_pp_bmi_v2___2 Standard deviation<br>3 female_exp_pp_bmi_v2___3 Median<br>4 female_exp_pp_bmi_v2___4 Range
	80	female_exp_mean_bmi_v2<br>Show the field ONLY if:<br>[female_exp_pp_bmi_v2(1)] = '1'	Mean	text, Required

	81	female_exp_stdev_bmi_v2<br><br>Show the field ONLY if:<br>[female_exp_pp_bmi_v2(2)] = '1'	Standard deviation	text, Required
	82	female_exp_median_bmi_v2<br><br>Show the field ONLY if:<br>[female_exp_pp_bmi_v2(3)] = '1'	Median	text, Required
	83	female_exp_range_bmi_v2<br><br>Show the field ONLY if:<br>[female_exp_pp_bmi_v2(4)] = '1'	Range	text, Required
	84	number_of_controls_v2<br><br>Show the field ONLY if:<br>[cntrl_participants_included] = 1	Section Header: *Methodology - Control Population Size*<br>Number of controls<br>*Report as: number*	text (number), Required
	85	number_of_males_control_v2<br><br>Show the field ONLY if:<br>[cntrl_participants_included] = 1	Number of control males<br>*Report as: number*	text, Required
	86	number_of_females_control_v2<br><br>Show the field ONLY if:<br>[number_of_controls_v2] = 1	Number of control females<br>*Report as: number*	text, Required
	87	cntrl_group_age_v2	Section Header: *Methodology - Control Demographic Information*<br>Control Group Age	descriptive
	88	total_cntrl_age_v2	Total Population	descriptive
	89	participant_cntrl_age_type_v2<br><br>Show the field ONLY if:<br>[number_of_controls_v2] = 1	How was the age of control participants reported?<br>*Select all relevant answers*	checkbox, Required<br>1 participant_cntrl_age_type_v2___1 Mean<br>2 participant_cntrl_age_type_v2___2 Standard deviation<br>3 participant_cntrl_age_type_v2___3 Median<br>4 participant_cntrl_age_type_v2___4 Range
	90	age_cntrl_mean_v2<br><br>Show the field ONLY if:<br>[participant_cntrl_age_type_v2(1)] = '1'	Mean	text, Required
	91	age_cntrl_stdev_v2<br><br>Show the field ONLY if:<br>[participant_cntrl_age_type_v2(2)] = '1'	Standard deviation	text, Required
	92	age_cntrl_median_v2<br><br>Show the field ONLY if:<br>[participant_cntrl_age_type_v2(3)] = '1'	Median	text, Required
	93	age_cntrl_range_v2<br><br>Show the field ONLY if:<br>[participant_cntrl_age_type_v2(4)] = '1'	Range	text, Required
	94	male_cntrl_age_v2	Male Population	descriptive
	95	male_pp_cntrl_age_type_v2	How was the age of control participants reported?<br>*Select all relevant answers*	checkbox, Required<br>1 male_pp_cntrl_age_type_v2___1 Mean<br>2 male_pp_cntrl_age_type_v2___2 Standard deviation<br>3 male_pp_cntrl_age_type_v2___3 Median<br>4 male_pp_cntrl_age_type_v2___4 Range
	96	age_cntrl_male_mean_v2<br><br>Show the field ONLY if:<br>[male_pp_cntrl_age_type_v2(1)] = '1'	Mean	text

	97	age_cntrl_male_stdev_v2<br>Show the field ONLY if:<br>[male_pp_cntrl_age_type_v2(2)] = '1'	Standard deviation	text
	98	age_cntrl_male_median_v2<br>Show the field ONLY if:<br>[male_pp_cntrl_age_type_v2(3)] = '1'	Median	text
	99	age_cntrl_male_range_v2<br>Show the field ONLY if:<br>[male_pp_cntrl_age_type_v2(4)] = '1'	Range	text
	100	female_cntrl_age_v2	Female Population	descriptive
	101	female_pp_cntrl_age_type_v2	How was the age of control participants reported?<br>*Select all relevant answers*	checkbox, Required<br>1 | female_pp_cntrl_age_type_v2___1 | Mean<br>2 | female_pp_cntrl_age_type_v2___2 | Standard deviation<br>3 | female_pp_cntrl_age_type_v2___3 | Median<br>4 | female_pp_cntrl_age_type_v2___4 | Range
	102	age_cntrl_female_mean_v2<br>Show the field ONLY if:<br>[female_pp_cntrl_age_type_v2(1)] = '1'	Mean	text
	103	age_cntrl_female_stdev_v2<br>Show the field ONLY if:<br>[female_pp_cntrl_age_type_v2(2)] = '1'	Standard deviation	text
	104	age_cntrl_female_median_v2<br>Show the field ONLY if:<br>[female_pp_cntrl_age_type_v2(3)] = '1'	Median	text
	105	age_cntrl_female_range_v2<br>Show the field ONLY if:<br>[female_pp_cntrl_age_type_v2(4)] = '1'	Range	text
	106	cntrl_anth_info_v2	Control Anthropometric Information	descriptive
	107	cntrl_anth_info_2_v2	Total Population	descriptive
	108	control_height_v2<br>Show the field ONLY if:<br>[number_of_controls_v2] = 1	Control height<br>*Meters*	text, Required
	109	control_mass_v2<br>Show the field ONLY if:<br>[number_of_controls_v2] = 1	Control mass<br>*Kilograms*	text, Required
	110	control_bmi_v2<br>Show the field ONLY if:<br>[number_of_controls_v2] = 1	Control body mass index<br>*Kilograms/meters^2*	text, Required
	111	male_cntrl_anth_info_v2	Male Population	descriptive
	112	female_cntrl_anth_info_v2	Female Population	descriptive
	113	injury_type_v2	Section Header: *Injury History*<br>Type of knee injury<br>*Select all relevant answers*	checkbox, Required<br>1 | injury_type_v2___1 | Intra-articular (general unspecified)<br>2 | injury_type_v2___2 | Ligament<br>3 | injury_type_v2___3 | Meniscus<br>4 | injury_type_v2___4 | Fracture<br>5 | injury_type_v2___5 | Dislocation<br>6 | injury_type_v2___6 | Contusion<br>7 | injury_type_v2___7 | Unclear

	114	time_since_injury_type_v2	How was time between injury incidence and observation/follow up reported?<br>*Select all relevant answers*	checkbox, Required<br>1 | time_since_injury_type_v2___1 | Mean<br>2 | time_since_injury_type_v2___2 | Standard deviation<br>3 | time_since_injury_type_v2___3 | Median<br>4 | time_since_injury_type_v2___4 | Range<br>5 | time_since_injury_type_v2___5 | Not reported
	115	time_since_injury_mean_v2<br>Show the field ONLY if:<br>[time_since_injury_type_v2(1)] = '1'	Mean	text (number), Required
	116	time_since_injury_stdev_v2<br>Show the field ONLY if:<br>[time_since_injury_type_v2(2)] = '1'	Standard deviation	text (number), Required
	117	time_since_injury_median_v2<br>Show the field ONLY if:<br>[time_since_injury_type_v2(3)] = '1'	Median	text (number), Required
	118	time_since_injury_range_v2<br>Show the field ONLY if:<br>[time_since_injury_type_v2(4)] = '1'	Range	text (number), Required
	119	knee_surgery_v2	Section Header: *Surgery History*<br>Did the participants have knee surgery?	yesno, Required<br>1 | Yes<br>0 | No
	120	surgery_type_v2<br>Show the field ONLY if:<br>[knee_surgery_v2] = '1'	Type of knee surgery<br>*Select all relevant answers*	checkbox, Required<br>1 | surgery_type_v2___1 | Tendon graft<br>2 | surgery_type_v2___2 | Meniscectomy<br>3 | surgery_type_v2___3 | Chondroplasty<br>7 | surgery_type_v2___7 | Unclear
	121	time_since_surgery_type_v2<br>Show the field ONLY if:<br>[knee_surgery_v2] = '1'	How was time between between surgery and observation/follow up reported?<br>*Select all relevant answers*	checkbox, Required<br>1 | time_since_surgery_type_v2___1 | Mean<br>2 | time_since_surgery_type_v2___2 | Standard deviation<br>3 | time_since_surgery_type_v2___3 | Median<br>4 | time_since_surgery_type_v2___4 | Range<br>5 | time_since_surgery_type_v2___5 | Not reported
	122	time_since_surgery_mean_v2<br>Show the field ONLY if:<br>[time_since_surgery_type_v2(1)] = 1	Mean	text, Required
	123	time_since_surgery_stdev_v2<br>Show the field ONLY if:<br>[time_since_surgery_type_v2(2)] = 1	Standard deviation	text, Required
	124	time_since_surgery_median_v2<br>Show the field ONLY if:<br>[time_since_surgery_type_v2(3)] = 1	Median	text, Required
	125	time_since_surgery_range_v2<br>Show the field ONLY if:<br>[time_since_surgery_type_v2(4)] = 1	Range	text, Required
	126	sport_type_v2	Section Header: *Sporting History*<br>What sport did the participants engage in at the time of injury?<br>*If not detailed state N/A*	text, Required
	127	sport_level_v2	What was the competition level at the time of injury?<br>*If not detailed state N/A*	text, Required

For peer review only - http://bmjopen.bmj.com/site/about/guidelines.xhtml

	128	was_there_intervention_v2	Section Header: *Intervention Details*<br>Was there an intervention?	yesno<br>1 Yes<br>0 No
	129	intervention_type_v2<br>Show the field ONLY if:<br>[was_there_intervention_v2] = 1	Intervention type<br>*Please detail the type of intervention*	notes, Required
	130	intervention_setting_v2<br>Show the field ONLY if:<br>[was_there_intervention_v2] = 1	Intervention setting<br>*E.g., home, hospital, physio clinic*	text, Required
	131	duration_of_intervention_v2<br>Show the field ONLY if:<br>[was_there_intervention_v2] = 1	Duration of intervention<br>*Months*	text (number), Required
	132	adherence_v2<br>Show the field ONLY if:<br>[was_there_intervention_v2] = 1	Adherence rate	text (number), Required
	133	drop_out_v2<br>Show the field ONLY if:<br>[was_there_intervention_v2] = 1	Drop out rate	text (number), Required
	134	independent_variable_v2	Independent Variables	descriptive
	135	what_did_the_study_measure_v2	What was the independent variable measuring?<br>*Select all relevant answers*	checkbox, Required<br>1 what_did_the_study_measure_v2___1 Physical activity<br>2 what_did_the_study_measure_v2___2 Systemic inflammation<br>3 what_did_the_study_measure_v2___3 Knee joint loading<br>4 what_did_the_study_measure_v2___4 Adiposity<br>5 what_did_the_study_measure_v2___5 Muscle size<br>6 what_did_the_study_measure_v2___6 Muscle strength<br>7 what_did_the_study_measure_v2___7 Intramuscular fat<br>8 what_did_the_study_measure_v2___8 Bone mineral density<br>9 what_did_the_study_measure_v2___9 Unclear (need to contact authors)
	136	physical_activity_tool_v2<br>Show the field ONLY if:<br>[what_did_the_study_measure_v2(1)] = 1	Please select the tool used to measure physical activity<br>*Select all relevant answers*	checkbox, Required<br>1 physical_activity_tool_v2___1 Doubly labelled water<br>2 physical_activity_tool_v2___2 Accelerometers<br>3 physical_activity_tool_v2___3 Pedometers<br>4 physical_activity_tool_v2___4 Subjective questionnaires<br>5 physical_activity_tool_v2___5 Diaries<br>6 physical_activity_tool_v2___6 Other (please state below)<br>7 physical_activity_tool_v2___7 Unclear (need to contact authors)
	137	subjective_pa_tool_type_v2<br>Show the field ONLY if:<br>[physical_activity_tool_v2(4)] = '1'	What was the name of the subjective assessment tool?<br>*Please use full name, not abbreviation. E.g., International Physical Activity Questionnaire*	text, Required

	138	inflammation_tool_v2<br>Show the field ONLY if:<br>[what_did_the_study_measure_v2(2)] = '1'	Which cytokine/protein(s) were used to measure systemic inflammation?<br>*Select all relevant answers*	checkbox, Required<br>1 inflammation_tool_v2___1 Interleukin 6 (IL-6)<br>2 inflammation_tool_v2___2 Interleukin 1Beta (IL-1β)<br>3 inflammation_tool_v2___3 Tumor Necrosis Factor Alpha (TNF-α)<br>4 inflammation_tool_v2___4 C-Reactive Protein (CRP)<br>5 inflammation_tool_v2___5 Other (please state below)<br>6 inflammation_tool_v2___6 Unclear (need to contact authors)
	139	knee_load_tool_v2<br>Show the field ONLY if:<br>[what_did_the_study_measure_v2(3)] = '1'	What tool(s) used to measure knee joint loading?<br>*Select all relevant answers*	checkbox, Required<br>1 knee_load_tool_v2___1 Ground reaction force<br>2 knee_load_tool_v2___2 Moments<br>3 knee_load_tool_v2___3 Inverse dynamics<br>4 knee_load_tool_v2___4 Forward dynamics<br>5 knee_load_tool_v2___5 Other (please state below)<br>6 knee_load_tool_v2___6 Unclear (need to contact authors)
	140	adiposity_tool_v2<br>Show the field ONLY if:<br>[what_did_the_study_measure_v2(4)] = '1'	What was the tool to measure adiposity?	checkbox, Required<br>1 adiposity_tool_v2___1 Body Mass Index (BMI)<br>2 adiposity_tool_v2___2 Air displacement plethysmograph (ADP)<br>3 adiposity_tool_v2___3 Bioelectrical impedance (BI)<br>4 adiposity_tool_v2___4 Dual energy x-ray absorptiometry (DEXA or DXA)<br>5 adiposity_tool_v2___5 Computerised tomography (CT)<br>6 adiposity_tool_v2___6 Peripheral computerised tomography (pQCT)<br>7 adiposity_tool_v2___7 Other (please state below)<br>8 adiposity_tool_v2___8 Unclear (need to contact authors)
	141	muslce_tool_v2	What was the tool used to measure muscle size?	checkbox<br>1 muslce_tool_v2___1 Ultrasound (US)<br>2 muslce_tool_v2___2 Dual energy x-ray absorptiometry (DEXA or DXA)<br>3 muslce_tool_v2___3 Peripheral quantitative computerised tomography (pQCT)<br>4 muslce_tool_v2___4 Computerised tomography (CT)<br>5 muslce_tool_v2___5 Magnetic resonance imaging (MRI)<br>6 muslce_tool_v2___6 Other (please state below)<br>7 muslce_tool_v2___7 Unclear (need to contact authors)
	142	muscle_strength_tool_v2<br>Show the field ONLY if:<br>[what_did_the_study_measure_v2(5)] = '1'	What was the tool to measure muscle strength?	checkbox, Required<br>1 muscle_strength_tool_v2___1 Isokinetic dynamometry<br>2 muscle_strength_tool_v2___2 Hand-held dynamometer<br>3 muscle_strength_tool_v2___3 Other (please state below)<br>4 muscle_strength_tool_v2___4 Unclear (need to contact authors)

For peer review only - http://bmjopen.bmj.com/site/about/guidelines.xhtml

	143	intra_muscular_fat_tool_v2<br>Show the field ONLY if: [what_did_the_study_measure_v2(6)] = '1'	What was the tool to measure intra-muscular fat?	checkbox, Required<br>1 intra_muscular_fat_tool_v2___1 Ultrasound (US)<br>2 intra_muscular_fat_tool_v2___2 Computerised tomography (CT)<br>3 intra_muscular_fat_tool_v2___3 Peripheral quantitative computerised tomography (pQCT)<br>4 intra_muscular_fat_tool_v2___4 Magnetic resonance imaging (MRI)<br>5 intra_muscular_fat_tool_v2___5 Other (please state below)<br>6 intra_muscular_fat_tool_v2___6 Unclear (need to contact authors)
	144	bone_mineral_density_tool_v2<br>Show the field ONLY if: [what_did_the_study_measure_v2(7)] = '1'	What was the tool to measure bone mineral density?	checkbox, Required<br>1 bone_mineral_density_tool_v2___1 Dual energy x-ray absorptiometry (DEXA or DXA)<br>2 bone_mineral_density_tool_v2___2 Computerised tomography (CT)<br>3 bone_mineral_density_tool_v2___3 Peripheral quantitative computerised tomography (pQCT)<br>4 bone_mineral_density_tool_v2___4 Other (please state below)<br>5 bone_mineral_density_tool_v2___5 Unclear (need to contact authors)
	145	ptoa_disease_head_v2	Section Header: *Outcome Variables*<br>PTOA Disease	descriptive
	146	ptoa_disease_v2	Does the study measure PTOA disease?	yesno<br>1 Yes<br>0 No
	147	radiography_diagnostic_v2	Radiography diagnostic system	checkbox, Required<br>1 radiography_diagnostic_v2___1 Kellgren and Lawrence (K/L) system<br>2 radiography_diagnostic_v2___2 Osteoarthritis Research Society International (OARSI) Atlas criteria<br>3 radiography_diagnostic_v2___3 Unclear (need to contact authors)
	148	mri_diagnostic_v2	Magnetic resonance imaging diagnostic system	checkbox, Required<br>1 mri_diagnostic_v2___1 MRI Osteoarthritis Knee Score (MOAKS)<br>2 mri_diagnostic_v2___2 Whole Organ Magnetic Resonance Imaging Score (WORMS)<br>3 mri_diagnostic_v2___3 Boston Leeds OA Knee Score (BLOKS)<br>4 mri_diagnostic_v2___4 Unclear (need to contact authors)

	149	indicators_of_disease_v2	Indicators of structural deterioration preceding or exacerbating PTOA disease	checkbox, Required<br>1 indicators_of_disease_v2___1 MRI quantified cartilage thickness and volume<br>2 indicators_of_disease_v2___2 T1rho mapping<br>3 indicators_of_disease_v2___3 T2 mapping<br>4 indicators_of_disease_v2___4 Delayed contrast-enhanced MRI of cartilage (dGEMRIC)<br>5 indicators_of_disease_v2___5 Unclear (need to contact authors)
	150	systemic_biomarkers_v2	Systemic biomarkers indicating structural deterioration of knee PTOA disease	checkbox<br>1 systemic_biomarkers_v2___1 Matrix Metalloprotease 3 (MMP-3)<br>2 systemic_biomarkers_v2___2 Matrix Metalloprotease 13 (MMP-13)<br>3 systemic_biomarkers_v2___3 N-Propeptide of Collagen IIA (PIIANP)<br>4 systemic_biomarkers_v2___4 C-Telopeptide of Type II Collagen (CTX)<br>5 systemic_biomarkers_v2___5 C-Propeptide of Type II Collagen (CP-II)<br>6 systemic_biomarkers_v2___6 CTX:CP-II<br>7 systemic_biomarkers_v2___7 Collagen Type II Cleavage Product (C2C)<br>8 systemic_biomarkers_v2___8 Col2-3/4 C-terminal cleavage product of types I and II collagen (C1,2C)<br>9 systemic_biomarkers_v2___9 Hyaluronic Acid (HA)<br>10 systemic_biomarkers_v2___10 Chondroitin Sulphate 846 Epitope (CS846)<br>11 systemic_biomarkers_v2___11 Aggrecan164 or Aggrecan Fragments (ARGS)<br>12 systemic_biomarkers_v2___12 Cartilage Oligomeric Matrix Protein (COMP)<br>13 systemic_biomarkers_v2___13 Unclear (need to contact authors)
	151	outcome	Section Header: *Results*<br>Outcome 1<br>*Primary Outcome*	descriptive
	152	outcome_name	Please state what the independent variable is (e.g., physical activity), how it is measured (e.g., accelerometery), and the make/model of the measurement tool.	notes
	153	iv_name	Please state what the outcome variable is (e.g., c-telopeptide of type II collagen), how it is measured (e.g., blood serum), and the make/model of the measurement tool.	notes
	154	sample_size	Sample size for the test	text

	155	stat_test_1	What was the type of statistical test used?	radio<br>1 Difference test (t-test, ANOVA)<br>2 Correlation<br>3 Regression<br>4 Odds Ratio<br>5 Risk Ratio
	156	outcome_mean	Injured mean	text
	157	outcome_stdev	Injured standard deviation	text
	158	control_mean	Control mean	text
	159	cntrl_outcome_stdev	Control standard deviation	text
	160	p_correlation_coefficient	Pearson correlation coefficient	text
	161	s_correlation_coefficient	Spearman's correlation coefficient	text
	162	odds_ratio	Odds ratio	text
	163	risk_ratio	Risk ratio	text
	164	outcome_2	Outcome 2<br>*Primary Outcome*	descriptive
	165	outcome_name_2	Please state what the independent variable is (e.g., physical activity), how it is measured (e.g., accelerometery), and the make/model of the measurement tool.	notes
	166	iv_name_2	Please state what the outcome variable is (e.g., c-telopeptide of type II collagen), how it is measured (e.g., blood serum), and the make/model of the measurement tool.	notes
	167	sample_size_2	Sample size for the test	text
	168	stat_test_2	What was the type of statistical test used?	radio<br>1 Difference test (t-test, ANOVA)<br>2 Correlation<br>3 Regression<br>4 Odds Ratio<br>5 Risk Ratio
	169	outcome_mean_2	Injured mean	text
	170	outcome_stdev_2	Injured standard deviation	text
	171	control_mean_2	Control mean	text
	172	cntrl_outcome_stdev_2	Control standard deviation	text
	173	p_correlation_coefficient_2	Pearson correlation coefficient	text
	174	s_correlation_coefficient_2	Spearman's correlation coefficient	text
	175	odds_ratio_2	Odds ratio	text
	176	risk_ratio_2	Risk ratio	text
	177	outcome_3	Outcome 3<br>*Primary Outcome*	descriptive
	178	outcome_name_3	Please state what the independent variable is (e.g., physical activity), how it is measured (e.g., accelerometery), and the make/model of the measurement tool.	notes
	179	iv_name_3	Please state what the outcome variable is (e.g., c-telopeptide of type II collagen), how it is measured (e.g., blood serum), and the make/model of the measurement tool.	notes
	180	sample_size_3	Sample size for the test	text
	181	stat_test_3	What was the type of statistical test used?	radio<br>1 Difference test (t-test, ANOVA)<br>2 Correlation<br>3 Regression<br>4 Odds Ratio<br>5 Risk Ratio
	182	outcome_mean_3	Injured mean	text
	183	outcome_stdev_3	Injured standard deviation	text
	184	control_mean_3	Control mean	text
	185	cntrl_outcome_stdev_3	Control standard deviation	text

For peer review only - http://bmjopen.bmj.com/site/about/guidelines.xhtml

	186	p_correlation_coefficient_3	Pearson correlation coefficient	text
	187	s_correlation_coefficient_3	Spearman's correlation coefficient	text
	188	odds_ratio_3	Odds ratio	text
	189	risk_ratio_3	Risk ratio	text
	190	conclusion01	Section Header: *Conclusion*<br>Please state the author's conclusions	notes
	191	other_comments	Please state any comments/conclusions you have	notes
	192	data_extraction_2_complete	Section Header: *Form Status*<br>Complete?	dropdown<br>0 Incomplete<br>1 Unverified<br>2 Complete

For peer review only

Supplementary Material 8. Strength of the treatment response or relationship between variables

Interpreting effect size values[1,2,3,4]

Effect Size Measure	Effect Size			
	**Small**	**Medium**	**Large**	**Very Large**
Odds Ratio	1.5	2.5	4	10
Cohen's *d*	0.2	0.5	0.8	1.3
r	0.1	0.3	0.5	0.7
Cohen's *f*	0.1	0.25	0.4	-
Eta-squared	0.01	0.06	0.14	-

1. Maher JM, Markey JC, Ebert-May D. The Other Half of the Story: Effect Size Analysis in Quantitative Research. *CBE Life Sci Educ* 2013;12(3):345-51. doi: 10.1187/cbe.13-04-0082

2. Rosenthal JA. Qualitative Descriptors of Strength of Association and Effect Size. J Soc Serv Res 1996;21(4):37-59. doi: 10.1300/j079v21n04_02

3. Cohen J. A power primer. Psychological bulletin 1992;112(1):155.

4. Cohen J. Statistical Power Analysis for the Behavioral Sciences. 2nd Edition ed. New York: Routledge 1988.

For peer review only

Supplementary Material 9. Adapted Grading of Recommendations, Assessment, Development and Evaluations (GRADE) Guidelines for Scoping Review

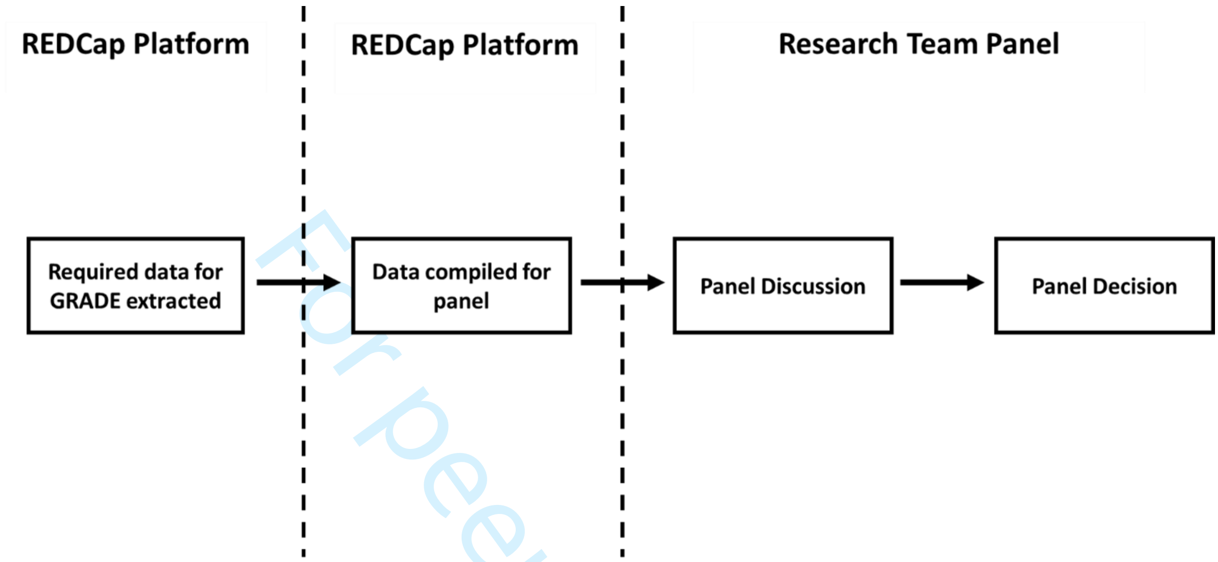

For peer review only

Draft - Adapted Grading of Recommendations, Assessment, Development and Evaluations (GRADE) Guidelines for Scoping Review

Overview:

Grading of Recommendations, Assessment, Development and Evaluations (GRADE)[1-3] is structured as:

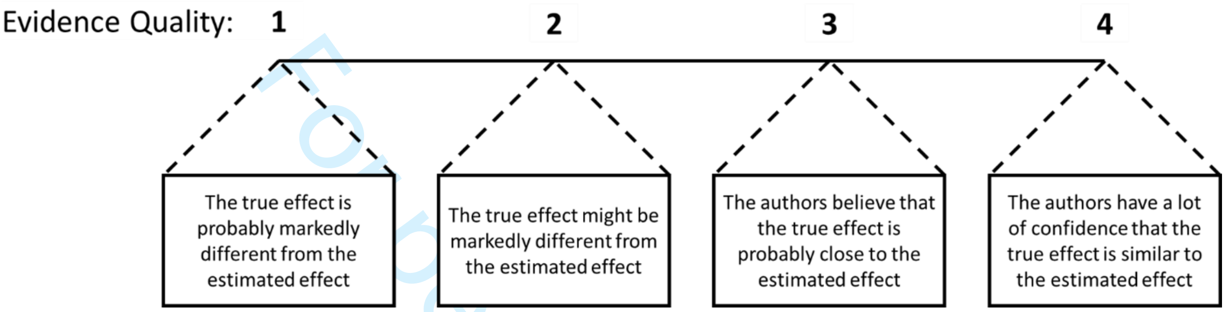

In GRADE, randomised control trials (RCTs) are the highest quality of research evidence (4), whilst observational studies (e.g., analytical cross-sectional studies) are the lowest form of evidence (1).[1-3] From there, individual studies will either be downgraded or upgraded for quality of evidence. For example, if an RCT is not downgraded it will provide the highest quality of evidence (4).[1-3]

In this review, the GRADE criteria will be applied to the research question '***what is the role of physical activity in the trajectory from intra-articular knee injury to post-traumatic osteoarthritis in young men and women?***' and will be considered through each of the following themes:

- Physical activity
- Systemic inflammation
- Adiposity
- Knee joint loading
- Lower body muscle strength
- Muscle size
- Intra-muscular fat
- Bone mineral density

For peer review only - http://bmjopen.bmj.com/site/about/guidelines.xhtml

Downgrade quality of evidence

1. Risk of Bias (RoB)

According to Ma *et al.*,[4] "Quality includes internal and external validity, while methodological quality usually refers to internal validity. Internal validity is also recommended as "risk of bias (RoB)" by the Cochrane Collaboration."

In this review, we will use the Joanna Briggs Institute (JBI) Critical Appraisal Checklists (Supplementary Material 6) to assess the RoB of each individual study. The Critical Appraisal Checklists apply to:

- Analytical Cross Sectional Studies[5]
- Case Control Studies[5]
- Cohort Studies[5]
- Studies Reporting Prevalence Data[6]
- Quasi-Experimental Studies[7]
- Randomised Control Trials[7]
- Systematic Review and Research Synthesis[8]

For the purpose of this review, we will classify the scores generated from JBI Critical Appraisal Checklists as either:

- Very low quality (0 – 25.0%)
- Low quality (>25.0 – 50.0%)
- Moderate quality (>50.0 – 75.0%)
- High quality (75.0 – 100%)

This scoring system will be applied to studies on a case-by-case basis.

Following application of the scoring system to all studies, we will adopt GRADE guidelines by downgrading studies if, collectively for the outcome, there is either a serious risk of bias or a very serious risk of bias. The scoring criteria for this review will be as follows:

- (**0**) No serious risk of bias overall = all studies scored moderate to high quality
- (**-1**) Serious risk of bias overall = more than 25% of studies considered low or very low quality
- (**-2**) Very serious risk of bias overall = More than 50% of studies considered low or very low quality

2. Inconsistency

Study results will be considered consistent when directions (e.g., positive or negative association), are considered similar enough to draw uniform conclusions. In this review, consistency in direction will be defined as >75% of the included studies for each theme demonstrating the same positive effect only or negative effect only.

For peer review only

- (**-1**) Serious risk of inconsistency when ≤75% of the included studies have the same direction of effect.
- (**-2**) Very serious risk of inconsistency when there is only one or no studies.

3. Indirectness

GRADE distinguishes several types of indirectness[9]. In this review, indirectness may be due to any of the factors detailed below:

Independent variable (*applicability*)[9]

- (**-1**) Serious risk of indirectness when the independent variable typically used by studies is deemed less valid than the gold standard (e.g., adiposity inferred from body mass index rather than dual-energy x-ray absorptiometry)
- (**-1**) Serious risk of indirectness an intervention cannot be completed in multiple settings (e.g., hospital and home based)

If no studies test a relevant intervention to the research question, or there is no research investigating any of the identified themes, we will conclude that the effects are uncertain.

Differences in outcome measures (*surrogate outcomes*)[9]

- (**-1**) Serious risk of indirectness when research typically uses of a surrogate measure which is associated with knee PTOA (e.g., systemic concentrations of type-II collagen biomarker), rather than directly diagnosing PTOA
- **(-1)** Serious risk of indirectness when studies do not provide information on full time course of PTOA development (e.g., only short-term effects of a physical activity intervention on a surrogate provide, and no studies investigating the long-term impact of PTOA diagnosis)

Indirect comparison[9]

- (**-1**) Serious risk of indirectness by lack of comparisons between physical activity types or exercise modalities
- (**-1**) Serious risk of indirectness by lack of comparison to alternative approaches (e.g., pharmaceutical driven pain relief)

4. Imprecision

As the data will not be combined in a meta-analysis, the following qualitative assessment will be applied to determine imprecision[10]:

- (**-1**) Serious risk of imprecision when the lower threshold of 95% CI reduces the effect to negligible

For peer review only

- (**-1**) Serious risk of imprecision when effect of estimate presented in only one or two studies or estimate comes from one or two events

- (**-2**) Very serious risk of imprecision if the lower threshold of 95% CI is consistent with conflicting conclusions

- (**-2**) Very serious risk of imprecision if CIs were not provided in any of the studies

5. Publication Bias

Assessment of publication bias using statistical methods or funnel plots will not be possible due to heterogeneity of the studies in this review. Publication bias will be screened for qualitatively as detailed in Chapter 7 of the Cochrane Handbook.

For peer review only

Upgrade quality of evidence

1. Large Magnitude of Effect

In this review, when a very large magnitude of effect is observed (assessed using Supplementary Material 8), there is greater certainty that there is at least a small effect.[11]

- (**+1**) when the effect size is large
- (**+2**) when the effect size is very large

The time-course of outcomes will also be considered in this review[12] when upgrading due to large magnitude of effect and devised into:

- (**+1**) Short-term (e.g., response to single exercise session/alteration of joint loading)
  - Systemic biomarkers in synovial fluid/serum/plasma/urine
  - MRI or US indicates deterioration
  - Symptoms or function

- (**+1**) Medium-term (e.g., response to 12-week activity intervention)
  - Systemic biomarkers in synovial fluid/serum/plasma/urine
  - MRI or US indicates deterioration
  - Symptoms or function

- (**+2**) Long-term (e.g., Body Mass Index at one-year post-injury and PTOA development in the years following)
  - X-Ray or MRI PTOA Diagnosis

This is because biomarkers concentrations changes can occur rapidly due to mechanical compression,[13-16] or an exercise-based intervention.[17] Furthermore, the basal protein synthesis rates in various musculoskeletal tissues (including cartilage, meniscus, bone) are within the same range of skeletal muscle protein synthesis rates[18] which may indicate pathology much faster than it takes for PTOA to fully develop.

Lastly, if there are major concerns regarding risk of bias, consistency, precision, or publication bias, in addition to being downgraded, the quality of evidence will not be upgraded via magnitude of effect.[12]

2. Dose-Response Gradient

This increases our confidence in the findings of observational studies and thus enhance the assigned quality of evidence.[12] This is best represented by RCTs and quasi-experimental trials.

- (**+1**) when there is evidence of a dose (e.g., physical activity-based intervention) and response (e.g., increased concentrations of aggrecan

For peer review only

synthesis, lower concentrations of type-II collagen breakdown, slower MRI defined joint tissue degeneration)

3. **All residual confounding would decrease magnitude of effect (in situations with an effect)**

All plausible residual confounders or biases would reduce a demonstrated effect, or suggest a spurious effect when results show no effect.[12]

REFERENCES

1. Guyatt GH, Oxman AD, Kunz R, et al. What is “quality of evidence” and why is it important to clinicians? *BMJ* 2008;336(7651):995-98. doi: 10.1136/bmj.39490.551019.be
2. Guyatt GH, Oxman AD, Vist GE, et al. GRADE: an emerging consensus on rating quality of evidence and strength of recommendations. *BMJ* 2008;336(7650):924-26. doi: 10.1136/bmj.39489.470347.ad
3. Guyatt G, Oxman AD, Akl EA, et al. GRADE guidelines: 1. Introduction-GRADE evidence profiles and summary of findings tables. *J Clin Epidemiol* 2011;64(4):383-94. doi: 10.1016/j.jclinepi.2010.04.026 [published Online First: 20101231]
4. Ma L-L, Wang Y-Y, Yang Z-H, et al. Methodological quality (risk of bias) assessment tools for primary and secondary medical studies: what are they and which is better? *Mil Med Res* 2020;7(1) doi: 10.1186/s40779-020-00238-8
5. Moola S, Munn Z, Tufanaru C, et al. Chapter 7: Systematic reviews of etiology and risk. In: Aromataris E, Munn Z, eds. JBI Manual for Evidence Synthesis: JBI, 2020.
6. Munn Z, Moola S, Lisy K, et al. Methodological guidance for systematic reviews of observational epidemiological studies reporting prevalence and cumulative incidence data. *Int J Evid Based Healthc* 2015;13(3):147-53. doi: 10.1097/XEB.0000000000000054
7. Tufanaru C, Munn Z, Aromataris E, et al. Chapter 3: Systematic reviews of effectiveness. In: Aromataris E, Munn Z, eds. JBI Manual for Evidence Synthesis: JBI, 2020.
8. Aromataris E, Fernandez R, Godfrey CM, et al. Summarizing systematic reviews: methodological development, conduct and reporting of an umbrella review approach. *Int J Evid Based Healthc* 2015;13(3):132-40. doi: 10.1097/XEB.0000000000000055
9. Guyatt GH, Oxman AD, Kunz R, et al. GRADE guidelines: 8. Rating the quality of evidence—indirectness. *J Clin Epidemiol* 2011;64(12):1303-10. doi: 10.1016/j.jclinepi.2011.04.014
10. Guyatt GH, Oxman AD, Kunz R, et al. GRADE guidelines 6. Rating the quality of evidence—imprecision. *J Clin Epidemiol* 2011;64(12):1283-93. doi: 10.1016/j.jclinepi.2011.01.012

For peer review only

11. Reed S, Guyatt G. What is GRADE? BMJ: BMJ; 2022 [Available from: https://bestpractice.bmj.com/info/toolkit/learn-ebm/what-is-grade/ accessed 20/06/2022 2022.
12. Guyatt GH, Oxman AD, Sultan S, et al. GRADE guidelines: 9. Rating up the quality of evidence. *J Clin Epidemiol* 2011;64(12):1311-16. doi: 10.1016/j.jclinepi.2011.06.004
13. Mundermann A, Dyrby CO, Andriacchi TP, et al. Serum concentration of cartilage oligomeric matrix protein (COMP) is sensitive to physiological cyclic loading in healthy 9adults. *Osteoarthritis Cartilage* 2005;13(1):34-38. doi: 10.1016/j.joca.2004.09.007 [published Online First: 2005/01/11]
14. Mündermann A, King KB, Smith RL, et al. Change in serum COMP concentration due to ambulatory load is not related to knee OA status. *J Orthop Res* 2009;27(11):1408-13. doi: 10.1002/jor.20908 [published Online First: 2009/05/08]
15. Mundermann A, Klenk C, Billich C, et al. Changes in Cartilage Biomarker Levels During a Transcontinental Multistage Footrace Over 4486 km. *Am J Sports Med* 2017;45(11):2630-36. doi: 10.1177/0363546517712945 [published Online First: 2017/06/27]
16. Roberts HM, Law RJ, Thom JM. The time course and mechanisms of change in biomarkers of joint metabolism in response to acute exercise and chronic training in physiologic and pathological conditions. *Eur J Appl Physiol* 2019;119(11-12):2401-20. doi: 10.1007/s00421-019-04232-4 [published Online First: 2019/10/28]
17. Azukizawa M, Ito H, Hamamoto Y, et al. The effects of well-rounded exercise program on systemic biomarkers related to cartilage metabolism. *Cartilage* 2019;10(4):451-58. doi: 10.1177/1947603518767998 [published Online First: 2018/04/13]
18. Smeets JSJ, Horstman AMH, Vles GF, et al. Protein synthesis rates of muscle, tendon, ligament, cartilage, and bone tissue in vivo in humans. *PLoS One* 2019;14(11):e0224745. doi: 10.1371/journal.pone.0224745 [published Online First: 2019/11/08]

For peer review only

BMJ Open

Understanding the Role of Physical Activity on the Pathway from Intra-Articular Knee Injury to Post-Traumatic Osteoarthritis Disease in Young People: A Scoping Review Protocol

Journal:	*BMJ Open*
Manuscript ID	bmjopen-2022-067147.R2
Article Type:	Protocol
Date Submitted by the Author:	14-Feb-2023

Complete List of Authors:	Morgan, Karl; University of Bath, Department for Health; Centre for Health and Injury and Illness Prevention in Sport (CHI2PS), University of Bath<br>Cowburn, James; University of Bath, Department for Health<br>Farrow, Matthew; University of Bath, Department for Health; Centre for Nutrition and Exercise Metabolism (CNEM), University of Bath<br>Carter, Josh; University of Bath, Department for Health<br>Cazzola, Dario; University of Bath, Department for Health; Centre for the Analysis of Motion, Entertainment Research and Applications (CAMERA), University of Bath<br>Walhin, Jean-Philippe; University of Bath, Department for Health; Centre for Nutrition and Exercise Metabolism (CNEM), University of Bath<br>McKay, Carly; University of Bath, Department for Health; Centre for Health and Injury and Illness Prevention in Sport (CHI2PS), University of Bath

**Primary Subject Heading**:	Sports and exercise medicine
Secondary Subject Heading:	Rheumatology
Keywords:	Knee < ORTHOPAEDIC & TRAUMA SURGERY, Orthopaedic sports trauma < ORTHOPAEDIC & TRAUMA SURGERY, SPORTS MEDICINE, RHEUMATOLOGY, Musculoskeletal disorders < ORTHOPAEDIC & TRAUMA SURGERY

SCHOLARONE™
Manuscripts

Title Page

Title: Understanding the Role of Physical Activity on the Pathway from Intra-Articular Knee Injury to Post-Traumatic Osteoarthritis Disease in Young People: A Scoping Review Protocol

Authors: Karl Morgan*[1 2 3 5], Dr James Cowburn[1 2], Dr Matthew Farrow[1 3 5], Josh Carter[1], Dr Dario Cazzola[1 2 4], Dr Jean-Philippe Walhin[1 3], Dr Carly McKay[1 2 5]

Department for Health, University of Bath, Bath, UK[1]

Centre for Health and Injury and Illness Prevention in Sport (CHI²PS), Department for Health, University of Bath, Bath, UK[2]

Centre for Nutrition and Exercise Metabolism (CNEM), Department for Health, University of Bath, Bath, UK[3]

Centre for the Analysis of Motion, Entertainment Research and Applications (CAMERA), University of Bath, Bath, UK[4]

Centre for Sport, Exercise and Osteoarthritis Versus Arthritis, University of Bath, Bath, UK[5]

***Corresponding Author:** kdm40@bath.ac.uk, Department for Health, Claverton Down, Bath, UK, BA2 7AY

Word Count: 2, 522

For peer review only - http://bmjopen.bmj.com/site/about/guidelines.xhtml

For peer review only

ABSTRACT

Introduction

The prevalence of intra-articular knee injuries and reparative surgeries is increasing in many countries. Alarmingly, there is a risk of developing post-traumatic osteoarthritis (PTOA) after sustaining a serious intra-articular knee injury. Although physical inactivity is suggested as a risk factor contributing to the high prevalence of the condition, there is a paucity of research characterising the association between physical activity and joint health. Consequently, the primary aim of this review will be to identify and present available empirical evidence regarding the association between physical activity and joint degeneration after intra-articular knee injury and summarise the evidence using an adapted Grading of Recommendations, Assessment, Development and Evaluations. The secondary aim will be to identify potential mechanistic pathways through which physical activity could influence PTOA pathogenesis. The tertiary aim will be to highlight gaps in current understanding of the association between PA and joint degeneration following joint injury.

Methods

A scoping review will be conducted using the Preferred Reporting Items for Systematic reviews and Meta-Analyses extension for Scoping Reviews checklist and best-practice recommendations. The review will be guided by the following research question: what is the role of physical activity in the trajectory from intra-articular knee injury to post-traumatic osteoarthritis in young men and women? We will identify primary research studies and grey literature by searching the electronic databases Scopus, Embase: Elsevier, PubMed, Web of Science: all databases, and Google Scholar. Reviewing pairs will screen abstracts, full texts, and will extract data. Data will be presented descriptively using charts, graphs, plots, and tables.

Ethics and dissemination

For peer review only - http://bmjopen.bmj.com/site/about/guidelines.xhtml

This research does not require ethical approval due to the data being published and publicly available. This review will be submitted for publication in a peer-reviewed sports medicine journal irrespective of discoveries and disseminated through scientific conference presentations and social media.

Registration

This scoping review is registered on the Open Science Framework (https://osf.io/84pnh/)

Strengths and limitations of this study

- A scoping review is the most appropriate approach to identify and describe current evidence on the complex relationship between PA and post-traumatic osteoarthritis due to the multi-faceted aetiology underpinning the condition.
- This novel research lays an important foundation for further empirical research by mapping current evidence in the literature, examining potential mechanistic pathways using a multi-disciplinary approach, and identifying gaps in current knowledge.
- Due to the broad nature of a scoping review, this approach may limit the ability to comprehensively elucidate pathological mechanisms.

For peer review only

INTRODUCTION

Knee injuries, resulting from both contact and non-contact mechanisms,[1-9] are a common orthopaedic complaint in young, sporting populations worldwide[10] and often involve damage to multiple tissues inside the joint.[11-14] Reconstruction surgery is commonly used to treat the injured tissue,[9 11 13 15 16] with hundreds of thousands of knee injuries and reparative reconstruction procedures performed worldwide every year. [13 17-19] Current data suggests rates of intra-articular injury and tissue repair are rising in several countries,[17-19] with knee injuries predominantly affecting people under 40 years of age, [13 17-19] which is alarming considering injury greatly increases the risk of developing knee osteoarthritis (OA).[20] Knee OA is a highly prevalent disorder[21] which is becoming more common,[21-23] and is composed of both a disease and an illness component.[24] OA disease is associated with illness,[25] and the threshold for disease inducing illness is low.[24] However, the presence of illness does not always indicate structural deterioration and disease does not always induce symptomology,[26 27] with history of knee injury alone enough to induce illness.[27]

Knee OA affecting people with a history of intra-articular injury is defined as post-traumatic osteoarthritis (PTOA).[28-31] There is 10-fold increased odds of experiencing PTOA disease between 3-10 years following a knee injury.[27] Further, the prevalence of the disease 11-17 years following knee trauma (48-52%)[29 32] is concerning when considering average age at diagnosis for idiopathic knee OA is 55 years,[33] which indicates a typical case of PTOA would increase disease burden duration. After disease diagnosis, the joint may deteriorate to end stage OA, resulting in increased odds for total knee replacement (TKR), which occurs at a noticeably younger age in populations with an injury history than those without.[34] Knee OA is the primary cause for TKR[35] and the number of TKRs in people aged under 55 is increasing.[36] As TKRs typically last 25 years,[37] these younger patients experiencing PTOA may sustain subsequent prosthesis failure within their lifetime.[38]

In terms of PTOA illness, after intra-articular knee injury or anterior cruciate ligament reconstruction there is a high prevalence of knee symptomology (e.g., pain, stiffness, swelling, crepitus) and loss of function.[39-43] Knee pain is described as the cardinal symptom of OA,[44] and often drives people to seek medical care.[45-47] When applying the pain-avoidance model,[48] developed from idiopathic

For peer review only - http://bmjopen.bmj.com/site/about/guidelines.xhtml

knee OA populations, to PTOA, experiencing disease and illness due to PTOA may exacerbate loss of function across the lifespan, thereby accelerating the decline below the threshold of independence (Figure 1).[46 49] Consequently, understanding joint deterioration and characterising risk factors for PTOA in knee injury populations, with a view to prevent or slow condition progression, is paramount.

FIGURE 1. HYPOTHETICAL TRAJECOTRY OF INJURY TO LIFELONG DISEASE BURDEN

Physical inactivity is postulated as one of several risk factors that may compound joint degeneration following intra-articular knee injury.[50 51] Subjective and objective measurements of physical activity (PA) levels indicate reduced participation following knee injury[52-60] and many individuals do not return to the same level of sports participation or return to sport at all after anterior cruciate ligament reconstruction.[61-63] Therapeutic exercise is a core treatment for knee OA, and is considered beneficial to joint health,[64] whilst physical inactivity has been touted as a key contributor to joint deterioration in idiopathic OA.[22 50] However, a recent narrative review suggested there is little minimal robust scientific evidence linking PA level and joint health following injury.[51] Furthermore, although PA often involves knee joint loading (e.g., walking or running), there is limited research investigating the association between cumulative joint loading (dictated by PA) and joint health after knee joint trauma. Lastly, there are several physiological maladaptations that can occur after a traumatic knee injury, such as; increased adiposity,[41 54 59 65-67] knee extensor and flexor weakness,[68-70] muscle atrophy,[71 72] intra-muscular fat accumulation,[73 74] and bone mineral density reduction,[75] which could influence joint health. However, these associations with PTOA are yet to be fully understood. Considering PA is causally associated with systemic inflammation,[76-78] adiposity,[79] muscle size[80 81] and quality,[81-86] and bone mineral density[87 88] in uninjured populations, exploring the associations between these factors in injured populations may help to explain the complex relationship between PA and joint health following intra-articular knee injury.

For peer review only

Consequently, this protocol documents the procedure for conducting the review. The primary aim of the scoping review will be to identify and present available empirical evidence regarding the association between PA and joint degeneration after intra-articular knee injury and summarise the evidence using an adapted Grading of Recommendations, Assessment, Development, and Evaluations (GRADE).[89-91] The secondary aim will be to establish potential mechanistic pathways through which PA could influence PTOA pathogenesis. The tertiary aim will be to highlight gaps in current understanding of the association between PA and joint health following injury.

METHODS

This scoping review is an exploratory project which will systematically map the literature relevant to the research question, identifies key concepts, theories and sources of evidence, and gaps in the research.[92 93] This protocol has been prepared to ensure the transparency of the review process and will be used as a guide when conducting the review and reporting the outcomes to minimise the risk of reporting bias.[94 95] Further, this protocol has been developed in accordance with the adapted Preferred Reporting Items for Systematic Review and Meta-Analysis Protocol (PRISMA-P)[96 97] for scoping reviews[95] (Supplementary Material 1). This review is registered on Open Science Framework (https://osf.io/84pnh/) and the final output will be underpinned both by the Preferred Reporting Items for Systematic reviews and Meta-Analyses extension for Scoping Reviews (PRISMA-ScR)[98] and best practice recommendations.[99-101] Deviations from this protocol when conducting the review will be documented and published in supplementary material of the final output. This scoping review is currently in progress, with the first search completed in August 2022 and has a projected end date of May 2023.

Patient and Public Involvement

During protocol design and planning, there was no patient or public involvement.

For peer review only - http://bmjopen.bmj.com/site/about/guidelines.xhtml

Framework

This review will follow the Arksey and O'Malley framework and adhere to the Joanna Briggs Institute quality assessment recommendations (Figure 2).[99 102]

FIGURE 2. OUTLINE OF THE STUDY METHODOLOGY

Stage 1: Identifying the Research Question

The review will be guided by the following research question: What is the role of PA on the trajectory from intra-articular knee injury to post-traumatic osteoarthritis in young men and women? As PTOA is a joint condition with a 'disease' component and an 'illness' component,[24] we will only describe the association between PA, and the factors physical activity underpins, with PTOA disease in this review. Disease will be defined as: 'abnormalities of the structure and function of body organs and systems that can be specifically identified and described by reference to certain biological, chemical or other evidence'.[103] Additionally, we will only include studies working with young participants (average age of study population at study start or follow up measurement 18-40 years old) in this review. Within the research question, the following themes have been identified *a priori*: PA, systemic inflammation, knee joint load, adiposity, strength (lower body), intra-muscular adipose tissue (lower body), muscle size (lower body), and bone mineral content. For this review, we will use consensus definitions for intra-articular knee injury, PA, the themes underpinned by PA, and PTOA (Table 1). If there is no agreed or clear definition, then best practices used by previous literature or consensus among the research team will be adopted.

Table 1. Review Definitions

Term	Definition

For peer review only

**Post-traumatic osteoarthritis**	Joint disorder characterised by extracellular matrix degradation initiated by micro- and macro-injury resulting in the manifestation of molecular derangement (e.g., abnormal joint tissue metabolism) followed by anatomic, and/or physiologic derangements (e.g., cartilage degradation), that can culminate in illness.[24] In this review, we will focus on the disease component of the disorder.
**Intra-articular knee injury**	Previous research has identified cruciate ligament and meniscus injuries have the highest risk of developing into post-traumatic osteoarthritis.[20] Considering these are also the most common types of intra-articular knee injury, our review will focus on both ligament and meniscus injuries only.
**Physical activity**	Any bodily movement produced by skeletal muscles that results in energy expenditure.[104] Further, we will include the term 'exercise' within this definition of physical activity, as although they describe different concepts, they are often used as synonyms.[104] Exercise is defined as a subset of physical activity that is planned, structured, and repetitive and has as a final or an immediate objective the improvement or maintenance of physical fitness.[104]
**Systemic inflammation**	A consensus definition on the type, number, and value of inflammatory biomarkers for systemic inflammation diagnosis has not been devised. In this review, we will use concentrations of key circulating proteins and cytokines identified in previous research as being involved with inflammation processes and OA.[105-110]
**Knee joint load**	The research team defines knee joint load as quantification of the forces and/or moments applied to the knee joint specifically.
**Strength (lower body)**	Strength is the ability to produce a maximal voluntary muscular contraction against an external resistance.[111] We consider the following movements as relevant to this review:<br>- Hip extension/flexion/abduction/adduction<br>- Knee extension/flexion/abduction/adduction<br>- Ankle dorsi/plantar flexion, supination/pronation
**Adipose tissue**	Specialised loose connective tissue that is extensively laden with adipocytes.[112] Adipose tissue is arranged in discrete depots,[113] and we consider the following depots at any body location relevent to this review:<br>- subcutaneous<br>- intraperitoneal/viseral<br>- inter-muscular (considered to be similar to intraperitoneal/visceral[114]
**Intra-muscular adipose tissue (lower body)**	Intra-muscular adipose tissue is the visible fat found within a muscle.[114] We consider the intra-muscular adipose content in any of the following muscle groups/muscles relevant to this review:<br>- Gluteal<br>- Hamstring<br>o Bicep femoris<br>o Semitendinosus<br>o Semimembranosus<br>- Quadriceps<br>o Vastus Lateralis<br>o Rectus Femoris<br>o Vastus Medialis<br>o Vastus Intermedius<br>- Tibialis anterior<br>- Gastrocnemius<br>- Soleus

For peer review only

**Muscle size (lower body)**	Muscle size if the dimension of skeletal muscle, which can be devised into a multi-scale[115] consisting of;<br>- organ<br>- tissue<br>- cellular<br>- sub-cellular<br>- molecular<br><br>We consider the muscle size of muscle groups/muscles relevant to this review:<br>- Gluteal<br>- Hamstring<br>○ Bicep Femoris<br>○ Semitendinosus<br>○ Semimembranosus<br>- Quadriceps<br>○ Vastus Lateralis<br>○ Rectus Femoris<br>○ Vastus Medialis<br>○ Vastus Intermedius<br>- Tibialis anterior<br>- Gastrocnemius<br>- Soleus
**Bone mineral content**	The research team defines bone mineral content as the amount of minerals (mostly calcium and phosphorous) contained in bone.<br><br>Total bone mineral content of the body is considered relevant to this review. Additionally, the mineral content of the following bones is also considered relevant to this review:<br>- Femur<br>- Tibia<br>- Fibular

100

101 Stage 2: Identifying Relevant Studies

102 The search terms include population, independent variable, and outcome (Supplementary
103 Material 2). Using the Peer Review of Electronic Search Strategy (PRESS)[116] guidelines, a professional
104 health science librarian was consulted to develop and tailor search strategies for each database.
105 Electronic databases Scopus, Embase: Elsevier, PubMed, Web of Science: all databases, and Google
106 Scholar will be searched. Literature published between 1970 and 2022 will be included, covering the
107 period during which clinical knee injury diagnosis was formalised and surgical reparative techniques
108 were popularised.[117 118] Primary research studies and grey literature will be included.[93 119 120]

109

110 Stage 3: Study Selection

111 Inclusion and exclusion criteria are presented in Table 2.

For peer review only - http://bmjopen.bmj.com/site/about/guidelines.xhtml

Table 2. Review Inclusion and Exclusion Criteria

Inclusion	Exclusion
Includes participants with a history of experiencing intra-articular injury or reparative surgery for the injury.	Reporting of any other medical condition (disease, illness, or disorder):<br>- Musculoskeletal<br>- Immunological<br>- Cardiological<br>- Respiratory<br>- Neurological<br>- Metabolic
Measures any association between PA or factors determined by PA (systemic markers of inflammation, joint loading, adiposity, muscle strength, lower body muscle size, inter-muscular fat, bone density), and either post-traumatic osteoarthritis diagnosis or indicators of joint degeneration.	Evidence of intra-articular injury incidence to any of the following joints prior to or following intra-articular knee injury:<br>- Ankle<br>- Knee<br>- Hip<br>- Shoulder<br>- Elbow
18-40 years of age	Revision surgery if undergone initial reparative surgery
Primary research studies, grey literature	Animal models, *in vitro* studies, purely *in silico* studies with no human component (i.e., simulated data with no data capture element working with humans)
Human research	
Published in English	
Literature published between 1970 and 2022	

Outcome measures for PTOA disease must include medical imaging or molecular indicators of joint health (e.g., metabolic, proteomic, genomic, transcriptomic).[24] Imaging diagnosis of disease may include radiography[121 122] or magnetic resonance imaging (MRI).[123-125] Imaging indicators for disease processes include MRI[126-129] and ultrasound (US) techniques[130-132]. For molecular indicators of disease processes, OsteoArthritis Research Society International (OARSI) recommended biomarkers[133] will be included alongside other biomarkers of cartilage metabolism which have been deemed of interest in previous reviews due to their association with joint health (Supplementary Material 3).[134-137] This list of outcome measures is not exhaustive and will be added to if other relevant outcomes are identified during Stage 2 - Identifying Relevant Studies.

To establish the face and content validity of the inclusion and exclusion criteria, and the selected outcome measures, feedback was sought from a surgeon, an osteopath, a physiotherapist, a physiologist,

For peer review only - http://bmjopen.bmj.com/site/about/guidelines.xhtml

For peer review only

and a biomechanist independent from the research team, with experience ranging from clinical practice to sport research. These inclusion and exclusion criteria will be applied to title/abstract screening, which will be conducted using Covidence (Covidence Systematic Review Software, Veritas Health Innovation, Melbourne, Australia. Available at www.covidence.org). Prior to screening start, JC, JC, and MF will be familiarised and trained[138] by the lead author (KM) to conduct title/abstract screening using a screening tool hosted on Research Electronic Data Capture Platform (REDCap)[139] (Supplementary Material 4). Following the training session, inter-rater reliability of the inclusion and exclusion criteria for title/abstract screening will be assessed based on a random sample of 120 titles/abstracts. Percentage agreement and Cohen's Kappa (κ)[140] will be determined between all reviewers (KM - 100% of records, JC - 33%, JC - 33%, MF - 33%). The inclusion and exclusion criteria will be deemed reliable if the agreement is 'strong' (percentage agreement >63% and κ >0.60),[141] and title/abstract screening will only begin once the agreement level is strong. Following training and provided 'strong' agreement between reviewers is achieved, title/abstract screening will begin. During this process reviewing pairs (KM - 100% of records, JC - 33%, JC - 33%, MF - 33%) will independently screen all titles and abstracts to reduce quantity of errors.[142] If disagreements cannot be resolved within reviewing pairs, a consensus decision across the wider research team will be made following a discussion. Duplicate results returned during title/abstract screening will be removed using Covidence.

Following title and abstract screening, full text screening will be conducted by reviewing pairs (KM - 100% of records, JC - 33%, JC - 33%, MF - 33%).[138] Prior to full text screening, JC, JC and MF will be familiarised and trained[138] by the lead author (KM) to conduct full text screening using a screening tool hosted on Research Electronic Data Capture Platform (REDCap)[139] (Supplementary Material 5). Following the training session, inter-rater reliability of the inclusion and exclusion criteria for the full text screening will be assessed based on a random sample of 20 full texts. Once 'strong' agreement between reviewers is achieved, full text screening will begin. All full texts will be independently screened (KM - 100% of records, JC - 33%, JC - 33%, MF - 33%) to reduce quantity of errors.[142] If disagreements cannot be resolved within reviewing pairs, a consensus decision across the research team will be made following a discussion. In line with common practice[143 144] and best-practice

For peer review only - http://bmjopen.bmj.com/site/about/guidelines.xhtml

recommendations,[145] we will contact corresponding and co-authors via e-mail if details of a study are unclear during title/abstract or full text screening (Supplementary Material 6). A maximum of two attempted contacts will be made. The number of corresponding authors contacted, co-authors contacted, replies, and whether adequate information was provided, will be reported. Following full text screening, the remaining relevant studies to the review will undergo forward/backward screening.

Quality and Critical Appraisal

In accordance with best-practice recommendations,[99] we will assess methodological quality before conducting data charting of included studies. As this review will include various study designs, we will employ Joanna Briggs Institute (JBI) appraisal tools respective to each study design (Supplementary Material 7).[146-149] Individual items within each JBI appraisal tool will be assigned either a 'yes' (1), 'no' (0), or 'unclear' (seek clarification/further information) response to questions relating to the research quality. If study details are unclear, corresponding authors will be contacted following the same process as described in Stage 3 – Study Selection (Supplementary Material 5). A quality percentage score will be calculated, with smaller scores indicating lower quality and larger scores indicating higher quality. Identified studies will be included regardless of the quality appraisal score. The appraisals will be conducted by reviewing pairs (KM - 100% of records, JC - 33%, JC - 33%, MF - 33%) using REDCap.[139]

Stage 4: Charting the Data

Data charting will be conducted in REDCap[139] by reviewing pairs (KM - 100% of records, JC - 33%, JC - 33%, MF - 33%) to reduce the risk of errors in data extraction.[142] Data charting will include author(s), country of study, study year, study design, participant age and sex/gender distributions, injury type, surgery type, type of injury management, time since injury/surgery, sporting history before injury (type and level), aim of study, independent variables, supervision level (if intervention), quantity (if intervention), adherence rate (if intervention), drop out (if intervention), outcome variables, who

For peer review only - http://bmjopen.bmj.com/site/about/guidelines.xhtml

assessed the measures, statistical methodology, and the strength of the treatment response or relationship between variables. This form (Supplementary Material 8) will be pilot tested by two reviewers and necessary amendments will be made if required (e.g., other relevant measurement tools or outcome variables). If study details are unclear, corresponding authors will be contacted following the same process as described in Stage 3 – Study Selection.

Stage 5: Summarising the Data

Data will be summarised in three steps.[99] The first step will present the results as a descriptive numerical summary which will include: number of studies on each theme of the research question; frequency of study design; definitions and variables used for joint disease; overview of methodological quality of studies. The second step will present the results as individual sections for each theme of the research question. Each theme will be structured as follows:

- Strength of the treatment response or relationship between variables[150-153] (Supplementary Material 9) for studies presented descriptively
- In accordance with a previous scoping review on ACL patients,[154] we will use the GRADE[89-91] (incorporates Bradford Hill Criteria[155-157]) for quality of evidence for either a protective or degenerative, or if there is an unknown effect (Supplementary Material 10). Study-by-study results will be presented in supplementary material, whilst the overall summary of results for each theme will be displayed within the article in a summary of findings table

The third step will be to discuss and consider the meaning of the findings as they relate to the overall review purpose. We will also discuss implications for future PTOA research.

ETHICS AND DISSEMINATION

For peer review only

Ethical approval is not required as this review maps and synthesises data generated from published literature. This review will be submitted for publication in a peer-reviewed sports medicine journal, irrespective of findings which indicate a positive or negative association between PA and joint health. Knowledge synthesised by this review will be presented at scientific conferences and disseminated via social media platforms.

CONCLUSION

This study seeks to address a salient topic for people who experience a traumatic intra-articular knee injury and clinicians working with this population. Using a scoping review methodology to examine the known influence of PA on PTOA pathogenesis is novel and appropriate, considering the complexity of the topic and the multi-disciplinary evidence base. This research may inform future PTOA research by helping to guide research priorities and identifying outcome measures for clinical interventions.

CRediT (Contributor Roles Taxonomy)

Karl Morgan: Conceptualisation, Methodology, Writing - Original Draft, Writing - Review & Editing. **Dr James Cowburn:** Methodology, Writing - Review & Editing. **Dr Matthew Farrow:** Methodology, Writing - Review & Editing. **Josh Carter :** Methodology, Writing - Review & Editing. **Dr Dario Cazzola:** Writing - Review & Editing, Supervision. **Dr Jean-Philippe Walhin:** Writing - Review & Editing, Supervision. **Dr Carly McKay:** Conceptualisation, Methodology, Writing - Review & Editing, Supervision.

Acknowledgements

We thank Peter Bradley, the University of Bath's Subject Librarian for Health and Social & Policy Sciences for his help in developing the search terms and his role when implementing the Peer

For peer review only - http://bmjopen.bmj.com/site/about/guidelines.xhtml

227 Review of Electronic Search Strategy (PRESS)[116] guidelines. We also thank Dr David Bruce, Dr Nicos Haralabidis, Rosy Hyman, Charli Robertson, and Lee Page for their expert feedback developing the inclusion/exclusion criteria and outcome measures.

Funding statement

This work was funded by the University of Bath (funding refence: EA-FH1112) in affiliation with the Centre for Sport, Exercise and Osteoarthritis Research Versus Arthritis, University of Bath (grant reference: 21595).

Conflict of interest statement

None declared.

For peer review only - http://bmjopen.bmj.com/site/about/guidelines.xhtml

REFERENCES

1. Cochrane JL, Lloyd DG, Buttfield A, et al. Characteristics of anterior cruciate ligament injuries in Australian football. *J Sci Med Sport* 2007;10(2):96-104. doi: 10.1016/j.jsams.2006.05.015 [published Online First: 20060627]

2. Walden M, Krosshaug T, Bjorneboe J, et al. Three distinct mechanisms predominate in non-contact anterior cruciate ligament injuries in male professional football players: a systematic video analysis of 39 cases. *Br J Sports Med* 2015;49(22):1452-60. doi: 10.1136/bjsports-2014-094573 [published Online First: 2015/04/25]

3. Montgomery C, Blackburn J, Withers D, et al. Mechanisms of ACL injury in professional rugby union: a systematic video analysis of 36 cases. *Br J Sports Med* 2018;52(15):994-1001. doi: 10.1136/bjsports-2016-096425 [published Online First: 2017/01/01]

4. Boden BP, Dean GS, Feagin JA, et al. Mechanisms of anterior cruciate ligament injury. *Orthopedics* 2000;23(6):573-8.

5. Olsen OE, Myklebust G, Engebretsen L, et al. Injury mechanisms for anterior cruciate ligament injuries in team handball: a systematic video analysis. *Am J Sports Med* 2004;32(4):1002-12. doi: 10.1177/0363546503261724 [published Online First: 2004/05/20]

6. Koga H, Nakamae A, Shima Y, et al. Mechanisms for noncontact anterior cruciate ligament injuries: knee joint kinematics in 10 injury situations from female team handball and basketball. *Am J Sports Med* 2010;38(11):2218-25. doi: 10.1177/0363546510373570 [published Online First: 2010/07/03]

7. Ruedl G, Linortner I, Schranz A, et al. Distribution of injury mechanisms and related factors in ACL-injured female carving skiers. *Knee Surg Sports Traumatol Arthrosc* 2009;17(11):1393-8. doi: 10.1007/s00167-009-0860-7 [published Online First: 2009/07/11]

8. Ruedl G, Webhofer M, Linortner I, et al. ACL injury mechanisms and related factors in male and female carving skiers: a retrospective study. *Int J Sports Med* 2011;32(10):801-6. doi: 10.1055/s-0031-1279719 [published Online First: 2011/06/23]

9. Gabr A, De Medici A, Haddad F. The National Ligament Registry 2019 Annual Report. In: Spalding T, ed. The National Ligament Registry, 2020:1-14.

10. Moses B, Orchard J, Orchard J. Systematic review: Annual incidence of ACL injury and surgery in various populations. *Res Sports Med* 2012;20(3-4):157-79. doi: 10.1080/15438627.2012.680633 [published Online First: 2012/06/30]

11. Noyes FR, Barber-Westin SD. Treatment of meniscus tears during anterior cruciate ligament reconstruction. *Arthroscopy* 2012;28(1):123-30. doi: 10.1016/j.arthro.2011.08.292 [published Online First: 2011/11/15]

12. Ghodadra N, Mall NA, Karas V, et al. Articular and meniscal pathology associated with primary anterior cruciate ligament reconstruction. *J Knee Surg* 2013;26(3):185-93. doi: 10.1055/s-0032-1327450 [published Online First: 2013/01/05]

For peer review only - http://bmjopen.bmj.com/site/about/guidelines.xhtml

13. Mall NA, Chalmers PN, Moric M, et al. Incidence and trends of anterior cruciate ligament reconstruction in the United States. *Am J Sports Med* 2014;42(10):2363-70. doi: 10.1177/0363546514542796 [published Online First: 2014/08/03]

14. Everhart JS, DiBartola AC, Swank K, et al. Cartilage damage at the time of anterior cruciate ligament reconstruction is associated with weaker quadriceps function and lower risk of future ACL injury. *Knee Surg Sports Traumatol Arthrosc* 2020;28(2):576-83. doi: 10.1007/s00167-019-05739-1 [published Online First: 2019/10/11]

15. Duthon VB, Barea C, Abrassart S, et al. Anatomy of the anterior cruciate ligament. *Knee Surg Sports Traumatol Arthrosc* 2006;14(3):204-13. doi: 10.1007/s00167-005-0679-9 [published Online First: 2005/10/20]

16. Lohmander LS, Englund PM, Dahl LL, et al. The long-term consequence of anterior cruciate ligament and meniscus injuries: osteoarthritis. *Am J Sports Med* 2007;35(10):1756-69. doi: 10.1177/0363546507307396 [published Online First: 2007/09/01]

17. Abram SGF, Price AJ, Judge A, et al. Anterior cruciate ligament (ACL) reconstruction and meniscal repair rates have both increased in the past 20 years in England: hospital statistics from 1997 to 2017. *Br J Sports Med* 2020;54(5):286-91. doi: 10.1136/bjsports-2018-100195 [published Online First: 2019/01/21]

18. Domnick C, Garcia P, Raschke MJ, et al. Trends and incidences of ligament-surgeries and osteotomies of the knee: an analysis of German inpatient records 2005-2013. *Arch Orthop Trauma Surg* 2017;137(7):989-95. doi: 10.1007/s00402-017-2704-0 [published Online First: 2017/05/04]

19. Zbrojkiewicz D, Vertullo C, Grayson JE. Increasing rates of anterior cruciate ligament reconstruction in young Australians, 2000-2015. *Med J Aust* 2018;208(8):354-58. doi: doi:10.5694/mja17.00974 [published Online First: 2018/04/20]

20. Snoeker B, Turkiewicz A, Magnusson K, et al. Risk of knee osteoarthritis after different types of knee injuries in young adults: a population-based cohort study. *Br J Sports Med* 2020;54(12):725-30. doi: 10.1136/bjsports-2019-100959 [published Online First: 2019/12/13]

21. Morgan OJ, Hillstrom HJ, Ellis SJ, et al. Osteoarthritis in England: incidence trends from National Health Service Hospital Episode Statistics. *ACR Open Rheumatol* 2019;1(8):493-98. doi: 10.1002/acr2.11071 [published Online First: 2019/11/30]

22. Wallace IJ, Worthington S, Felson DT, et al. Knee osteoarthritis has doubled in prevalence since the mid-20th century. *Proc Natl Acad Sci U S A* 2017;114(35):9332-36. doi: 10.1073/pnas.1703856114 [published Online First: 2017/08/16]

23. Kiadaliri AA, Rinaldi G, Lohmander LS, et al. Temporal trend and regional disparity in osteoarthritis hospitalisations in Sweden 1998–2015. *Scand J Public Health* 2019;47(1):53-60. doi: 10.1177/1403494818766785

24. Kraus VB, Blanco FJ, Englund M, et al. Call for standardized definitions of osteoarthritis and risk stratification for clinical trials and clinical use. *Osteoarthritis Cartilage* 2015;23(8):1233-41. doi: 10.1016/j.joca.2015.03.036 [published Online First: 2015/04/14]

For peer review only - http://bmjopen.bmj.com/site/about/guidelines.xhtml

25. Illingworth KD, El Bitar Y, Siewert K, et al. Correlation of WOMAC and KOOS scores to tibiofemoral cartilage loss on plain radiography and 3 Tesla MRI: data from the osteoarthritis initiative. *Knee Surg Sports Traumatol Arthrosc* 2014;22(7):1649-58.

26. Bedson J, Croft PR. The discordance between clinical and radiographic knee osteoarthritis: a systematic search and summary of the literature. *BMC Musculoskelet Disord* 2008;9:116. doi: 10.1186/1471-2474-9-116 [published Online First: 2008/09/04]

27. Whittaker JL, Toomey CM, Woodhouse LJ, et al. Association between MRI-defined osteoarthritis, pain, function and strength 3-10 years following knee joint injury in youth sport. *Br J Sports Med* 2018;52(14):934-39. doi: 10.1136/bjsports-2017-097576 [published Online First: 2017/10/12]

28. Friel NA, Chu CR. The role of ACL injury in the development of posttraumatic knee osteoarthritis. *Clin Sports Med* 2013;32(1):1-12. doi: 10.1016/j.csm.2012.08.017 [published Online First: 2012/11/28]

29. Luc B, Gribble PA, Pietrosimone BG. Osteoarthritis prevalence following anterior cruciate ligament reconstruction: a systematic review and numbers-needed-to-treat analysis. *J Athl Train* 2014;49(6):806-19. doi: 10.4085/1062-6050-49.3.35 [published Online First: 2014/09/19]

30. Gelber AC, Hochberg MC, Mead LA, et al. Joint injury in young adults and risk for subsequent knee and hip osteoarthritis. *Ann Intern Med* 2000;133(5):321-8. doi: 10.7326/0003-4819-133-5-200009050-00007 [published Online First: 2000/09/09]

31. Roos EM. Joint injury causes knee osteoarthritis in young adults. *Curr Opin Rheumatol* 2005;17(2):195-200. doi: 10.1097/01.bor.0000151406.64393.00 [published Online First: 2005/02/16]

32. Chen T, Wang S, Li Y, et al. Radiographic osteoarthritis prevalence over ten years after anterior cruciate ligament reconstruction. *Int J Sports Med* 2019;40(11):683-95. doi: 10.1055/a-0902-8539 [published Online First: 2019/09/19]

33. Losina E, Weinstein AM, Reichmann WM, et al. Lifetime risk and age at diagnosis of symptomatic knee osteoarthritis in the US. *Arthritis Care Res (Hoboken)* 2013;65(5):703-11. doi: 10.1002/acr.21898 [published Online First: 2012/12/04]

34. Khan T, Alvand A, Prieto-Alhambra D, et al. ACL and meniscal injuries increase the risk of primary total knee replacement for osteoarthritis: a matched case-control study using the Clinical Practice Research Datalink (CPRD). *Br J Sports Med* 2019;53(15):965-68. doi: 10.1136/bjsports-2017-097762 [published Online First: 2018/01/15]

35. Price AJ, Alvand A, Troelsen A, et al. Knee replacement. *Lancet* 2018;392(10158):1672-82. doi: 10.1016/s0140-6736(18)32344-4

36. W-Dahl A, Robertsson O, Lidgren L. Surgery for knee osteoarthritis in younger patients. *Acta Orthop* 2010;81(2):161-64. doi: 10.3109/17453670903413186

37. Evans JT, Walker RW, Evans JP, et al. How long does a knee replacement last? A systematic review and meta-analysis of case series and national registry reports with more than 15 years of follow-

For peer review only - http://bmjopen.bmj.com/site/about/guidelines.xhtml

up. *Lancet* 2019;393(10172):655-63. doi: 10.1016/S0140-6736(18)32531-5 [published Online First: 2019/02/21]

38. Bayliss LE, Culliford D, Monk AP, et al. The effect of patient age at intervention on risk of implant revision after total replacement of the hip or knee: a population-based cohort study. *Lancet* 2017;389(10077):1424-30. doi: 10.1016/s0140-6736(17)30059-4

39. Wasserstein D, Huston LJ, Nwosu S, et al. KOOS pain as a marker for significant knee pain two and six years after primary ACL reconstruction: a Multicenter Orthopaedic Outcomes Network (MOON) prospective longitudinal cohort study. *Osteoarthritis Cartilage* 2015;23(10):1674-84. doi: 10.1016/j.joca.2015.05.025 [published Online First: 2015/06/15]

40. Antosh IJ, Svoboda SJ, Peck KY, et al. Change in KOOS and WOMAC scores in a young athletic population with and without anterior cruciate ligament injury. *Am J Sports Med* 2018;46(7):1606-16. doi: 10.1177/0363546518768753 [published Online First: 2018/05/08]

41. Whittaker JL, Woodhouse LJ, Nettel-Aguirre A, et al. Outcomes associated with early post-traumatic osteoarthritis and other negative health consequences 3-10 years following knee joint injury in youth sport. *Osteoarthritis Cartilage* 2015;23(7):1122-9. doi: 10.1016/j.joca.2015.02.021 [published Online First: 2015/03/01]

42. Englund M, Roos EM, Lohmander LS. Impact of type of meniscal tear on radiographic and symptomatic knee osteoarthritis: a sixteen-year followup of meniscectomy with matched controls. *Arthritis Rheum* 2003;48(8):2178-87. doi: 10.1002/art.11088 [published Online First: 2003/08/09]

43. Harkey MS, Baez S, Lewis J, et al. Prevalence of early knee osteoarthritis illness among various patient-reported classification criteria after anterior cruciate ligament reconstruction. *Arthritis Care Res (Hoboken)* 2022;74(3):377-85. doi: 10.1002/acr.24809

44. Arden N, Nevitt MC. Osteoarthritis: epidemiology. *Best Pract Res Clin Rheumatol* 2006;20(1):3-25. doi: 10.1016/j.berh.2005.09.007 [published Online First: 2006/02/18]

45. Rathleff MS, Roos EM, Olesen JL, et al. High prevalence of daily and multi-site pain--a cross-sectional population-based study among 3000 Danish adolescents. *BMC Pediatr* 2013;13:191. doi: 10.1186/1471-2431-13-191 [published Online First: 2013/11/21]

46. Peat G. Knee pain and osteoarthritis in older adults: a review of community burden and current use of primary health care. *Ann Rheum Dis* 2001;60(2):91-97. doi: 10.1136/ard.60.2.91

47. Turkiewicz A, Gerhardsson De Verdier M, Engstrom G, et al. Prevalence of knee pain and knee OA in southern Sweden and the proportion that seeks medical care. *Rheumatology (Oxford)* 2015;54(5):827-35. doi: 10.1093/rheumatology/keu409

48. Holla JF, Sanchez-Ramirez DC, van der Leeden M, et al. The avoidance model in knee and hip osteoarthritis: a systematic review of the evidence. *J Behav Med* 2014;37(6):1226-41. doi: 10.1007/s10865-014-9571-8 [published Online First: 2014/05/21]

For peer review only - http://bmjopen.bmj.com/site/about/guidelines.xhtml

49. Shortland A. Muscle deficits in cerebral palsy and early loss of mobility: can we learn something from our elders? *Dev Med Child Neurol* 2009;51:59-63. doi: 10.1111/j.1469-8749.2009.03434.x

50. Osteoarthritis Research Society International. Osteoarthritis: A Serious Disease: Osteoarthritis Research Society International, 2016.

51. Whittaker JL, Roos EM. A pragmatic approach to prevent post-traumatic osteoarthritis after sport or exercise-related joint injury. *Best Pract Res Clin Rheumatol* 2019;33(1):158-71. doi: 10.1016/j.berh.2019.02.008 [published Online First: 2019/08/23]

52. Kvist J, Ek A, Sporrstedt K, et al. Fear of re-injury: a hindrance for returning to sports after anterior cruciate ligament reconstruction. *Knee Surg Sports Traumatol Arthrosc* 2005;13(5):393-7. doi: 10.1007/s00167-004-0591-8 [published Online First: 2005/02/11]

53. Ardern CL, Osterberg A, Sonesson S, et al. Satisfaction with knee function after primary anterior cruciate ligament reconstruction is associated with self-efficacy, quality of life, and returning to the preinjury physical activity. *Arthroscopy* 2016;32(8):1631-38. doi: 10.1016/j.arthro.2016.01.035 [published Online First: 2016/03/30]

54. Toomey CM, Whittaker JL, Nettel-Aguirre A, et al. Higher fat mass is associated with a history of knee injury in youth sport. *J Orthop Sports Phys Ther* 2017;47(2):80-87. doi: 10.2519/jospt.2017.7101 [published Online First: 2017/02/02]

55. Bell DR, Pfeiffer KA, Cadmus-Bertram LA, et al. Objectively measured physical activity in patients after anterior cruciate ligament reconstruction. *Am J Sports Med* 2017;45(8):1893-900. doi: 10.1177/0363546517698940 [published Online First: 2017/04/19]

56. Toomey C, Whittaker JL, Doyle-Baker PK, et al. The association between physical activity and 3–15 year history of sport-related intra-articular knee injury: a matched cohort design [Abstract]. *Br J Sports Med* 2018; 52. https://bjsm.bmj.com/content/52/Suppl_1/A6.1 (accessed 01/04/2021).

57. Kuenze C, Cadmus-Bertram L, Pfieffer K, et al. Relationship between physical activity and clinical outcomes after ACL reconstruction. *J Sport Rehabil* 2019;28(2):180-87. doi: 10.1123/jsr.2017-0186 [published Online First: 2017/11/16]

58. Kuenze C, Lisee C, Pfeiffer KA, et al. Sex differences in physical activity engagement after ACL reconstruction. *Phys Ther Sport* 2019;35:12-17. doi: 10.1016/j.ptsp.2018.10.016 [published Online First: 2018/11/06]

59. Triplett AN, Kuenze CM. Characterizing body composition, cardiorespiratory fitness, and physical activity in women with anterior cruciate ligament reconstruction. *Phys Ther Sport* 2020;48:54-59. doi: 10.1016/j.ptsp.2020.12.014 [published Online First: 2020/12/30]

60. Baez SE, Hoch MC, Hoch JM. Psychological factors are associated with return to pre-injury levels of sport and physical activity after ACL reconstruction. *Knee Surg Sports Traumatol Arthrosc* 2020;28(2):495-501. doi: 10.1007/s00167-019-05696-9 [published Online First: 2019/09/06]

For peer review only - http://bmjopen.bmj.com/site/about/guidelines.xhtml

61. Webster KE, Feller JA. Expectations for return to preinjury sport before and after anterior cruciate ligament reconstruction. *Am J Sports Med* 2019;47(3):578-83. doi: 10.1177/0363546518819454 [published Online First: 2019/01/17]

62. Ardern CL, Webster KE, Taylor NF, et al. Return to sport following anterior cruciate ligament reconstruction surgery: a systematic review and meta-analysis of the state of play. *Br J Sports Med* 2011;45(7):596-606. doi: 10.1136/bjsm.2010.076364 [published Online First: 2011/03/15]

63. Ardern CL, Taylor NF, Feller JA, et al. Fifty-five per cent return to competitive sport following anterior cruciate ligament reconstruction surgery: an updated systematic review and meta-analysis including aspects of physical functioning and contextual factors. *Br J Sports Med* 2014;48(21):1543-52. doi: 10.1136/bjsports-2013-093398

64. National Institute for Health and Care Excellence. Osteoarthritis: care and management (update): National Institute for Health and Care Excellence; 2022 [Available from: https://www.nice.org.uk/guidance/indevelopment/gid-ng10127/consultation/html-content-2 accessed 31/05/2022 2022.

65. MacAlpine EM, Talwar D, Storey EP, et al. Weight gain after ACL reconstruction in pediatric and adolescent patients. *Sports Health* 2020;12(1):29-35. doi: 10.1177/1941738119870192 [published Online First: 2019/09/06]

66. Patterson BE, Culvenor AG, Barton CJ, et al. Worsening knee osteoarthritis features on magnetic resonance imaging 1 to 5 years after anterior cruciate ligament reconstruction. *Am J Sports Med* 2018;46(12):2873-83. doi: 10.1177/0363546518789685 [published Online First: 2018/09/05]

67. Ezzat AM, Whittaker JL, Toomey C, et al. Knee confidence in youth and young adults at risk of post-traumatic osteoarthritis 3-10 years following intra-articular knee injury. *J Sci Med Sport* 2018;21(7):671-75. doi: 10.1016/j.jsams.2017.11.012 [published Online First: 2017/12/14]

68. Tayfur B, Charuphongsa C, Morrissey D, et al. Neuromuscular function of the knee joint following knee injuries: does it ever get back to normal? A systematic review with meta-analyses. *Sports Med* 2021;51(2):321-38. doi: 10.1007/s40279-020-01386-6 [published Online First: 2020/11/29]

69. Hart JM, Pietrosimone B, Hertel J, et al. Quadriceps activation following knee injuries: a systematic review. *J Athl Train* 2010;45(1):87-97. doi: 10.4085/1062-6050-45.1.87 [published Online First: 2010/01/13]

70. Lisee C, Lepley AS, Birchmeier T, et al. Quadriceps strength and volitional activation after anterior cruciate ligament reconstruction: a systematic review and meta-analysis. *Sports Health* 2019;11(2):163-79. doi: 10.1177/1941738118822739 [published Online First: 2019/01/15]

71. Birchmeier T, Lisee C, Kane K, et al. Quadriceps muscle size following ACL injury and reconstruction: a systematic review. *J Orthop Res* 2020;38(3):598-608. doi: 10.1002/jor.24489 [published Online First: 2019/10/15]

For peer review only - http://bmjopen.bmj.com/site/about/guidelines.xhtml

72. Dutaillis B, Maniar N, Opar DA, et al. Lower limb muscle size after anterior cruciate ligament injury: a systematic review and meta-analysis. *Sports Med* 2021; in press. https://www.ncbi.nlm.nih.gov/pubmed/33492623

https://link.springer.com/content/pdf/10.1007/s40279-020-01419-0.pdf (accessed 15/03/2021).

73. Lindstrom M, Strandberg S, Wredmark T, et al. Functional and muscle morphometric effects of ACL reconstruction. A prospective CT study with 1 year follow-up. *Scand J Med Sci Sports* 2013;23(4):431-42. doi: 10.1111/j.1600-0838.2011.01417.x [published Online First: 2011/11/24]

74. Lopresti C, Kirkendall DT, Street GM, et al. Quadriceps insufficiency following repair of the anterior cruciate ligament. *J Orthop Sports Phys Ther* 1988;9(7):245-49.

75. Van Meer BL, Waarsing JH, Van Eijsden WA, et al. Bone mineral density changes in the knee following anterior cruciate ligament rupture. *Osteoarthritis Cartilage* 2014;22(1):154-61. doi: 10.1016/j.joca.2013.11.005

76. Fischer CP, Berntsen A, Perstrup LB, et al. Plasma levels of interleukin-6 and C-reactive protein are associated with physical inactivity independent of obesity. *Scand J Med Sci Sports* 2007;17(5):580-7. doi: 10.1111/j.1600-0838.2006.00602.x [published Online First: 2006/11/02]

77. Hamer M. The relative influences of fitness and fatness on inflammatory factors. *Prev Med* 2007;44(1):3-11. doi: 10.1016/j.ypmed.2006.09.005 [published Online First: 2006/10/27]

78. Lavin KM, Perkins RK, Jemiolo B, et al. Effects of aging and lifelong aerobic exercise on basal and exercise-induced inflammation. *J Appl Physiol (1985)* 2020;128(1):87-99. doi: 10.1152/japplphysiol.00495.2019 [published Online First: 2019/11/22]

79. Myers A, Gibbons C, Finlayson G, et al. Associations among sedentary and active behaviours, body fat and appetite dysregulation: investigating the myth of physical inactivity and obesity. *Br J Sports Med* 2017;51(21):1540-44. doi: 10.1136/bjsports-2015-095640 [published Online First: 2016/04/06]

80. Krogh-Madsen R, Thyfault JP, Broholm C, et al. A 2-wk reduction of ambulatory activity attenuates peripheral insulin sensitivity. *J Appl Physiol (1985)* 2010;108(5):1034-40. doi: 10.1152/japplphysiol.00977.2009 [published Online First: 2010/01/02]

81. Manini TM, Clark BC, Nalls MA, et al. Reduced physical activity increases intermuscular adipose tissue in healthy young adults. *Am J Clin Nutr* 2007;85(2):377-84. doi: 10.1093/ajcn/85.2.377 [published Online First: 2007/02/08]

82. Farr JN, Van Loan MD, Lohman TG, et al. Lower physical activity is associated with skeletal muscle fat content in girls. *Med Sci Sports Exerc* 2012;44(7):1375-81. doi: 10.1249/MSS.0b013e31824749b2 [published Online First: 2012/01/06]

83. Dimmick HL, Miller JD, Sterczala AJ, et al. Vastus lateralis muscle tissue composition and motor unit properties in chronically endurance-trained vs. sedentary women. *Eur J Appl Physiol* 2018;118(9):1789-800. doi: 10.1007/s00421-018-3909-9 [published Online First: 2018/06/28]

For peer review only - http://bmjopen.bmj.com/site/about/guidelines.xhtml

84. Pagano AF, Brioche T, Arc-Chagnaud C, et al. Short-term disuse promotes fatty acid infiltration into skeletal muscle. *J Cachexia Sarcopenia Muscle* 2018;9(2):335-47. doi: 10.1002/jcsm.12259 [published Online First: 2017/12/17]

85. MacLennan RJ, Sahebi M, Becker N, et al. Declines in skeletal muscle quality vs. size following two weeks of knee joint immobilization. *PeerJ* 2020; 8. https://www.ncbi.nlm.nih.gov/pmc/articles/PMC6964688/

https://www.ncbi.nlm.nih.gov/pmc/articles/PMC6964688/pdf/peerj-08-8224.pdf (accessed 18/04/2021).

86. Tuttle LJ, Sinacore DR, Cade WT, et al. Lower physical activity is associated with higher intermuscular adipose tissue in people with type 2 diabetes and peripheral neuropathy. *Phys Ther* 2011;91(6):923-30. doi: 10.2522/ptj.20100329 [published Online First: 2011/04/09]

87. Chastin SF, Mandrichenko O, Helbostadt J, et al. Associations between objectively-measured sedentary behaviour and physical activity with bone mineral density in adults and older adults, the NHANES study. *Bone* 2014;64:254-62.

88. Nordström A, Olsson T, Nordström P. Sustained Benefits from Previous Physical Activity on Bone Mineral Density in Males. *J Clin Endocrinol Metab* 2006;91(7):2600-04. doi: 10.1210/jc.2006-0151

89. Guyatt GH, Oxman AD, Kunz R, et al. What is "quality of evidence" and why is it important to clinicians? *BMJ* 2008;336(7651):995-98. doi: 10.1136/bmj.39490.551019.be

90. Guyatt GH, Oxman AD, Vist GE, et al. GRADE: an emerging consensus on rating quality of evidence and strength of recommendations. *BMJ* 2008;336(7650):924-26. doi: 10.1136/bmj.39489.470347.ad

91. Guyatt G, Oxman AD, Akl EA, et al. GRADE guidelines: 1. Introduction-GRADE evidence profiles and summary of findings tables. *J Clin Epidemiol* 2011;64(4):383-94. doi: 10.1016/j.jclinepi.2010.04.026 [published Online First: 20101231]

92. Grimshaw J. A Guide to Knowledge Synthesis: A Knowledge Synthesis Chapter: Canadian Institutes of Health Research, 2020.

93. Peters MDJ, Marnie C, Tricco AC, et al. Updated methodological guidance for the conduct of scoping reviews. *JBI Evid Synth* 2020;18(10):2119-26. doi: 10.11124/jbies-20-00167

94. Khalil H, Peters MD, Tricco AC, et al. Conducting high quality scoping reviews-challenges and solutions. *J Clin Epidemiol* 2021;130:156-60. doi: 10.1016/j.jclinepi.2020.10.009

95. Peters MDJ, Godfrey C, McInerney P, et al. Best practice guidance and reporting items for the development of scoping review protocols. *JBI Evid Synth* 2022;20(4)

96. Shamseer L, Moher D, Clarke M, et al. Preferred reporting items for systematic review and meta-analysis protocols (PRISMA-P) 2015: elaboration and explanation. *BMJ* 2015;349(jan02 1):g7647-g47. doi: 10.1136/bmj.g7647

For peer review only - http://bmjopen.bmj.com/site/about/guidelines.xhtml

97. Moher D, Shamseer L, Clarke M, et al. Preferred reporting items for systematic review and meta-analysis protocols (PRISMA-P) 2015 statement. *Syst Rev* 2015;4(1):1. doi: 10.1186/2046-4053-4-1

98. Tricco AC, Lillie E, Zarin W, et al. PRISMA Extension for Scoping Reviews (PRISMA-ScR): checklist and explanation. *Ann Intern Med* 2018;169(7):467-73. doi: 10.7326/m18-0850

99. Levac D, Colquhoun H, O'Brien KK. Scoping studies: advancing the methodology. *Implement Sci* 2010;5(1):69. doi: 10.1186/1748-5908-5-69

100. Pham MT, Rajić A, Greig JD, et al. A scoping review of scoping reviews: advancing the approach and enhancing the consistency. *Res Synth Methods* 2014;5(4):371-85. doi: 10.1002/jrsm.1123

101. Munn Z, Peters MDJ, Stern C, et al. Systematic review or scoping review? Guidance for authors when choosing between a systematic or scoping review approach. *BMC Med Res Methodol* 2018;18(1):143. doi: 10.1186/s12874-018-0611-x [published Online First: 2018/11/21]

102. Arksey H, O'Malley L. Scoping studies: towards a methodological framework. *Int J Soc Res Methodol* 2005;8(1):19-32. doi: 10.1080/1364557032000119616

103. Helman CG. Disease versus illness in general practice. *J R Coll Gen Pract* 1981;31(230):548-52.

104. Caspersen CJ, Powell KE, Christenson GM. Physical activity, exercise, and physical fitness: definitions and distinctions for health-related research. *Public Health Rep* 1985;100(2):126-31. [published Online First: 1985/03/01]

105. Bricca A, Struglics A, Larsson S, et al. Impact of exercise therapy on molecular biomarkers related to cartilage and inflammation in individuals at risk of, or with established, knee osteoarthritis: a systematic review and meta-analysis of randomized controlled trials. *Arthritis Care Res (Hoboken)* 2019;71(11):1504-15. doi: 10.1002/acr.23786

106. Stannus O, Jones G, Blizzard L, et al. Associations between serum levels of inflammatory markers and change in knee pain over 5 years in older adults: a prospective cohort study. *Ann Rheum Dis* 2013;72(4):535-40. doi: 10.1136/annrheumdis-2011-201047

107. Stannus O, Jones G, Cicuttini F, et al. Circulating levels of IL-6 and TNF-alpha are associated with knee radiographic osteoarthritis and knee cartilage loss in older adults. *Osteoarthritis Cartilage* 2010;18(11):1441-7. doi: 10.1016/j.joca.2010.08.016 [published Online First: 2010/09/08]

108. Ruan G, Xu J, Wang K, et al. Associations between knee structural measures, circulating inflammatory factors and MMP13 in patients with knee osteoarthritis. *Osteoarthritis Cartilage* 2018;26(8):1063-69. doi: 10.1016/j.joca.2018.05.003 [published Online First: 2018/05/14]

109. Kobayashi M, Squires GR, Mousa A, et al. Role of interleukin-1 and tumor necrosis factor α in matrix degradation of human osteoarthritic cartilage. *Arthritis Rheum* 2005;52(1):128-35. doi: 10.1002/art.20776

For peer review only

110. Wojdasiewicz P, Poniatowski LA, Szukiewicz D. The role of inflammatory and anti-inflammatory cytokines in the pathogenesis of osteoarthritis. *Mediators Inflamm* 2014; 2014. https://www.hindawi.com/journals/mi/2014/561459/

https://downloads.hindawi.com/journals/mi/2014/561459.pdf (accessed 03/02/2021).

111. Williams TD, Tolusso DV, Fedewa MV, et al. Comparison of Periodized and Non-Periodized Resistance Training on Maximal Strength: A Meta-Analysis. *Sports Med* 2017;47(10):2083-100. doi: 10.1007/s40279-017-0734-y

112. Shen W, Wang Z, Punyanita M, et al. Adipose tissue quantification by imaging methods: a proposed classification. *Obes Res* 2003;11(1):5-16. doi: 10.1038/oby.2003.3 [published Online First: 2003/01/17]

113. Thompson D, Karpe F, Lafontan M, et al. Physical Activity and Exercise in the Regulation of Human Adipose Tissue Physiology. *Physiol Rev* 2012;92(1):157-91. doi: 10.1152/physrev.00012.2011

114. Addison O, Marcus RL, Lastayo PC, et al. Intermuscular fat: a review of the consequences and causes. *Int J Endocrinol* 2014;2014:309570. doi: 10.1155/2014/309570 [published Online First: 2014/02/15]

115. Wisdom KM, Delp SL, Kuhl E. Use it or lose it: multiscale skeletal muscle adaptation to mechanical stimuli. *Biomech Model Mechanobiol* 2015;14(2):195-215. doi: 10.1007/s10237-014-0607-3 [published Online First: 2014/09/10]

116. McGowan J, Sampson M, Salzwedel DM, et al. PRESS Peer Review of Electronic Search Strategies: 2015 Guideline Statement. *J Clin Epidemiol* 2016;75:40-46. doi: 10.1016/j.jclinepi.2016.01.021

117. Chambat P, Guier C, Sonnery-Cottet B, et al. The evolution of ACL reconstruction over the last fifty years. *Int Orthop* 2013;37(2):181-86. doi: 10.1007/s00264-012-1759-3

118. Di Matteo B, Moran CJ, Tarabella V, et al. A history of meniscal surgery: from ancient times to the twenty-first century. *Knee Surg Sports Traumatol Arthrosc* 2016;24(5):1510-18. doi: 10.1007/s00167-015-3717-2

119. Peters MD, Godfrey CM, Khalil H, et al. Guidance for conducting systematic scoping reviews. *Int J Evid Based Healthc* 2015;13(3):141-46.

120. Paez A. Grey literature: An important resource in systematic reviews. *J Evid Based Med* 2017 doi: 10.1111/jebm.12265

121. Kellgren JH, Lawrence JS. Radiological Assessment of Osteo-Arthrosis. *Ann Rheum Dis* 1957;16(4):494-502. doi: 10.1136/ard.16.4.494

122. Altman RD, Gold GE. Atlas of individual radiographic features in osteoarthritis, revised. *Osteoarthritis Cartilage* 2007;15:A1-A56. doi: 10.1016/j.joca.2006.11.009

For peer review only

123. Hunter DJ, Guermazi A, Lo GH, et al. Evolution of semi-quantitative whole joint assessment of knee OA: MOAKS (MRI Osteoarthritis Knee Score). *Osteoarthritis Cartilage* 2011;19(8):990-1002. doi: 10.1016/j.joca.2011.05.004 [published Online First: 2011/06/08]

124. Peterfy CG, Guermazi A, Zaim S, et al. Whole-Organ Magnetic Resonance Imaging Score (WORMS) of the knee in osteoarthritis. *Osteoarthritis Cartilage* 2004;12(3):177-90. doi: 10.1016/j.joca.2003.11.003

125. Hunter DJ, Lo GH, Gale D, et al. The reliability of a new scoring system for knee osteoarthritis MRI and the validity of bone marrow lesion assessment: BLOKS (Boston Leeds Osteoarthritis Knee Score). *Ann Rheum Dis* 2008;67(2):206-11. doi: 10.1136/ard.2006.066183 [published Online First: 2007/05/03]

126. Eckstein F, Cicuttini F, Raynauld JP, et al. Magnetic resonance imaging (MRI) of articular cartilage in knee osteoarthritis (OA): morphological assessment. *Osteoarthritis Cartilage* 2006;14:46-75. doi: 10.1016/j.joca.2006.02.026

127. Dunn TC, Lu Y, Jin H, et al. T2 relaxation time of cartilage at MR imaging: comparison with severity of knee osteoarthritis. *Radiology* 2004;232(2):592-8. doi: 10.1148/radiol.2322030976 [published Online First: 20040623]

128. Burstein D, Velyvis J, Scott KT, et al. Protocol issues for delayed Gd(DTPA)2--enhanced MRI (dGEMRIC) for clinical evaluation of articular cartilage. *Magn Reson Med* 2001;45(1):36-41. doi: 10.1002/1522-2594(200101)45:1<36::aid-mrm1006>3.0.co;2-w

129. Bashir A, Gray ML, Boutin RD, et al. Glycosaminoglycan in articular cartilage: in vivo assessment with delayed Gd(DTPA)(2-)-enhanced MR imaging. *Radiology* 1997;205(2):551-8. doi: 10.1148/radiology.205.2.9356644

130. Moller I, Bong D, Naredo E, et al. Ultrasound in the study and monitoring of osteoarthritis. *Osteoarthritis Cartilage* 2008;16:S4-7. doi: 10.1016/j.joca.2008.06.005 [published Online First: 2008/09/02]

131. Naredo E, Acebes C, Moller I, et al. Ultrasound validity in the measurement of knee cartilage thickness. *Ann Rheum Dis* 2009;68(8):1322-7. doi: 10.1136/ard.2008.090738 [published Online First: 2008/08/08]

132. Schmitz RJ, Wang HM, Polprasert DR, et al. Evaluation of knee cartilage thickness: A comparison between ultrasound and magnetic resonance imaging methods. *The Knee* 2017;24(2):217-23. doi: 10.1016/j.knee.2016.10.004 [published Online First: 2016/12/05]

133. Kraus VB, Collins JE, Hargrove D, et al. Predictive validity of biochemical biomarkers in knee osteoarthritis: data from the FNIH OA Biomarkers Consortium. *Ann Rheum Dis* 2017;76(1):186-95. doi: 10.1136/annrheumdis-2016-209252 [published Online First: 2016/06/15]

134. Roberts HM, Law RJ, Thom JM. The time course and mechanisms of change in biomarkers of joint metabolism in response to acute exercise and chronic training in physiologic and pathological conditions. *Eur J Appl Physiol* 2019;119(11-12):2401-20. doi: 10.1007/s00421-019-04232-4 [published Online First: 2019/10/28]

For peer review only - http://bmjopen.bmj.com/site/about/guidelines.xhtml

135. Bay-Jensen AC, Mobasheri A, Thudium CS, et al. Blood and urine biomarkers in osteoarthritis–an update on cartilage associated type II collagen and aggrecan markers. *Curr Opin Rheumatol* 2022;34(1):54-60.

136. Engbersen MP, Huang Z, Kraus VB. Bone Biomarkers Related to Osteoarthritis: Springer Netherlands 2017:993-1021.

137. Van Spil WE, Degroot J, Lems WF, et al. Serum and urinary biochemical markers for knee and hip-osteoarthritis: a systematic review applying the consensus BIPED criteria. *Osteoarthritis and cartilage* 2010;18(5):605-12. doi: 10.1016/j.joca.2010.01.012

138. Li T, Vedula SS, Hadar N, et al. Innovations in data collection, management, and archiving for systematic reviews. *Ann Intern Med* 2015;162(4):287-94.

139. Harris PA, Taylor R, Minor BL, et al. The REDCap consortium: Building an international community of software platform partners. *J Biomed Inform* 2019;95:103208. doi: 10.1016/j.jbi.2019.103208 [published Online First: 2019/05/13]

140. Cohen J. A coefficient of agreement for nominal scales. *Educational and psychological measurement* 1960;20(1):37-46.

141. McHugh ML. Interrater reliability: the kappa statistic. *Biochem Med (Zagreb)* 2012;22(3):276-82.

142. Buscemi N, Hartling L, Vandermeer B, et al. Single data extraction generated more errors than double data extraction in systematic reviews. *J Clin Epidemiol* 2006;59(7):697-703. doi: 10.1016/j.jclinepi.2005.11.010

143. Meursinge Reynders R, Ladu L, Di Girolamo N. Contacting of authors modified crucial outcomes of systematic reviews but was poorly reported, not systematic, and produced conflicting results. *J Clin Epidemiol* 2019;115:64-76. doi: 10.1016/j.jclinepi.2019.07.001

144. Mullan RJ, Flynn DN, Carlberg B, et al. Systematic reviewers commonly contact study authors but do so with limited rigor. *J Clin Epidemiol* 2009;62(2):138-42. doi: 10.1016/j.jclinepi.2008.08.002

145. Higgins JPT TJ, Chandler J, Cumpston M, Li T, Page MJ, Welch VA (editors). Chapter 5: Collecting data. In: Li T HJ, Deeks JJ (editors), ed. Cochrane Handbook for Systematic Reviews of Interventions version 63 (updated February 2022): Cochrane, 2022.

146. Aromataris E, Fernandez R, Godfrey CM, et al. Summarizing systematic reviews: methodological development, conduct and reporting of an umbrella review approach. *Int J Evid Based Healthc* 2015;13(3):132-40. doi: 10.1097/XEB.0000000000000055

147. Tufanaru C, Munn Z, Aromataris E, et al. Chapter 3: Systematic reviews of effectiveness. In: Aromataris E, Munn Z, eds. JBI Manual for Evidence Synthesis: JBI, 2020.

148. Munn Z, Moola S, Lisy K, et al. Methodological guidance for systematic reviews of observational epidemiological studies reporting prevalence and cumulative incidence data. *Int J Evid Based Healthc* 2015;13(3):147-53. doi: 10.1097/XEB.0000000000000054

For peer review only - http://bmjopen.bmj.com/site/about/guidelines.xhtml

149. Moola S, Munn Z, Tufanaru C, et al. Chapter 7: Systematic reviews of etiology and risk. In: Aromataris E, Munn Z, eds. JBI Manual for Evidence Synthesis: JBI, 2020.

150. Maher JM, Markey JC, Ebert-May D. The Other Half of the Story: Effect Size Analysis in Quantitative Research. *CBE Life Sci Educ* 2013;12(3):345-51. doi: 10.1187/cbe.13-04-0082

151. Rosenthal JA. Qualitative Descriptors of Strength of Association and Effect Size. *J Soc Serv Res* 1996;21(4):37-59. doi: 10.1300/j079v21n04_02

152. Cohen J. A power primer. *Psychological bulletin* 1992;112(1):155.

153. Cohen J. Statistical Power Analysis for the Behavioral Sciences. 2nd Edition ed. New York: Routledge 1988.

154. Sonnery-Cottet B, Saithna A, Quelard B, et al. Arthrogenic muscle inhibition after ACL reconstruction: a scoping review of the efficacy of interventions. *Br J Sports Med* 2019;53(5):289-98. doi: 10.1136/bjsports-2017-098401

155. Fedak KM, Bernal A, Capshaw ZA, et al. Applying the Bradford Hill criteria in the 21st century: how data integration has changed causal inference in molecular epidemiology. *Emerg Themes Epidemiol* 2015;12(1) doi: 10.1186/s12982-015-0037-4

156. Hill AB. The Enviroment and Disease: Association or Causation? *Proc R Soc Med* 1965;58(5):295-300.

157. Schünemann H, Hill S, Guyatt G, et al. The GRADE approach and Bradford Hill's criteria for causation. *J Epidemiol Community Health* 2011;65(5):392. doi: 10.1136/jech.2010.119933

For peer review only

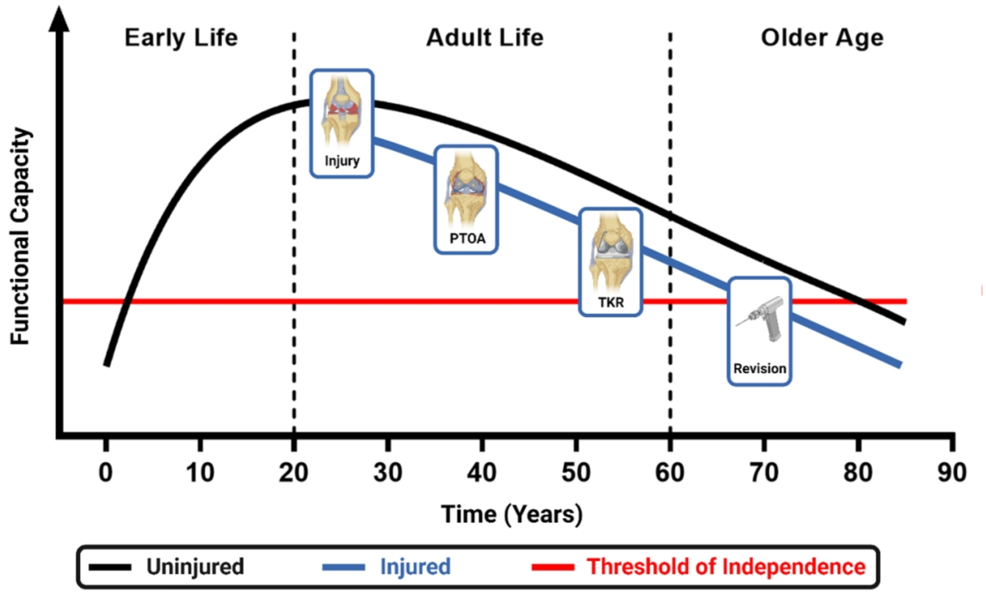

Figure 1. Hypothetical trajectory of injury to lifelong disease burden. Incidence of knee injury leads to post-traumatic osteoarthritis (PTOA),[28-31] which leads to earlier age for total knee replacement (TKR),[34] which increases the risk of revision surgery.[38] This accelerates the loss of knee functional capacity, which could impact the ability to complete self-sustaining tasks.

402x244mm (87 x 87 DPI)

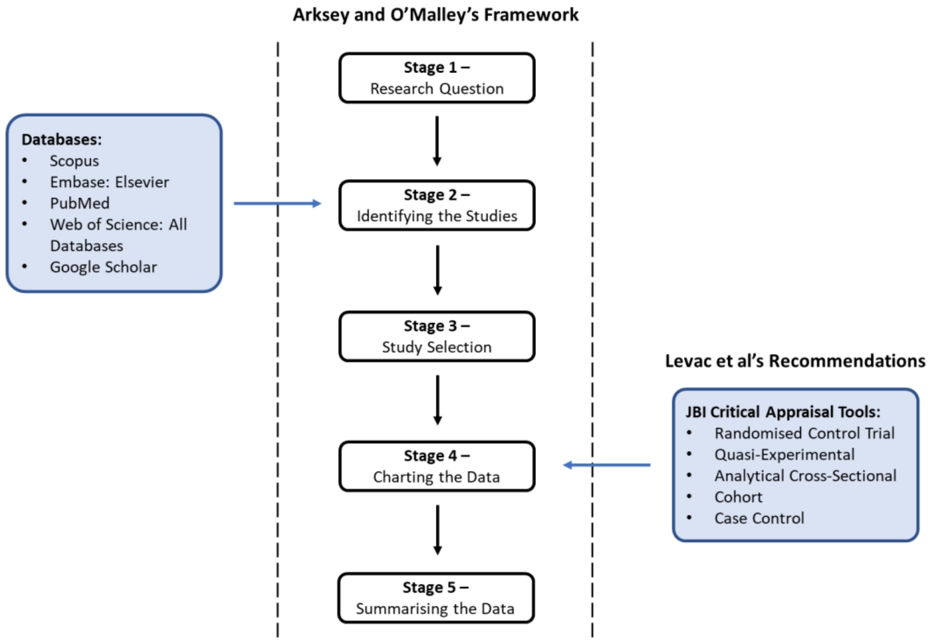

Figure 2. Outline of the study methodology for the present review incorporating Arksey and O'Malley's[102] framework and Levac's[99] recommendations (inclusion of methodological quality appraisal) for conducting a scoping review. JBI denotes the Joanna Briggs Institute.

709x471mm (59 x 59 DPI)

Supplementary Material 1. Preferred Reporting Items for Systematic review and Meta-Analysis Protocols 2015 Checklist[1 2] Adapted for Scoping Reviews[3]

Section and topic	Item No	Checklist item	
**ADMINISTRATIVE INFORMATION**			
Protocol title:			
Identification	1a	Identify the report as a protocol of a scoping review	- Study title<br>- Page 6 of main manuscript
Update	1b	If the protocol is for an update of a previous scoping review, identify as such	Not applicable
Registration	2	If registered, provide the name of the registry (such as Open Science Framework, Figshare or Research Gate) and registration number	- Abstract, page 3 of main manuscript<br>- Methods, page 6 of the main manuscript
Authors:			
Contact	3a	Provide name, institutional affiliation, e-mail address of all protocol authors; provide physical mailing address of corresponding author	Title page, page 1 of main manuscript
Contributions	3b	Describe contributions of protocol authors and identify the guarantor of the review	CRediT statement, page 14 of main manuscript
Amendments	4	If the protocol represents an amendment of a previously completed or published protocol, identify as such and list changes; otherwise, state plan for documenting important protocol amendments	Not applicable
Support:			
Sources	5a	Indicate sources of financial or other support for the review	- Funding statement, page 15 of main manuscript<br>- Acknowledgements, page 15 of main manuscript
Sponsor	5b	Provide name for the review funder and/or sponsor	Funding statement, page 15 of main manuscript
Role of sponsor or funder	5c	Describe roles of funder(s), sponsor(s), and/or institution(s), if any, in developing the protocol	Not applicable

**INTRODUCTION**			
Rationale	6	Describe the rationale for the review in the context of what is already known	Introduction, pages 4-6 of the main manuscript
Objectives	7	Provide an explicit statement of the question(s) the review will address with reference to the inclusion/exclusion criteria	- Introduction, review aims, page 6 of the main manuscript<br>- Methods, framework, stage 1, pages 7-8 of the main manuscript
**METHODS**			
Eligibility criteria	8	Specify the study characteristics (such as PICO/PCC or other, study design, setting, time frame) and report characteristics (such as years considered, language, publication status) to be used as criteria for eligibility for the review	- Table 1., review definitions, page 8-9 of main manuscript<br>- Table 2., review inclusion and exclusion criteria, page 10 of main manuscript
Information sources	9	Describe all intended information sources (such as electronic databases, contact with study authors, trial registers or other grey literature sources) with planned dates of coverage	- Abstract, methods, page 2 of main manuscript<br>- Methods, framework, stage 2, page 9 of main manuscript
Search strategy	10	Present draft of search strategy to be used for at least one electronic database, including planned limits, such that it could be repeated	Supplementary material 2
Study records:			
Data management	11a	Describe the mechanism(s) that will be used to manage records and data throughout the review	- Supplementary material 4, 5, 7, 8<br>- Pages 11-13 of main manuscript
Selection process	11b	State the process that will be used for selecting studies (such as two independent reviewers) through each phase of the review (that is, screening, eligibility, and inclusion)	Pages 11-13 of main manuscript
Data collection process	11c	Describe planned method of extracting data from reports (such as piloting forms, done independently, in duplicate), any processes for obtaining and confirming data from investigators	Pages 11-13 of main manuscript
Data items	12	List and define all variables for which data will be sought (such as PICO/PCC or other items, funding sources), any pre-planned data assumptions and simplifications	Supplementary material 3

For peer review only

Outcomes and prioritization	13	List and define all outcomes for which data will be sought, including prioritization of main and additional outcomes, with rationale (applicable if extracting outcomes)	Supplementary material 3
Risk of bias in individual studies	14	Describe anticipated methods for assessing risk of bias of individual studies, including whether this will be done at the outcome or study level, or both; state how this information will be used in data synthesis	Supplementary material 7
Data synthesis	15a	Describe criteria under which study data will be presented	Methods, framework, stage 4, page 13 of main manuscript
	15b	Describe the planned approach to how, extracted data will be presented (such as figures, tables, evidence gaps maps)	Methods, framework, stage 4 and stage 5, page 13 of main manuscript
	15c	Describe any proposed additional analyses (such as thematic analyses)	Methods, framework, stage 5, pages 13-14 of main manuscript
	15d	If quantitative synthesis is not appropriate, describe the type of summary planned	Methods, framework, stage 5, pages 13-14 of main manuscript
Meta-bias(es)	16	Specify any planned assessment of meta-bias(es) (such as publication bias across studies, selective reporting within studies)	Not applicable
Confidence in cumulative evidence	17	Describe how the strength of the body of evidence will be assessed (such as GRADE)	Methods, framework, stage 5, pages 13-14 of main manuscript

1. Moher D, Shamseer L, Clarke M, et al. Preferred reporting items for systematic review and meta-analysis protocols (PRISMA-P) 2015 statement. *Syst Rev* 2015;4(1):1. doi: 10.1186/2046-4053-4-1
2. Shamseer L, Moher D, Clarke M, et al. Preferred reporting items for systematic review and meta-analysis protocols (PRISMA-P) 2015: elaboration and explanation. *BMJ* 2015;349(jan02 1):g7647-g47. doi: 10.1136/bmj.g7647
3. Peters MDJ, Godfrey C, McInerney P, et al. Best practice guidance and reporting items for the development of scoping review protocols. *JBI Evid Synth* 2022;20(4)

For peer review only

PubMed Search Strategy

The search terms are structured as follows:

Population AND independent variables (e.g., physical activity, adiposity, knee joint loading) AND outcome variables (e.g., osteoarthritis, biomarkers of cartilage or bone metabolism)

Search Terms

(("knee injur*" OR "cruciate ligament injur*" OR "cruciate ligament tear" OR "cruciate ligament damage*" OR "cruciate ligament rupture*" OR “injured cruciate ligament” OR "torn cruciate ligament" OR “damaged cruciate ligament” OR “ruptured cruciate ligament” OR “ACL injury” OR “ACL tear” OR “ACL damage” OR “ACL rupture” OR “injured ACL” OR “torn ACL” OR “damaged ACL” OR “ruptured ACL” OR “PCL injury” OR “PCL tear” OR “PCL damage” OR “PCL rupture” OR “injured PCL” OR “torn PCL” OR “PCL damage” OR “ruptured PCL” OR "cruciate ligament reconstruct*" OR "cruciate ligament surger*" OR “cruciate ligament repair” OR “ACL reconstr*” OR “ACL surger*” OR “PCL reconstr*” OR “PCL surger*” OR "meniscus injur*" OR "meniscal injur*" OR "meniscus tear" OR "meniscal tear" OR "meniscus damage" OR "meniscal damage" OR "meniscus rupture" OR "meniscal rupture" OR “injured menisc*” OR “torn menisc*” OR "meniscus repair" OR “repaired menis*” OR "meniscus surger*" OR Anterior Cruciate Ligament Injuries[MeSH Terms] OR Anterior Cruciate Ligament Reconstruction[MeSH Terms] OR Posterior Cruciate Ligament Reconstruction[MeSH Terms] OR Tibial Meniscus Injuries[MeSH Terms] OR Meniscectomy[MeSH Terms]) AND ("physical activit*" OR "physical inactivit*" OR exercis* OR "rehab*" OR train* OR inflamma* OR "c reactive protein" OR cytokine* OR interleukin OR "reaction force*" OR "contact force*" OR "knee load*" OR "cumulative load*" OR "knee adduction moment" OR "knee abduction moment" OR "knee flexion moment" OR "knee extension moment" OR "contact pressure" OR "ground reaction force*" OR "musculoskeletal model*" OR "inverse dynamics" OR simulat* OR "muscle strength" OR "muscle contract*" OR neurophysiolog* OR neuromuscular OR "body composition" OR "body mass inde*" OR adipos* OR obes* OR fat OR "muscle quality" OR "echo intensity" OR echogenicity OR "inter-muscular adipose" OR "intermuscular adipose" OR "intermuscular fat" OR "intra-muscular adipose" OR "intramuscular adipose" OR "intra-muscular fat" OR "intramuscular fat" OR "muscle attenuation" OR "muscle density" OR "muscle mass" OR "muscle volume" OR "muscle cross-sectional area" OR "muscle thickness" OR "muscle atrophy" OR "bone mineral" OR "bone density" OR Exercise[MeSH Terms] OR Sedentary Behaviour[MeSH Terms] OR Rehabilitation[MeSH Terms] OR Inflammation[MeSH Terms] OR Cytokines[MeSH Terms] OR Interleukins[MeSH Terms] OR Muscle Strength[MeSH Terms] OR Muscle Contraction[MeSH Terms] OR Muscle Weakness[MeSH Terms] OR Body Composition[MeSH Terms] OR Body Mass Index[MeSH Terms] OR Subcutaneous Fat[MeSH Terms] OR Adipose Tissue[MeSH Terms] OR Obesity[MeSH Terms] OR Leptin[MeSH Terms] OR Adiponectin[MeSH Terms] OR Muscular Atrophy[MeSH Terms] OR Bone Density[MeSH Terms]) AND (osteoarthr* OR chondrosis OR “degenerative arthritis” OR "t1 relaxation time" OR t1rho OR "t2 relaxation time" OR t2 OR "delayed gadolinium-enhanced magnetic resonance imaging of cartilage" OR dGEMRIC OR “cartilage volume” OR "cartilage thickness" OR matrix OR MMP OR collagen OR “pro-collagen” OR procollagen OR collagenase OR gelatinase OR proteoglycan OR hyaluronic OR “keratin

For peer review only - http://bmjopen.bmj.com/site/about/guidelines.xhtml

sulphate" OR chondroitin OR pyridinoline OR "alkaline phosphatase bone isoenzyme" OR "bone alkaline phosphatase" OR "tartrate-resistant acid phosphatase 5b" OR osteocalcin OR deoxypyridinoline OR osteophyte* OR "bone remodel*" OR "bone regenerat*" OR 'insulin growth factor 1' OR 'transforming growth factor beta' OR gene* OR allele OR polymorphism OR variant OR expression OR proteom* OR "mass spectrometry" OR stromelysin OR 'a disintegrin and metalloproteinase with thrombospondin motifs' OR ADAMTS OR metaboli* OR biomarker* OR "blood marker" OR "urine marker" OR "synovial fluid marker" OR biochemical OR Osteoarthritis, Knee[MeSH Terms] OR Fibrillar Collagens[MeSH Terms] OR Proteoglycans[MeSH Terms] OR Bone Remodelling[MeSH Terms] OR Bone Regeneration[MeSH Terms] OR Osteogenesis[MeSH Terms] OR Osteophytes[MeSH Terms] OR Biomarkers[MeSH Terms]) NOT (Review[Publication Type] OR Systematic Review[Publication Type] OR Meta Analysis[Publication Type] OR Scoping Review[Publication Type] OR Aged[MeSH Terms] OR Arthroplasty, Replacement, Knee[MeSH Terms] OR (animals[mh] NOT humans[mh])))

Filters Applied: Results by Year: 1970-2023, Species: Human, Language: English

Number of Records: 2,810

For peer review only

Supplementary Material 3. PTOA Disease Outcomes

This list of outcome measures is not exhaustive and will be added to if other relevant outcomes are identified during Stage 2 - Identifying Relevant Studies.

Table 1. Disease Outcome Measures

**Diagnoses of Knee PTOA Disease**	
**Radiographic Diagnostic System**	**Scoring System**
Kellgren and Lawrence (K/L) system[1]	A K/L score of 1 indicates early knee OA and an >1 K/L score indicates knee OA[1]
Osteoarthritis Research Society International (OARSI) Atlas criteria[2]	OARSI Atlas scores for knee OA diagnosis are joint space narrowing ≥ grade 2, or grade 1 joint space narrowing occurred in combination with grade 1 osteophyte[2]
International Knee Documentation Committee (IKDC) System[3]	An IKDC score from C-D indicates reduction in joint space, development of small osteophytes, slight sclerosis, or femoral condyle flattening[3]
Ahlback System[4]	An Ahlback score of >0 indicates development of osteoarthritic features[4]
Fairbank Classification[5]	A Fairbank classification of >0 indicates development of osteoarthritic features[5]
Brandt Grading Scale[6]	
Jager-Wirth Classification[7]	A Jager-Wirth Classification of >0 indicates development of osteoarthritic features[7]
**Magnetic Resonance Imaging Diagnostic System**	**Scoring System**
Magnetic Resonance Imaging Osteoarthritis Knee Score (MOAKS)[8]	For brevity due to the multi-tissue assessment lower scores for each tissue inside the joint indicates degeneration.[8]
Whole Organ Magnetic Resonance Imaging Score (WORMS)[9]	For brevity due to the multi-tissue assessment, lower scores for each tissue inside the joint indicates degeneration.[9]
Boston Leeds OA Knee Score (BLOKS)[10]	For brevity due to the multi-tissue assessment, lower scores for each tissue inside the joint indicates degeneration.[10]

For peer review only

Indicators of Structural Deterioration Preceding PTOA Disease

Measurement Tool	Index
Magnetic resonance imaging (MRI) quantified cartilage thickness and volume[11]	Lower MRI cartilage thickness and volume indicates degenerated cartilage[11 12]
T1rho mapping[12]	Higher signal intensity of T1rho mapping indicates lower collagen and/or proteoglycan content[12 13]
T2 mapping[14]	Higher signal intensity of T2 mapping indicates lower collagen and/or proteoglycan content[12 13]
Delayed contrast-enhanced MRI of cartilage (dGEMRIC)[15]	Higher signal intensity for dGEMRIC is indicative of low glycosaminoglycan (GAG) content[12 15]
Ultrasonography (US) quantified cartilage thickness[16-18]	Lower US cartilage thickness and volume indicates degenerated cartilage[16-18]

Systemic Biomarkers Indicating Structural Deterioration of Knee PTOA Disease

Substrate Biomarkers	Index
Matrix Metalloprotease 3 (MMP-3)[19]	Serum MMP-3 concentration positively associated with knee OA and cartilage volume loss[20-22]
Matrix Metalloprotease 9 (MMP-9)[23]	Serum and synovial fluid MMP-9 concentration positively associated with OA diagnosis[24]
Matrix Metalloprotease 13 (MMP-13) [25-29]	Serum MMP-13 concentration negatively associated with knee cartilage volume and positively associated with knee OA diagnosis[22 30]
A Disintegrin and Metalloproteinase with Thrombospondin Motifs 4 (ADAMTS-4)[31]	Serum ADAMTS-4 concentration positively associated with knee OA diagnosis[32]
Tissue Inhibitor of Metalloproteinase 1 (TIMP-1)[33]	Plasma TIMP-1 concentration positively associated with OA severity[34]
**Collagen Biomarker**	**Index**
N-Propeptide of Collagen IIA (PIIANP)[35]	Serum PIIANP concentration negatively associated with knee osteophytes[24] and knee OA[35 36]
N-Propeptide of Collagen IIB (PIIBNP)[37]	Serum and Plasma concentrations negatively associated with joint space narrowing[38]
C-Telopeptide of Type II Collagen (CTX-II)[39]	Urinary concentration of CTX-II positively associated with knee OA diagnosis and cartilage degeneration[21 40-44]
C-Propeptide of Type II Collagen (CP-II) [45]	Serum concentration of CP-II negatively associated with knee OA diagnosis[45 46]

For peer review only

CTX-II:CP-II[46]	Ratio of urinary concentration of CTX-II to serum concentrations of CP-II positively associated with knee OA[35 46]
Collagen Type II Cleavage Product (C2C)[47]	Serum and urine C2C concentration positively associated with cartilage degeneration and knee joint pain worsening and joint space narrowing[48-50]
Col2-3/4 C-terminal cleavage product of types I and II collagen (C1,2C)[25]	Serum C1,2C concentration positively associated with knee OA diagnosis[50]
Neoepitope of Collagen X (C10C or Col10Neo)[50]	Serum C10C concentration positively associated with knee OA diagnosis[51] and plasma C10C/Col10Neo concentration positively associated with knee OA diagnosis[52]
**Proteoglycan Biomarker**	**Index**
Hyaluronic Acid (HA)[53]	Serum HA concentration positively associated with knee OA diagnosis and joint space narrowing[21 48 54-56]
Chondroitin Sulphate 846 Epitope (CS846)[57]	Serum CS846 concentration positively associated with femur cartilage thickening [58 59]
Aggrecan[60] or Aggrecan Fragments (ARGS)[61 62]	Serum aggrecan concentration at baseline inversely associated with joint space narrowing in medial compartment after 10 years of follow-up[63] and synovial fluid ARGS concentration negatively associated with joint space narrowing[52 64]
Cartilage Oligomeric Matrix Protein (COMP)[53 65]	Serum COMP concentration positively associate with knee OA diagnosis and cartilage degeneration[21 41 43 44 50 63 66-70]
**Bone Formation**	**Index**
Osteocalcin (OC)[71]	Serum increase in OC concentration over a year positively associated with knee OA severity progression over three years[72]
Bone Specific Alkaline Phosphate (BAP) or alkaline phosphatase (ALP)[73]	Serum ALP concentrations positively associated with knee OA severity[74]
Procollagen Type I N-Terminal Propeptide (PINP)[75]	Baseline PINP serum concentration predicts OA progression[76]
**Bone Resorption**	**Index**
Pyridinoline (PYD)[71 77]	Urinary PYD concentration negatively associated with the joint space width and OA severity[34]
Deoxypyridinoline (DPD)[71]	Synovial fluid DPD concentration positively associated with disease severity[78]
N-Telopeptide of Type I Collagen (NTX-I)[79 80]	Urinary and serum NTX-I concentration positively associated with knee OA severity progression[48 81]
C-Telopeptide of Type I Collagen (CTX-I)[79]	Urinary and serum CTX-I concentration positively associated with knee OA severity progression[48]

For peer review only

For peer review only

References

1. Kellgren JH, Lawrence JS. Radiological Assessment of Osteo-Arthrosis. *Ann Rheum Dis* 1957;16(4):494-502. doi: 10.1136/ard.16.4.494
2. Altman RD, Gold GE. Atlas of individual radiographic features in osteoarthritis, revised. *Osteoarthritis Cartilage* 2007;15:A1-A56. doi: 10.1016/j.joca.2006.11.009
3. Hefti E, Müller W, Jakob RP, et al. Evaluation of knee ligament injuries with the IKDC form. *Knee Surgery, Sports Traumatology, Arthroscopy* 1993;1(3-4):226-34. doi: 10.1007/bf01560215
4. Ahlbäck S. Osteoarthrosis of the knee. A radiographic investigation. *Acta Radiol Diagn (Stockh)* 1968:Suppl 277:7-72.
5. Fairbank TJ. KNEE JOINT CHANGES AFTER MENISCECTOMY. *The Journal of Bone and Joint Surgery British volume* 1948;30-B(4):664-70. doi: 10.1302/0301-620X.30B4.664
6. Brandt KD, Fife RS, Braunstein EM, et al. Radiographic grading of the severity of knee osteoarthritis: Relation of the kellgren and lawrence grade to a grade based on joint space narrowing, and correlation with arthroscopic evidence of articular cartilage degeneration. *Arthritis & Rheumatism* 2010;34(11):1381-86. doi: 10.1002/art.1780341106
7. Scheller G, Sobau C, Bülow JU. Arthroscopic partial lateral meniscectomy in an otherwise normal knee: Clinical, functional, and radiographic results of a long-term follow-up study. *Arthroscopy* 2001;17(9):946-52. doi: 10.1053/jars.2001.28952
8. Hunter DJ, Guermazi A, Lo GH, et al. Evolution of semi-quantitative whole joint assessment of knee OA: MOAKS (MRI Osteoarthritis Knee Score). *Osteoarthritis Cartilage* 2011;19(8):990-1002. doi: 10.1016/j.joca.2011.05.004 [published Online First: 2011/06/08]
9. Peterfy CG, Guermazi A, Zaim S, et al. Whole-Organ Magnetic Resonance Imaging Score (WORMS) of the knee in osteoarthritis. *Osteoarthritis Cartilage* 2004;12(3):177-90. doi: 10.1016/j.joca.2003.11.003
10. Hunter DJ, Lo GH, Gale D, et al. The reliability of a new scoring system for knee osteoarthritis MRI and the validity of bone marrow lesion assessment: BLOKS (Boston Leeds Osteoarthritis Knee Score). *Ann Rheum Dis* 2008;67(2):206-11. doi: 10.1136/ard.2006.066183 [published Online First: 2007/05/03]
11. Eckstein F, Cicuttini F, Raynauld JP, et al. Magnetic resonance imaging (MRI) of articular cartilage in knee osteoarthritis (OA): morphological assessment. *Osteoarthritis Cartilage* 2006;14:46-75. doi: 10.1016/j.joca.2006.02.026
12. Blumenkrantz G, Majumdar S. Quantitative magnetic resonance imaging of articular cartilage in osteoarthritis. *Eur Cell Mater* 2007; 13. http://europepmc.org/abstract/MED/17506024

https://doi.org/10.22203/ecm.v013a08 (accessed 2007/05//).
13. Atkinson HF, Birmingham TB, Moyer RF, et al. MRI T2 and T1ρ relaxation in patients at risk for knee osteoarthritis: a systematic review and meta-analysis. *BMC Musculoskelet Disord* 2019;20(1):1-18.
14. Dunn TC, Lu Y, Jin H, et al. T2 relaxation time of cartilage at MR imaging: comparison with severity of knee osteoarthritis. *Radiology* 2004;232(2):592-8. doi: 10.1148/radiol.2322030976 [published Online First: 20040623]
15. Bashir A, Gray ML, Boutin RD, et al. Glycosaminoglycan in articular cartilage: in vivo assessment with delayed Gd(DTPA)(2-)-enhanced MR imaging. *Radiology* 1997;205(2):551-8. doi: 10.1148/radiology.205.2.9356644
16. Naredo E, Acebes C, Moller I, et al. Ultrasound validity in the measurement of knee cartilage thickness. *Ann Rheum Dis* 2009;68(8):1322-7. doi: 10.1136/ard.2008.090738 [published Online First: 2008/08/08]
17. Abraham AM, Goff I, Pearce MS, et al. Reliability and validity of ultrasound imaging of features of knee osteoarthritis in the community. *BMC Musculoskelet Disord* 2011;12(1):70. doi: 10.1186/1471-2474-12-70

For peer review only - http://bmjopen.bmj.com/site/about/guidelines.xhtml

18. Schmitz RJ, Wang HM, Polprasert DR, et al. Evaluation of knee cartilage thickness: A comparison between ultrasound and magnetic resonance imaging methods. *The Knee* 2017;24(2):217-23. doi: 10.1016/j.knee.2016.10.004 [published Online First: 2016/12/05]
19. Okada Y, Shinmei M, Tanaka O, et al. Localization of matrix metalloproteinase 3 (stromelysin) in osteoarthritic cartilage and synovium. *Lab Invest* 1992;66(6):680-90. [published Online First: 1992/06/01]
20. Pelletier JP, Raynauld JP, Caron J, et al. Decrease in serum level of matrix metalloproteinases is predictive of the disease-modifying effect of osteoarthritis drugs assessed by quantitative MRI in patients with knee osteoarthritis. *Ann Rheum Dis* 2010;69(12):2095-101. doi: 10.1136/ard.2009.122002 [published Online First: 2010/06/24]
21. Joseph GB, Nevitt MC, McCulloch CE, et al. Associations between molecular biomarkers and MR-based cartilage composition and knee joint morphology: data from the Osteoarthritis Initiative. *Osteoarthritis Cartilage* 2018;26(8):1070-77. doi: 10.1016/j.joca.2018.04.019 [published Online First: 2018/05/29]
22. Pengas I, Eldridge S, Assiotis A, et al. MMP-3 in the peripheral serum as a biomarker of knee osteoarthritis, 40 years after open total knee meniscectomy. *J Exp Orthop* 2018;5(1):1-8.
23. Fosang AJ, Neame PJ, Last K, et al. The interglobular domain of cartilage aggrecan is cleaved by PUMP, gelatinases, and cathepsin B. *Journal of Biological Chemistry* 1992;267(27):19470-74. doi: https://doi.org/10.1016/S0021-9258(18)41799-1
24. Zeng GQ, Chen AB, Li W, et al. High MMP-1, MMP-2, and MMP-9 protein levels in osteoarthritis. *Genetics and Molecular Research* 2015;14(4):14811-22. doi: 10.4238/2015.november.18.46
25. Billinghurst RC, Dahlberg L, Ionescu M, et al. Enhanced cleavage of type II collagen by collagenases in osteoarthritic articular cartilage. *J Clin Invest* 1997;99(7):1534-45. doi: 10.1172/JCI119316 [published Online First: 1997/04/01]
26. Neuhold LA, Killar L, Zhao W, et al. Postnatal expression in hyaline cartilage of constitutively active human collagenase-3 (MMP-13) induces osteoarthritis in mice. *J Clin Invest* 2001;107(1):35-44. doi: 10.1172/JCI10564 [published Online First: 2001/01/03]
27. Shlopov BV, Lie WR, Mainardi CL, et al. Osteoarthritic lesions: involvement of three different collagenases. *Arthritis Rheum* 1997;40(11):2065-74. doi: 10.1002/art.1780401120 [published Online First: 1997/11/19]
28. Wang M, Sampson ER, Jin H, et al. MMP13 is a critical target gene during the progression of osteoarthritis. *Arthritis Res Ther* 2013;15(1):R5. doi: 10.1186/ar4133 [published Online First: 2013/01/10]
29. Hu Q, Ecker M. Overview of MMP-13 as a promising target for the treatment of osteoarthritis. *Int J Mol Sci* 2021;22(4):1742. doi: 10.3390/ijms22041742
30. Ruan G, Xu J, Wang K, et al. Associations between knee structural measures, circulating inflammatory factors and MMP13 in patients with knee osteoarthritis. *Osteoarthritis Cartilage* 2018;26(8):1063-69. doi: 10.1016/j.joca.2018.05.003 [published Online First: 2018/05/14]
31. Tortorella M, Pratta M, Liu R-Q, et al. The Thrombospondin Motif of Aggrecanase-1 (ADAMTS-4) Is Critical for Aggrecan Substrate Recognition and Cleavage. *Journal of Biological Chemistry* 2000;275(33):25791-97. doi: 10.1074/jbc.m001065200
32. Li W, Du C, Wang H, et al. Increased serum ADAMTS-4 in knee osteoarthritis: a potential indicator for the diagnosis of osteoarthritis in early stages. *Genetics and Molecular Research* 2014;13(4):9642-49. doi: 10.4238/2014.november.14.9
33. Woolley DE, Roberts DR, Evanson JM. Inhibition of human collagenase activity by a small molecular weight serum protein. *Biochem Biophys Res Commun* 1975;66(2):747-54. doi: 10.1016/0006-291x(75)90573-2
34. Takahashi M, Naito K, Abe M, et al. *Arthritis Research & Therapy* 2004;6(3):R208. doi: 10.1186/ar1166

For peer review only

35. Rousseau JC, Sandell LJ, Delmas PD, et al. Development and clinical application in arthritis of a new immunoassay for serum type IIA procollagen NH2 propeptide. *Methods Mol Med* 2004;101:25-37. doi: 10.1385/1-59259-821-8:025 [published Online First: 2004/08/10]
36. Daghestani HN, Jordan JM, Renner JB, et al. Serum N-propeptide of collagen IIA (PIIANP) as a marker of radiographic osteoarthritis burden. *PLoS One* 2017;12(12):e0190251. doi: 10.1371/journal.pone.0190251
37. Luo Y, He Y, Reker D, et al. A Novel High Sensitivity Type II Collagen Blood-Based Biomarker, PRO-C2, for Assessment of Cartilage Formation. *International Journal of Molecular Sciences* 2018;19(11):3485. doi: 10.3390/ijms19113485
38. Luo Y, Samuels J, Krasnokutsky S, et al. A low cartilage formation and repair endotype predicts radiographic progression of symptomatic knee osteoarthritis. *J Orthop Traumatol* 2021;22(1) doi: 10.1186/s10195-021-00572-0
39. Christgau S, Garnero P, Fledelius C, et al. Collagen type II C-telopeptide fragments as an index of cartilage degradation. *Bone* 2001;29(3):209-15. doi: 10.1016/s8756-3282(01)00504-x [published Online First: 2001/09/15]
40. Cheng H, Hao B, Sun J, et al. C-terminal cross-linked telopeptides of type II collagen as biomarker for radiological knee osteoarthritis: a meta-analysis. *Cartilage* 2020;11(4):512-20. doi: 10.1177/1947603518798884 [published Online First: 2018/09/18]
41. Valdes AM, Meulenbelt I, Chassaing E, et al. Large scale meta-analysis of urinary C-terminal telopeptide, serum cartilage oligomeric protein and matrix metalloprotease degraded type II collagen and their role in prevalence, incidence and progression of osteoarthritis. *Osteoarthritis Cartilage* 2014;22(5):683-9. doi: 10.1016/j.joca.2014.02.007 [published Online First: 2014/03/01]
42. Dam EB, Byrjalsen I, Karsdal MA, et al. Increased urinary excretion of C-telopeptides of type II collagen (CTX-II) predicts cartilage loss over 21 months by MRI. *Osteoarthritis Cartilage* 2009;17(3):384-9. doi: 10.1016/j.joca.2008.07.009 [published Online First: 2008/09/05]
43. Sowers MF, Karvonen-Gutierrez CA, Yosef M, et al. Longitudinal changes of serum COMP and urinary CTX-II predict X-ray defined knee osteoarthritis severity and stiffness in women. *Osteoarthritis Cartilage* 2009;17(12):1609-14. doi: 10.1016/j.joca.2009.06.001 [published Online First: 2009/07/01]
44. Hao HQ, Zhang JF, He QQ, et al. Cartilage oligomeric matrix protein, C-terminal cross-linking telopeptide of type II collagen, and matrix metalloproteinase-3 as biomarkers for knee and hip osteoarthritis (OA) diagnosis: a systematic review and meta-analysis. *Osteoarthritis Cartilage* 2019;27(5):726-36. doi: 10.1016/j.joca.2018.10.009 [published Online First: 2018/11/06]
45. Nelson F, Dahlberg L, Laverty S, et al. Evidence for altered synthesis of type II collagen in patients with osteoarthritis. *J Clin Invest* 1998;102(12):2115-25. doi: 10.1172/JCI4853 [published Online First: 1998/12/17]
46. Cibere J, Zhang H, Garnero P, et al. Association of biomarkers with pre-radiographically defined and radiographically defined knee osteoarthritis in a population-based study. *Arthritis Rheum* 2009;60(5):1372-80. doi: 10.1002/art.24473 [published Online First: 2009/05/01]
47. Poole AR, Ionescu M, Fitzcharles MA, et al. The assessment of cartilage degradation in vivo: development of an immunoassay for the measurement in body fluids of type II collagen cleaved by collagenases. *J Immunol Methods* 2004;294(1-2):145-53. doi: 10.1016/j.jim.2004.09.005 [published Online First: 2005/01/11]
48. Kraus VB, Collins JE, Hargrove D, et al. Predictive validity of biochemical biomarkers in knee osteoarthritis: data from the FNIH OA Biomarkers Consortium. *Ann Rheum Dis* 2017;76(1):186-95. doi: 10.1136/annrheumdis-2016-209252 [published Online First: 2016/06/15]
49. King KB, Lindsey CT, Dunn TC, et al. A study of the relationship between molecular biomarkers of joint degeneration and the magnetic resonance-measured characteristics of cartilage in 16

For peer review only

symptomatic knees. *Magn Reson Imaging* 2004;22(8):1117-23. doi: 10.1016/j.mri.2004.08.001
50. Kong SY, Stabler TV, Criscione LG, et al. Diurnal variation of serum and urine biomarkers in patients with radiographic knee osteoarthritis. *Arthritis Rheum* 2006;54(8):2496-504. doi: 10.1002/art.21977 [published Online First: 2006/07/27]
51. He Y, Siebuhr AS, Brandt-Hansen NU, et al. Type X collagen levels are elevated in serum from human osteoarthritis patients and associated with biomarkers of cartilage degradation and inflammation. *BMC Musculoskelet Disord* 2014;15(1):309. doi: 10.1186/1471-2474-15-309
52. He Y, Manon-Jensen T, Arendt-Nielsen L, et al. Potential diagnostic value of a type X collagen neo-epitope biomarker for knee osteoarthritis. *Osteoarthritis Cartilage* 2019;27(4):611-20. doi: 10.1016/j.joca.2019.01.001
53. Laurent TC, Laurent UB, Fraser JRE. Serum hyaluronan as a disease marker. *Ann Med* 1996;28(3):241-53.
54. Georges C, Vigneron H, Ayral X, et al. Serum biologic markers as predictors of disease progression in osteoarthritis of the knee. *Arthritis Rheum* 1997;40(3):590-1. doi: 10.1002/art.1780400333 [published Online First: 1997/03/01]
55. Elliott AL, Kraus VB, Luta G, et al. Serum hyaluronan levels and radiographic knee and hip osteoarthritis in African Americans and Caucasians in the Johnston County Osteoarthritis Project. *Arthritis Rheum* 2005;52(1):105-11. doi: 10.1002/art.20724 [published Online First: 2005/01/11]
56. Sharif M, George E, Shepstone L, et al. Serum hyaluronic acid level as a predictor of disease progression in osteoarthritis of the knee. *Arthritis Rheum* 1995;38(6):760-7. doi: 10.1002/art.1780380608 [published Online First: 1995/06/01]
57. Rizkalla G, Reiner A, Bogoch E, et al. Studies of the articular cartilage proteoglycan aggrecan in health and osteoarthritis. Evidence for molecular heterogeneity and extensive molecular changes in disease. *J Clin Invest* 1992;90(6):2268-77. doi: 10.1172/JCI116113 [published Online First: 1992/12/01]
58. Chu CR, Sheth S, Erhart-Hledik JC, et al. Mechanically stimulated biomarkers signal cartilage changes over 5 years consistent with disease progression in medial knee osteoarthritis patients. *J Orthop Res* 2018;36(3):891-97. doi: 10.1002/jor.23720 [published Online First: 2017/09/02]
59. Mazzuca SA, Poole AR, Brandt KD, et al. Associations between joint space narrowing and molecular markers of collagen and proteoglycan turnover in patients with knee osteoarthritis. *J Rheumatol* 2006;33(6):1147-51.
60. Lohmander SL, Neame PJ, Sandy JD. The structure of aggrecan fragments in human synovial fluid. evidence that aggrecanase mediates cartilage degradation in inflammatory joint disease, joint injury, and osteoarthritis. *Arthritis Rheum* 1993;36(9):1214-22. doi: https://doi.org/10.1002/art.1780360906
61. Larsson S, Lohmander LS, Struglics A. Synovial fluid level of aggrecan ARGS fragments is a more sensitive marker of joint disease than glycosaminoglycan or aggrecan levels: a cross-sectional study. *Arthritis Res Ther* 2009;11(3):R92. doi: 10.1186/ar2735
62. Larsson S, Lohmander LS, Struglics A. An ARGS-aggrecan assay for analysis in blood and synovial fluid. *Osteoarthritis Cartilage* 2014;22(2):242-9. doi: 10.1016/j.joca.2013.12.010 [published Online First: 2013/12/24]
63. Blumenfeld O, Williams FMK, Hart DJ, et al. Association between cartilage and bone biomarkers and incidence of radiographic knee osteoarthritis (RKOA) in UK females: a prospective study. *Osteoarthritis Cartilage* 2013;21(7):923-29. doi: 10.1016/j.joca.2013.04.009
64. Larsson S, Englund M, Struglics A, et al. Association between synovial fluid levels of aggrecan ARGS fragments and radiographic progression in knee osteoarthritis. *Arthritis Res Ther* 2010;12(6):R230. doi: 10.1186/ar3217

65. Saxne T, Heinegård D. Cartilage oligomeric matrix protein: a novel marker of cartilage turnover detectable in synovial fluid and blood. *Rheumatology* 1992;31(9):583-91. doi: 10.1093/rheumatology/31.9.583
66. Bi X. Correlation of serum cartilage oligomeric matrix protein with knee osteoarthritis diagnosis: a meta-analysis. *J Orthop Surg Res* 2018;13(1):262. doi: 10.1186/s13018-018-0959-y [published Online First: 2018/10/21]
67. Vilim V, Olejarova M, Machacek S, et al. Serum levels of cartilage oligomeric matrix protein (COMP) correlate with radiographic progression of knee osteoarthritis. *Osteoarthritis Cartilage* 2002;10(9):707-13. doi: 10.1053/joca.2002.0819 [published Online First: 2002/08/31]
68. Verma P, Dalal K. Serum cartilage oligomeric matrix protein (COMP) in knee osteoarthritis: A novel diagnostic and prognostic biomarker. *J Orthop Res* 2013;31(7):999-1006. doi: 10.1002/jor.22324
69. Hoch JM, Mattacola CG, Medina McKeon JM, et al. Serum cartilage oligomeric matrix protein (sCOMP) is elevated in patients with knee osteoarthritis: a systematic review and meta-analysis. *Osteoarthritis Cartilage* 2011;19(12):1396-404. doi: 10.1016/j.joca.2011.09.005 [published Online First: 2011/10/18]
70. Clark AG, Jordan JM, Vilim V, et al. Serum cartilage oligomeric matrix protein reflects osteoarthritis presence and severity: The Johnston county osteoarthritis project. *Arthritis Rheum* 1999;42(11):2356-64. doi: 10.1002/1529-0131(199911)42:11<2356::aid-anr14>3.0.co;2-r
71. Delmas PD. Biochemical markers of bone turnover. *J Bone Miner Res* 1993;8 Suppl 2:S549-55. doi: 10.1002/jbmr.5650081323
72. Bruyere O, Collette JH, Ethgen O, et al. Biochemical markers of bone and cartilage remodeling in prediction of longterm progression of knee osteoarthritis. *J Rheumatol* 2003;30(5):1043-50.
73. Gomez B, Jr., Ardakani S, Ju J, et al. Monoclonal antibody assay for measuring bone-specific alkaline phosphatase activity in serum. *Clinical Chemistry* 1995;41(11):1560-66. doi: 10.1093/clinchem/41.11.1560
74. Park H-M, Lee J-H, Lee Y-J. Positive Association of Serum Alkaline Phosphatase Level with Severe Knee Osteoarthritis: A Nationwide Population-Based Study. *Diagnostics* 2020;10(12):1016. doi: 10.3390/diagnostics10121016
75. Orum O, Hansen M, Jensen CH, et al. Procollagen type I N-terminal propeptide (PINP) as an indicator of type I collagen metabolism: ELISA development, reference interval, and hypovitaminosis D induced hyperparathyroidism. *Bone* 1996;19(2):157-63. doi: 10.1016/8756-3282(96)00165-2
76. Kumm J, Tamm A, Lintrop M, et al. Diagnostic and prognostic value of bone biomarkers in progressive knee osteoarthritis: a 6-year follow-up study in middle-aged subjects. *Osteoarthritis and cartilage* 2013;21(6):815-22. doi: 10.1016/j.joca.2013.03.008
77. Delmas PD, Schlemmer A, Gineyts E, et al. Urinary excretion of pyridinoline crosslinks correlates with bone turnover measured on iliac crest biopsy in patients with vertebral osteoporosis. *J Bone Miner Res* 1991;6(6):639-44. doi: 10.1002/jbmr.5650060615
78. Schmidt-Rohlfing B, Thomsen M, Niedhart C, et al. Correlation of bone and cartilage markers in the synovial fluid with the degree of osteoarthritis. *Rheumatology International* 2002;21(5):193-99. doi: 10.1007/s00296-001-0170-y
79. Rosen HN, Moses AC, Garber J, et al. Serum CTX: A New Marker of Bone Resorption That Shows Treatment Effect More Often Than Other Markers Because of Low Coefficient of Variability and Large Changes with Bisphosphonate Therapy. *Calcified Tissue International* 2000;66(2):100-03. doi: 10.1007/pl00005830
80. Rosen HN, Dresner-Pollak R, Moses AC, et al. Specificity of urinary excretion of cross-linked N-telopeptides of type I collagen as a marker of bone turnover. *Calcified Tissue International* 1994;54(1):26-29. doi: 10.1007/bf00316285

For peer review only

81. Bettica P, Cline G, Hart DJ, et al. Evidence for increased bone resorption in patients with progressive knee osteoarthritis: Longitudinal results from the Chingford study. *Arthritis & Rheumatism* 2002;46(12):3178-84. doi: 10.1002/art.10630

For peer review only

Screening Tool: Title/Abstract

Codebook

Data Dictionary Codebook

2022-12-22 21:32:53

Collapse all instruments

#	Variable / Field Name	Field Label<br>*Field Note*	Field Attributes (Field Type, Validation, Choices, Calculations, etc.)
Instrument: **Screening Tool** (screening_tool)			Collapse
1	record_id	Record ID	text
2	instructions	Yes - There is explicit evidence in the title or abstract of the research including this.	descriptive
3	maybe	Maybe - It is unclear whether there is evidence of this in the title/abstract. It may or may not be relevant but I feel this needs further investigation.	descriptive
4	no	No - There is explicit evidence in the title or abstract of the research not including this.	descriptive
5	reviewer_screening	Section Header: *Reviewer Details*<br>Reviewer	radio, Required<br>1 Karl Morgan<br>2 James Cowburn<br>3 Mathew Farrow<br>4 Josh Carter
6	reviewer_date_screening	Date<br>*Just press 'Today'*	text (date_dmy), Required
7	record_number_screening	Record number	text (number), Required
8	review_no_meta	Section Header: *1. Study Information*<br>Is this a review, meta-analysis, book, protocol or commentary?	checkbox<br>1 review_no_meta___1 No<br>2 review_no_meta___2 Maybe<br>3 review_no_meta___3 Yes
9	animal<br>Show the field ONLY if:<br>[review_no_meta(3)] <> 1	Is the study an animal model?	checkbox<br>1 animal___1 No<br>2 animal___2 Maybe<br>3 animal___3 Yes
10	in_silico<br>Show the field ONLY if:<br>[review_no_meta(3)] <> 1 and [animal(3)] <> 1	Is the study purely in silico?	checkbox<br>1 in_silico___1 No<br>2 in_silico___2 Maybe<br>3 in_silico___3 Yes
11	in_vitro<br>Show the field ONLY if:<br>[review_no_meta(3)] <> 1 and [animal(3)] <> 1 and [in_silico (3)] <> 1	Is the study purely in vitro (i.e. no human participants)?	checkbox<br>1 in_vitro___1 No<br>2 in_vitro___2 Maybe<br>3 in_vitro___3 Yes
12	cadavers<br>Show the field ONLY if:<br>[review_no_meta(3)] <> 1 and [animal(3)] <> 1 and [in_silico (3)] <> 1 and [in_vitro(3)] <> 1	Is the study only working with cadavers?	checkbox<br>1 cadavers___1 No<br>2 cadavers___2 Maybe<br>3 cadavers___3 Yes
13	knee_oa<br>Show the field ONLY if:<br>[review_no_meta(3)] <> 1 and [animal(3)] <> 1 and [in_silico (3)] <> 1 and [in_vitro(3)] <> 1 and [cadavers(3)] <> 1	Do the participants already have knee osteoarthritis (e.g., a cross-sectional study looking at the effects of exercise in people who already have knee OA disease) or another type of arthritis/disease?	checkbox<br>1 knee_oa___1 No<br>2 knee_oa___2 Maybe<br>3 knee_oa___3 Yes

	14	knee_injury<br>Show the field ONLY if:<br>[review_no_meta(3)] <> 1 and [animal(3)] <> 1 and [in_silico(3)] <> 1 and [in_vitro(3)] <> 1 and [cadavers(3)] <> 1 and [knee_oa(3)] <> 1	Section Header: *2. Population*<br>Could this study involve those at risk of knee post-traumatic osteoarthritis (PTOA) such as after a knee injury? Cruciate ligament injuryCruciate ligament reconstructionMeniscus injuryMeniscectomy<br><br>This does NOT include:<br>Total knee replacementKnee arthroplastyosteotomy<br>*Please select one option*	checkbox, Required<br>1 | knee_injury___1 | No<br>2 | knee_injury___2 | Maybe<br>3 | knee_injury___3 | Yes
	15	age<br>Show the field ONLY if:<br>[review_no_meta(3)] <> 1 and [animal(3)] <> 1 and [in_silico(3)] <> 1 and [in_vitro(3)] <> 1 and [cadavers(3)] <> 1 and [knee_oa(3)] <> 1 and [knee_injury(1)] <> 1	Could the study participants be aged 18-40?<br>At least 50% of the population must be within 18-40 years old.<br><br>If the study is longitudinal, use the baseline age.<br>*Please select one option.*	checkbox, Required<br>1 | age___1 | No<br>2 | age___2 | Maybe<br>3 | age___3 | Yes
	16	independent_variable<br>Show the field ONLY if:<br>[review_no_meta(3)] <> 1 and [animal(3)] <> 1 and [in_silico(3)] <> 1 and [in_vitro(3)] <> 1 and [cadavers(3)] <> 1 and [knee_oa(3)] <> 1 and [knee_injury(1)] <> 1 and [age(1)] <> 1	Section Header: *3. Independent Variable*<br>Could the study have an independent variable which measures:<br>Physical activity (including Tegner)ExerciseSystemic inflammation (e.g., IL-6 concentrations)Knee joint load (moments or MSK modelling)Adipose tissueMuscle strengthMuscle sizeIntra-muscular adipose tissueBone mineral content or density<br>Or may the study have an intervention involving:<br><br>Physical activity or exerciseManipulation of joint loadWeight lossDisuse or bedrest<br>*Please select one option*	checkbox, Required<br>1 | independent_variable___1 | No<br>2 | independent_variable___2 | Maybe<br>3 | independent_variable___3 | Yes
	17	diagnosis_header<br>Show the field ONLY if:<br>[review_no_meta(3)] <> 1 and [animal(3)] <> 1 and [in_silico(3)] <> 1 and [in_vitro(3)] <> 1 and [cadavers(3)] <> 1 and [knee_oa(3)] <> 1 and [knee_injury(1)] <> 1 and [age(1)] <> 1 and [independent_variable(1)] <> 1	Section Header: *4. Outcome Variable*<br>Knee Osteoarthritis (OA) Diagnosis	descriptive
	18	disease_diagnoses<br>Show the field ONLY if:<br>[review_no_meta(3)] <> 1 and [animal(3)] <> 1 and [in_silico(3)] <> 1 and [in_vitro(3)] <> 1 and [cadavers(3)] <> 1 and [knee_oa(3)] <> 1 and [knee_injury(1)] <> 1 and [age(1)] <> 1 and [independent_variable(1)] <> 1	Could the study investigate knee OA diagnosis including the following scales:<br>Kellgren and Lawrence (K/L)Osteoarthritis Research Society International (OARSI) AtlasInternational Knee Documentation Committee (IKDC) SystemAhlbäck SystemFairbank ClassificationBrandt Grading ScaleJäger-Wirth ClassificationMerchant SystemMRI Osteoarthritis Knee Score (MOAKS)Whole Organ Magnetic Resonance Imaging Score (WORMS)Boston Leeds OA Knee Score (BLOKS)<br><br>P.s if there is another system mentioned that your are unsure of then please send me a message.<br>*Please select one option*	checkbox, Required<br>1 | disease_diagnoses___1 | No<br>2 | disease_diagnoses___2 | Maybe<br>3 | disease_diagnoses___3 | Yes
	19	imaging_indicator_header<br>Show the field ONLY if:<br>[review_no_meta(3)] <> 1 and [animal(3)] <> 1 and [in_silico(3)] <> 1 and [in_vitro(3)] <> 1 and [cadavers(3)] <> 1 and [knee_oa(3)] <> 1 and [knee_injury(1)] <> 1 and [age(1)] <> 1 and [independent_variable(1)] <> 1	Medical Imaging Indicator	descriptive
	20	medical_imaging_indicators<br>Show the field ONLY if:<br>[review_no_meta(3)] <> 1 and [animal(3)] <> 1 and [in_silico(3)] <> 1 and [in_vitro(3)] <> 1 and [cadavers(3)] <> 1 and [knee_oa(3)] <> 1 and [knee_injury(1)] <> 1 and [age(1)] <> 1 and [independent_variable(1)] <> 1	Could the study investigate medical imaging indicators of tissue health including the following techniques:<br>MRI quantified cartilage thickness and volumeT1rho mappingT2 mappingDelayed contrast-enhanced MRI of cartilage (dGEMRIC)Ultrasound quantified cartilage thickness<br>*Please select one option*	checkbox, Required<br>1 | medical_imaging_indicators___1 | No<br>2 | medical_imaging_indicators___2 | Maybe<br>3 | medical_imaging_indicators___3 | Yes

	21	biomarkers_header<br>Show the field ONLY if: [review_no_meta(3)] <> 1 and [animal(3)] <> 1 and [in_silico(3)] <> 1 and [in_vitro(3)] <> 1 and [cadavers(3)] <> 1 and [knee_oa(3)] <> 1 and [knee_injury(1)] <> 1 and [age(1)] <> 1 and [independent_variable(1)] <> 1	Biomarkers	descriptive
	22	biomarkers<br>Show the field ONLY if: [review_no_meta(3)] <> 1 and [animal(3)] <> 1 and [in_silico(3)] <> 1 and [in_vitro(3)] <> 1 and [cadavers(3)] <> 1 and [knee_oa(3)] <> 1 and [knee_injury(1)] <> 1 and [age(1)] <> 1 and [independent_variable(1)] <> 1	Could the study investigate biomarkers including the following:<br>Cartilage synthesisC-propeptide of type II collagen (CP-II)Chondroitin sulphate 846 epitope (CS846)N-Propeptide of Collagen IIA (PIIANP)N-Propeptide of Collagen IIB (PIIBNP)<br>Cartilage degradationMatrix metalloproteases (e.g., MMP-3, MMP-9, MMP-13)A disintegrin and metalloproteinase with thrombospondin motifs (e.g., ADAMTS-4, ADAMTS-5)Collagen type II cleavage product (C2C)Col2-3/4 C-terminal cleavage product of types I and II collagen (C1,2C)Neoepitope of collagen X (C10C or Col10Neo)Hyaluronic acid (HA) Aggrecan or aggrecan fragments (ARGS)Keratin sulphate (KS)Cartilage oligomeric matrix protein (COMP)<br>Bone formationOsteocalcin (OC)Bone alkaline phosphatase (BAP)Procollagen type I N-terminal propeptide (PINP)Procollagen type I C-terminal propeptide (PICP)<br>Bone resorptionPyridinoline (PYD)Deoxypyridinoline (DPD)Tartrate-resistant acid phosphatase 5b (TRAP-5)N-telopeptide if type I collagen (NTX-I)C-telopeptide of type I collagen (CTX-I)Insulin-like growth factor 1 (IGF-1)Transforming growth factor beta (TGFB)<br>*Please select one option*	checkbox, Required<br>1 | biomarkers___1 | No<br>2 | biomarkers___2 | Maybe<br>3 | biomarkers___3 | Yes
	23	all_yes_dec<br>Show the field ONLY if: [knee_injury(3)]= 1 and [age(3)] = 1 and [independent_variable(3)] = 1 and [review_no_meta(3)] <> 1 and [animal(3)] <> 1 and [in_silico(3)] <> 1 and [cadavers(3)] <> 1 or [disease_diagnoses(3)] = 1 or [medical_imaging_indicators(3)] = 1 or [biomarkers(3)] = 1	Section Header: *Decision*<br>Select 'Yes' in Covidence	descriptive
	24	all_maybe_dec<br>Show the field ONLY if: [knee_injury(2)]= 1 and [age(2)] = 1 and [independent_variable(2)] = 1 and [review_no_meta(1)] <> 1 and [animal(1)] <> 1 and [in_silico(1)] <> 1 and [cadavers(1)] <> 1	Select 'Maybe' in Covidence	descriptive
	25	knee_inj_maybe<br>Show the field ONLY if: [knee_injury(2)]= 1 and [age(1)] = 1 and [independent_variable(1)] = 1 and [disease(1)] = 1	Select 'Maybe' in Covidence	descriptive
	26	age_maybe<br>Show the field ONLY if: [knee_injury(1)]= 1 and [age(2)] = 1 and [independent_variable(1)] = 1 and [disease(1)] = 1 and [review_no_meta(1)] <> 1 and [animal(1)] <> 1 and [in_silico(1)] <> 1 and [cadavers(1)] <> 1	Select 'Maybe' in Covidence	descriptive

	27	iv_maybe<br>Show the field ONLY if: [knee_injury(1)]= 1 and [age(1)] = 1 and [independent_variable(2)] = 1 and [disease(1)] = 1 and [review_no_meta(1)] <> 1 and [animal(1)] <> 1 and [in_silico(1)] <> 1 and [cadavers(1)] <> 1	Select 'Maybe' in Covidence	descriptive
	28	ov_maybe<br>Show the field ONLY if: [knee_injury(1)]= 1 and [age(1)] = 1 and [independent_variable(1)] = 1 and [disease(2)] = 1 and [review_no_meta(1)] <> 1 and [animal(1)] <> 1 and [in_silico(1)] <> 1 and [cadavers(1)] <> 1	Select 'Maybe' in Covidence	descriptive
	29	age_iv_ov_maybe<br>Show the field ONLY if: [knee_injury(1)]= 1 and [age(2)] = 1 and [independent_variable(2)] = 1 and [disease(2)] = 1 and [review_no_meta(1)] <> 1 and [animal(1)] <> 1 and [in_silico(1)] <> 1 and [cadavers(1)] <> 1	Select 'Maybe' in Covidence	descriptive
	30	knee_inj_iv_ov_maybe<br>Show the field ONLY if: [knee_injury(2)]= 1 and [age(1)] = 1 and [independent_variable(2)] = 1 and [disease(2)] = 1 and [review_no_meta(1)] <> 1 and [animal(1)] <> 1 and [in_silico(1)] <> 1 and [cadavers(1)] <> 1	Select 'Maybe' in Covidence	descriptive
	31	knee_inj_age_ov_maybe<br>Show the field ONLY if: [knee_injury(2)]= 1 and [age(2)] = 1 and [independent_variable(1)] = 1 and [disease(2)] = 1 and [review_no_meta(1)] <> 1 and [animal(1)] <> 1 and [in_silico(1)] <> 1 and [cadavers(1)] <> 1	Select 'Maybe' in Covidence	descriptive
	32	knee_inj_age_iv_maybe<br>Show the field ONLY if: [knee_injury(2)]= 1 and [age(2)] = 1 and [independent_variable(2)] = 1 and [review_no_meta(1)] <> 1 and [animal(1)] <> 1 and [in_silico(1)] <> 1 and [cadavers(1)] <> 1	Select 'Maybe' in Covidence	descriptive
	33	knee_inj_age_iv_maybe_2<br>Show the field ONLY if: [disease_diagnoses(2)] = 1 AND [medical_imaging_indicators(2)] = 1 AND [biomarkers(2)] = 1	Select 'Maybe' in Covidence	descriptive
	34	select_no<br>Show the field ONLY if: [review_no_meta(3)] = 1 or [animal(3)] = 1 or [in_silico(3)] = 1 or [in_vitro(3)] = 1 or [cadavers(3)] = 1 or [knee_oa(3)] =1 or [knee_injury(1)] = 1 or [age(1)] = 1 or [independent_variable(1)] = 1	Select 'No' in Covidence	descriptive

	35	select_no_2<br><br>Show the field ONLY if:<br>[disease_diagnoses(1)] = 1 AND [medical_imaging_indicators(1)] = 1 AND [biomarkers(1)] = 1	Select 'No' in Covidence	descriptive
	36	screening_tool_complete	Section Header: *Form Status*<br><br>Complete?	dropdown<br>0 Incomplete<br>1 Unverified<br>2 Complete

For peer review only

Screening Tool: Full Text

📖 Codebook

📖 Data Dictionary Codebook

2022-12-22 21:35:11

Collapse all instruments

	#	Variable / Field Name	Field Label<br>*Field Note*	Field Attributes (Field Type, Validation, Choices, Calculations, etc.)
Instrument: **Screening Tool** (screening_tool)				Collapse
	1	record_id	Record ID	text
	2	instructions	Yes - There is explicit evidence in the title or abstract of the research including this.	descriptive
	3	maybe	Maybe - It is unclear whether there is evidence of this in the title/abstract. It may or may not be relevant but I feel this needs further investigation.	descriptive
	4	no	No - There is explicit evidence in the title or abstract of the research not including this.	descriptive
	5	reviewer_screening	Section Header: *Reviewer Details*<br>Reviewer	radio, Required<br>1 Karl Morgan<br>2 James Cowburn<br>3 Mathew Farrow<br>4 Josh Carter
	6	reviewer_date_screening	Date<br>*Just press 'Today'*	text (date_dmy), Required
	7	record_number_screening	Record number	text (number), Required
	8	review_no_meta	Section Header: *1. Study Information*<br>Is this a review, meta-analysis, book, protocol or commentary?	checkbox<br>1 review_no_meta___1 No<br>2 review_no_meta___2 Maybe<br>3 review_no_meta___3 Yes
	9	animal<br>Show the field ONLY if:<br>[review_no_meta(3)] <> 1	Is the study an animal model?	checkbox<br>1 animal___1 No<br>2 animal___2 Maybe<br>3 animal___3 Yes
	10	in_silico<br>Show the field ONLY if:<br>[review_no_meta(3)] <> 1 and [animal(3)] <> 1	Is the study purely in silico?	checkbox<br>1 in_silico___1 No<br>2 in_silico___2 Maybe<br>3 in_silico___3 Yes
	11	in_vitro<br>Show the field ONLY if:<br>[review_no_meta(3)] <> 1 and [animal(3)] <> 1 and [in_silico (3)] <> 1	Is the study purely in vitro (i.e. no human participants)?	checkbox<br>1 in_vitro___1 No<br>2 in_vitro___2 Maybe<br>3 in_vitro___3 Yes
	12	cadavers<br>Show the field ONLY if:<br>[review_no_meta(3)] <> 1 and [animal(3)] <> 1 and [in_silico (3)] <> 1 and [in_vitro(3)] <> 1	Is the study only working with cadavers?	checkbox<br>1 cadavers___1 No<br>2 cadavers___2 Maybe<br>3 cadavers___3 Yes
	13	knee_oa<br>Show the field ONLY if:<br>[review_no_meta(3)] <> 1 and [animal(3)] <> 1 and [in_silico (3)] <> 1 and [in_vitro(3)] <> 1 and [cadavers(3)] <> 1	Do the participants already have knee osteoarthritis (e.g., a cross-sectional study looking at the effects of exercise in people who already have knee OA disease) or another type of arthritis/disease?	checkbox<br>1 knee_oa___1 No<br>2 knee_oa___2 Maybe<br>3 knee_oa___3 Yes

	14	knee_injury<br><br>Show the field ONLY if:<br>[review_no_meta(3)] <> 1 and [animal(3)] <> 1 and [in_silico(3)] <> 1 and [in_vitro(3)] <> 1 and [cadavers(3)] <> 1 and [knee_oa(3)] <> 1	Section Header: *2. Population*<br><br>Could this study involve those at risk of knee post-traumatic osteoarthritis (PTOA) such as after a knee injury? Cruciate ligament injuryCruciate ligament reconstructionMeniscus injuryMeniscectomy<br><br>This does NOT include:<br>Total knee replacementKnee arthroplastyosteotomy<br>*Please select one option*	checkbox, Required<br>1 | knee_injury___1 | No<br>2 | knee_injury___2 | Maybe<br>3 | knee_injury___3 | Yes
	15	age<br><br>Show the field ONLY if:<br>[review_no_meta(3)] <> 1 and [animal(3)] <> 1 and [in_silico(3)] <> 1 and [in_vitro(3)] <> 1 and [cadavers(3)] <> 1 and [knee_oa(3)] <> 1 and [knee_injury(1)] <> 1	Could the study participants be aged 18-40?<br>At least 50% of the population must be within 18-40 years old.<br><br>If the study is longitudinal, use the baseline age.<br>*Please select one option.*	checkbox, Required<br>1 | age___1 | No<br>2 | age___2 | Maybe<br>3 | age___3 | Yes
	16	independent_variable<br><br>Show the field ONLY if:<br>[review_no_meta(3)] <> 1 and [animal(3)] <> 1 and [in_silico(3)] <> 1 and [in_vitro(3)] <> 1 and [cadavers(3)] <> 1 and [knee_oa(3)] <> 1 and [knee_injury(1)] <> 1 and [age(1)] <> 1	Section Header: *3. Independent Variable*<br><br>Could the study have an independent variable which measures:<br>Physical activity (including Tegner)ExerciseSystemic inflammation (e.g., IL-6 concentrations)Knee joint load (moments or MSK modelling)Adipose tissueMuscle strengthMuscle sizeIntra-muscular adipose tissueBone mineral content or density<br>Or may the study have an intervention involving:<br><br>Physical activity or exerciseManipulation of joint loadWeight lossDisuse or bedrest<br>*Please select one option*	checkbox, Required<br>1 | independent_variable___1 | No<br>2 | independent_variable___2 | Maybe<br>3 | independent_variable___3 | Yes
	17	diagnosis_header<br><br>Show the field ONLY if:<br>[review_no_meta(3)] <> 1 and [animal(3)] <> 1 and [in_silico(3)] <> 1 and [in_vitro(3)] <> 1 and [cadavers(3)] <> 1 and [knee_oa(3)] <> 1 and [knee_injury(1)] <> 1 and [age(1)] <> 1 and [independent_variable(1)] <> 1	Section Header: *4. Outcome Variable*<br><br>Knee Osteoarthritis (OA) Diagnosis	descriptive
	18	disease_diagnoses<br><br>Show the field ONLY if:<br>[review_no_meta(3)] <> 1 and [animal(3)] <> 1 and [in_silico(3)] <> 1 and [in_vitro(3)] <> 1 and [cadavers(3)] <> 1 and [knee_oa(3)] <> 1 and [knee_injury(1)] <> 1 and [age(1)] <> 1 and [independent_variable(1)] <> 1	Could the study investigate knee OA diagnosis including the following scales:<br>Kellgren and Lawrence (K/L)Osteoarthritis Research Society International (OARSI) AtlasInternational Knee Documentation Committee (IKDC) SystemAhlbäck SystemFairbank ClassificationBrandt Grading ScaleJäger-Wirth ClassificationMerchant SystemMRI Osteoarthritis Knee Score (MOAKS)Whole Organ Magnetic Resonance Imaging Score (WORMS)Boston Leeds OA Knee Score (BLOKS)<br><br>P.s if there is another system mentioned that your are unsure of then please send me a message.<br>*Please select one option*	checkbox, Required<br>1 | disease_diagnoses___1 | No<br>2 | disease_diagnoses___2 | Maybe<br>3 | disease_diagnoses___3 | Yes
	19	imaging_indicator_header<br><br>Show the field ONLY if:<br>[review_no_meta(3)] <> 1 and [animal(3)] <> 1 and [in_silico(3)] <> 1 and [in_vitro(3)] <> 1 and [cadavers(3)] <> 1 and [knee_oa(3)] <> 1 and [knee_injury(1)] <> 1 and [age(1)] <> 1 and [independent_variable(1)] <> 1	Medical Imaging Indicator	descriptive
	20	medical_imaging_indicators<br><br>Show the field ONLY if:<br>[review_no_meta(3)] <> 1 and [animal(3)] <> 1 and [in_silico(3)] <> 1 and [in_vitro(3)] <> 1 and [cadavers(3)] <> 1 and [knee_oa(3)] <> 1 and [knee_injury(1)] <> 1 and [age(1)] <> 1 and [independent_variable(1)] <> 1	Could the study investigate medical imaging indicators of tissue health including the following techniques:<br>MRI quantified cartilage thickness and volumeT1rho mappingT2 mappingDelayed contrast-enhanced MRI of cartilage (dGEMRIC)Ultrasound quantified cartilage thickness<br>*Please select one option*	checkbox, Required<br>1 | medical_imaging_indicators___1 | No<br>2 | medical_imaging_indicators___2 | Maybe<br>3 | medical_imaging_indicators___3 | Yes

For peer review only - http://bmjopen.bmj.com/site/about/guidelines.xhtml

	21	biomarkers_header<br>Show the field ONLY if:<br>[review_no_meta(3)] <> 1 and [animal(3)] <> 1 and [in_silico(3)] <> 1 and [in_vitro(3)] <> 1 and [cadavers(3)] <> 1 and [knee_oa(3)] <> 1 and [knee_injury(1)] <> 1 and [age(1)] <> 1 and [independent_variable(1)] <> 1	Biomarkers	descriptive
	22	biomarkers<br>Show the field ONLY if:<br>[review_no_meta(3)] <> 1 and [animal(3)] <> 1 and [in_silico(3)] <> 1 and [in_vitro(3)] <> 1 and [cadavers(3)] <> 1 and [knee_oa(3)] <> 1 and [knee_injury(1)] <> 1 and [age(1)] <> 1 and [independent_variable(1)] <> 1	Could the study investigate biomarkers including the following:<br>Cartilage synthesisC-propeptide of type II collagen (CP-II)Chondroitin sulphate 846 epitope (CS846)N-Propeptide of Collagen IIA (PIIANP)N-Propeptide of Collagen IIB (PIIBNP)<br>Cartilage degradationMatrix metalloproteases (e.g., MMP-3, MMP-9, MMP-13)A disintegrin and metalloproteinase with thrombospondin motifs (e.g., ADAMTS-4, ADAMTS-5)Collagen type II cleavage product (C2C)Col2-3/4 C-terminal cleavage product of types I and II collagen (C1,2C)Neoepitope of collagen X (C10C or Col10Neo)Hyaluronic acid (HA) Aggrecan or aggrecan fragments (ARGS)Keratin sulphate (KS)Cartilage oligomeric matrix protein (COMP)<br>Bone formationOsteocalcin (OC)Bone alkaline phosphatase (BAP)Procollagen type I N-terminal propeptide (PINP)Procollagen type I C-terminal propeptide (PICP)<br>Bone resorptionPyridinoline (PYD)Deoxypyridinoline (DPD)Tartrate-resistant acid phosphatase 5b (TRAP-5)N-telopeptide if type I collagen (NTX-I)C-telopeptide of type I collagen (CTX-I)Insulin-like growth factor 1 (IGF-1)Transforming growth factor beta (TGFB)<br>*Please select one option*	checkbox, Required<br>1 | biomarkers___1 | No<br>2 | biomarkers___2 | Maybe<br>3 | biomarkers___3 | Yes
	23	all_yes_dec<br>Show the field ONLY if:<br>[knee_injury(3)]= 1 and [age(3)] = 1 and [independent_variable(3)] = 1 and [review_no_meta(3)] <> 1 and [animal(3)] <> 1 and [in_silico(3)] <> 1 and [cadavers(3)] <> 1 or [disease_diagnoses(3)] = 1 or [medical_imaging_indicators(3)] = 1 or [biomarkers(3)] = 1	Section Header: *Decision*<br>Select 'Yes' in Covidence	descriptive
	24	all_maybe_dec<br>Show the field ONLY if:<br>[knee_injury(2)]= 1 and [age(2)] = 1 and [independent_variable(2)] = 1 and [review_no_meta(1)] <> 1 and [animal(1)] <> 1 and [in_silico(1)] <> 1 and [cadavers(1)] <> 1	Select 'Maybe' in Covidence	descriptive
	25	knee_inj_maybe<br>Show the field ONLY if:<br>[knee_injury(2)]= 1 and [age(1)] = 1 and [independent_variable(1)] = 1 and [disease(1)] = 1	Select 'Maybe' in Covidence	descriptive
	26	age_maybe<br>Show the field ONLY if:<br>[knee_injury(1)]= 1 and [age(2)] = 1 and [independent_variable(1)] = 1 and [disease(1)] = 1 and [review_no_meta(1)] <> 1 and [animal(1)] <> 1 and [in_silico(1)] <> 1 and [cadavers(1)] <> 1	Select 'Maybe' in Covidence	descriptive

	27	iv_maybe<br>Show the field ONLY if: [knee_injury(1)]= 1 and [age(1)] = 1 and [independent_variable(2)] = 1 and [disease(1)] = 1 and [review_no_meta(1)] <> 1 and [animal(1)] <> 1 and [in_silico(1)] <> 1 and [cadavers(1)] <> 1	Select 'Maybe' in Covidence	descriptive
	28	ov_maybe<br>Show the field ONLY if: [knee_injury(1)]= 1 and [age(1)] = 1 and [independent_variable(1)] = 1 and [disease(2)] = 1 and [review_no_meta(1)] <> 1 and [animal(1)] <> 1 and [in_silico(1)] <> 1 and [cadavers(1)] <> 1	Select 'Maybe' in Covidence	descriptive
	29	age_iv_ov_maybe<br>Show the field ONLY if: [knee_injury(1)]= 1 and [age(2)] = 1 and [independent_variable(2)] = 1 and [disease(2)] = 1 and [review_no_meta(1)] <> 1 and [animal(1)] <> 1 and [in_silico(1)] <> 1 and [cadavers(1)] <> 1	Select 'Maybe' in Covidence	descriptive
	30	knee_inj_iv_ov_maybe<br>Show the field ONLY if: [knee_injury(2)]= 1 and [age(1)] = 1 and [independent_variable(2)] = 1 and [disease(2)] = 1 and [review_no_meta(1)] <> 1 and [animal(1)] <> 1 and [in_silico(1)] <> 1 and [cadavers(1)] <> 1	Select 'Maybe' in Covidence	descriptive
	31	knee_inj_age_ov_maybe<br>Show the field ONLY if: [knee_injury(2)]= 1 and [age(2)] = 1 and [independent_variable(1)] = 1 and [disease(2)] = 1 and [review_no_meta(1)] <> 1 and [animal(1)] <> 1 and [in_silico(1)] <> 1 and [cadavers(1)] <> 1	Select 'Maybe' in Covidence	descriptive
	32	knee_inj_age_iv_maybe<br>Show the field ONLY if: [knee_injury(2)]= 1 and [age(2)] = 1 and [independent_variable(2)] = 1 and [review_no_meta(1)] <> 1 and [animal(1)] <> 1 and [in_silico(1)] <> 1 and [cadavers(1)] <> 1	Select 'Maybe' in Covidence	descriptive
	33	knee_inj_age_iv_maybe_2<br>Show the field ONLY if: [disease_diagnoses(2)] = 1 AND [medical_imaging_indicators(2)] = 1 AND [biomarkers(2)] = 1	Select 'Maybe' in Covidence	descriptive
	34	select_no<br>Show the field ONLY if: [review_no_meta(3)] = 1 or [animal(3)] = 1 or [in_silico(3)] = 1 or [in_vitro(3)] = 1 or [cadavers(3)] = 1 or [knee_oa(3)] =1 or [knee_injury(1)] = 1 or [age(1)] = 1 or [independent_variable(1)] = 1	Select 'No' in Covidence	descriptive

	35	select_no_2<br><br>Show the field ONLY if:<br>[disease_diagnoses(1)] = 1 AND [medical_imaging_indicators(1)] = 1 AND [biomarkers(1)] = 1	Select 'No' in Covidence	descriptive
	36	screening_tool_complete	Section Header: *Form Status*<br><br>Complete?	dropdown<br>0 Incomplete<br>1 Unverified<br>2 Complete

For peer review only

Supplementary Material 5. Protocol for Contacting Authors

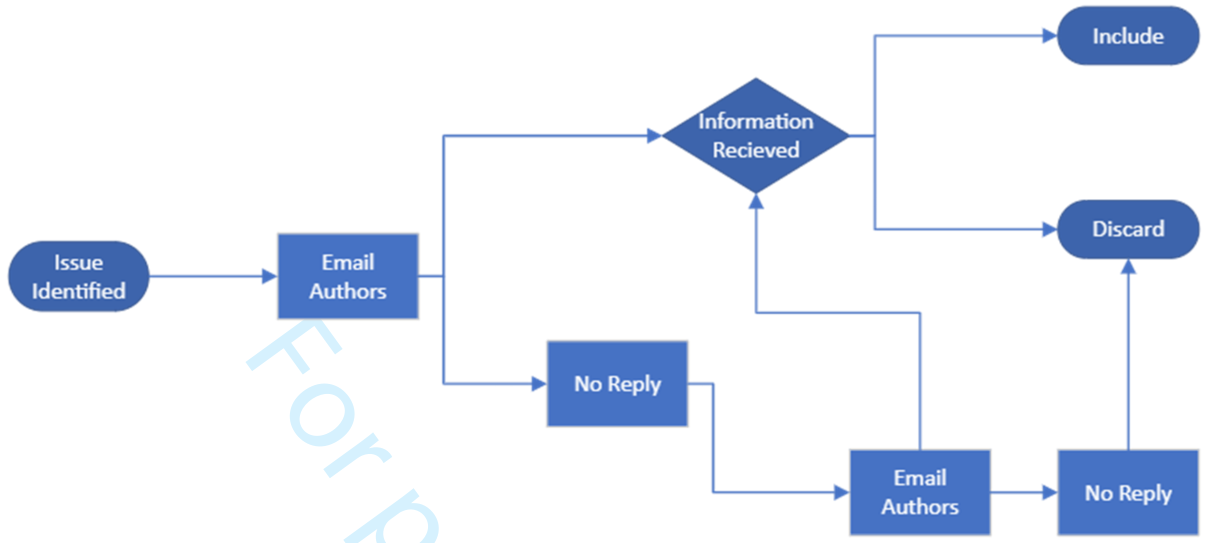

For peer review only

Supplementary Material 6. Johanna Brigg's Institute (JBI) Appraisal Criteria

REDCap Flow Diagram for Critical Appraisal

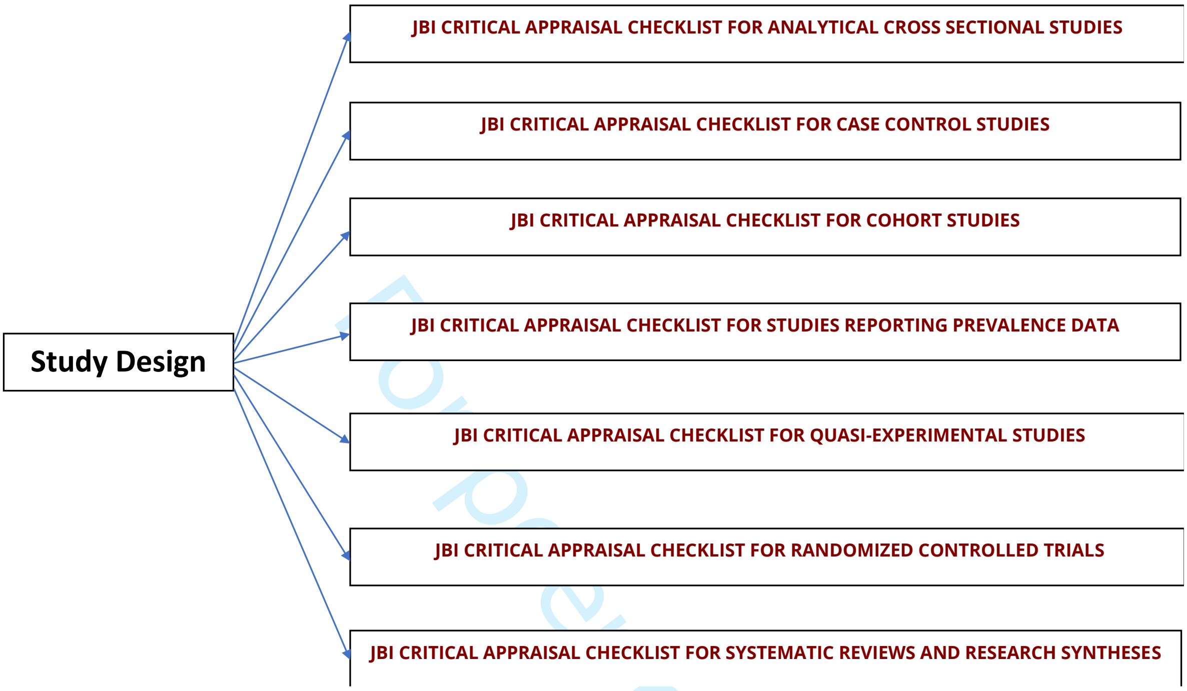

For peer review only

JBI Quality Appraisal

Codebook

Data Dictionary Codebook

2022-08-02 19:46:29

Collapse all instruments

	#	Variable / Field Name	Field Label<br>*Field Note*	Field Attributes (Field Type, Validation, Choices, Calculations, etc.)
Instrument: **Study Design** (study_design)				Collapse
	1	record_id	Record ID	text
	2	study_design	Please select the study design	radio<br>1 Analytical Cross-Sectional Study<br>2 Case Control Study<br>3 Cohort Study<br>4 Prevalence Study<br>5 Quasi-Experimental Study<br>6 Randomised Control Trial<br>7 Systematic Review and Research Synthesis<br>8 Unclear
	3	study_design_complete	Section Header: *Form Status*<br>Complete?	dropdown<br>0 Incomplete<br>1 Unverified<br>2 Complete
Instrument: **Study Design Unclear** (study_design_unclear)				Collapse
	4	reviewer_name_unclear	Section Header: *Reviewer Details*<br>Reviewer	dropdown<br>1 Karl Morgan<br>2 James Cowburn<br>3 Matthew Farrow
	5	reviewer_date_unclear	Date reviewed<br>*Just press 'Today'*	text (date_dmy)
	6	authors_unclear	Section Header: *Study Information*<br>Authors	text
	7	year_unclear	Publication year	text
	8	record_number_unclear	Record number	text
	9	study_design_unclear_complete	Section Header: *Form Status*<br>Complete?	dropdown<br>0 Incomplete<br>1 Unverified<br>2 Complete
Instrument: **JBI Critical Appraisal Checklist for Analytical Cross-Sectional Studies** (jbi_critical_appraisal_checklist_for_analytical_cr)				Collapse
	10	reviewer_name_cross	Section Header: *Reviewer Details*<br>Reviewer	dropdown<br>1 Karl Morgan<br>2 James Cowburn<br>3 Matthew Farrow
	11	reviewer_date_cross	Date reviewed<br>*Just press 'Today'*	text (date_dmy)
	12	authors_cross	Section Header: *Study Information*<br>Authors	text
	13	year_cross	Publication year	text
	14	record_number_cross	Record number	text

	15	cross_section_01	Section Header: *JBI Critical Appraisal Checklist for Analytical Cross-Sectional Studies*<br>1. Were the criteria for inclusion in the sample clearly defined?	radio (Matrix)<br>1 Yes<br>2 No<br>3 Unclear<br>4 Not Applicable
	16	cross_section_02	2. Were the study subjects and the setting described in detail?	radio (Matrix)<br>1 Yes<br>2 No<br>3 Unclear<br>4 Not Applicable
	17	cross_section_03	3. Was the exposure measured in a valid and reliable way?	radio (Matrix)<br>1 Yes<br>2 No<br>3 Unclear<br>4 Not Applicable
	18	cross_section_04	4. Were objective, standard criteria used for measurement of the condition?	radio (Matrix)<br>1 Yes<br>2 No<br>3 Unclear<br>4 Not Applicable
	19	cross_section_05	5. Were confounding factors identified?	radio (Matrix)<br>1 Yes<br>2 No<br>3 Unclear<br>4 Not Applicable
	20	cross_section_06	6. Were strategies to deal with confounding factors stated?	radio (Matrix)<br>1 Yes<br>2 No<br>3 Unclear<br>4 Not Applicable
	21	cross_section_07	7. Were the outcomes measured in a valid and reliable way?	radio (Matrix)<br>1 Yes<br>2 No<br>3 Unclear<br>4 Not Applicable
	22	cross_section_08	8. Was appropriate statistical analysis used?	radio (Matrix)<br>1 Yes<br>2 No<br>3 Unclear<br>4 Not Applicable
	23	jbi_critical_appraisal_checklist_for_analytical_cr_complete	Section Header: *Form Status*<br>Complete?	dropdown<br>0 Incomplete<br>1 Unverified<br>2 Complete
Instrument: **JBI Critical Appraisal Checklist for Case Control Studies** (jbi_critical_appraisal_checklist_for_case_control) Collapse				
	24	reviewer_name_case	Section Header: *Reviewer Details*<br>Reviewer	dropdown<br>1 Karl Morgan<br>2 James Cowburn<br>3 Matthew Farrow
	25	reviewer_date_case	Date reviewed<br>*Just press 'Today'*	text (date_dmy)
	26	authors_case	Section Header: *Study Information*<br>Authors	text

	27	year_case	Publication year	text
	28	record_number_case	Record number	text
	29	case_control_01	Section Header: *JBI Critical Appraisal Checklist for Case Control Studies*<br>1. Were the groups comparable other than the presence of disease in cases or the absence of disease in controls?	radio (Matrix)<br>1 Yes<br>2 No<br>3 Unclear<br>4 Not Applicable
	30	case_control_02	2. Were cases and controls matched appropriately?	radio (Matrix)<br>1 Yes<br>2 No<br>3 Unclear<br>4 Not Applicable
	31	case_control_03	3. Were the same criteria used for identification of cases and controls?	radio (Matrix)<br>1 Yes<br>2 No<br>3 Unclear<br>4 Not Applicable
	32	case_control_04	4. Was exposure measured in a standard, valid and reliable way?	radio (Matrix)<br>1 Yes<br>2 No<br>3 Unclear<br>4 Not Applicable
	33	case_control_05	5. Was exposure measured in the same way for cases and controls?	radio (Matrix)<br>1 Yes<br>2 No<br>3 Unclear<br>4 Not Applicable
	34	case_control_06	6. Were confounding factors identified?	radio (Matrix)<br>1 Yes<br>2 No<br>3 Unclear<br>4 Not Applicable
	35	case_control_07	7. Were strategies to deal with confounding factors stated?	radio (Matrix)<br>1 Yes<br>2 No<br>3 Unclear<br>4 Not Applicable
	36	case_control_08	8. Were outcomes assessed in a standard, valid and reliable way for cases and controls?	radio (Matrix)<br>1 Yes<br>2 No<br>3 Unclear<br>4 Not Applicable
	37	case_control_09	9. Was the exposure period of interest long enough to be meaningful?	radio (Matrix)<br>1 Yes<br>2 No<br>3 Unclear<br>4 Not Applicable
	38	case_control_10	10. Was appropriate statistical analysis used?	radio (Matrix)<br>1 Yes<br>2 No<br>3 Unclear<br>4 Not Applicable

	39	jbi_critical_appraisal_checklist_for_case_control_complete	Section Header: *Form Status*<br>Complete?	dropdown<br>0 Incomplete<br>1 Unverified<br>2 Complete
Instrument: **JBI Critical Appraisal Checklist for Cohort Studies** (jbi_critical_appraisal_checklist_for_cohort_studie)				Collapse
	40	reviewer_name_cohort	Section Header: *Reviewer Details*<br>Reviewer	dropdown<br>1 Karl Morgan<br>2 James Cowburn<br>3 Matthew Farrow
	41	reviewer_date_cohort	Date reviewed<br>*Just press 'Today'*	text (date_dmy)
	42	authors_cohort	Section Header: *Study Information*<br>Authors<br>*Surname*	text
	43	year_cohort	Publication year<br>*e.g., 2010*	text
	44	record_number_cohort	Record number	text
	45	cohort_01	Section Header: *JBI Critical Appraisal Checklist for Cohort Studies*<br>1. Were the two groups similar and recruited from the same population?	radio (Matrix)<br>1 Yes<br>2 No<br>3 Unclear<br>4 Not Applicable
	46	cohort_02	2. Were the exposures measured similarly to assign people to both exposed and unexposed groups?	radio (Matrix)<br>1 Yes<br>2 No<br>3 Unclear<br>4 Not Applicable
	47	cohort_03	3. Was the exposure measured in a valid and reliable way?	radio (Matrix)<br>1 Yes<br>2 No<br>3 Unclear<br>4 Not Applicable
	48	cohort_04	4. Were confounding factors identified?	radio (Matrix)<br>1 Yes<br>2 No<br>3 Unclear<br>4 Not Applicable
	49	cohort_05	5. Were strategies to deal with confounding factors stated?	radio (Matrix)<br>1 Yes<br>2 No<br>3 Unclear<br>4 Not Applicable
	50	cohort_06	6. Were the groups/participants free of the outcome at the start of the study (or at the moment of exposure)?	radio (Matrix)<br>1 Yes<br>2 No<br>3 Unclear<br>4 Not Applicable
	51	cohort_07	7. Were the outcomes measured in a valid and reliable way?	radio (Matrix)<br>1 Yes<br>2 No<br>3 Unclear<br>4 Not Applicable

	52	cohort_08	8. Was the follow up time reported and sufficient to be long enough for outcomes to occur?	radio (Matrix)<br>1 Yes<br>2 No<br>3 Unclear<br>4 Not Applicable
	53	cohort_09	9. Was follow up complete, and if not, were the reasons to loss to follow up described and explored?	radio (Matrix)<br>1 Yes<br>2 No<br>3 Unclear<br>4 Not Applicable
	54	cohort_10	10. Were strategies to address incomplete follow up utilized?	radio (Matrix)<br>1 Yes<br>2 No<br>3 Unclear<br>4 Not Applicable
	55	cohort_11	11. Was appropriate statistical analysis used?	radio (Matrix)<br>1 Yes<br>2 No<br>3 Unclear<br>4 Not Applicable
	56	jbi_critical_appraisal_checklist_for_cohort_studie_complete	Section Header: *Form Status*<br>Complete?	dropdown<br>0 Incomplete<br>1 Unverified<br>2 Complete
Instrument: **JBI Critical Appraisal Checklist for Studies Reporting Prevalence Data** (jbi_critical_appraisal_checklist_for_studies_repor) Collapse				
	57	reviewer_name_prevalence	Section Header: *Reviewer Details*<br>Reviewer	dropdown<br>1 Karl Morgan<br>2 James Cowburn<br>3 Matthew Farrow
	58	reviewer_date_prevalence	Date reviewed<br>*Just press 'Today'*	text (date_dmy)
	59	authors_prevalence	Section Header: *Study Information*<br>Authors	text
	60	year_prevalence	Publication year	text
	61	record_number_prevalence	Record number	text
	62	prevalence_01	Section Header: *JBI Critical Appraisal Checklist for Studies Reporting Prevalence Data*<br>1. Was the sample frame appropriate to address the target population?	radio (Matrix)<br>1 Yes<br>2 No<br>3 Unclear<br>4 Not Applicable
	63	prevalence_02	2. Were study participants sampled in an appropriate way?	radio (Matrix)<br>1 Yes<br>2 No<br>3 Unclear<br>4 Not Applicable
	64	prevalence_03	3. Was the sample size adequate?	radio (Matrix)<br>1 Yes<br>2 No<br>3 Unclear<br>4 Not Applicable

	65	prevalence_04	4. Were the study subjects and the setting described in detail?	radio (Matrix)<br>1 Yes<br>2 No<br>3 Unclear<br>4 Not Applicable
	66	prevalence_05	5. Was the data analysis conducted with sufficient coverage of the identified sample?	radio (Matrix)<br>1 Yes<br>2 No<br>3 Unclear<br>4 Not Applicable
	67	prevalence_06	6. Were valid methods used for the identification of the condition?	radio (Matrix)<br>1 Yes<br>2 No<br>3 Unclear<br>4 Not Applicable
	68	prevalence_07	7. Was the condition measured in a standard, reliable way for all participants?	radio (Matrix)<br>1 Yes<br>2 No<br>3 Unclear<br>4 Not Applicable
	69	prevalence_08	8. Was there appropriate statistical analysis?	radio (Matrix)<br>1 Yes<br>2 No<br>3 Unclear<br>4 Not Applicable
	70	prevalence_09	9. Was the response rate adequate, and if not, was the low response rate managed appropriately?	radio (Matrix)<br>1 Yes<br>2 No<br>3 Unclear<br>4 Not Applicable
	71	jbi_critical_appraisal_checklist_for_studies_repor_complete	Section Header: *Form Status*<br>Complete?	dropdown<br>0 Incomplete<br>1 Unverified<br>2 Complete
Instrument: **JBI Critical Appraisal Checklist for Quasi-Experimental Studies** (jbi_critical_appraisal_checklist_for_quasiexperime) Collapse				
	72	reviewer_name_quasi	Section Header: *Reviewer Details*<br>Reviewer	dropdown<br>1 Karl Morgan<br>2 James Cowburn<br>3 Matthew Farrow
	73	reviewer_date_quasi	Date reviewed<br>*Just press 'Today'*	text (date_dmy)
	74	author_quasi	Section Header: *Study Information*<br>Authors	text
	75	year_quasi	Publication year	text
	76	record_number_quasi	Record number	text
	77	quasi_experimental_01	Section Header: *JBI Critical Appraisal Checklist for Quasi-Experimental Studies*<br>1. Is it clear in the study what is the 'cause' and what is the 'effect' (i.e. there is no confusion about which variable comes first)?	radio (Matrix)<br>1 Yes<br>2 No<br>3 Unclear<br>4 Not Applicable

	78	quasi_experimental_02	2. Were the participants included in any comparisons similar?	radio (Matrix)<br>1 Yes<br>2 No<br>3 Unclear<br>4 Not Applicable
	79	quasi_experimental_03	3. Were the participants included in any comparisons receiving similar treatment/care, other than the exposure or intervention of interest?	radio (Matrix)<br>1 Yes<br>2 No<br>3 Unclear<br>4 Not Applicable
	80	quasi_experimental_04	4. Was there a control group?	radio (Matrix)<br>1 Yes<br>2 No<br>3 Unclear<br>4 Not Applicable
	81	quasi_experimental_05	5. Were there multiple measurements of the outcome both pre and post the intervention/exposure?	radio (Matrix)<br>1 Yes<br>2 No<br>3 Unclear<br>4 Not Applicable
	82	quasi_experimental_06	6. Was follow up complete and if not, were differences between groups in terms of their follow up adequately described and analyzed?	radio (Matrix)<br>1 Yes<br>2 No<br>3 Unclear<br>4 Not Applicable
	83	quasi_experimental_07	7. Were the outcomes of participants included in any comparisons measured in the same way?	radio (Matrix)<br>1 Yes<br>2 No<br>3 Unclear<br>4 Not Applicable
	84	quasi_experimental_08	8. Were outcomes measured in a reliable way?	radio (Matrix)<br>1 Yes<br>2 No<br>3 Unclear<br>4 Not Applicable
	85	quasi_experimental_09	9. Was appropriate statistical analysis used?	radio (Matrix)<br>1 Yes<br>2 No<br>3 Unclear<br>4 Not Applicable
	86	jbi_critical_appraisal_checklist_for_quasiexperime_complete	Section Header: *Form Status*<br>Complete?	dropdown<br>0 Incomplete<br>1 Unverified<br>2 Complete
Instrument: **JBI Critical Appraisal Checklist for Randomised Controlled Trials** (jbi_critical_appraisal_checklist_for_randomised_co) Collapse				
	87	reviewer_name_rct	Section Header: *Reviewer Details*<br>Reviewer	dropdown<br>1 Karl Morgan<br>2 James Cowburn<br>3 Matthew Farrow
	88	reviewer_date_rct	Date reviewed<br>*Just press 'Today'*	text (date_dmy)

	89	authors_rct	Section Header: *Study Information*<br>Authors	text
	90	year_rct	Publication year	text
	91	record_number_rct	Record number	text
	92	rct_01	Section Header: *JBI Critical Appraisal Checklist for Randomised Controlled Trials*<br>1. Was true randomization used for assignment of participants to treatment groups?	radio (Matrix)<br>1 Yes<br>2 No<br>3 Unclear<br>4 Not Applicable
	93	rct_02	2. Was allocation to treatment groups concealed?	radio (Matrix)<br>1 Yes<br>2 No<br>3 Unclear<br>4 Not Applicable
	94	rct_03	3. Were treatment groups similar at the baseline?	radio (Matrix)<br>1 Yes<br>2 No<br>3 Unclear<br>4 Not Applicable
	95	rct_04	4. Were participants blind to treatment assignment?	radio (Matrix)<br>1 Yes<br>2 No<br>3 Unclear<br>4 Not Applicable
	96	rct_05	5. Were those delivering treatment blind to treatment assignment?	radio (Matrix)<br>1 Yes<br>2 No<br>3 Unclear<br>4 Not Applicable
	97	rct_06	6. Were outcomes assessors blind to treatment assignment?	radio (Matrix)<br>1 Yes<br>2 No<br>3 Unclear<br>4 Not Applicable
	98	rct_07	7. Were treatment groups treated identically other than the intervention of interest?	radio (Matrix)<br>1 Yes<br>2 No<br>3 Unclear<br>4 Not Applicable
	99	rct_08	8. Was follow up complete and if not, were differences between groups in terms of their follow up adequately described and analyzed?	radio (Matrix)<br>1 Yes<br>2 No<br>3 Unclear<br>4 Not Applicable
	100	rct_09	9. Were participants analyzed in the groups to which they were randomized?	radio (Matrix)<br>1 Yes<br>2 No<br>3 Unclear<br>4 Not Applicable

For peer review only - http://bmjopen.bmj.com/site/about/guidelines.xhtml

101	rct_10	10. Were outcomes measured in the same way for treatment groups?	radio (Matrix)<br>1 Yes<br>2 No<br>3 Unclear<br>4 Not Applicable
102	rct_11	11. Were outcomes measured in a reliable way?	radio (Matrix)<br>1 Yes<br>2 No<br>3 Unclear<br>4 Not Applicable
103	rct_12	12. Was appropriate statistical analysis used?	radio (Matrix)<br>1 Yes<br>2 No<br>3 Unclear<br>4 Not Applicable
104	rct_13	13. Was the trial design appropriate, and any deviations from the standard RCT design (individual randomization, parallel groups) accounted for in the conduct and analysis of the trial?	radio (Matrix)<br>1 Yes<br>2 No<br>3 Unclear<br>4 Not Applicable
105	jbi_critical_appraisal_checklist_for_randomised_co_complete	Section Header: *Form Status*<br>Complete?	dropdown<br>0 Incomplete<br>1 Unverified<br>2 Complete
Instrument: **JBI Critical Appraisal Checklist for Systematic Reviews and Research Syntheses** (jbi_critical_appraisal_checklist_for_systematic_re)			Collapse
106	reviewer_name_review	Section Header: *Study Information*<br>Reviewer	dropdown<br>1 Karl Morgan<br>2 James Cowburn<br>3 Matthew Farrow
107	reviewer_date_review	Date reviewed<br>*Just press 'Today'*	text (date_dmy)
108	authors_review	Section Header: *Study Information*<br>Authors	text
109	year_review	Publication year	text
110	record_number_review	Record number	text
111	reviews_01	Section Header: *JBI Critical Appraisal Checklist for Systematic Reviews and Research Syntheses*<br>1. Is the review question clearly and explicitly stated?	radio (Matrix)<br>1 Yes<br>2 No<br>3 Unclear<br>4 Not Applicable
112	reviews_02	2. Were the inclusion criteria appropriate for the review question?	radio (Matrix)<br>1 Yes<br>2 No<br>3 Unclear<br>4 Not Applicable
113	reviews_03	3. Was the search strategy appropriate?	radio (Matrix)<br>1 Yes<br>2 No<br>3 Unclear<br>4 Not Applicable

	114	reviews_04	4. Were the sources and resources used to search for studies adequate?	radio (Matrix)<br>1 Yes<br>2 No<br>3 Unclear<br>4 Not Applicable
	115	reviews_05	5. Were the criteria for appraising studies appropriate?	radio (Matrix)<br>1 Yes<br>2 No<br>3 Unclear<br>4 Not Applicable
	116	reviews_06	6. Was critical appraisal conducted by two or more reviewers independently?	radio (Matrix)<br>1 Yes<br>2 No<br>3 Unclear<br>4 Not Applicable
	117	reviews_07	7. Were there methods to minimize errors in data extraction?	radio (Matrix)<br>1 Yes<br>2 No<br>3 Unclear<br>4 Not Applicable
	118	reviews_08	8. Were the methods used to combine studies appropriate?	radio (Matrix)<br>1 Yes<br>2 No<br>3 Unclear<br>4 Not Applicable
	119	reviews_09	9. Was the likelihood of publication bias assessed?	radio (Matrix)<br>1 Yes<br>2 No<br>3 Unclear<br>4 Not Applicable
	120	reviews_10	10. Were recommendations for policy and/or practice supported by the reported data?	radio (Matrix)<br>1 Yes<br>2 No<br>3 Unclear<br>4 Not Applicable
	121	reviews_11	11. Were the specific directives for new research appropriate?	radio (Matrix)<br>1 Yes<br>2 No<br>3 Unclear<br>4 Not Applicable
	122	jbi_critical_appraisal_checklist_for_systematic_re_complete	Section Header: *Form Status*<br>Complete?	dropdown<br>0 Incomplete<br>1 Unverified<br>2 Complete

Data Charting Draft

Codebook

Data Dictionary Codebook

2022-12-22 21:44:12

Collapse all instruments

	#	Variable / Field Name	Field Label<br>*Field Note*	Field Attributes (Field Type, Validation, Choices, Calculations, etc.)
Instrument: **Data Extraction 2** (data_extraction_2)				Collapse
	1	record_id	Record ID	text
	2	reviewer_name_extraction_v2	Reviewer	dropdown, Required<br>1 Karl Morgan<br>2 James Cowburn<br>3 Mathew Farrow<br>4 Josh Carter
	3	reviewer_date_extraction_v2	Date of extraction<br>*Just press 'Today'*	text (date_dmy), Required
	4	record_number_extraction_v2	Section Header: *Study Information*<br>Record number	text, Required
	5	first_author_v2	First author<br>*Format as: second name, first name initial (e.g., Morgan, K.).*	text, Required
	6	co_authors_v2	Co-authors<br>*Format as: Initial followed by surname (e.g., Morgan, K.). Write n/a if none.*	notes, Required
	7	publication_year_v2	Publication year<br>*Please insert as number (e.g., 2006).*	text (number), Required
	8	country_research_origin_v2	Country of research origin<br><br>ISO Alpha 3 codes<br>*Please use ISO Alpha 3 codes (e.g., USA, GBR, FRA, DNK, CAN). Write unclear if unknown.*	text, Required
	9	study_aim_1_v2	Section Header: *Study aims*<br>Study aim 1<br>*Write unclear if unknown.*	notes, Required
	10	study_aim_2_v2	Study aim 2<br>*Write unclear if unknown. If no secondary aim write n/a.*	notes, Required
	11	study_aim_3_v2	Study aim 3<br>*Write unclear if unknown. If no tertiary aim write n/a.*	notes, Required
	12	study_design_v2	Section Header: *Methodology - Study Design*<br>Study design	radio, Required<br>1 Analytical cross-sectional<br>2 Case Control<br>3 Cohort<br>4 Prevalence data<br>5 Quasi-experimental<br>6 Randomised control trial<br>7 Unclear
	13	number_of_participants_v2	Section Header: *Methodology - Experimental Population Size*<br>Total number of participants<br>*Report as: number*	text (number), Required
	14	number_of_males_v2	Number of males<br>*Report as: number*	text, Required
	15	number_of_females_v2	Number of females<br>*Report as: number*	text, Required
	16	age_heading_v2	Section Header: *Methodology - Experimental Group Demographic Information*<br>Experimental Group Age	descriptive
	17	total_population_age_v2	Total Population	descriptive

	18	participant_age_total_v2	How was the age of the total study population reported?<br>*Select all relevant answers*	checkbox, Required<br>1 participant_age_total_v2___1 Mean<br>2 participant_age_total_v2___2 Standard deviation<br>3 participant_age_total_v2___3 Median<br>4 participant_age_total_v2___4 Range
	19	age_mean_v2<br>Show the field ONLY if: [participant_age_total_v2(1)] = '1'	Mean<br>*Years*	text, Required
	20	age_stdev_v2<br>Show the field ONLY if: [participant_age_total_v2(2)] = '1'	Standard deviation<br>*Years*	text, Required
	21	age_median_v2<br>Show the field ONLY if: [participant_age_total_v2(3)] = '1'	Median<br>*Years*	text, Required
	22	age_range_v2<br>Show the field ONLY if: [participant_age_total_v2(4)] = '1'	Range<br>*Years*	text, Required
	23	male_population_age_v2	Male Population	descriptive
	24	participant_age_male_v2	How was the age of the male study population reported?<br>*Select all relevant answers*	checkbox, Required<br>1 participant_age_male_v2___1 Mean<br>2 participant_age_male_v2___2 Standard deviation<br>3 participant_age_male_v2___3 Median<br>4 participant_age_male_v2___4 Range
	25	age_mean_male_v2<br>Show the field ONLY if: [participant_age_male_v2(1)] = '1'	Mean<br>*Years*	text, Required
	26	age_stdev_male_v2<br>Show the field ONLY if: [participant_age_male_v2(2)] = '1'	Standard deviation<br>*Years*	text, Required
	27	age_median_male_v2<br>Show the field ONLY if: [participant_age_male_v2(3)] = '1'	Median<br>*Years*	text, Required
	28	age_range_male_v2<br>Show the field ONLY if: [participant_age_male_v2(4)] = '1'	Range<br>*Years*	text, Required
	29	population_age_female_v2	Female Population	descriptive
	30	participant_age_female_v2	How was the age of the female study population reported?<br>*Select all relevant answers*	checkbox, Required<br>1 participant_age_female_v2___1 Mean<br>2 participant_age_female_v2___2 Standard deviation<br>3 participant_age_female_v2___3 Median<br>4 participant_age_female_v2___4 Range
	31	age_mean_female_v2<br>Show the field ONLY if: [participant_age_female_v2(1)] = '1'	Mean<br>*Years*	text, Required
	32	age_stdev_female_v2<br>Show the field ONLY if: [participant_age_female_v2(2)] = '1'	Standard deviation<br>*Years*	text, Required

	33	age_median_female_v2<br><br>Show the field ONLY if:<br>[participant_age_female_v2(3)] = '1'	Median<br>*Years*	text, Required
	34	age_range_female_v2<br><br>Show the field ONLY if:<br>[participant_age_female_v2(4)] = '1'	Range<br>*Years*	text, Required
	35	participant_anth_info_v2	Participant Anthropometric Information	descriptive
	36	total_population_anthro_v2	Total Population	descriptive
	37	participant_height_v2	How was participant height reported?<br>*Meters*	checkbox, Required<br>1 participant_height_v2___1 Mean<br>2 participant_height_v2___2 Standard deviation<br>3 participant_height_v2___3 Median<br>4 participant_height_v2___4 Range
	38	participant_height_mean_v2<br><br>Show the field ONLY if:<br>[participant_height_v2(1)] = '1'	Mean	text, Required
	39	participant_height_stdev_v2<br><br>Show the field ONLY if:<br>[participant_height_v2(2)] = '1'	Standard deviation	text, Required
	40	participant_height_median_v2<br><br>Show the field ONLY if:<br>[participant_height_v2(3)] = '1'	Median	text, Required
	41	participant_height_range_v2<br><br>Show the field ONLY if:<br>[participant_height_v2(4)] = '1'	Range	text, Required
	42	participant_mass_v2	Participant mass<br>*Kilograms*	checkbox, Required<br>1 participant_mass_v2___1 Mean<br>2 participant_mass_v2___2 Standard deviation<br>3 participant_mass_v2___3 Median<br>4 participant_mass_v2___4 Range
	43	participant_mass_mean_v2<br><br>Show the field ONLY if:<br>[participant_mass_v2(1)] = '1'	Mean	text, Required
	44	participant_mass_stdev_v2<br><br>Show the field ONLY if:<br>[participant_mass_v2(2)] = '1'	Standard deviation	text, Required
	45	participant_mass_median_v2<br><br>Show the field ONLY if:<br>[participant_mass_v2(3)] = '1'	Median	text, Required
	46	participant_mass_range_v2<br><br>Show the field ONLY if:<br>[participant_mass_v2(4)] = '1'	Range	text, Required
	47	participant_bmi_v2	Participant BMI<br>*Kilograms/meters^2*	checkbox, Required<br>1 participant_bmi_v2___1 Mean<br>2 participant_bmi_v2___2 Standard deviation<br>3 participant_bmi_v2___3 Median<br>4 participant_bmi_v2___4 Range
	48	participant_bmi_mean_v2<br><br>Show the field ONLY if:<br>[participant_bmi_v2(1)] = '1'	Mean	text, Required
	49	participant_bmi_stdev_v2<br><br>Show the field ONLY if:<br>[participant_bmi_v2(2)] = '1'	Standard deviation	text, Required
	50	participant_bmi_median_v2<br><br>Show the field ONLY if:<br>[participant_bmi_v2(3)] = '1'	Median	text, Required

For peer review only - http://bmjopen.bmj.com/site/about/guidelines.xhtml

	51	participant_bmi_range_v2<br>Show the field ONLY if:<br>[participant_bmi_v2(4)] = '1'	Range	text, Required
	52	male_population_anthro_v2	Male Population	descriptive
	53	male_exp_pp_height_v2	How was participant height reported?<br>*Meters*	checkbox, Required<br>1 | male_exp_pp_height_v2___1 | Mean<br>2 | male_exp_pp_height_v2___2 | Standard deviation<br>3 | male_exp_pp_height_v2___3 | Median<br>4 | male_exp_pp_height_v2___4 | Range
	54	male_exp_mean_height_v2<br>Show the field ONLY if:<br>[male_exp_pp_height_v2(1)] = '1'	Mean	text, Required
	55	male_exp_stdev_height_v2<br>Show the field ONLY if:<br>[male_exp_pp_height_v2(2)] = '1'	Standard deviation	text, Required
	56	male_exp_median_height_v2<br>Show the field ONLY if:<br>[male_exp_pp_height_v2(3)] = '1'	Median	text, Required
	57	male_exp_range_height_v2<br>Show the field ONLY if:<br>[male_exp_pp_height_v2(4)] = '1'	Range	text, Required
	58	male_exp_pp_mass_v2	How was participant mass reported?<br>*Meters*	checkbox, Required<br>1 | male_exp_pp_mass_v2___1 | Mean<br>2 | male_exp_pp_mass_v2___2 | Standard deviation<br>3 | male_exp_pp_mass_v2___3 | Median<br>4 | male_exp_pp_mass_v2___4 | Range
	59	male_exp_mean_mass_v2<br>Show the field ONLY if:<br>[male_exp_pp_mass_v2(1)] = '1'	Mean	text, Required
	60	male_exp_stdev_mass_v2<br>Show the field ONLY if:<br>[male_exp_pp_mass_v2(2)] = '1'	Standard deviation	text, Required
	61	male_exp_median_mass_v2<br>Show the field ONLY if:<br>[male_exp_pp_mass_v2(3)] = '1'	Median	text, Required
	62	male_exp_range_mass_v2<br>Show the field ONLY if:<br>[male_exp_pp_mass_v2(4)] = '1'	Range	text, Required
	63	male_exp_pp_bmi_v2	How was participant body mass index reported?<br>*Meters*	checkbox, Required<br>1 | male_exp_pp_bmi_v2___1 | Mean<br>2 | male_exp_pp_bmi_v2___2 | Standard deviation<br>3 | male_exp_pp_bmi_v2___3 | Median<br>4 | male_exp_pp_bmi_v2___4 | Range
	64	male_exp_mean_bmi_v2<br>Show the field ONLY if:<br>[male_exp_pp_bmi_v2(1)] = '1'	Mean	text, Required
	65	male_exp_stdev_bmi_v2<br>Show the field ONLY if:<br>[male_exp_pp_bmi_v2(2)] = '1'	Standard deviation	text, Required
	66	male_exp_median_bmi_v2<br>Show the field ONLY if:<br>[male_exp_pp_bmi_v2(3)] = '1'	Median	text, Required

For peer review only - http://bmjopen.bmj.com/site/about/guidelines.xhtml

	67	male_exp_range_bmi_v2<br><br>Show the field ONLY if:<br>[male_exp_pp_bmi_v2(4)] = '1'	Range	text, Required
	68	female_population_anthro_v2	Female Population	descriptive
	69	female_exp_pp_height_v2	How was participant height reported?<br>*Meters*	checkbox, Required<br>1 | female_exp_pp_height_v2___1 | Mean<br>2 | female_exp_pp_height_v2___2 | Standard deviation<br>3 | female_exp_pp_height_v2___3 | Median<br>4 | female_exp_pp_height_v2___4 | Range
	70	female_exp_mean_height_v2<br><br>Show the field ONLY if:<br>[female_exp_pp_height_v2(1)] = '1'	Mean	text, Required
	71	female_exp_sdtev_height_v2<br><br>Show the field ONLY if:<br>[female_exp_pp_height_v2(2)] = '1'	Standard deviation	text, Required
	72	female_exp_median_height_v2<br><br>Show the field ONLY if:<br>[female_exp_pp_height_v2(3)] = '1'	Median	text, Required
	73	female_exp_range_height_v2<br><br>Show the field ONLY if:<br>[female_exp_pp_height_v2(4)] = '1'	Range	text, Required
	74	female_exp_pp_mass_v2	How was participant mass reported?<br>*Meters*	checkbox, Required<br>1 | female_exp_pp_mass_v2___1 | Mean<br>2 | female_exp_pp_mass_v2___2 | Standard deviation<br>3 | female_exp_pp_mass_v2___3 | Median<br>4 | female_exp_pp_mass_v2___4 | Range
	75	female_exp_mean_mass_v2<br><br>Show the field ONLY if:<br>[female_exp_pp_mass_v2(1)] = '1'	Mean	text, Required
	76	female_exp_stdev_mass_v2<br><br>Show the field ONLY if:<br>[female_exp_pp_mass_v2(2)] = '1'	Standard deviation	text, Required
	77	female_exp_median_mass_v2<br><br>Show the field ONLY if:<br>[female_exp_pp_mass_v2(3)] = '1'	Median	text, Required
	78	female_exp_range_mass_v2<br><br>Show the field ONLY if:<br>[female_exp_pp_mass_v2(4)] = '1'	Range	text, Required
	79	female_exp_pp_bmi_v2	How was participant body mass index reported?<br>*Meters*	checkbox, Required<br>1 | female_exp_pp_bmi_v2___1 | Mean<br>2 | female_exp_pp_bmi_v2___2 | Standard deviation<br>3 | female_exp_pp_bmi_v2___3 | Median<br>4 | female_exp_pp_bmi_v2___4 | Range
	80	female_exp_mean_bmi_v2<br><br>Show the field ONLY if:<br>[female_exp_pp_bmi_v2(1)] = '1'	Mean	text, Required

	81	female_exp_stdev_bmi_v2<br><br>Show the field ONLY if:<br>[female_exp_pp_bmi_v2(2)] = '1'	Standard deviation	text, Required
	82	female_exp_median_bmi_v2<br><br>Show the field ONLY if:<br>[female_exp_pp_bmi_v2(3)] = '1'	Median	text, Required
	83	female_exp_range_bmi_v2<br><br>Show the field ONLY if:<br>[female_exp_pp_bmi_v2(4)] = '1'	Range	text, Required
	84	number_of_controls_v2<br><br>Show the field ONLY if:<br>[cntrl_participants_included] = 1	Section Header: *Methodology - Control Population Size*<br><br>Number of controls<br>*Report as: number*	text (number), Required
	85	number_of_males_control_v2<br><br>Show the field ONLY if:<br>[cntrl_participants_included] = 1	Number of control males<br>*Report as: number*	text, Required
	86	number_of_females_control_v2<br><br>Show the field ONLY if:<br>[number_of_controls_v2] = 1	Number of control females<br>*Report as: number*	text, Required
	87	cntrl_group_age_v2	Section Header: *Methodology - Control Demographic Information*<br><br>Control Group Age	descriptive
	88	total_cntrl_age_v2	Total Population	descriptive
	89	participant_cntrl_age_type_v2<br><br>Show the field ONLY if:<br>[number_of_controls_v2] = 1	How was the age of control participants reported?<br>*Select all relevant answers*	checkbox, Required<br>1 participant_cntrl_age_type_v2___1 Mean<br>2 participant_cntrl_age_type_v2___2 Standard deviation<br>3 participant_cntrl_age_type_v2___3 Median<br>4 participant_cntrl_age_type_v2___4 Range
	90	age_cntrl_mean_v2<br><br>Show the field ONLY if:<br>[participant_cntrl_age_type_v2(1)] = '1'	Mean	text, Required
	91	age_cntrl_stdev_v2<br><br>Show the field ONLY if:<br>[participant_cntrl_age_type_v2(2)] = '1'	Standard deviation	text, Required
	92	age_cntrl_median_v2<br><br>Show the field ONLY if:<br>[participant_cntrl_age_type_v2(3)] = '1'	Median	text, Required
	93	age_cntrl_range_v2<br><br>Show the field ONLY if:<br>[participant_cntrl_age_type_v2(4)] = '1'	Range	text, Required
	94	male_cntrl_age_v2	Male Population	descriptive
	95	male_pp_cntrl_age_type_v2	How was the age of control participants reported?<br>*Select all relevant answers*	checkbox, Required<br>1 male_pp_cntrl_age_type_v2___1 Mean<br>2 male_pp_cntrl_age_type_v2___2 Standard deviation<br>3 male_pp_cntrl_age_type_v2___3 Median<br>4 male_pp_cntrl_age_type_v2___4 Range
	96	age_cntrl_male_mean_v2<br><br>Show the field ONLY if:<br>[male_pp_cntrl_age_type_v2(1)] = '1'	Mean	text

	97	age_cntrl_male_stdev_v2<br><br>Show the field ONLY if:<br>[male_pp_cntrl_age_type_v2(2)] = '1'	Standard deviation	text
	98	age_cntrl_male_median_v2<br><br>Show the field ONLY if:<br>[male_pp_cntrl_age_type_v2(3)] = '1'	Median	text
	99	age_cntrl_male_range_v2<br><br>Show the field ONLY if:<br>[male_pp_cntrl_age_type_v2(4)] = '1'	Range	text
	100	female_cntrl_age_v2	Female Population	descriptive
	101	female_pp_cntrl_age_type_v2	How was the age of control participants reported?<br>*Select all relevant answers*	checkbox, Required<br>1 female_pp_cntrl_age_type_v2___1 Mean<br>2 female_pp_cntrl_age_type_v2___2 Standard deviation<br>3 female_pp_cntrl_age_type_v2___3 Median<br>4 female_pp_cntrl_age_type_v2___4 Range
	102	age_cntrl_female_mean_v2<br><br>Show the field ONLY if:<br>[female_pp_cntrl_age_type_v2(1)] = '1'	Mean	text
	103	age_cntrl_female_stdev_v2<br><br>Show the field ONLY if:<br>[female_pp_cntrl_age_type_v2(2)] = '1'	Standard deviation	text
	104	age_cntrl_female_median_v2<br><br>Show the field ONLY if:<br>[female_pp_cntrl_age_type_v2(3)] = '1'	Median	text
	105	age_cntrl_female_range_v2<br><br>Show the field ONLY if:<br>[female_pp_cntrl_age_type_v2(4)] = '1'	Range	text
	106	cntrl_anth_info_v2	Control Anthropometric Information	descriptive
	107	cntrl_anth_info_2_v2	Total Population	descriptive
	108	control_height_v2<br><br>Show the field ONLY if:<br>[number_of_controls_v2] = 1	Control height<br>*Meters*	text, Required
	109	control_mass_v2<br><br>Show the field ONLY if:<br>[number_of_controls_v2] = 1	Control mass<br>*Kilograms*	text, Required
	110	control_bmi_v2<br><br>Show the field ONLY if:<br>[number_of_controls_v2] = 1	Control body mass index<br>*Kilograms/meters^2*	text, Required
	111	male_cntrl_anth_info_v2	Male Population	descriptive
	112	female_cntrl_anth_info_v2	Female Population	descriptive
	113	injury_type_v2	Section Header: *Injury History*<br><br>Type of knee injury<br>*Select all relevant answers*	checkbox, Required<br>1 injury_type_v2___1 Intra-articular (general unspecified)<br>2 injury_type_v2___2 Ligament<br>3 injury_type_v2___3 Meniscus<br>4 injury_type_v2___4 Fracture<br>5 injury_type_v2___5 Dislocation<br>6 injury_type_v2___6 Contusion<br>7 injury_type_v2___7 Unclear

	114	time_since_injury_type_v2	How was time between injury incidence and observation/follow up reported?<br>*Select all relevant answers*	checkbox, Required<br>1 | time_since_injury_type_v2___1 | Mean<br>2 | time_since_injury_type_v2___2 | Standard deviation<br>3 | time_since_injury_type_v2___3 | Median<br>4 | time_since_injury_type_v2___4 | Range<br>5 | time_since_injury_type_v2___5 | Not reported
	115	time_since_injury_mean_v2<br>Show the field ONLY if: [time_since_injury_type_v2(1)] = '1'	Mean	text (number), Required
	116	time_since_injury_stdev_v2<br>Show the field ONLY if: [time_since_injury_type_v2(2)] = '1'	Standard deviation	text (number), Required
	117	time_since_injury_median_v2<br>Show the field ONLY if: [time_since_injury_type_v2(3)] = '1'	Median	text (number), Required
	118	time_since_injury_range_v2<br>Show the field ONLY if: [time_since_injury_type_v2(4)] = '1'	Range	text (number), Required
	119	knee_surgery_v2	Section Header: *Surgery History*<br>Did the participants have knee surgery?	yesno, Required<br>1 | Yes<br>0 | No
	120	surgery_type_v2<br>Show the field ONLY if: [knee_surgery_v2] = '1'	Type of knee surgery<br>*Select all relevant answers*	checkbox, Required<br>1 | surgery_type_v2___1 | Tendon graft<br>2 | surgery_type_v2___2 | Meniscectomy<br>3 | surgery_type_v2___3 | Chondroplasty<br>7 | surgery_type_v2___7 | Unclear
	121	time_since_surgery_type_v2<br>Show the field ONLY if: [knee_surgery_v2] = '1'	How was time between between surgery and observation/follow up reported?<br>*Select all relevant answers*	checkbox, Required<br>1 | time_since_surgery_type_v2___1 | Mean<br>2 | time_since_surgery_type_v2___2 | Standard deviation<br>3 | time_since_surgery_type_v2___3 | Median<br>4 | time_since_surgery_type_v2___4 | Range<br>5 | time_since_surgery_type_v2___5 | Not reported
	122	time_since_surgery_mean_v2<br>Show the field ONLY if: [time_since_surgery_type_v2 (1)] = 1	Mean	text, Required
	123	time_since_surgery_stdev_v2<br>Show the field ONLY if: [time_since_surgery_type_v2 (2)] = 1	Standard deviation	text, Required
	124	time_since_surgery_median_v2<br>Show the field ONLY if: [time_since_surgery_type_v2 (3)] = 1	Median	text, Required
	125	time_since_surgery_range_v2<br>Show the field ONLY if: [time_since_surgery_type_v2 (4)] = 1	Range	text, Required
	126	sport_type_v2	Section Header: *Sporting History*<br>What sport did the participants engage in at the time of injury?<br>*If not detailed state N/A*	text, Required
	127	sport_level_v2	What was the competition level at the time of injury?<br>*If not detailed state N/A*	text, Required

For peer review only - http://bmjopen.bmj.com/site/about/guidelines.xhtml

	128	was_there_intervention_v2	Section Header: *Intervention Details*<br>Was there an intervention?	yesno<br>1 Yes<br>0 No
	129	intervention_type_v2<br>Show the field ONLY if:<br>[was_there_intervention_v2] = 1	Intervention type<br>*Please detail the type of intervention*	notes, Required
	130	intervention_setting_v2<br>Show the field ONLY if:<br>[was_there_intervention_v2] = 1	Intervention setting<br>*E.g., home, hospital, physio clinic*	text, Required
	131	duration_of_intervention_v2<br>Show the field ONLY if:<br>[was_there_intervention_v2] = 1	Duration of intervention<br>*Months*	text (number), Required
	132	adherence_v2<br>Show the field ONLY if:<br>[was_there_intervention_v2] = 1	Adherence rate	text (number), Required
	133	drop_out_v2<br>Show the field ONLY if:<br>[was_there_intervention_v2] = 1	Drop out rate	text (number), Required
	134	independent_variable_v2	Independent Variables	descriptive
	135	what_did_the_study_measure_v2	What was the independent variable measuring?<br>*Select all relevant answers*	checkbox, Required<br>1 what_did_the_study_measure_v2___1 Physical activity<br>2 what_did_the_study_measure_v2___2 Systemic inflammation<br>3 what_did_the_study_measure_v2___3 Knee joint loading<br>4 what_did_the_study_measure_v2___4 Adiposity<br>5 what_did_the_study_measure_v2___5 Muscle size<br>6 what_did_the_study_measure_v2___6 Muscle strength<br>7 what_did_the_study_measure_v2___7 Intramuscular fat<br>8 what_did_the_study_measure_v2___8 Bone mineral density<br>9 what_did_the_study_measure_v2___9 Unclear (need to contact authors)
	136	physical_activity_tool_v2<br>Show the field ONLY if:<br>[what_did_the_study_measure_v2(1)] = 1	Please select the tool used to measure physical activity<br>*Select all relevant answers*	checkbox, Required<br>1 physical_activity_tool_v2___1 Doubly labelled water<br>2 physical_activity_tool_v2___2 Accelerometers<br>3 physical_activity_tool_v2___3 Pedometers<br>4 physical_activity_tool_v2___4 Subjective questionnaires<br>5 physical_activity_tool_v2___5 Diaries<br>6 physical_activity_tool_v2___6 Other (please state below)<br>7 physical_activity_tool_v2___7 Unclear (need to contact authors)
	137	subjective_pa_tool_type_v2<br>Show the field ONLY if:<br>[physical_activity_tool_v2(4)] = '1'	What was the name of the subjective assessment tool?<br>*Please use full name, not abbreviation. E.g., International Physical Activity Questionnaire*	text, Required

	138	inflammation_tool_v2<br>Show the field ONLY if:<br>[what_did_the_study_measure_v2(2)] = '1'	Which cytokine/protein(s) were used to measure systemic inflammation?<br>*Select all relevant answers*	checkbox, Required<br>1 inflammation_tool_v2___1 Interleukin 6 (IL-6)<br>2 inflammation_tool_v2___2 Interleukin 1Beta (IL-1β)<br>3 inflammation_tool_v2___3 Tumor Necrosis Factor Alpha (TNF-α)<br>4 inflammation_tool_v2___4 C-Reactive Protein (CRP)<br>5 inflammation_tool_v2___5 Other (please state below)<br>6 inflammation_tool_v2___6 Unclear (need to contact authors)
	139	knee_load_tool_v2<br>Show the field ONLY if:<br>[what_did_the_study_measure_v2(3)] = '1'	What tool(s) used to measure knee joint loading?<br>*Select all relevant answers*	checkbox, Required<br>1 knee_load_tool_v2___1 Ground reaction force<br>2 knee_load_tool_v2___2 Moments<br>3 knee_load_tool_v2___3 Inverse dynamics<br>4 knee_load_tool_v2___4 Forward dynamics<br>5 knee_load_tool_v2___5 Other (please state below)<br>6 knee_load_tool_v2___6 Unclear (need to contact authors)
	140	adiposity_tool_v2<br>Show the field ONLY if:<br>[what_did_the_study_measure_v2(4)] = '1'	What was the tool to measure adiposity?	checkbox, Required<br>1 adiposity_tool_v2___1 Body Mass Index (BMI)<br>2 adiposity_tool_v2___2 Air displacement plethysmograph (ADP)<br>3 adiposity_tool_v2___3 Bioelectrical impedance (BI)<br>4 adiposity_tool_v2___4 Dual energy x-ray absorptiometry (DEXA or DXA)<br>5 adiposity_tool_v2___5 Computerised tomography (CT)<br>6 adiposity_tool_v2___6 Peripheral computerised tomography (pQCT)<br>7 adiposity_tool_v2___7 Other (please state below)<br>8 adiposity_tool_v2___8 Unclear (need to contact authors)
	141	muslce_tool_v2	What was the tool used to measure muscle size?	checkbox<br>1 muslce_tool_v2___1 Ultrasound (US)<br>2 muslce_tool_v2___2 Dual energy x-ray absorptiometry (DEXA or DXA)<br>3 muslce_tool_v2___3 Peripheral quantitative computerised tomography (pQCT)<br>4 muslce_tool_v2___4 Computerised tomography (CT)<br>5 muslce_tool_v2___5 Magnetic resonance imaging (MRI)<br>6 muslce_tool_v2___6 Other (please state below)<br>7 muslce_tool_v2___7 Unclear (need to contact authors)
	142	muscle_strength_tool_v2<br>Show the field ONLY if:<br>[what_did_the_study_measure_v2(5)] = '1'	What was the tool to measure muscle strength?	checkbox, Required<br>1 muscle_strength_tool_v2___1 Isokinetic dynamometry<br>2 muscle_strength_tool_v2___2 Hand-held dynamometer<br>3 muscle_strength_tool_v2___3 Other (please state below)<br>4 muscle_strength_tool_v2___4 Unclear (need to contact authors)

For peer review only - http://bmjopen.bmj.com/site/about/guidelines.xhtml

	143	intra_muscular_fat_tool_v2<br>Show the field ONLY if:<br>[what_did_the_study_measure_v2(6)] = '1'	What was the tool to measure intra-muscular fat?	checkbox, Required<br>1 intra_muscular_fat_tool_v2___1 Ultrasound (US)<br>2 intra_muscular_fat_tool_v2___2 Computerised tomography (CT)<br>3 intra_muscular_fat_tool_v2___3 Peripheral quantitative computerised tomography (pQCT)<br>4 intra_muscular_fat_tool_v2___4 Magnetic resonance imaging (MRI)<br>5 intra_muscular_fat_tool_v2___5 Other (please state below)<br>6 intra_muscular_fat_tool_v2___6 Unclear (need to contact authors)
	144	bone_mineral_density_tool_v2<br>Show the field ONLY if:<br>[what_did_the_study_measure_v2(7)] = '1'	What was the tool to measure bone mineral density?	checkbox, Required<br>1 bone_mineral_density_tool_v2___1 Dual energy x-ray absorptiometry (DEXA or DXA)<br>2 bone_mineral_density_tool_v2___2 Computerised tomography (CT)<br>3 bone_mineral_density_tool_v2___3 Peripheral quantitative computerised tomography (pQCT)<br>4 bone_mineral_density_tool_v2___4 Other (please state below)<br>5 bone_mineral_density_tool_v2___5 Unclear (need to contact authors)
	145	ptoa_disease_head_v2	Section Header: *Outcome Variables*<br>PTOA Disease	descriptive
	146	ptoa_disease_v2	Does the study measure PTOA disease?	yesno<br>1 Yes<br>0 No
	147	radiography_diagnostic_v2	Radiography diagnostic system	checkbox, Required<br>1 radiography_diagnostic_v2___1 Kellgren and Lawrence (K/L) system<br>2 radiography_diagnostic_v2___2 Osteoarthritis Research Society International (OARSI) Atlas criteria<br>3 radiography_diagnostic_v2___3 Unclear (need to contact authors)
	148	mri_diagnostic_v2	Magnetic resonance imaging diagnostic system	checkbox, Required<br>1 mri_diagnostic_v2___1 MRI Osteoarthritis Knee Score (MOAKS)<br>2 mri_diagnostic_v2___2 Whole Organ Magnetic Resonance Imaging Score (WORMS)<br>3 mri_diagnostic_v2___3 Boston Leeds OA Knee Score (BLOKS)<br>4 mri_diagnostic_v2___4 Unclear (need to contact authors)

	149	indicators_of_disease_v2	Indicators of structural deterioration preceding or exacerbating PTOA disease	checkbox, Required<br>1 | indicators_of_disease_v2___1 | MRI quantified cartilage thickness and volume<br>2 | indicators_of_disease_v2___2 | T1rho mapping<br>3 | indicators_of_disease_v2___3 | T2 mapping<br>4 | indicators_of_disease_v2___4 | Delayed contrast-enhanced MRI of cartilage (dGEMRIC)<br>5 | indicators_of_disease_v2___5 | Unclear (need to contact authors)
	150	systemic_biomarkers_v2	Systemic biomarkers indicating structural deterioration of knee PTOA disease	checkbox<br>1 | systemic_biomarkers_v2___1 | Matrix Metalloprotease 3 (MMP-3)<br>2 | systemic_biomarkers_v2___2 | Matrix Metalloprotease 13 (MMP-13)<br>3 | systemic_biomarkers_v2___3 | N-Propeptide of Collagen IIA (PIIANP)<br>4 | systemic_biomarkers_v2___4 | C-Telopeptide of Type II Collagen (CTX)<br>5 | systemic_biomarkers_v2___5 | C-Propeptide of Type II Collagen (CP-II)<br>6 | systemic_biomarkers_v2___6 | CTX:CP-II<br>7 | systemic_biomarkers_v2___7 | Collagen Type II Cleavage Product (C2C)<br>8 | systemic_biomarkers_v2___8 | Col2-3/4 C-terminal cleavage product of types I and II collagen (C1,2C)<br>9 | systemic_biomarkers_v2___9 | Hyaluronic Acid (HA)<br>10 | systemic_biomarkers_v2___10 | Chondroitin Sulphate 846 Epitope (CS846)<br>11 | systemic_biomarkers_v2___11 | Aggrecan164 or Aggrecan Fragments (ARGS)<br>12 | systemic_biomarkers_v2___12 | Cartilage Oligomeric Matrix Protein (COMP)<br>13 | systemic_biomarkers_v2___13 | Unclear (need to contact authors)
	151	outcome	Section Header: *Results*<br>Outcome 1<br>*Primary Outcome*	descriptive
	152	outcome_name	Please state what the independent variable is (e.g., physical activity), how it is measured (e.g., accelerometery), and the make/model of the measurement tool.	notes
	153	iv_name	Please state what the outcome variable is (e.g., c-telopeptide of type II collagen), how it is measured (e.g., blood serum), and the make/model of the measurement tool.	notes
	154	sample_size	Sample size for the test	text

	155	stat_test_1	What was the type of statistical test used?	radio<br>1 Difference test (t-test, ANOVA)<br>2 Correlation<br>3 Regression<br>4 Odds Ratio<br>5 Risk Ratio
	156	outcome_mean	Injured mean	text
	157	outcome_stdev	Injured standard deviation	text
	158	control_mean	Control mean	text
	159	cntrl_outcome_stdev	Control standard deviation	text
	160	p_correlation_coefficient	Pearson correlation coefficient	text
	161	s_correlation_coefficient	Spearman's correlation coefficient	text
	162	odds_ratio	Odds ratio	text
	163	risk_ratio	Risk ratio	text
	164	outcome_2	Outcome 2<br>*Primary Outcome*	descriptive
	165	outcome_name_2	Please state what the independent variable is (e.g., physical activity), how it is measured (e.g., accelerometery), and the make/model of the measurement tool.	notes
	166	iv_name_2	Please state what the outcome variable is (e.g., c-telopeptide of type II collagen), how it is measured (e.g., blood serum), and the make/model of the measurement tool.	notes
	167	sample_size_2	Sample size for the test	text
	168	stat_test_2	What was the type of statistical test used?	radio<br>1 Difference test (t-test, ANOVA)<br>2 Correlation<br>3 Regression<br>4 Odds Ratio<br>5 Risk Ratio
	169	outcome_mean_2	Injured mean	text
	170	outcome_stdev_2	Injured standard deviation	text
	171	control_mean_2	Control mean	text
	172	cntrl_outcome_stdev_2	Control standard deviation	text
	173	p_correlation_coefficient_2	Pearson correlation coefficient	text
	174	s_correlation_coefficient_2	Spearman's correlation coefficient	text
	175	odds_ratio_2	Odds ratio	text
	176	risk_ratio_2	Risk ratio	text
	177	outcome_3	Outcome 3<br>*Primary Outcome*	descriptive
	178	outcome_name_3	Please state what the independent variable is (e.g., physical activity), how it is measured (e.g., accelerometery), and the make/model of the measurement tool.	notes
	179	iv_name_3	Please state what the outcome variable is (e.g., c-telopeptide of type II collagen), how it is measured (e.g., blood serum), and the make/model of the measurement tool.	notes
	180	sample_size_3	Sample size for the test	text
	181	stat_test_3	What was the type of statistical test used?	radio<br>1 Difference test (t-test, ANOVA)<br>2 Correlation<br>3 Regression<br>4 Odds Ratio<br>5 Risk Ratio
	182	outcome_mean_3	Injured mean	text
	183	outcome_stdev_3	Injured standard deviation	text
	184	control_mean_3	Control mean	text
	185	cntrl_outcome_stdev_3	Control standard deviation	text

For peer review only - http://bmjopen.bmj.com/site/about/guidelines.xhtml

	186	p_correlation_coefficient_3	Pearson correlation coefficient	text
	187	s_correlation_coefficient_3	Spearman's correlation coefficient	text
	188	odds_ratio_3	Odds ratio	text
	189	risk_ratio_3	Risk ratio	text
	190	conclusion01	Section Header: *Conclusion*<br>Please state the author's conclusions	notes
	191	other_comments	Please state any comments/conclusions you have	notes
	192	data_extraction_2_complete	Section Header: *Form Status*<br>Complete?	dropdown<br>0 Incomplete<br>1 Unverified<br>2 Complete

For peer review only

Supplementary Material 8. Strength of the treatment response or relationship between variables

Interpreting effect size values[1,2,3,4]

Effect Size Measure	Effect Size			
	**Small**	**Medium**	**Large**	**Very Large**
Odds Ratio	1.5	2.5	4	10
Cohen's *d*	0.2	0.5	0.8	1.3
r	0.1	0.3	0.5	0.7
Cohen's *f*	0.1	0.25	0.4	-
Eta-squared	0.01	0.06	0.14	-

1. Maher JM, Markey JC, Ebert-May D. The Other Half of the Story: Effect Size Analysis in Quantitative Research. *CBE Life Sci Educ* 2013;12(3):345-51. doi: 10.1187/cbe.13-04-0082

2. Rosenthal JA. Qualitative Descriptors of Strength of Association and Effect Size. J Soc Serv Res 1996;21(4):37-59. doi: 10.1300/j079v21n04_02

3. Cohen J. A power primer. Psychological bulletin 1992;112(1):155.

4. Cohen J. Statistical Power Analysis for the Behavioral Sciences. 2nd Edition ed. New York: Routledge 1988.

For peer review only

Supplementary Material 9. Adapted Grading of Recommendations, Assessment, Development and Evaluations (GRADE) Guidelines for Scoping Review

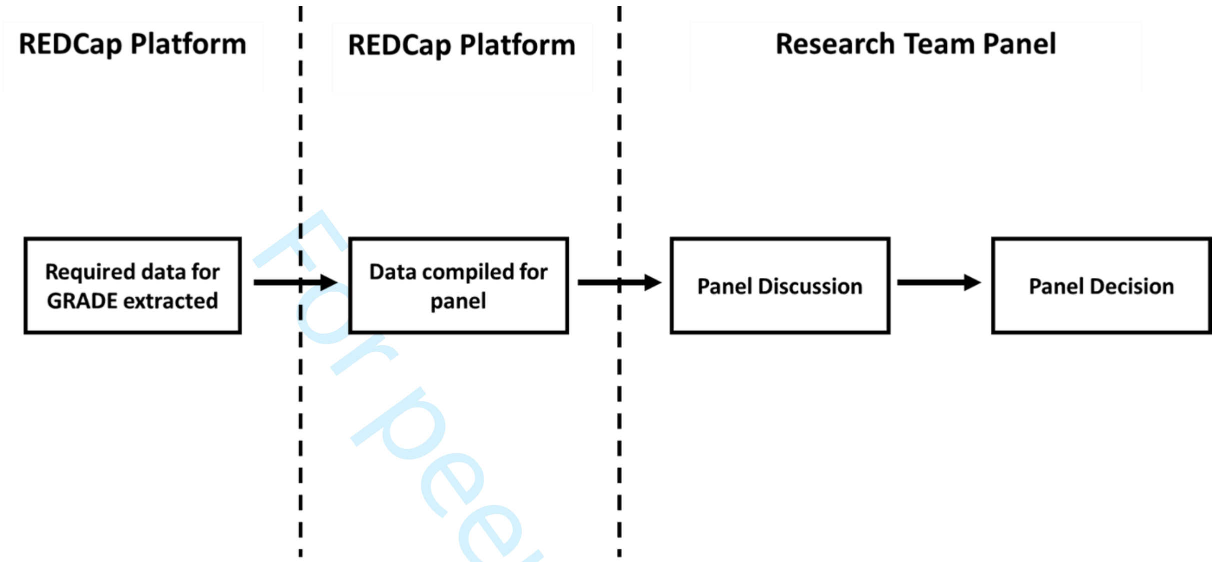

For peer review only

Draft - Adapted Grading of Recommendations, Assessment, Development and Evaluations (GRADE) Guidelines for Scoping Review

Overview:

Grading of Recommendations, Assessment, Development and Evaluations (GRADE)[1-3] is structured as:

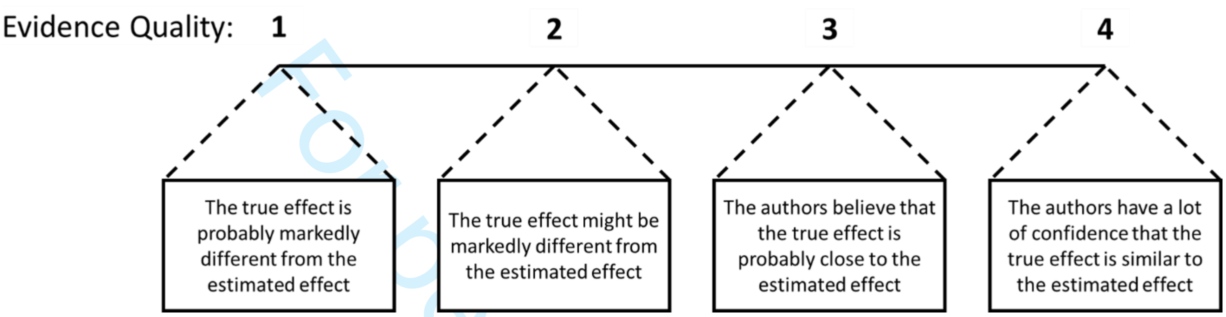

In GRADE, randomised control trials (RCTs) are the highest quality of research evidence (4), whilst observational studies (e.g., analytical cross-sectional studies) are the lowest form of evidence (1).[1-3] From there, individual studies will either be downgraded or upgraded for quality of evidence. For example, if an RCT is not downgraded it will provide the highest quality of evidence (4).[1-3]

In this review, the GRADE criteria will be applied to the research question '***what is the role of physical activity in the trajectory from intra-articular knee injury to post-traumatic osteoarthritis in young men and women?***' and will be considered through each of the following themes:

- Physical activity
- Systemic inflammation
- Adiposity
- Knee joint loading
- Lower body muscle strength
- Muscle size
- Intra-muscular fat
- Bone mineral density

Downgrade quality of evidence

1. Risk of Bias (RoB)

According to Ma *et al.*,[4] "Quality includes internal and external validity, while methodological quality usually refers to internal validity. Internal validity is also recommended as "risk of bias (RoB)" by the Cochrane Collaboration."

In this review, we will use the Joanna Briggs Institute (JBI) Critical Appraisal Checklists (Supplementary Material 6) to assess the RoB of each individual study. The Critical Appraisal Checklists apply to:

- Analytical Cross Sectional Studies[5]
- Case Control Studies[5]
- Cohort Studies[5]
- Studies Reporting Prevalence Data[6]
- Quasi-Experimental Studies[7]
- Randomised Control Trials[7]
- Systematic Review and Research Synthesis[8]

For the purpose of this review, we will classify the scores generated from JBI Critical Appraisal Checklists as either:

- Very low quality (0 – 25.0%)
- Low quality (>25.0 – 50.0%)
- Moderate quality (>50.0 – 75.0%)
- High quality (75.0 – 100%)

This scoring system will be applied to studies on a case-by-case basis.

Following application of the scoring system to all studies, we will adopt GRADE guidelines by downgrading studies if, collectively for the outcome, there is either a serious risk of bias or a very serious risk of bias. The scoring criteria for this review will be as follows:

- (**0**) No serious risk of bias overall = all studies scored moderate to high quality
- (**-1**) Serious risk of bias overall = more than 25% of studies considered low or very low quality
- (**-2**) Very serious risk of bias overall = More than 50% of studies considered low or very low quality

2. Inconsistency

Study results will be considered consistent when directions (e.g., positive or negative association), are considered similar enough to draw uniform conclusions. In this review, consistency in direction will be defined as >75% of the included studies for each theme demonstrating the same positive effect only or negative effect only.

For peer review only

- (**-1**) Serious risk of inconsistency when ≤75% of the included studies have the same direction of effect.
- (**-2**) Very serious risk of inconsistency when there is only one or no studies.

3. Indirectness

GRADE distinguishes several types of indirectness[9]. In this review, indirectness may be due to any of the factors detailed below:

Independent variable (*applicability*)[9]

- (**-1**) Serious risk of indirectness when the independent variable typically used by studies is deemed less valid than the gold standard (e.g., adiposity inferred from body mass index rather than dual-energy x-ray absorptiometry)
- (**-1**) Serious risk of indirectness an intervention cannot be completed in multiple settings (e.g., hospital and home based)

If no studies test a relevant intervention to the research question, or there is no research investigating any of the identified themes, we will conclude that the effects are uncertain.

Differences in outcome measures (*surrogate outcomes*)[9]

- (**-1**) Serious risk of indirectness when research typically uses of a surrogate measure which is associated with knee PTOA (e.g., systemic concentrations of type-II collagen biomarker), rather than directly diagnosing PTOA
- (**-1**) Serious risk of indirectness when studies do not provide information on full time course of PTOA development (e.g., only short-term effects of a physical activity intervention on a surrogate provide, and no studies investigating the long-term impact of PTOA diagnosis)

Indirect comparison[9]

- (**-1**) Serious risk of indirectness by lack of comparisons between physical activity types or exercise modalities
- (**-1**) Serious risk of indirectness by lack of comparison to alternative approaches (e.g., pharmaceutical driven pain relief)

4. Imprecision

As the data will not be combined in a meta-analysis, the following qualitative assessment will be applied to determine imprecision[10]:

- (**-1**) Serious risk of imprecision when the lower threshold of 95% CI reduces the effect to negligible

For peer review only

- (**-1**) Serious risk of imprecision when effect of estimate presented in only one or two studies or estimate comes from one or two events

- (**-2**) Very serious risk of imprecision if the lower threshold of 95% CI is consistent with conflicting conclusions

- (**-2**) Very serious risk of imprecision if CIs were not provided in any of the studies

5. Publication Bias

Assessment of publication bias using statistical methods or funnel plots will not be possible due to heterogeneity of the studies in this review. Publication bias will be screened for qualitatively as detailed in Chapter 7 of the Cochrane Handbook.

For peer review only

Upgrade quality of evidence

1. Large Magnitude of Effect

In this review, when a very large magnitude of effect is observed (assessed using Supplementary Material 8), there is greater certainty that there is at least a small effect.[11]

- (**+1**) when the effect size is large
- (**+2**) when the effect size is very large

The time-course of outcomes will also be considered in this review[12] when upgrading due to large magnitude of effect and devised into:

- (**+1**) Short-term (e.g., response to single exercise session/alteration of joint loading)
    - Systemic biomarkers in synovial fluid/serum/plasma/urine
    - MRI or US indicates deterioration
    - Symptoms or function

- (**+1**) Medium-term (e.g., response to 12-week activity intervention)
    - Systemic biomarkers in synovial fluid/serum/plasma/urine
    - MRI or US indicates deterioration
    - Symptoms or function

- (**+2**) Long-term (e.g., Body Mass Index at one-year post-injury and PTOA development in the years following)
    - X-Ray or MRI PTOA Diagnosis

This is because biomarkers concentrations changes can occur rapidly due to mechanical compression,[13-16] or an exercise-based intervention.[17] Furthermore, the basal protein synthesis rates in various musculoskeletal tissues (including cartilage, meniscus, bone) are within the same range of skeletal muscle protein synthesis rates[18] which may indicate pathology much faster than it takes for PTOA to fully develop.

Lastly, if there are major concerns regarding risk of bias, consistency, precision, or publication bias, in addition to being downgraded, the quality of evidence will not be upgraded via magnitude of effect.[12]

2. Dose-Response Gradient

This increases our confidence in the findings of observational studies and thus enhance the assigned quality of evidence.[12] This is best represented by RCTs and quasi-experimental trials.

- (**+1**) when there is evidence of a dose (e.g., physical activity-based intervention) and response (e.g., increased concentrations of aggrecan

For peer review only

synthesis, lower concentrations of type-II collagen breakdown, slower MRI defined joint tissue degeneration)

3. All residual confounding would decrease magnitude of effect (in situations with an effect)

All plausible residual confounders or biases would reduce a demonstrated effect, or suggest a spurious effect when results show no effect.[12]

REFERENCES

1. Guyatt GH, Oxman AD, Kunz R, et al. What is "quality of evidence" and why is it important to clinicians? *BMJ* 2008;336(7651):995-98. doi: 10.1136/bmj.39490.551019.be
2. Guyatt GH, Oxman AD, Vist GE, et al. GRADE: an emerging consensus on rating quality of evidence and strength of recommendations. *BMJ* 2008;336(7650):924-26. doi: 10.1136/bmj.39489.470347.ad
3. Guyatt G, Oxman AD, Akl EA, et al. GRADE guidelines: 1. Introduction-GRADE evidence profiles and summary of findings tables. *J Clin Epidemiol* 2011;64(4):383-94. doi: 10.1016/j.jclinepi.2010.04.026 [published Online First: 20101231]
4. Ma L-L, Wang Y-Y, Yang Z-H, et al. Methodological quality (risk of bias) assessment tools for primary and secondary medical studies: what are they and which is better? *Mil Med Res* 2020;7(1) doi: 10.1186/s40779-020-00238-8
5. Moola S, Munn Z, Tufanaru C, et al. Chapter 7: Systematic reviews of etiology and risk. In: Aromataris E, Munn Z, eds. JBI Manual for Evidence Synthesis: JBI, 2020.
6. Munn Z, Moola S, Lisy K, et al. Methodological guidance for systematic reviews of observational epidemiological studies reporting prevalence and cumulative incidence data. *Int J Evid Based Healthc* 2015;13(3):147-53. doi: 10.1097/XEB.0000000000000054
7. Tufanaru C, Munn Z, Aromataris E, et al. Chapter 3: Systematic reviews of effectiveness. In: Aromataris E, Munn Z, eds. JBI Manual for Evidence Synthesis: JBI, 2020.
8. Aromataris E, Fernandez R, Godfrey CM, et al. Summarizing systematic reviews: methodological development, conduct and reporting of an umbrella review approach. *Int J Evid Based Healthc* 2015;13(3):132-40. doi: 10.1097/XEB.0000000000000055
9. Guyatt GH, Oxman AD, Kunz R, et al. GRADE guidelines: 8. Rating the quality of evidence—indirectness. *J Clin Epidemiol* 2011;64(12):1303-10. doi: 10.1016/j.jclinepi.2011.04.014
10. Guyatt GH, Oxman AD, Kunz R, et al. GRADE guidelines 6. Rating the quality of evidence—imprecision. *J Clin Epidemiol* 2011;64(12):1283-93. doi: 10.1016/j.jclinepi.2011.01.012

For peer review only

11. Reed S, Guyatt G. What is GRADE? BMJ: BMJ; 2022 [Available from: https://bestpractice.bmj.com/info/toolkit/learn-ebm/what-is-grade/ accessed 20/06/2022 2022.
12. Guyatt GH, Oxman AD, Sultan S, et al. GRADE guidelines: 9. Rating up the quality of evidence. *J Clin Epidemiol* 2011;64(12):1311-16. doi: 10.1016/j.jclinepi.2011.06.004
13. Mundermann A, Dyrby CO, Andriacchi TP, et al. Serum concentration of cartilage oligomeric matrix protein (COMP) is sensitive to physiological cyclic loading in healthy 9adults. *Osteoarthritis Cartilage* 2005;13(1):34-38. doi: 10.1016/j.joca.2004.09.007 [published Online First: 2005/01/11]
14. Mündermann A, King KB, Smith RL, et al. Change in serum COMP concentration due to ambulatory load is not related to knee OA status. *J Orthop Res* 2009;27(11):1408-13. doi: 10.1002/jor.20908 [published Online First: 2009/05/08]
15. Mundermann A, Klenk C, Billich C, et al. Changes in Cartilage Biomarker Levels During a Transcontinental Multistage Footrace Over 4486 km. *Am J Sports Med* 2017;45(11):2630-36. doi: 10.1177/0363546517712945 [published Online First: 2017/06/27]
16. Roberts HM, Law RJ, Thom JM. The time course and mechanisms of change in biomarkers of joint metabolism in response to acute exercise and chronic training in physiologic and pathological conditions. *Eur J Appl Physiol* 2019;119(11-12):2401-20. doi: 10.1007/s00421-019-04232-4 [published Online First: 2019/10/28]
17. Azukizawa M, Ito H, Hamamoto Y, et al. The effects of well-rounded exercise program on systemic biomarkers related to cartilage metabolism. *Cartilage* 2019;10(4):451-58. doi: 10.1177/1947603518767998 [published Online First: 2018/04/13]
18. Smeets JSJ, Horstman AMH, Vles GF, et al. Protein synthesis rates of muscle, tendon, ligament, cartilage, and bone tissue in vivo in humans. *PLoS One* 2019;14(11):e0224745. doi: 10.1371/journal.pone.0224745 [published Online First: 2019/11/08]

For peer review only
